# Abstract Supplement Abstracts from IAS 2023, the 12th IAS Conference on HIV Science, 23 – 26 July, Brisbane, Australia & Virtual

**DOI:** 10.1002/jia2.26134

**Published:** 2023-07-23

## ORAL ABSTRACT

### OAA0102 Resident microbiota enhance HIV acquisition, replication and pathogenesis in vivo

A. Wahl^1^, W. Yao^1^, B. Liao^1^, M. Chateau^1^, C. Richardson^1^, L. Ling^1^, J. Tucker^1^, I. McGowan^2^, R.B. Sartor^1^, J.V. Garcia ^1^

^1^University of North Carolina at Chapel Hill, Chapel Hill, United States, ^2^University of Pittsburgh Medical School, Pittsburgh, United States

**Background**: Resident microbiota maintain intestinal homeostasis by regulating digestion, metabolism, immune development and providing protection from infection. Pioneering work has also shown that resident microbiota enhance the transmission of a limited number of viruses that target the intestinal tract. The intestinal tract is a major site of HIV infection. However, the effect of resident microbiota on HIV acquisition, replication and pathogenesis in the intestinal tract is unknown due to the strict species tropism of HIV.

**Methods**: Bone marrow/liver/thymus (BLT) humanized mice have been extensively utilized to study HIV acquisition, pathogenesis and prevention strategies in vivo. To examine the role of resident microbiota in HIV acquisition and infection, we constructed germ‐free (GF) BLT mice and BLT mice colonized with resident intestinal microbiota. First, we rederived a GF strain of immune deficient mice by sterile embryo transfer. GF‐BLT humanized mice were then surgically constructed in a sterile gnotobiotic surgical isolator. All GF mice were housed and experiments performed in sterile gnotobiotic isolators and the GF status of mice confirmed longitudinally. BLT mice colonized with resident microbiota were also constructed. We then evaluated HIV acquisition, replication and pathogenesis in GF BLT mice and BLT mice colonized with resident microbiota following an oral or rectal HIV exposure. HIV‐RNA levels were monitored longitudinally in the peripheral blood plasma of mice weekly by real‐time PCR analysis. At necropsy, we measured the levels of HIV‐DNA, HIV‐RNA, and T‐cell activation blood and tissues.

**Results**: Our results show that HIV acquisition, replication and pathogenesis in the intestinal tract is greatly enhanced in the presence of resident microbiota. HIV acquisition was 300% higher following an oral HIV challenge (*p* = 0.013) and 200% higher following a rectal HIV challenge (*p* = 0.0286) in the presence of resident microbiota. Mean plasma viral loads were up to 34‐fold higher and cell‐associated HIV‐RNA levels in tissue were also over a 1000‐fold higher in the presence of resident microbes. Furthermore, HIV‐associated CD8^+^ T‐cell activation was higher in the intestinal tract in the presence of resident microbiota.

**Conclusions**: These data directly demonstrate for the first time that resident microbiota are a major driver of HIV acquisition, replication and pathogenesis.

### OAA0103 Gut dysfunction as an independent driver of neuroinflammation in ART‐suppressed SIV

S. Byrnes ^1^, K. Busman‐Sahay^2^, T. Angelovich^1^, S. Younger^2^, S. Taylor‐Brill^2^, M. Nekorchuk^2^, S. Bondoc^2^, R. Dannay^2^, M. Terry^2^, C. Cochrane^1^, T. Jenkins^1^, M. Roche^3^, C. Deleage^4^, S. Bosinger^5^, M. Paiardini^5^, B. Brew^6^, J. Estes^2^, M. Churchill^1^

^1^RMIT, Melbourne, Australia, ^2^OHSU, Portland, United States, ^3^University of Melbourne, Melbourne, Australia, ^4^Frederick National Laboratory for Cancer Research, Frederick, United States, ^5^Emory, Atlanta, United States, ^6^UNSW, Sydney, Australia

**Background**: Currently ∼30% of people with HIV (PWH) who are virally suppressed with antiretroviral therapy (ART) develop a form of HIV‐associated neurocognitive disorder (HAND). The presence of a viral reservoir (and associated neuroinflammation) in the brain and ongoing systemic inflammation penetrating the central nervous system (CNS) are thought to play crucial roles. However, the precise effects of viral mediated and/or independent pathways on the brain remain ill‐defined.

**Methods**: Here, we characterized the CNS reservoir and immune environment of SIV‐infected (SIV+) rhesus macaques (RMs) during acute (*n* = 4), chronic (*n* = 12) or ART‐suppressed SIV infection (*n* = 11) using spatial multiplex immunofluorescence analyses. Specifically, measures of SIV vRNA/DNA, blood−brain barrier integrity and ongoing immune activation/inflammation were quantified at a cellular level in the brain and matched gut tissue of SIV‐infected animals. Furthermore, immune activation/inflammation was also measured in a novel model of gut damage in SIV uninfected RMs (*n* = 4) to understand whether gut damage alone can drive neuroinflammation independent of SIV.

**Results**: SIV DNA+ and RNA+ cells were detected in the brain and gut tissue of all SIV+ groups tested. Additionally, the frequency of vDNA+ cells was not reduced in the brain or gut in ART‐treated animals (*p*<0.05), supporting the presence of a stable viral reservoir in the brain. SIV+ animals had enhanced inflammatory responses, expansion of activated astrocytes and myeloid cells as well as reduced BBB integrity compared to uninfected animals, which persisted despite ART (*p*<0.05 for all). Surprisingly, BBB breakdown and neuroinflammation correlated strongly with measures of gut inflammation, but not brain viremia. Similar immune activation profiles were present in the brains of the SIV uninfected gut damage model group, indicating that damage to the gut can contribute to immune activation in the brain independent of SIV‐infection.

**Conclusions**: Here, we show that ART‐suppressed SIV+ RMs have BBB breakdown and heightened activation of astrocytes and myeloid cells which is associated in part with SIV vDNA and immune activation in the gut and not resolved by ART treatment. These findings provide the strongest evidence to date that the brain of SIV‐infected animals remains in an activated state despite long‐term ART treatment and that gut damage can induce neuroimmune activation.

### OAA0104 Gut memory CD4^+^ T cells from people with HIV on suppressive ART express high levels of intracellular HIV sensors

A. George ^1,2^, J. Neidleman^1,2^, X. Luo^1,2^, J. Frouard^1,2^, K. Yin^1,2^, K. Young^1,2^, T. Ma^1,2^, A. Chaillon^3^, M. Porrachia^3^, B. Woodworth^3^, D. Smith^3^, S. Gianella^3^, N. Roan^1,2^

^1^Gladstone Institute of Virology, San Francisco, United States, ^2^University of California San Francisco, Department of Urology, San Francisco, United States, ^3^University of California San Diego, Division of Infectious Diseases and Global Public Health, La Jolla, United States

**Background**: Memory CD4^+^ T cells (CD4^+^ Tm) are major targets of HIV infection. Despite antiretroviral therapy (ART), HIV persists in tissues, with the gastrointestinal tract harbouring a major proportion of the reservoir. Here, we test whether CD4^+^ Tm from different tissue sites differentially express viral sensors with potential roles in detecting, restricting or responding to HIV infection.

**Methods**: We developed a CyTOF panel designed to quantitate 19 intracellular viral sensors. To determine if sensor expression differs across tissues, we applied our CyTOF panel on freshly isolated CD4^+^ Tm from eight tissue sites (gastrointestinal tract, lymph node, spleen, liver, kidney, bone marrow, heart and lung) harvested by rapid research autopsy from five people with HIV (PWH) on suppressive ART enrolled in the Last Gift Cohort. Last Gift is an end‐of‐life cohort of PWH that were diagnosed with a terminal illness, and who donate their bodies for HIV cure research.

**Results**: The gastrointestinal tract uniquely harboured a sub‐population of CD4^+^ Tm cells co‐expressing high levels of multiple sensors targeting viral components from multiple stages of the HIV replication cycle. These sensors included PQBP1 and MX2 which sense incoming viral capsid protein, STING and IRF3 which indirectly sense HIV DNA, SLFN11 which senses HIV RNA and inhibits viral protein synthesis, and MX1 which responds to type I interferons induced as a result of viral sensing. This sub‐population of CD4^+^ Tm was absent in the other tissues. As the gut is a main site of HIV persistence during ART suppression, elevated viral sensor expression at this site may reflect an ongoing detection of viral components produced by the persistent HIV reservoir. Consistent with this observation, in vitro infection assays revealed that infection of CD4^+^ Tm upregulated the expression of these same viral sensors.

**Conclusions**: CD4^+^ Tm express a diverse array of intracellular host factors capable of detecting and responding to the presence of HIV in a tissue‐dependent manner, with particularly high levels in the gastrointestinal tract, a major site of HIV persistence. The constant sensing of HIV components in the gastrointestinal tract may contribute to ongoing low levels of chronic inflammation in PWH.

### OAA0105 Are moto‐neurons susceptible to SARS‐CoV‐2 infection?

G. Cappelletti ^1^, C. Colombrita^2^, F. Limanaqi^1,3^, S. Strizzi^1^, C. Vanetti^1^, M. Garziano^1,3^, D. Trabattoni^1^, S. Santangelo^2,4^, V. Silani^2,5^, A. Ratti^2,4^, M. Biasin^1^

^1^University of Milan, Department of Biomedical and Clinical Sciences, Milan, Italy, ^2^IRCCS Istituto Auxologico Italiano, Department of Neurology and Laboratory of Neuroscience, Milan, Italy, ^3^University of Milan, Department of Pathophysiology and Transplantation, Milan, Italy, ^4^University of Milan, Department of Medical Biotechnology and Translational Medicine, Milan, Italy, ^5^University of Milan, Department of Pathophysiology and Transplantation, Dino Ferrari Center, Milan, Italy

**Background**: COVID‐19 typically causes respiratory disorders, but surprisingly a high proportion of patients also reported CNS symptoms as well as myopathies during and after SARS‐CoV‐2 infection. Notwithstanding, the impact of SARS‐CoV‐2 exposure on motor neuronal cells has not been investigated so far. Thus, by using human iPSC‐derived motor neurons (MNs), we assessed: their infectability by SARS‐CoV‐2; the expression of SARS‐CoV‐2 main receptors; and the effect of SARS‐CoV‐2 exposure on iPSC‐MN transcriptome.

**Methods**: Human iPSC lines from healthy donors were obtained by reprogramming fibroblasts and MNs were obtained by iPSC differentiation. The expression of the main SARS‐CoV‐2 human‐receptors was assessed in MNs by PCR and immunofluorescence (IF). MNs were in vitro infected with SARS‐CoV‐2, and viral replication was assessed by qPCR on two viral targets (N1 and N2) in cell culture supernatants at 24, 48 and 72 hours post infection (hpi). To confirm the results obtained, these same supernatants were used to re‐infect susceptible VeroE6 cells. Viral infection was monitored by IF and Qgene expression as well. In parallel, we profiled the gene expression of 46 different targets involved in viral entry as well as antiviral and immune response in SARS‐CoV‐2‐infected MNs.

**Results**: Gene expression profiling of the main receptors recognized by SARS‐CoV‐2 revealed that all of them are expressed with a lower level of ACE2 compared to CD147 and NRP1. By analysing N1 and N2 gene expressions over time, we observed that human iPSC‐MNs were productively infected by SARS‐CoV‐2, although viral replication was not accompanied by cytopathic effect. Supernatant collected from SARS‐CoV‐2‐infected MNs was able to re‐infect Vero E6 cells. Image analyses of SARS‐CoV‐2 nucleocapsid proteins by IF confirmed the results obtained. Furthermore, SARS‐CoV‐2 infection was accompanied by the activation of the antiviral and inflammatory response (HLA‐A, MX1 and BCL2) in MNs.

**Conclusions**: These results suggest for the very first time that SARS‐CoV‐2 can infect human MNs probably by binding CD147 and NRP1 receptors. New evidence indicate that these proteins have higher and broader patterns of expression in the human brain than ACE2 or TMPRSS2.

Such information will be essential to unveil the neuromuscular disorders characterizing SARS‐CoV‐2 infection and the so‐called long‐COVID symptoms.

### OAA0202 Depletion of plasmacytoid dendritic cells in ART‐suppressed SIV‐infected rhesus macaques to reverse dysfunction and exhaustion of cytotoxic CD8^+^ T lymphocytes and perturb the SIV reservoir

M.Y.‐H. Lee ^1^, G. Li^2,3^, J. Ma^3^, R. Mopuri^1^, K.A. Karunakaran^1^, T. Ton^1^, S.A. Lapp^1^, A.M. Metz^1^, K. Engelman^4^, S. Liang^5^, M.C. Lin^1^, K.A. Easley^6^, V. Govindu^7^, A.D. Silva Trenkle^8^, K. Gill^9^, E.A. Mahar^1^, N. Schoof^10^, K. Pellegrini^11^, M. Mavigner^10,12^, G.A. Kwong^8,13,14,15,16^, D. Magnani^4^, J.D. Altman^1,7,17^, A. Chahroudi^10,12^, L. Su^2,3^, R.R. Amara^1,17^, S.E. Bosinger^1,11,18^

^1^Division of Microbiology and Immunology, Emory National Primate Research Center, Emory University, Atlanta, United States, ^2^Division of Virology, Pathogenesis, and Cancer, Institute of Human Virology, Departments of Pharmacology and Microbiology and Immunology, University of Maryland School of Medicine, Baltimore, United States, ^3^Lineberger Comprehensive Cancer Center, Department of Microbiology and Immunology, School of Medicine, University of North Carolina at Chapel Hill, Chapel Hill, United States, ^4^MassBiologics, University of Massachusetts Medical School, Boston, United States, ^5^Emory National Primate Research Center and Emory Vaccine Center, Emory University, Atlanta, United States, ^6^Emory University, Department of Biostatistics and Bioinformatics, Rollins School of Public Health, Atlanta, United States, ^7^NIH Tetramer Core Facility, Emory University, Atlanta, United States, ^8^Wallace H. Coulter Department of Biomedical Engineering, Georgia Institute of Technology and Emory University, Atlanta, United States, ^9^Flow Cytometry Core, Emory Vaccine Center, Emory University, Atlanta, United States, ^10^Emory University School of Medicine, Department of Pediatrics, Atlanta, United States, ^11^Emory NPRC Genomics Core Laboratory, Emory National Primate Research Center, Emory University, Atlanta, United States, ^12^Center for Childhood Infections and Vaccines of Children's Healthcare of Atlanta and Emory University, Atlanta, United States, ^13^Winship Cancer Institute of Emory University, Atlanta, United States, ^14^Institute for Electronics and Nanotechnology, Georgia Institute of Technology, Atlanta, United States, ^15^Parker H. Petit Institute of Bioengineering and Bioscience, Georgia Institute of Technology, Atlanta, United States, ^16^Integrated Cancer Research Center, Georgia Institute of Technology, Atlanta, United States, ^17^Emory University School of Medicine, Department of Microbiology and Immunology, Atlanta, United States, ^18^Emory University School of Medicine, Department of Pathology and Laboratory Medicine, Atlanta, United States

**Background**: For people living with HIV (PLWH), unabated activation of plasmacytoid dendritic cells (pDCs) and Type I IFN signalling is associated with immune suppression and HIV‐1 persistence. Depletion of pDCs or blocking of the IFN I receptor during ART‐treated chronic HIV‐1 infection in humanized mice reverses HIV‐1 pathogenesis and rescues HIV‐specific CD8^+^ T cells.

**Methods**: We performed pDC depletion in SIVmac251‐infected rhesus macaques under long‐term ART using 1D3, a novel anti‐BDCA2/CD303 monoclonal antibody, and assessed the impact on CD8^+^ T‐cell exhaustion and SIV reservoirs. To study the SIV‐specific CD8^+^ T‐cell response, we generated barcoded Gag‐CM9 tetramers and performed CITE‐Seq and TCR immune profiling.

**Results**: We achieved depletion of pDCs in the peripheral blood (PB), lymph nodes (LNs) and bone marrow. In the LN, we observed a significant reduction in ISGs in CD4^+^ and CD8^+^ T cells at 16 days post‐infusion and a reduced expression of PD‐1 on central memory (CD95^+^ CCR7^+^) CD8^+^ T cells at 30 days post‐infusion. Two out of six macaques that received 1D3 controlled viremia after ART interruption compared to 0/6 control animals. There was no change in SIV‐DNA and SIV‐RNA in PB and LN CD4^+^ T cells after pDC depletion. Gag‐CM9 Tetramer^+^ CD8^+^ T cells of three Mamu‐A*01+ control animals were tracked at 26 days before and at 5 weeks after treatment interruption. We identified two major populations of LN SIV‐specific CD8^+^ T cells: exhausted (TOX^+^ PD‐1^+^ TIGIT^+^) and stem‐like cells (TCF1^+^ PD‐1^int^). By matching TCR clones at both time points, we demonstrate that the expansion of these populations to recrudescent SIV differs significantly.

**Conclusions**: Taken collectively, we report that in vivo pDC depletion was able to effectively suppress expression of ISGs and checkpoint blockade proteins in LN CD8^+^ T cells. Importantly, a subset of RMs in the depletion group maintained virological control off ART. These data suggest that therapeutics targeting the pDC/IFN axis are capable of functionally improving anti‐SIV CD8^+^ T‐cell responses to control SIV infection. Ultimately, this is a promising strategy to reverse the effects of chronic inflammation in ART‐suppressed PLWH that could be used to restore host immunity and contribute to an HIV cure.

### OAA0203 In vivo genome engineering of human T cells results in ART‐free control of HIV‐1 in humanized mice

P. Kumar ^1^, J. Beloor^1^, J. Rajashekhar^1^, I. Ullah^1^, P. Uchil^2^, L. Zhu^1^, Y. Sun^1^, S. Zeller^1^

^1^Yale University School of Medicine, Internal Medicine/ Infectious Diseases, New Haven, United States, ^2^Yale University School of Medicine, Microbial Pathogenesis, New Haven, United States

**Background**: Autologous cell transplant approaches for HIV cure typically involve re‐infusion of a person's own gene‐modified hematopoietic stem and/or T cells. However, poor post‐infusion engraftment of the gene‐modified cells remains a major hurdle for achieving successful control of HIV‐1 viral loads and ART‐free remission. In vivo gene therapy, which does not involve ex‐vivo cell manipulations, may address this issue. That said, effective transgene delivery to human hematopoietic cells in vivo is a formidable challenge in the gene therapy field.

**Methods**: We exploited the dominant surface expression of CD7 on human T cells and monocytes to allow targeted and effective transduction of these cell types with virus‐like particles (VLPs) surface‐decorated with a humanized antibody to human CD7. Importantly, as a key step towards clinical application, we adapted this platform for the delivery of genome‐integrating ORFs encoding short‐hairpin RNA (long‐term expression) and/or “scarless” delivery of packaged CRISPR ribonucleoprotein complexes (RNPs, transient expression). The platform was tested for abrogating expression of HIV‐1 host dependency factors in human T cells in humanized mouse models of HIV‐1 infection after systemic (intravenous) administration.

**Results**: Simple intravenous injection of CD7 antibody‐guided VLPs into humanized mice resulted in selective transduction of primary human T cells (and monocytes) with negligible off‐targeting and hepatotoxicity. We obtained impressive in vivo gene marking frequencies of > 50% of human T cells. Gene‐marked CD4 T cells selectively expanded in HIV‐1_JRCSF_‐infected humanized mice after withdrawal of antiretroviral therapy (ART) resulting in control of plasma viral loads and stabilization of CD4 T‐cell levels. Importantly, treated mice were resistant to repeated challenge with HIV‐1. ART‐free control of viral loads was also achieved humanized mice transplanted with CD4 T cells from people living with HIV (PLWH) demonstrating potential applicability as a cure strategy for HIV‐1.

**Conclusions**: The approach we report here represents an important advance to the field of gene therapy‐based cure for HIV in that it obviates the need for cell transplant protocols with ex‐vivo transduced hematopoietic cells. We expect our work to be a significant advance towards achieving ART‐free remission and of compelling interest to the fields of clinical gene‐therapy for HIV‐1 and other diseases.

### OAA0204 The EZH2 inhibitor Tazemetostat increases MHC‐I antigen presentation in vitro and in vivo, enhancing antiviral activities of HIV‐specific CTLs

A. Gramatica ^1^, A. Danesh^1^, F. Kahn^1^, I. Miller^1^, J. Weiler^1^, D. Copertino^1^, A. Ward^1^, T. Mota^1^, L. Leyre^1^, U. Chukwukere^1^, B. Jones^1,1^

^1^Weill Cornell Medical College, New York, United States

**Background**: Infected cells vary in their intrinsic susceptibilities to cytotoxic T‐lymphocytes (CTLs) mediated killing. We reported that overexpression of the survival factor BCL‐2 in reservoir cells confers a degree of resistance to CTLs, and that inhibiting BCL‐2 potentiated “shock and kill” reservoir reduction ex vivo. Here, we evaluate Enhancer of Zeste Homolog 2 (EZH2) as an additional mechanism of resistance, implicated by its transcriptional overexpression in HIV‐infected CD4^+^ T cells that survive CTL co‐culture. EZH2 is a histone‐methyltransferase that negatively regulates MHC‐I expression and is inhibited by the FDA‐approved drug Tazemetostat.

**Methods**: CD4^+^ T cells isolated from HIV‐positive donors were superinfected with HIV‐1_JRCSF_, treated with Tazemetostat and cocultured with HIV‐specific CTLs (in vitro‐killing assay), or co‐cultured with autologous CD8^+^ T cells in the presence of Tazemetostat (viral‐inhibition assay). Enhancement in the individual's antiviral response, driven by Tazemetostat, was evaluated by comparing the reduction in Gag(p24)^+^CD4^−^ T cells in treated versus untreated conditions. NodSCID‐/‐IL2rgnull mice were engrafted with memory CD4^+^ T cells from an HIV‐positive donor. Mice were then infected with HIV_JRCSF_ and either co‐engrafted or not with autologous memory CD8^+^ T cells. Tazemetostat or vehicle control were administered orally, up to 500 mg/kg BID. Viral loads and phenotypical analysis of T cells were conducted weekly.

**Results**: In both the in vitro‐killing and viral‐inhibition assays, co‐cultures of infected cells with HIV‐specific CTL clones, or bulk CD8^+^ T cells, resulted in the elimination of 50%–70% of infected cells. However, treatment with Tazemetostat induced higher MHC‐I levels on infected cells and significantly greater killing (up to 80%–90%). No such enhancement was observed with cells infected with a Nef‐deficient virus, suggesting that Tazemetostat may act by offsetting Nef‐mediated MHC‐I downregulation. In mice, viral loads were significantly reduced in +CD8 versus no‐CD8 mice. Tazemetostat drove a 2.1‐fold increase in surface MHC‐I on infected cells (*n* = 8, *p* value = 0.0004), and a further decrease of 1‐log (mean value) of viral load, relative to vehicle control +CD8 mice.

**Conclusions**: Tazemetostat increases MHC‐I expression in infected cells, counterbalancing Nef‐mediated immunoevasion. This resulted in enhanced infected‐cell elimination in vitro and decreased viral loads in vivo. Our results provide impetus for pre‐clinical studies assessing the impact on reservoir formation and for consideration of future clinical studies.

### OAA0205 Anti‐PD‐1 chimeric antigen receptor T cells efficiently target SIV‐infected CD4^+^ T cells in germinal centres of rhesus macaques

K. Eichholz ^1^, Y. Fukazawa^2^, C. Peterson^1^, F. Haeseleer^1^, M. Medina^2^, S. Hoffmeister^2^, D. Duell^2^, B. Varco‐Merth^2^, S. Dross^3^, H. Park^2^, C. Labriola^2^, M. Axthelm^2^, R. Murane^4^, J. Smedley^2^, L. Jin^1^, B. Rust^1^, D. Fuller^3^, H.‐P. Kiem^1^, L. Picker^2^, A. Okoye^2^, L. Corey^1^

^1^Fred Hutchinson Cancer Center, Vaccine and Infectious Disease Division, Seattle, United States, ^2^OHSU, Vaccine and Gene Therapy Institute, Portland, United States, ^3^University of Washington, Department of Microbiology, Seattle, United States, ^4^University of Washington, Washington National Primate Research Center, Seattle, United States

**Background**: Programmed cell death protein 1 (PD‐1) is an immune checkpoint marker commonly expressed on memory T cells and enriched in latently infected CD4^+^ T cells containing replication‐competent human immune deficiency virus 1 (HIV) provirus in people with HIV on antiretroviral therapy (ART).

**Methods**: We engineered novel chimeric antigen receptor (CAR) T cells that can efficiently kill PD‐1 expressing cells in vitro and in vivo to assess the impact of PD‐1 depletion on viral reservoirs and rebound dynamics in simian immunodeficiency virus (SIV) mac239‐infected rhesus macaques (RMs). Adoptive transfer experiments of anti‐PD‐1 CAR T cells were done in two SIV naïve and four SIV‐infected RMs on ART.

**Results**: In three of six RMs, one SIV naïve and two SIV+ RMs, anti‐PD‐1 CAR T cells expanded efficiently and persisted for up to 100 days concomitant with the depletion of PD‐1^+^ memory T cells in blood and tissues, including CD4^+^ follicular helper T cells (T_FH_). This depletion of T_FH_ in lymph nodes was also associated with the depletion of detectable SIV RNA from the germinal centre (GC). However, following an ART interruption, there was a marked increase in SIV replication in extrafollicular portions of lymph nodes, a 2‐log higher plasma viremia relative to controls and accelerated disease progression, associated with the acute depletion of CD8^+^ memory T cells after CAR T infusion in SIV+ RMs on ART.

**Conclusions**: These data indicate that anti‐PD‐1 CAR T cells can target and deplete PD‐1^+^ T cells in vivo including GC T_FH_ cells and eradicate SIV from this immunological sanctuary. Approaches to limit CAR T cell‐mediated depletion to PD‐1^+^ CD4^+^ T‐cell reservoirs and reduced off target CD8^+^ memory T‐cell depletion should be pursued.

### OAA0302 Differential susceptibility of cells infected with defective and intact proviruses to HIV‐selective cell killing by small molecule therapies

G.N. Kadiyala^1^, S. Telwatte^2^, A. Wedrychowski^1^, J. Janssens^1^, S.J. Kim^1^, P. Kim^3^, S. Deeks^4^, J. Wong^1^, S. Yukl ^1^

^1^University of California, San Francisco (UCSF) and San Francisco VA Medical Center, Medicine, San Francisco, United States, ^2^The University of Melbourne, Infectious Diseases, Melbourne, Australia, ^3^San Francisco VA Medical Center, San Francisco, United States, ^4^University of California, San Francisco (UCSF), Medicine, San Francisco, United States

**Background**: Some drugs that augment cell‐intrinsic defences or modulate cell death/survival pathways have been reported to selectively kill HIV‐infected cells and/or reduce HIV DNA. We hypothesized that these drugs may differ in their ability to kill cells infected with intact and defective proviruses.

**Methods**: We tested drugs currently in clinical use or human trials, including interferon alpha2A, interferon gamma, acitretin (RIG‐I inducer), GS‐9620/vesatolimod (TLR7 agonist), nivolumab (PD‐1 blocker), auranofin (p53 modulator), obatoclax (Bcl‐2 inhibitor), FX‐1 (Bcl‐6 inhibitor), bortezomib (proteasome inhibitor), birinapant (IAP inhibitor) and INK128/sapanisertib (mTOR[c]1/2 inhibitor). Drug concentrations were chosen based on levels attainable in plasma (if known) or previously tested in vitro. PBMCs were isolated from eight ART‐suppressed PWH, aliquoted into single or duplicate wells (6 × 10^6^ cells/well), and cultured for 6 days with ARVs and either DMSO (negative control), anti‐CD3/CD28^+^IL‐2 or individual drugs. After 6 days, we measured cell counts/viabilities and extracted DNA/RNA. Total, intact and defective HIV DNA was measured by IPDA (4–10 replicates), normalized to copies/million cells (using DNA mass and housekeeping genes), expressed as percent of the DMSO control and compared to DMSO (Wilcoxon signed rank test).

**Results**: A trend towards lower cell viability was observed with auranofin (median = 86.7% of DMSO; *p* = 0.11), FX‐1 (median = 84.6%; *p* = 0.063) and obatoclax (median = 93.9%; *p* = 0.078), so the doses were subsequently reduced. Obatoclax reduced intact HIV DNA (median = 17.0% of DMSO [range 0%–82.9%]; *p* = 0.0078) but not defective or total HIV DNA. A trend towards lower intact HIV DNA was also observed with auranofin (median = 53.9% [0%–156.5%]; *p* = 0.11), bortezomib (median = 78.6% [0%–121.5%]; *p* = 0.11) and INK128 (median = 33.2% [0%–138.2%]; *p* = 0.12). IFNalpha2A resulted in the lowest median intact HIV DNA (18.2%), but the effects were not consistent (range: 0%–232%; *p* = NS). Vesatolimod reduced 3’‐defective HIV DNA (median = 63.7% [0%–87.3%]; *p* = 0.031) and tended to reduce 5’‐defective DNA (median = 75.0% [0.5%–101.4%]; *p* = 0.063) but not intact or total HIV DNA. Other drugs showed no statistically significant effects.

**Conclusions**: Several drugs induced selective ex vivo depletion of cells with intact proviruses (obatoclax, possibly auranofin, INK128 and bortezomib) or defective proviruses (vesatolimod). Their distinct modes of action on cell death/survival and innate immune response provide a strong rationale to compare the effects of drug combinations ex vivo and in animal or human trials.

### OAA0303 Follicular T helper cells (TFHs) are a minor source of the active HIV reservoir in secondary lymphoid tissues of people with HIV (PWH) on prolonged suppressive antiretroviral therapy (ART)

J.M. Folkvord ^1^, M. Ollerton^1^, B.I. Mitchell^2^, F. Yost^2^, C.M. Shikuma^2^, L.C. Ndhlovu^3^, A. Chaillon^4^, D. Smith^4^, M. Porrachia^4^, S. Gianella^4^, E. Connick^1^

^1^University of Arizona, Tucson, United States, ^2^University of Hawaii/Hawaii Center for HIV/AIDS, Honolulu, United States, ^3^Weill Cornell Medicine, New York, United States, ^4^University of California San Diego, San Diego, United States

**Background**: TFHs are considered major HIV RNA expressing (vRNA^+^) cells in secondary lymphoid tissues during chronic, untreated HIV/SIV. TFHs are variably defined based on location in germinal centres, and/or expression of the canonical transcription factor BCL6 and multiple cell surface markers including PD‐1. The phenotype of vRNA^+^ cells within secondary lymphoid tissues of PWH on prolonged suppressive ART is unknown. We investigated the distribution and expression of BCL‐6 and PD‐1 by vRNA^+^ cells in lymph nodes (LNs) and spleen of PWH receiving virally suppressive ART.

**Methods**: Formalin‐fixed paraffin‐embedded LN tissues from six males on suppressive ART a median of 20 years were sectioned, baked at 60˚C, deparaffinized and subjected to antigen retrieval and protease treatment. In situ hybridization (ISH) for vRNA (ACDbio), and immunofluorescent antibody staining for CD20 was performed and vRNA^+^ cell frequencies determined. Spleens from six male PWH on suppressive ART a median of >5 years were embedded in OCT, sectioned and fixed in paraformaldehyde. ISH for vRNA and BCL6, and immunofluorescent antibody staining for PD1 and CD20 were performed in three LNs and six spleens and vRNA^+^ cell phenotypes determined. CD20^+^/CD20^−^ areas defined follicular/extrafollicular regions, respectively. Data are reported as medians.

**Results**: Most vRNA^+^ cells in LN (55%; range 33%–69%) and spleen (91%; range 82%–100%) were located in extrafollicular regions. Frequencies of vRNA^+^ cells were higher in follicular versus extrafollicular regions in LN (0.28 vs. 0.17 cells/mm^2^) and spleen (0.07 vs. 0.05 cells/mm^2^), but only 31% and 5.4% of tissues consisted of follicle, respectively. Twenty‐five HIV RNA^+^ cells were evaluated in LN (9, range 7–9 cells; *n* = 3) and 182 in spleen (32, range 6–52 cells; *n* = 6). Germinal centres were not observed. BCL6 was observed in 71% and 27%, and PD1 in 14% and 12% of vRNA^+^ cells in LN and spleen, respectively. Of vRNA^+^ cells, 14% (range 0%–33%) in LN and 4% (range 0%–17%) in spleen were TFH (BCL6^+^/PD1^+^).

**Conclusions**: TFHs are a minor source of the active reservoir in secondary lymphoid tissues of PWH on prolonged suppressive ART. Strategies to eliminate the active reservoir must identify and target additional cell phenotypes.

### OAA0304 Hypoxic adaptation uncovers a glycolytic dependence of HIV‐1 latency reversal

Y.I. Kayode ^1^, D.C. Clemmer^1^, A.A. Owolabi^1^, L.M. Clark^1^, H.E. Taylor^1^

^1^SUNY Upstate Medical University, Microbiology and Immunology, Syracuse, United States

**Background**: The main barrier to HIV‐1 curative strategies is the persistence of latently infected CD4 T cells in lymphoid tissue compartments which readily fuel viral rebound following antiretroviral therapy (ART) interruption in people living with HIV (PLWH). However, our incomplete understanding of the role of the unique metabolic microenvironment in these tissues in controlling latency reversal remains a limitation to advancements in the field of HIV curative research. Based on prior associations of glycolytic metabolites with post‐translational modifications that facilitate HIV‐1 replication during primary infection, and with viral rebound following ART interruption, we directly tested the hypothesis that glycolysis facilitates epigenetic modifications that promote HIV‐1 latency reversal.

**Methods**: We utilized orthogonal pharmacological, metabolomic and genetic tools in functional cell‐based assays using multiple CD4 T‐cell models of HIV‐1 latency. We also utilized Epi‐FLOW, a single‐cell flow cytometry‐based assay developed in our lab to assess global epigenetic marks in reactivating cells. All experimentation was performed in our hypoxic workstation which is maintained at 1% oxygen (physiologic hypoxia) or in standard normoxic tissue culture conditions (21% O_2_).

**Results**: We show that CD4 T‐cell adaption to physiological hypoxia present in lymphoid tissues drives glucose sensitivity and glycolytic‐dependence of HIV‐1 latency reversal. By modelling physiologic variations in oxygen and glucose availability as found in HIV‐1‐harbouring tissues in vivo, we uncover a differential glycolytic dependence of compounds from two clinically relevant latency reversal agent (LRA) classes; the PKC agonists and histone deacetylase inhibitors (HDACis) during physiologic hypoxia. Our metabolomic profiling reveals that this differential dependence on glycolysis is attributable to differential capacities of LRAs to induce glucose uptake and glycolytic flux. We further define a role for glycolysis in facilitating both histone acetylation and lactylation, post‐translational modifications associated with latency reversal.

**Conclusions**: Taken together, our current findings uncover glucose and oxygen availability as critical metabolic determinants of HIV‐1 latency reversal and underscore the importance of executing studies of latency reversal under physiological conditions to identify compounds that effectively target the latent reservoir in vivo.

### OAA0305 Analysis of HIV transcribing cells from viraemic and virally suppressed individuals living with HIV using novel single‐cell RNAseq method

S. Telwatte ^1,2,3^, J. Frouard^4^, X. Luo^4^, D. Arneson^5^, GN. Kadiyala^3^, A. Wedrychowski^3^, R. Hoh^6^, A. Butte^5^, S. Deeks^6^, S. Lee^6^, N. Roan^4^, S. Yukl^2,3^

^1^The Peter Doherty Institute for Infection and Immunity, Infectious Diseases, Melbourne, Australia, ^2^University of California, San Francisco, Infectious Diseases, San Francisco, United States, ^3^San Francisco Veteran Affairs Medical Center, Infectious Diseases, San Francisco, United States, ^4^Gladstone Institute of Virology and Immunology, San Francisco, United States, ^5^University of California, San Francisco, Bakar Computational Health Sciences Institute, San Francisco, United States, ^6^San Francisco General Hospital, San Francisco, United States

**Background**: Latently infected CD4^+^ T cells are considered the main barrier to a cure for HIV‐1. During viral suppression under antiretroviral therapy (ART), a proportion of infected cells transcribe HIV, and these are predictive of time to viral rebound after ART cessation. Characterization of this HIV‐transcribing “reservoir” has been limited by technical challenges, including the very low frequency of HIV‐1‐transcribing cells, the low levels per cell of HIV RNA (most of which is not polyadenylated) in ART‐treated individuals, the lack of cellular biomarkers to identify HIV‐infected cells, and the fact that most HIV‐infected cells reside in lymphoid tissues.

**Methods**: To address these limitations, we developed “HIV‐Seq,” a new single‐cell (sc)RNAseq approach that incorporates custom‐designed HIV‐specific capture sequences and DNA‐barcoded antibodies to key cell surface proteins (CITE‐seq) into a single cell RNAseq (scRNAseq) workflow (10X Genomics). Using this approach, we characterized the transcriptome and surface proteome of unstimulated HIV‐infected cells from blood and gut tissue from people living with HIV (PWH).

HIV‐seq was applied to longitudinal samples obtained from four PWH before ART (Week 0) and after ART suppression (Week 24 or 45). CD4^+^ T cells were enriched using magnetic beads and stained with DNA‐tagged antibodies. HIV capture sequences were incorporated during library preparation. We also characterized leukocytes (CD45^+^) and T cells (CD3^+^) from the blood and gut of one ART‐suppressed individual. scRNAseq and CITE‐seq analyses were performed and sequences were aligned to a constructed subtype B consensus reference sequence.

**Results**: In the viraemic samples, HIV‐seq enabled the identification of 32%–72% more HIV RNA^+^ cells than scRNAseq without HIV‐specific primers. HIV transcripts aligned to pol and gag regions at the highest frequency, irrespective of capture; however, HIV‐seq typically yielded more HIV transcripts/infected cell. In total, we identified 1345 HIV RNA^+^ cells from viraemic time points and 26 HIV RNA^+^ cells from the ART‐suppressed time points representing the transcriptionally active reservoir.

**Conclusions**: Our HIV‐seq method enables efficient identification and characterization of HIV‐infected cells including in the context of ART suppression, allowing for in‐depth transcriptomic and surface phenotypic analysis of HIV‐transcribing reservoir cells.

### OAA0402 Novel V1 deleted‐envelope vaccine based on VLP protects against SHIV infection

I. Silva de Castro ^1^, A.M. Rahman^1^, M. Bissa^1^, J.D Stamos^1^, S. Sarkis^1^, M. Doster^1^, A. Gutowska^1^, K. N'guessan^2^, D. Paquin‐Proulx^2^, T. Cardozo^3^, G. Franchini^1^

^1^NIH, CCR, Bethesda, United States, ^2^MHRP, Walter reed Army Institute of Research, Silver Spring, United States, ^3^New York University School of Medicine, New York, United States

**Background**: Our group have demonstrated that vaccination based in viral like particles (VLPs) using DNA/ALVAC/gp120^+^Alum platforms delivering SIV envelope immunogens engineered by V1 deletion to favour a‐helix conformation of V2 was superior to wild‐type (WT) envelope immunogens in decreasing the risk of SIVmac_251_ acquisition in macaques. In the current study, we investigate whether a similarly engineered vaccine based on HIV also differed from WT‐based envelope immunogens in immunogenicity and efficacy.

**Methods**: We created a DV1 gp160 DNA vaccine by deleting V1 (DV1 gp160) in the envelope of HIV clade A/E (HIV A244) and expressed also the corresponding DV1 A244 gp120 protein in CHO cells. DV1 or WT gp160 combined with p55Gag DNA vaccine were given at weeks 0 and 4, followed by two immunizations with ALVAC‐HIV at weeks 8 and 12; one WT or DV1 HIV A244 gp120 protein boost in alum was given at week 12. Two months following the last immunization, animals were exposed to 11 weekly low doses of SHIV 1157(QNE) Y173H intrarectally.

**Results**: The DV1 immunogens decreased the risk of SHIV 1157 (QNE) acquisition by 81% compared to controls, whereas the WT immunogens did not, recapitulating the results obtained with SIV‐based vaccines. Analyses of immune response elicited by the HIV A244WT and A244DV1 immunogens revealed several differences. Immunization with DV1 elicited higher V2‐specific ADCC, and more efficient efferocytosis, responses correlating with the decreased risk of virus acquisition also in the SIV. Similarly, the frequency of mucosal CD14^+^ cells, IgG envelope‐specific B cells (that correlated directly with V2‐specific ADCC), CCR2^+^ and of CD73^+^ rectal macrophages and in non‐classical blood monocytes was higher in DV1 than in WT‐immunized animals. The two immunization regimens differed also in the level of trogocitosis that was higher in WT immunized animals and correlated negatively with V2‐specific ADCC.

**Conclusions**: Thus, for reasons that are unclear at present, we conclude that V1 affects protective responses against HIV and further studies will be needed to address the mechanism(s).

### OAA0403 Perturbation of mucosal granulocytic effector cells in lentivirus infections

C. Manickam ^1^, A. Afifi^2^, R. Jones^2^, S. Sugawara^2^, R.K. Reeves^2^

^1^Duke University, Surgery, Durham, United States, ^2^Duke University, Durham, United States

**Background**: Granulocytes, including eosinophils and neutrophils, are critical innate effector cells bearing high Fc receptor expression and armed with a preformed pool of inflammatory and cytotoxic mediators and are also highly enriched in the GI mucosae. However, their roles in lentiviral‐mediated intestinal pathology and immunoprotection have been largely overlooked. To address this deficit, we studied granulocyte phenotypes, distribution, function and signalling using non‐human primate models of HIV infection as well as human blood samples.

**Methods**: Mononuclear cells and tissue samples from jejunum, colon, cervix, vagina, lymph nodes, spleen, liver and whole blood of naïve, acute SIVmac251‐, and chronic SHIVsf162p3‐infected rhesus macaques (RM) were analysed by imaging cytometry and advanced polychromatic flow cytometry. Peripheral granulocytes of naïve RM and humans were used for controls and for functional assays, including respiratory burst assay by flow cytometry, ‘net'osis assay by confocal microscopy and multiplex signalling analyses. A neutrophil antibody‐mediated cell phagocytosis (ADNP) assay used HIV‐gag opsonized fluorescent microbeads and HIV‐specific antibodies.

**Results**: Flow cytometric and imaging data confirmed granulocyte phenotypes as CD45^+^CD66abce^+^CD14^+^CD49d^−^ neutrophils and CD45^+^CD66abce^+^CD14^−^CD49d^+^ eosinophils based on their surface marker expression, nuclear morphology and cytoplasmic granularity in whole blood and tissues. Significant modulation of granulocytic subsets was observed in mucosal sites of SIV/SHIV replication as evidenced by the depletion of jejunal eosinophils and vaginal neutrophils and eosinophils. Interestingly, neutrophils in circulation and colorectal biopsies were elevated indicating tissue‐specific modulation of granulocytes in chronic SHIV infection. Further, the efficacy of RM and human granulocytes as Fc effector cells was substantiated in vitro by their ability to generate reactive oxygen species, phosphorylation of important signalling adaptors, including Syk, ZAP70, Lck and LAT upon CD32 and CD16 crosslinking and extracellular trap generation. In addition to FcgR‐mediated responses, we observed elevated phagocytosis when neutrophils were cultured in the presence of VRC01‐IgA compared to the VRC01‐IgG subtype, indicating HIV‐specific mucosal activity.

**Conclusions**: Granulocytes are depleted in SIV/SHIV infection, notably in the gastrointestinal and reproductive mucosae where significant inflammation and disruption occurs in lentivirus‐induced disease. Mucosal depletion of granulocytes could potentially lead to pathogenic co‐morbidities and also adversely affect the outcome of antibody‐based therapies and HIV cure, thus warranting further studies.

### OAA0404 Diverse envelope trimers with altered glycan coverage around the CD4‐binding site elicit neutralizing antibodies of >50% breadth in NHPs

X. Chen ^1^, T. Zhou^1^, C. Cheng^1^, Z. Sheng^2^, E.‐S. Stancofski^1^, A.R. Corrigan^1^, E. Sarfo^1^, S. Charaf^1^, K. Mckee^1^, M. Louder^1^, N.A. Doria‐Rose^1^, B. Zhang^1^, J.R. Mascola^1^, R. Koup^1^, L. Shapiro^2^, P. Kwong^1^

^1^VRC/NIAID/NIH, Bethesda, United States, ^2^Columbia University, New York, United States

**Background**: Vaccine elicitation of broadly neutralizing antibodies (bnAbs) remains a challenge to the development of an effective HIV‐1 vaccine. BnAbs targeting the conserved CD4‐binding site (CD4BS) have been identified in many HIV patients and are often broad and potent. Immunization with stabilized HIV‐1 envelope trimers (Env) generally only elicits autologous or narrow‐breadth neutralization. We previously reported that Env trimers with four glycans removed around the CD4BS elicited strong neutralizing response to the glycan‐removed CD4BS but not to wild‐type glycan‐covered envelopes in NHPs.

**Methods**: We further boosted the above NHPs with diverse Env trimers, first with removed glycans partially restored (2–3x each) and then with natively glycosylated HIV‐1 Envs (6x). Sera and PBMCs were collected 2 weeks after each immunization and analysed for neutralization and B‐cell recognition. B‐cell sorting and RT‐PCR for antibody identification were performed at terminal time point. Binding, neutralization and cryo‐EM analyses were performed on identified IgGs. Longitudinal NGS was performed for two NHPs with bnAb lineages.

**Results**: Boosting with partially glycan‐restored and natively glycosylated HIV‐1 Envs resulted in broadly serum neutralizing responses in a subgroup of non‐human primates, four of which neutralized more than 10 out of 17 tested tier II isolates. From B‐cell sorting with BG505.SOSIP and its CD4BS‐knock‐out mutant as probes, we isolated multiple neutralizing antibodies from these four NHPs. The top two antibodies, A11V093‐10 and A13V144‐91, from two different NHPs, neutralized 56% and 54% of viruses in a 208‐isolate panel, respectively. Cryo‐EM structures showed that they both targeted the CD4BS with heavy‐light chain‐orientation flipped relative to that of VRC01. Both mimicked key CD4 and VRC01 interactions to HIV‐1 gp120, but A13V144‐91 used a flexible and shortened CDR‐H1 to accommodate glycan 276, while A11V093‐10 bound to gp120 with an epitope that shifted away from glycan 276 to reduce interaction to this glycan and achieve broad neutralization. Longitudinal NGS analyses of the two bnAb lineages indicate that the initial expansion of the lineages coincided with immunization by the first glycan‐restored trimer containing a restored glycan at N197.

**Conclusions**: Env trimer immunization with glycans modulated around the CD4BS can elicit broadly neutralizing CD4BS antibodies in NHP.

### OAA0405 Protective efficacy of intranasal vaccination with a Sendai virus vector expressing a spike antigen against SARS‐CoV‐2 infection in mice

H. Ishii ^1^, K. Miyauchi^1^, S. Harada^1^, M. Nakamura‐Hoshi^1^, S. Seki^1^, D. Kaneda^1,2^, M. Okazaki^1^, T. Matano^1,2,3^

^1^AIDS Research Center, National Institute of Infectious Diseases, Tokyo, Japan, ^2^Institute of Medical Science, University of Tokyo, Tokyo, Japan, ^3^Joint Research Center for Human Retrovirus, Kumamoto University, Kumamoto, Japan

**Background**: Currently approved COVID‐19 vaccines have shown strong protective efficacy against disease onset and progression after SARS‐CoV‐2 infection. However, systemic immune responses induced by intramuscular vaccination have limited impact on the protection of viral transmission. Intranasal vaccination is an important strategy for the induction of mucosal immune responses for protection of viral transmission. We have previously reported protective efficacy of intranasal vaccination with a Sendai virus (SeV) inducing CD8^+^ T‐cell responses against SARS‐CoV‐2 infection (Cell Rep Med 3:100520, 2022). In the present study, we investigated the potential of intranasal SeV vaccination inducing mucosal neutralizing antibody responses to prevent SARS‐CoV‐2 infection in a mice model.

**Methods**: BALB/c mice received two times of intranasal vaccination with a SeV vector expressing SARS‐CoV‐2 S1 domain fused with foldon, the trimerization domain of T4 fibritin (SeV‐S1‐fol), produced by ID Pharma, Co., Ltd. SeV‐S1‐fol‐vaccinated and unvaccinated mice were intranasally challenged with a mouse‐adapted SARS‐CoV‐2 strain QHmusX provided by Noriyo Nagata (Sci Adv 8:eabh3827, 2022). Viral sgRNA levels and infectious viral titres were examined in bronchoalveolar lavage fluid (BALF) obtained on day 3 post‐challenge.

**Results**: Intranasal SeV‐S1‐fol vaccination induced not only anti‐S IgG in plasma but also anti‐S IgA in BALF and nasal wash. Anti‐SARS‐CoV‐2 neutralizing activity was detected not only in plasma but also in BALF in the vaccinated mice. After SARS‐CoV‐2 challenge, all the nine unvaccinated mice showed significant body weight loss and high viral loads, sgRNA levels and infectious viral titres, in BALF. In contrast, all the wight vaccinated mice with no body weight loss had no detectable viral sgRNA nor infectious viruses in BALF except for one mouse with marginal sgRNA.

**Conclusions**: The present study indicates the potential of intranasal SeV‐S1‐fol vaccination to efficiently induce mucosal anti‐SARS‐CoV‐2 neutralizing antibody responses and confer sterile protection from SARS‐CoV‐2 infection.

### OAB0102 Persistent accelerated epigenetic ageing is associated with altered neuroimaging and immune biomarkers in a longitudinal cohort of vertically acquired HIV‐positive adolescents

S. Heany ^1^, J. Hoare^1^, N. Phillips^1^, D. Stein^1^, A. Levine^2^, H. Zar^3^, L. Myer^4^, S. Horvath^2^

^1^University of Cape Town, Psychiatry and Mental Health, Cape Town, South Africa, ^2^University of California, Los Angeles, Los Angeles, United States, ^3^University of Cape Town, Paediatrics, Cape Town, South Africa, ^4^University of Cape Town, Epidemiology, Cape Town, South Africa

**Background**: We have previously shown accelerated ageing in adolescents living with perinatally acquired HIV (PHIV+), based on discrepancies between epigenetic and chronological age. The current study examines follow‐up longitudinal patterns of epigenetic ageing, and the association of epigenetic ageing with cognition as well as whole brain structure changes in PHIV+ and healthy controls enrolled in the Cape Town Adolescent Antiretroviral Cohort Study (CTAAC).

**Methods**: The Illumina EPIC array was used to generate blood DNA methylation data from 60 PHIV+ adolescents and 36 age‐matched controls aged 9–12 years old at baseline, and again at a 36‐month follow‐up. Epigenetic clock software estimated two measures of epigenetic age acceleration: extrinsic epigenetic accelerated ageing (EEAA) and age acceleration difference (AAD) at both time points. At follow‐up, each participant completed neuropsychological testing, structural magnetic resonance imaging and diffusion tensor imaging.

**Results**: At follow‐up, PHIV status remained associated with increased EEAA and AAD. Accelerated epigenetic ageing remained positively associated with viral load and negatively associated with CD4 ratio. EEAA was positively associated with whole brain grey matter volume and alterations in whole brain white matter integrity. These results did not differ between genders. AAD and EEAA were not associated with cognitive function within the PHIV+ group.

**Conclusions**: Measures of epigenetic ageing, as detected in DNA methylation patterns, remain increased in PHIV+ adolescents across a 36‐month period. Associations between epigenetic ageing measures, viral biomarkers and alterations in brain micro and macrostructure also persist at 36‐month follow‐up. Increased variance in DNAmAging might indicate varying degrees of immune health response to treatment and development trajectories. Accelerated epigenetic ageing is also associated with virologic and treatment‐related variables, such as CD4 ratio and VL, indicating the importance of disease management during the adolescent stage. Neuroimaging results showed that epigenetic ageing is associated with reduced white matter fractional anisotropy and increased mean diffusion, indicating white matter microstructural damage. PHIV+ additionally have larger grey matter volumes, possibly due to ineffective pruning during the adolescent period.

### OAB0103 Is the recommended valganciclovir dosing for treatment of cytomegalovirus in infants adequate for treatment of cytomegalovirus pneumonia in HIV‐positive infants in sub‐Saharan Africa? A pharmacokinetic sub‐study in the EMPIRICAL trial

V. Mumbiro ^1^, T. Jacobs^2^, C. Moraleda^3^, L. Beca^4^, A. Passanduca^5^, L. Kakooza^6^, M. Chitsamatanga^1^, B. Nduna^7^, J. Bramugy^8^, A. Tagarro^3,9,10^, K.D. Chhaganlal^11^, S. Mutesi^12^, M. Bwakura^1^, N. Namuzizya^13^, Á. Ballesteros^3^, S. Dominguez‐Rodríguez^3^, A. Colbers^2^, J. Sacarlal^5^, D. Nalwanga^6^, H.A. Mujuru^1^, C. Chabala^14,13,15^, L. Madrid^3,16^, W.C. Buck^17,5^, V. Musiime^6^, P. Rojo^3,18,19^, D. Burger^2^, EMPIRICAL Clinical Trial Group

^1^University of Zimbabwe Clinical Research Center, Harare, Zimbabwe, ^2^Radboud University Medical Center, Department of Pharmacy, Nijmegen, Netherlands, ^3^Pediatric Unit for Research and Clinical Trials (UPIC), Hospital 12 de Octubre Health Research Institute (i+12), Biomedical Foundation of Hospital Universitario 12 de Octubre (FIB‐H12O, Madrid, Spain, ^4^Universidade Lúrio Faculty of Health Sciences, Nampula, Mozambique, ^5^Universidade Eduardo Mondlane Faculty of Medicine, Maputo, Mozambique, ^6^Makerere University, Kampala, Uganda, ^7^Arthur Davidson Children's Hospital, Ndola, Zambia, ^8^Centro de Investigação em Saúde de Manhiça, Manhiça, Mozambique, ^9^Pediatric Service. Infanta Sofia University Hospital, Servicio Madrileño de Salud (SERMAS), Madrid, Spain, ^10^Universidad Europea de Madrid, Madrid, Spain, ^11^Universidade Católica de Moçambique Faculty of Health Sciences, Beira, Mozambique, ^12^Jinja Regional Referral Hospital, Jinja, Uganda, ^13^University Teaching Hospital, Children's Hospital, Lusaka, Zambia, ^14^University of Zambia, School of Medicine, Lusaka, Zambia, ^15^Herpez Limited, Lusaka, Zambia, ^16^London School of Hygiene and Tropical Medicine (LMC), London, United Kingdom, ^17^University of California Los Angeles David Geffen School of Medicine, Los Angeles, United States, ^18^Pediatric Service, Hospital Universitario 12 de Octubre, Servicio Madrileño de Salud (SERMAS), Madrid, Spain, ^19^Complutense University of Madrid, Madrid, Spain

**Background**: Cytomegalovirus (CMV) is a cause of severe pneumonia in children with advanced HIV. Valganciclovir, the oral prodrug of ganciclovir, is used to treat CMV in immunocompromised hosts. No pharmacokinetic data are available to support valganciclovir dosing in infants living with HIV. This study aimed to determine the adequacy of dosing and sources of pharmacokinetic variability of ganciclovir in infants living with HIV with severe pneumonia.

**Methods**: The study was conducted within EMPIRICAL trial (#NCT03915366) for the treatment of severe pneumonia in infants living with HIV. Participants randomized to receive valganciclovir for empirical CMV treatment were recruited from hospitals in Zimbabwe, Zambia, Mozambique and Uganda between August 2020 and August 2022. Valganciclovir reconstituted syrup was given at 16 mg/kg/dose every 12 hours and pharmacokinetic sampling was done 2 and 5 hours post‐administration on day 3 of enrolment after at least three doses. Ganciclovir area‐under‐curve for a 12‐hour dosing interval (AUC_0‐12h_) was estimated using a limited sampling equation (AUC_0‐12h_ = 2.7*AUC_2‐5h_+6). The geometric mean AUC_0‐12h_ and number of subjects within the pharmacokinetic target for CMV treatment (AUC_0‐12h_ = 80–120 h*mg/L) were determined. Spearman's rank test was applied to test the extent to which baseline parameters (age, weight, weight‐for‐age, eGFR and BSA) correlated with ganciclovir AUC_0‐12h_.

**Results**: Geometric mean AUC_0‐12h_ (%CV) was 77.7 (54.4) h*mg/L. Of the 85 recruited participants, only 30 (35%) had AUC_0‐12h_ within the pre‐defined efficacy target for CMV treatment. The remaining subjects were either below (40 [47%]) or above (15 [18%]) the target. There was a positive correlation between AUC_0‐12h_ and weight‐for‐height z‐score (*r*(83) = 0.22, *p* = 0.042). A negative correlation was observed between AUC and age (*r*(83) = −0.41, *p*<0.001), eGFR (*r*(83) = −.036, *p* = 0.001) and BSA (*r*(83) = −0.25, *p* = 0.021).

**Conclusions**: A significant number of participants did not achieve the PK target for CMV treatment when receiving valganciclovir at 16 mg/kg/dose twice daily. This could result in decreased treatment response or failure. Exposure to valganciclovir decreased with increasing age, BSA, eGFR and poorer nutritional status. Future studies should investigate the clinical significance of these findings and if higher dosing or an alternative dosing strategy is required. This project is part of the EDCTP2 programme supported by the European Union (grant number RIA RIA2017MC‐2013).

### OAB0104 Pharmacokinetics, safety and efficacy of bictegravir/emtricitabine/tenofovir alafenamide (B/F/TAF) in virologically suppressed pregnant women with HIV

H. Zhang^1^, H. Martin^1^, L. Lin^1^, M. Davis^1^, H. Huang^1^, D. Xiao^1^, P. Arora^1^, A. Avihingsanon^2^, E. Koenig^3^, R. Palaparthy^1^, S. Girish^1^, D. Marathe ^1^

^1^Gilead Sciences, Inc., Foster City, United States, ^2^HIV‐NAT, Thai Red Cross AIDS Research Centre and CE of Tuberculosis, Faculty of Medicine, Chulalongkorn University, Bangkok, Thailand, ^3^Dominican Institute of Virological Studies (IDEV), Santiago, Dominican Republic

**Background**: Safe, effective and convenient treatment options are needed for pregnant women with HIV. Bictegravir (BIC) is highly protein bound and metabolized by uridine diphosphate glucuronosyltransferase 1A1 (UGT1A1) and cytochrome P450‐3A4 (CYP3A4). Physiological changes during pregnancy, including increased CYP3A4 and UGT1A1 activities, have been reported; however, limited data exist on B/F/TAF pharmacokinetics, safety and efficacy during pregnancy.

**Methods**: A dedicated open‐label study (NCT03960645) was conducted in 33 virologically suppressed pregnant women with HIV‐1. Steady‐state plasma samples were collected over 24 hours following oral administration of B/F/TAF during second and/or third trimesters of pregnancy, and 6 and 12 weeks postpartum. For BIC and TAF, protein binding was measured, and serial sparse samples collected in neonates. Geometric least‐squares mean (%GLSM) ratios were calculated for pharmacokinetic comparisons between pregnancy and postpartum samples. Plasma HIV‐1 RNA and trough peripheral blood mononuclear cell (PBMC) tenofovir diphosphate (TFV‐DP) levels were measured. The proportion of participants with HIV‐1 RNA <50 copies/ml (missing = excluded) at delivery was calculated.

**Results**: GLSM values for plasma B/F/TAF were lower during pregnancy versus postpartum (%GLSM ratios <100). For BIC and TAF, %GLSM ratios were higher when adjusted for protein binding, although they remained lower during pregnancy (Table). Trough PBMC TFV‐DP levels were generally similar during pregnancy and postpartum. All pregnant women maintained virologic suppression, with HIV‐1 RNA <50 copies/ml at delivery (*n* = 32 [100%]). In neonates, median (IQR) BIC half‐life was 43 (38, 58) hours, and TAF was below the quantitation limit in all neonates. There were no adverse events (AEs) leading to premature discontinuation and no drug‐related AEs in pregnant women or neonates.

**Figure:**
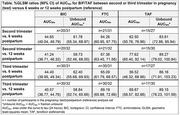
**Abstract OAB0104‐Table 1**.

**Conclusions**: Despite the comparatively lower exposure to BIC, emtricitabine and TAF during pregnancy versus postpartum, all adult participants maintained virologic suppression, and B/F/TAF was generally well tolerated, suggesting appropriateness for the use of B/F/TAF during pregnancy and indicating that no dose change is needed.

### OAB0105 Virological dynamics among children living with HIV transitioned to a dolutegravir‐based regimen in Nigeria

I. Ani ^1^, M. Unimuke^2^, F. Agu^3^, E. Umana^3^, A. Achanya^3^, E. Udofia^2^, C. Ezissi^1^, A. Idemudia^4^, E. Nwanja^1^, U. Akpan^1^, K. Agu^3^, O. Toyo^1^, A. Adeoye^1^, O. Onyedinachi^1^, D. Ogundehin^5^, E. James^5^, A. Eyo^1^, O. Omeh^3^, D. Oqua^3^

^1^Excellence Community Education Welfare Scheme, Uyo, Nigeria, ^2^Achieving Health Nigeria Initiative, Uyo, Nigeria, ^3^Howard University, Abuja, Nigeria, ^4^FHI 360, Abuja, Nigeria, ^5^USAID, Abuja, Nigeria

**Background**: Nigeria adopted the use of dolutegravir (DTG)‐based regimen as the preferred first‐line ART regimen for children living with HIV (CLHIV) in July 2021 and immediately commenced transitioning those on protease inhibitor (PI)‐based regimen to DTG. This paper aims to assess virological dynamic upon transitioning from PI‐ to DTG‐based regimen among CLHIV in Nigeria.

**Methods**: We conducted an institutional‐based retrospective cohort study using data from the electronic medical records from 155 health facilities in Akwa Ibom and Cross River States support by PEPFAR through United State Agency for International Development (USAID). The cohort included CLHIV (< = 9 years) who were transitioned from PI‐based to DTG‐based regimen between July 2021 and December 2021. The baseline viral load at transitioning and 12 months after transition was abstracted and categorized as undetectable viral load (< = 40 copies/ml), low‐level viremia (41–999 copies/ml) or unsuppressed viral load (> = 1000 copies/ml). Chi‐square statistics was used to compare the proportional difference in viral load change using STATA version 14 with statistical significance set at *p*<0.05.

**Results**: A total of 2358 CLHIV were transitioned to DTG‐based regimen as of December 2021. Median age was 6 years [IQR: 4–7 years], and 51.0% (*n* = 1203) were females. At baseline, 81.6% (*n* = 1924) were undetectable, 14.6% (*n* = 345) had low‐level viremia, while 3.8% (*n* = 89) were unsuppressed (> = 1000 copies/ml). Of the 2148 (91.1%) CLHIV who remained on ART 12 months after transitioning, 90.6% (*n* = 1947) were undetectable, 7.0% (*n* = 150) had low‐level viremia, while 2.4% (*n* = 51) were unsuppressed (> = 1000 copies/ml). There was no sex difference in virological dynamics (male = 91.7% vs. female = 92.5%; *p* = 0.374).

**Conclusions**: Children living with HIV achieved favourable virological changes when transitioned to DTG‐based regimen. Programmes should prioritize DTG‐based regimen in children in order to improve their treatment outcomes.

### OAB0202 Prior M184V/I and multiple prior virological failures have no impact on the efficacy of switching HIV‐positive adults to DTG/3TC through 96 wks in SOLAR‐3D

G. Blick ^1^, E. Cerreta^1^, G. Mancini^1^, A. Cosenza^1^, L. Fang^2^

^1^Health Care Advocates International, Stratford, United States, ^2^Pharstat Inc., Raleigh, United States

**Background**: DTG/3TC is approved for virologically suppressed HIV‐positive adults switching from stable ART with no prior DTG or 3TC resistance or prior virologic/treatment failure. SOLAR‐3D prospectively evaluated the ability of DTG/3TC to maintain virologic suppression in adults switched in the setting of prior and current M184V/I and multiple virologic failures.

**Methods**: SOLAR‐3D is a prospective, open‐label, comparative 96‐week study of HIV‐1‐positive adults virologically suppressed for ≥6 months, with ≥3 prior ART and prior virologic failures, enrolled from May 2019 to April 2020 and stratified by the history of prior M184V/I. There were no exclusions for prior INSTI use, any CD4, prior M184V/I or 3TC‐associated mutations detected at BL by proviral DNA NGS.

Primary and Secondary Efficacy Endpoints: Participants with PCR≥50 at Wk48 and 96, resp. by FDA snapshot (ITT‐E and PP). Additional Secondary Endpoints: PCR< 50 at Wk48 and 96 (ITT‐E, PP), incidence of AEs and discontinuation due to CVF (PCR≥50 followed by PCR >200).

**Results**: Hundred adults switched to DTG/3TC, *n* = 50 with historical/prior M184V/I (*n* = 15 with current M184V/I by proviral DNA NGS) and *n* = 50 without prior M184V/I. Those with prior M184V/I had more prior virologic failures (*n*[IQR]: 9[7–13]v4[3–5], *p*<0.001) and longer median duration HIV (28.4v15.5yrs, *p*<0.001), were older and had lower nadir CD4, longer ART duration and longer duration PCR<50 c/ml. Median time on DTG/3TC was 137 wks for both groups.

At wks 48 and 96, no difference in efficacy was observed in those with prior M184V/I compared to those without prior M184V/1:

Primary Endpoint: PCR≥50c/ml, *n*[%]: Wk48: 1[2]v3[6], ITT‐E (2.1v6.4, PP); Wk96: 2[4]v1[2], ITT‐E (4.6v2.2, PP);

Secondary Endpoint: PCR<50, *n*[%]: Wk48: 46[92]v44[88], ITT‐E (97.9v93.6, PP); W96: 42[84.0]v44[88.0], ITT‐E (95.5v97.8, PP);

Viral blips ≥50 at Wk96 (2v1) re‐suppressed at follow‐up. No CVFs were observed in either group;

At Wk96, no differences regarding PCR TND (<20), viral blips, AEs or discontinuations were observed;

Baseline proviral DNA NGS demonstrated that M184V/I was no longer present in 64.4% of those with prior M184V/I.

**Conclusions**: Through 96 wks, SOLAR‐3D, the largest prospective trial evaluating DTG/3TC switch in individuals with prior 3TC‐associated mutations, confirms that prior history/current presence of M184V/I does not impact the efficacy of switching virologically suppressed HIV‐positive adults with multiple prior virologic failures.

### OAB0203 Weight and body composition after switch to doravirine/islatravir (DOR/ISL) 100/0.75 mg once daily: week 48 results from two randomized active‐controlled phase 3 trials, MK8591A‐017 (P017) and MK8591A‐018 (P018)

G.A. McComsey ^1^, J.‐M. Molina^2^, A.M. Mills^3^, J.K. Rockstroh^4^, S. Walmsley^5^, R. Mngqibisa^6^, D. Braun^7^, Y.‐P. Zhou^8^, S.O. Klopfer^8^, A. Grandhi^8^, F.‐H. Su^8^, M.C. Fox^8^, A.J. Kim^8^, T.A. Correll^8^

^1^Case Western Reserve University, Cleveland, United States, ^2^University of Paris Cité, Paris, France, ^3^Men's Health Foundation, Los Angeles, United States, ^4^University Hospital Bonn, Department of Medicine I, Bonn, Germany, ^5^University Health Network, University of Toronto, Department of Medicine, Toronto, Canada, ^6^Enhancing Care Foundation, Wentworth Hospital, Durban, South Africa, ^7^University of Zurich, Institute of Medical Virology, Zurich, Switzerland, ^8^Merck & Co., Inc., Rahway, United States

**Background**: Some components of approved antiretroviral therapies (ARTs) are associated with weight gain and/or lipodystrophy (peripheral lipoatrophy and/or central fat hypertrophy). We evaluated changes in weight and body composition 48 weeks after switch to once‐daily DOR/ISL (100/0.75 mg) in two phase 3 studies.

**Figure:**
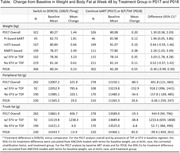
**Abstract OAB0203‐Table 1**.

**Methods**: P017 (NCT04223778) was an open‐label study in adults receiving any oral 2‐ or 3‐drug ART regimen. P018 (NCT04223791) was a double‐blind study in adults receiving bictegravir/emtricitabine/tenofovir alafenamide (B/F/TAF). Participants with HIV‐1 RNA <50 copies/ml were randomized (1:1) to switch to once‐daily DOR/ISL (100/0.75 mg) or to continue baseline ART (bART) (P017) or B/F/TAF (P018); P017 randomization was stratified by bART, which was PI‐based in 14% of participants, InSTI‐based in 52% and other (mainly NNRTI‐based) in 34%. Peripheral fat and trunk fat were measured by DEXA scan and were evaluated by a central imaging reader.

**Results**: Six hundred and fifty‐eight participants switched to DOR/ISL (100/0.75 mg), 336 remained on bART and 319 remained on B/F/TAF. Baseline weight differed by prior regimen in P017 (Table). Mean weight gain was similar for DOR/ISL versus continued B/F/TAF or other InSTI‐based regimens but was higher for DOR/ISL versus continued PI‐based or NNRTI‐based regimens (Table). Mean changes from baseline in peripheral and trunk fat were similar for DOR/ISL versus continued B/F/TAF in P018 but were higher for DOR/ISL versus continued bART in P017 (Table). In a post hoc analysis of P017, mean increases in weight, peripheral fat and trunk fat were higher for DOR/ISL versus continued bART containing efavirenz (EFV) and/or TDF but were similar for DOR/ISL versus continued non‐EFV/non‐TDF regimens (Table).

**Conclusions**: Changes in weight and body fat after switch to DOR/ISL were similar to continuing bART, except among participants who switched from EFV and/or TDF, which are known to suppress weight gain. Switching from InSTI‐based regimens to DOR/ISL did not reduce weight over 48 weeks.

### OAB0204 High rates of long‐term HIV RNA re‐suppression after virological failure on dolutegravir in the ADVANCE trial

B. Bosch^1^, S. Sokhela^1^, G. Akpomiemie^1^, N. Chandiwana^1^, W.D.F. Venter^1^, B. Simmons^2^, K. McCann^3^, M. Mirchandani^3^, A. Hill ^4^

^1^Ezintsha, University of the Witwatersrand, Faculty of Health Sciences, Johannesburg, South Africa, ^2^London School of Economics and Political Science, LSE Health, London, United Kingdom, ^3^Imperial College London, Faculty of Medicine, London, United Kingdom, ^4^University of Liverpool, Department of Pharmacology and Therapeutics, Liverpool, United Kingdom

**Background**: WHO Guidelines currently recommend switching to next‐line antiretroviral treatment (ART) for individuals with sustained HIV RNA viral load (VL) ≥1000 copies/ml despite adherence counselling. However, individuals can re‐suppress after adherence counselling, with no change in treatment. We compared the rates of virological failure and re‐suppression in the ADVANCE trial of first‐line treatment in South Africa.

**Methods**: In ADVANCE, 1053 treatment‐naive individuals were randomized to TAF/FTC/DTG, TDF/FTC/DTG or TDF/FTC/EFV for 192 weeks. All individuals with VL >1000 copies/ml received enhanced adherence counselling within 4 weeks. Time to first VL ≤50 copies/ml was compared between treatment arms using Kaplan–Meier methods. Rates of virologic failure (any VL≥1000 copies/ml after Week 24) were then compared. For individuals with virological rebound, rates of VL re‐suppression <50 copies/ml were compared with follow up to Week 192.

**Results**: Time to suppression ≤50 copies/ml was significantly shorter in the combined DTG arms (4 weeks) compared to the EFV arm (12 weeks); (log‐rank *p*<0.001). The proportion with virologic failure was similar across arms (combined DTG 87/702 [12%] vs. EFV 33/351 [9%]; log‐rank *p* = 0.343). However, more individuals on EFV remained viraemic prior to failure (12/33 [36%] compared with DTG 10/87 [11%]; *p* = 0.002). For individuals with rebound ≥1000 copies/ml after Week 24, time to re‐suppression was significantly shorter for DTG (12 weeks) than EFV (26 weeks); log‐rank *p*<0.001. There were no cases of treatment‐emergent DTG resistance in the individuals with virological failure ≥1000 copies/ml.

**Conclusions**: In ADVANCE, episodes of viraemia≥1000 copies/ml were seen at similar rates across treatment arms. However, HIV RNA re‐suppression after viraemia ≥1000 copies/ml was significantly more likely for individuals taking either TDF/FTC/DTG or TAF/FTC/DTG, compared with TDF/FTC/EFV. Long‐term follow‐up suggests that most individuals on continued DTG after viraemia elevation can re‐suppress with enhanced adherence counselling. These results question the need for switch to second‐line PIs after VF on DTG.

**Figure:**
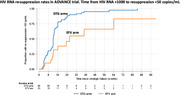
**Abstract OAB0204‐Figure 1**.

### OAB0205 Lenacapavir oral bridging maintains efficacy with a similar safety profile when SC LEN cannot be administered

O. Ogbuagu ^1^, A. Avihingsanon^2^, S. Segal‐Maurer^3^, H. Wang^4^, M. Rhee^4^, H. Dvory‐Sobol^4^, P. Sklar^4^, J.‐M. Molina^5^

^1^Yale University School of Medicine, New Haven, United States, ^2^Thai Red Cross AIDS Research Centre, HIV‐NAT, Bangkok, Thailand, ^3^New York Presbyterian Queens, New York, United States, ^4^Gilead Sciences, Inc., Foster City, United States, ^5^University of Paris Cité, Paris, France

**Background**: Lenacapavir (LEN) is a first‐in‐class, long‐acting, HIV‐1 capsid inhibitor, recently approved, in combination with other antiretrovirals, for the treatment of multidrug‐resistant (MDR) HIV‐1. Subcutaneous (SC) LEN provides a Q6M treatment option for HIV‐1; however, potential SC treatment interruptions may lead to management challenges due to SC treatment gaps. This sub‐analysis investigated the efficacy and safety of an oral bridging (OB) regimen (LEN 300 mg PO QW) in participants with MDR HIV‐1 as well as treatment‐naïve people with HIV‐1 (PWH) enrolled in the CAPELLA and CALIBRATE studies, respectively, when SC LEN dosing was interrupted due to its clinical hold.

**Methods**: Virologic suppression, CD4^+^ cell counts and safety outcomes were assessed from available data at baseline (time of initiation of OB) and until SC resumption or early discontinuation from OB due to FDA clinical hold (12/2021–05/2022).

**Results**: Of 72 participants who received SC LEN in CAPELLA, 57 received OB (79%) and were included in this analysis. Of 105 participants who received SC LEN in CALIBRATE, 82 received OB (78%). In both studies, demographic and baseline characteristics were similar between OB and overall analysis sets. Median OB exposure was 19 weeks, and OB adherence (by pill count) was ≥95% in most participants. Results were consistent across both sub‐analyses. High virologic suppression rates were maintained among those already suppressed (HIV‐1 RNA <50 copies/ml) at OB baseline in CAPELLA and in all participants in CALIBRATE (Table). CD4 (abs/%) remained stable or increased from OB baseline. One participant (CAPELLA) who missed two non‐consecutive oral LEN doses did not maintain virologic suppression during OB. Treatment‐emergent AEs were similar to SC LEN. Two participants in CAPELLA (3.5%) and one participant in CALIBRATE (1.2%) experienced treatment‐related diarrhoea.

**Table:** 

**Abstract OAB0205‐Table 1. Number and proportion of participants with HIV‐1 RNA < 50 copies/ml by visit, missing = excluded**		
*n*/*N* (%)	CAPELLA^a,b^	CALIBRATE^b^
**OB Baseline**	46/46 (100)	82/82 (100)
**OB Week 10**	44/45 (98)	77/77 (100)
**OB Week 20**	30/31 (97)	58/58 (100)
**OB Week 30**	10/10 (100)	5/5 (100)

^a^ Participants had virologic suppression at OB Baseline.

^b^ Denominators reflect participants who reached the specified duration of OB.

**Conclusions**: High rates of virologic suppression, and stable or increased CD4, support the efficacy of OB in PWH, including those with MDR HIV‐1 whose SC LEN treatment was interrupted. During OB, LEN was generally safe and well tolerated.

### OAB0302 HIV among mpox cases: clinical characteristics and outcomes in the WHO Global Surveillance 2022

A. Hoxha ^1^, S. Kerr^2^, H. Laurenson‐Schafer^1^, N. Sklenovská^1^, P. Ndumbi Ngamala^1^, B.B. Mirembe^1^, J. Fitzer^1^, V. Ndarukwa^1^, I. Hammermeister Nezu^1^, K. Kaasik‐Aaslav^1^, B.J. Greene‐Cramer^1^, L. Alexandrova Ezerska^1^, T.P. Metcalf^1^, E. Hamblion^1^, M. Doherty^1^, M. Prochazka^1^, A.N. Seale^1^, R. Lewis^1^, O. Le Polain de Waroux^1^, B.I. Pavlin^1^, WHO Global Monkeypox Epidemiology Team

^1^World Health Organization, Geneva, Switzerland, ^2^CPC Analytics, Berlin, Germany

**Background**: Since May 2022, the ongoing multi‐country mpox outbreak has primarily affected gay, bisexual and other men who have sex with men (GBMSM). In some countries, up to 50% of cases have been reported among people living with HIV (PLHIV), and although HIV is not a risk factor for mpox, it might lead to an increased risk for complications and severe disease.

**Methods**: We analysed data from January to December 2022 from the mpox global case‐based surveillance system, established by WHO in collaboration with regional and national partners. We included all cases with available information on HIV status, and described epidemiological, clinical characteristics and outcomes in PLHIV. We used binomial logistic regression to explore whether living with HIV and immunosuppression (reported as Yes/No) were risk factors for hospitalization, ICU admission or death.

**Results**: Information on HIV status was available for 44% (34,973/80,843) of reported cases. Of these, 48% (16,788/34,973) were PLHIV, most of whom were male (99%; 16,497/16,550). Among PLHIV with information, 92% reported being GBMSM (12,071/13,166), 85% aged 18–44 years old (14,197/16,782) and sexual contact was reported as the main route of acquisition in 63% of cases (5360/8529). Clinical symptoms among PLHIV with information included any rash (79%; 10,248/12,997), fever (64%), genital rash (53%), lymphadenopathy (35%) and headache (35%). Among PLHIV, immunosuppression was reported in 5023 cases, 735 were hospitalized, 20 admitted to intensive care and 23 died. When compared to cases who were HIV negative and not immunosuppressed, immunosuppressed PLHIV were found to be at higher risk for hospitalization (OR = 2.00, CI = 1.64–2.43, *p* = <0.001) as were those who were immunosuppressed and HIV negative (OR = 3.56, CI = 1.80–7.01, *p* = <0.001). Living with HIV alone was not a risk factor. Due to the small sample size, no risk factors for ICU admission and death were found.

**Conclusions**: Among cases with mpox, PLHIV were not at increased risk for hospitalization unless immunosuppressed. Given that uncontrolled HIV might have led to disproportionate mpox morbidity, health systems need to ensure that PLHIV are aware of their diagnosis, linked to care, on effective antiretroviral treatment and achieve viral suppression. For individuals with unknown HIV status, mpox testing can be an opportunity for HIV testing, prevention and care.

### OAB0303 High rate of hepatitis C and reinfection after direct‐acting antiviral treatment among men‐who‐have‐sex‐with‐men living with HIV in Bangkok, Thailand

P. Promsena ^1,2^, F. Ocampo^1^, C.P. Sacdalan^1,2^, M. Paudel^3,4^, S. Pinyakorn^3,4^, D.J. Colby^3,4^, N. Ratnaratorn^1^, K. Poltavee^1^, N. Chomchey^1^, S. Sriplienchan^1^, T. Wansom^5^, N. Phanuphak^6^, S. Vasan^3,4^, D. Hsu^3,4^, L. Trautmann^3,4^

^1^SEARCH Research Foundation, Bangkok, Thailand, ^2^Faculty of Medicine, Chulalongkorn University, Bangkok, Thailand, ^3^U.S. Military HIV Research Program, Walter Reed Army Institute of Research, Silver Spring, MD, United States, ^4^Henry M. Jackson Foundation for the Advancement of Military Medicine, Inc., Bethesda, MD, United States, ^5^Dreamlopments Foundation, Bangkok, Thailand, ^6^Institute of HIV Research and Innovation, Bangkok, Thailand

**Background**: Hepatitis C virus (HCV) infection has been increasing among men‐who‐have‐sex‐with‐men (MSM) living with HIV. Direct‐acting antivirals (DAAs) are associated with high HCV cure rates, but re‐infection can occur if exposed to HCV again. This analysis describes HCV incidence, treatment outcomes and reinfection rates in an early‐treated acute HIV infection (AHI) cohort in Thailand.

**Methods**: SEARCH010/RV254 enrols participants with AHI. HCV antibody was measured at enrolment and annually. Participants with HCV viremia initiated DAAs. Sustained virological response (SVR) was assessed ≥ 12 weeks after treatment completion. HCV RNA was monitored annually in participants with HCV clearance (spontaneous or SVR). HCV incidence rates and reinfection rates with 95% confidence intervals (CI) per 100 person‐years of follow‐up (PYFU) were calculated using the exact method.

**Results**: Between 2009 and 2022, 694 HCV seronegative participants were enrolled. Most (92.5%) were MSM with median age 26.0 years (IQR 23.0–32.0). During total 3678 PYFU, 98 (14.1%) acquired HCV infection; incidence rate was 2.7/100 PYFU (95% CI 2.2–3.3). Median time to HCV diagnosis after AHI was 3.0 years (IQR 0.5–5.5). Differences in baseline demographic characteristics between participants who developed HCV and who remained HCV negative were not statistically significant (Table 1). Among 98 incident cases, the most common HCV genotype was 1/1a (*N* = 70, 93.3%). Sofosbuvir/ledipasvir (56.8%) and sofosbuvir/velpatasvir (33.8%) were the most frequently initiated DAAs. Of 72 participants who completed treatment, 68 (94.4%) achieved SVR. Additionally, 10 participants spontaneously cleared HCV RNA. HCV reinfection occurred in 9/78 (11.5%) during 106.6 total PYFU after clearance; at a rate of 8.5/100 PYFU (95% CI 3.9–16.0). The median time to reinfection was 1.0 years (IQR 0.9–2.5). 3/10 (30.0%) reinfections occurred after spontaneous clearance and 6/68 (8.8%) after SVR.

**Figure:**
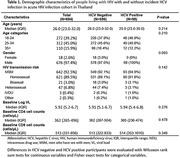
**Abstract OAB0303‐Table 1**.

**Conclusions**: In this early‐treated AHI cohort of Thai MSM, there is a high incidence of HCV infection. Treatment with DAAs resulted in high SVR rates, but HCV reinfection was common. Post‐clearance follow‐up with harm reduction measures is essential to identify and minimize HCV reinfection.

### OAB0304 Short‐course rifapentine‐based regimens for latent tuberculosis infection among people living with HIV who received integrase inhibitor‐based antiretroviral therapy

K.‐Y. Lin ^1^, H.‐Y. Sun^1^, C.‐J. Yang^2^, P.‐L. Lu^3^, N.‐Y. Lee^4^, Y.‐T. Lee^5^, H.‐J. Tang^6^, B.‐H. Liou^7^, N.‐C. Wang^8^, I.‐M. Hii^9^, T.‐C. Chen^3^, A.‐T. Peng^10^, C.‐Y. Lin^11^, C.‐Y. Cheng^12^, C.‐C. Hung^1^

^1^National Taiwan University Hospital, Taipei, Taiwan, ^2^Far Eastern Memorial Hospital, New Taipei City, Taiwan, ^3^Kaohsiung Medical University Hospital, Kaohsiung, Taiwan, ^4^National Cheng Kung University Hospital, Tainan, Taiwan, ^5^Chung Shan Medical University Hospital, Taichung, Taiwan, ^6^Chi Mei Medical Center, Tainan, Taiwan, ^7^Hsinchu MacKay Memorial Hospital, Hsinchu, Taiwan, ^8^Tri‐Service General Hospital, Taipei, Taiwan, ^9^Changhua Christian Hospital, Changhua, Taiwan, ^10^National Taiwan University Hospital Hsin‐Chu Branch, Hsin‐Chu, Taiwan, ^11^National Taiwan University Hospital Yu‐Lin Branch, Yunlin, Taiwan, ^12^Taoyuan General Hospital, Taoyuan, Taiwan

**Background**: Rifapentine‐based regimens have been shown to have a high completion rate for latent tuberculosis infection (LTBI) treatment; however, rifapentine reduces plasma concentrations of co‐administered ART among PLWH. This study aimed to retrospectively evaluate the outcomes of short‐course rifapentine‐based regimens among PLWH who received integrase strand‐transfer inhibitor (InSTI)‐based ART.

**Methods**: During August 2019–October 2022, PLWH testing positive or indeterminate for interferon‐gamma release assay (IGRA) were advised to receive directly observed therapy for LTBI after excluding active tuberculosis. Those receiving 3‐month once‐weekly rifapentine plus isoniazid (3HP) or 1‐month daily rifapentine plus isoniazid (1HP) combined with INSTI‐based ART were included. The primary outcome was maintenance of virologic response (PVL<200 copies/ml) at months 3–6 after the completion of LTBI treatment.

**Results**: During the study period, 456 PLWH were included; they were mostly male (94.3%) with a median age of 43 years, and 91.9% received InSTI‐based ART with a median CD4 count of 651 cells/mm^3^ and 97.6% having achieved PVL<200 copies/ml. Among those receiving InSTI‐based ART, 142 PLWH received 1HP and bictegravir (BIC)‐containing regimens (1HP/BIC group), 46 received 1HP and dolutegravir (DTG)‐containing regimens (1HP/DTG group), 28 received 3HP and BIC‐containing regimens and (3HP/BIC group), and 203 received 3HP and DTG‐containing regimens (3HP/DTG group). In the per‐protocol analysis, the proportions of PLWH who maintained PVL <200 copies/ml at months 3–6 after the completion of LTBI treatment in the four study groups were 100% (125/125), 100% (44/44), 100% (17/17), and 96.7% (178/184), respectively. The completion rates were similar in the 1HP and 3HP groups (92.7% vs. 90.0%). None of the PLWH discontinued INSTI‐based ART that had been taking before combinations with rifapentine‐based LTBI treatment. The rates of PLWH experiencing any adverse event (AE) were also similar in the 1HP and 3HP groups (60.9% vs. 57.4%), and the AEs were mainly of grade 1 (40.1%) or 2 (13.4%) in severity. While the most commonly reported AEs were flu‐like symptoms (40.1%), more dermatologic AEs were observed in the 1HP group compared with the 3HP group (17.8% vs. 9.6%).

**Conclusions**: Combinations of InSTI‐containing regimens and short‐course rifapentine‐based regimens had a good safety profile and maintained higher rates of viral suppression.

### OAB0305 Co‐infection of high‐risk human papillomavirus and human T‐lymphotropic virus‐1 among women living with HIV on antiretroviral therapy at a tertiary hospital in Kenya

J. Kangethe ^1,2,3^, E. Odari^4^, J. Pintye^5,6^, S. Gichuhi^7^, K. Mutai^8^, M. Mureithi^9,10^, A. Maiyo^11^

^1^Kenyatta National Hospital and University of Nairobi, Comprehensive Care Center for HIV, Medical Research, Microbiology and Immunology, Nairobi, Kenya, ^2^Consortium for Advanced Research Training in Africa, Nairobi, Kenya, ^3^African Research‐Data, African Population Studies, Accra, Ghana, ^4^Jomo Kenyatta University of Agriculture and Technology, Medical Microbiology, Nairobi, Kenya, ^5^University of Washington, Department of Biobehavioral Nursing and Health Informatics, Seattle, United States, ^6^University of Washington, Department of Global Health, International AIDS Research and Training Program, Seattle, United States, ^7^University of Nairobi, Ophthalmology, Nairobi, Kenya, ^8^Kenyatta National Hospital, Comprehensive Care Center for HIV, Nairobi, Kenya, ^9^University of Nairobi, Medical Microbiology and Immunology, Nairobi, Kenya, ^10^Kenya AIDS Vaccine Initiative, Institute of Clinical Research, Nairobi, Kenya, ^11^Kenya Medical Research Institute, Center for Virus Research, Nairobi, Kenya

**Background**: Virus‐associated cancers have emerged in recent years and account for 15% of cancers reported globally. Cancer‐causing viruses include high‐risk human papillomavirus (HR‐HPV), the causative agent of cervical cancer, and human T‐cell lymphotropic virus type‐1 (HTLV‐1), the causative agent of adult T‐cell leukaemia (ATL). In Kenya, there is a paucity of data on the burden of HR‐HPV/ HTLV‐1 co‐infection among women living with HIV (WLHIV). We determined the co‐infection of HR‐HPV and HTLV‐1 among WLHIV on antiretroviral therapy (ART) at the Kenyatta National Hospital (KNH).

**Methods**: We conducted a cross‐sectional study among WLHIV attending KNH (Kenya's national referral hospital) ART clinics. Cervical cytology was performed by KNH medical providers per standard of care. Study nurses collected a cervical sample with a cytobrush for HPV genotyping using Gene Xpert ® assays and HPV Genotypes 14 Real‐TM Quant″ V67‐100 FRT kits. Peripheral blood mononuclear cells (PBMCs) were used for HTLV‐1 DNA detection (Bioline Ltd., London, UK). The association of HR‐HPV and HTLV‐1 co‐infections was done using the Chi‐square function for trends.

**Results**: A total of 647 WLHIV enrolled in this study and had a mean age of 42.8 years (SD 8.7). All of the study participants were on ART; 7% were initiated on ART in ≤ 12 months and about 8.8% were not virally suppressed (>1000 copies/ml). The HTLV‐1 positivity rate among WLHIV was 3.1% and 7.6% among those with HR‐HPV. Participants with HR‐HPV/ HTLV‐1 co‐infections were significantly older (50+ years, 35.3%) than those having HPV mono‐infection or did not have any infection (*p* = <0.001). A significantly higher proportion of women with HR‐HPV/HTLV‐1 co‐infections had their sex debut before the age of 18 years (*p* = 0.015). A higher proportion of participants (52.2%) with HPV mono‐infection had previously been treated for STDs as compared to WLHIV without either HR‐HPV or HTLV‐1 infections (*p* = 0.006).

**Conclusions**: The study showed increasing trends of HR‐HPV/HTLV‐1 co‐infections among WLHIV on ART despite their CD4 cell count or HIV 1 RNA viral suppression at Kenya's national referral hospital.

### OAB0402 Integrase strand inhibitors (INSTI)‐related changes in BMI and risk of diabetes

D. Rupasinghe ^1^, K. Petoumenos^1^, B. Neesgaard^2^, E. Florence^3^, J. Begovac^4^, A. Rauch^5^, P. Tarr^5^, R. Zangerle^6^, F. Wit^7^, A. d'Arminio Monforte^8^, C. Mussini^9^, A. Sönnerborg^10^, M. Stecher^11^, V. Brandes^11^, S. De Wit^12^, E. Fontas^13^, N. Chkhartishvili^14^, A. Groh^15^, N. Jaschinski^2^, L. Greenberg^2^, L.B. Matharu^16^, A.A. Volny^17^, C. Cohen^18^, V. Vannappagari^19^, L. Young^20^, L. Ryom^2^, M. Law^1^

^1^The Australian HIV Observational Database (AHOD), Kirby Institute, Biostatistics and Databases Program, Unsw Sydney, Australia, ^2^CHIP, Department of Infectious Diseases, Rigshospitalet, University of Copenhagen, Copenhagen, Denmark, ^3^Institute of Tropical Medicine, Antwerp, Belgium, ^4^Reference Center for Diagnostics and Treatment of HIV‐infection, University Hospital for Infectious Diseases, Zagreb, Croatia, ^5^Swiss HIV Cohort Study (SHCS), University of Zurich, Zurich, Switzerland, ^6^Austrian HIV Cohort Study (AHIVCOS), Medizinische Universität Innsbruck, Innsbruck, Austria, ^7^AIDS Therapy Evaluation in the Netherlands (ATHENA) Cohort, HIV Monitoring Foundation, Amsterdam, Netherlands, ^8^Italian Cohort Naive Antiretrovirals (ICONA), ASST Santi Paolo e Carlo, Milano, Italy, ^9^Modena HIV Cohort, Università degli Studi di Modena, Modena, Italy, ^10^Swedish InfCare HIV Cohort, Karolinska University Hospital, Stockholm, Sweden, ^11^University Hospital Cologne, Cologne, Germany, ^12^CHU Saint‐Pierre, Centre de Recherche en Maladies Infectieuses a.s.b.l., Brussels, Belgium, ^13^Nice HIV Cohort, Université Côte d'Azur et Centre Hospitalier Universitaire, Nice, France, ^14^Georgian National AIDS Health Information System (AIDS HIS), Infectious Diseases, AIDS and Clinical Immunology Research Center, Tbilisi, Georgia, ^15^Frankfurt HIV Cohort Study, Johann Wolfgang Goethe‐University Hospital, Frankfurt, Germany, ^16^Centre for Clinical Research, Epidemiology, Modelling and Evaluation (CREME), Institute for Global Health, University College London, London, United Kingdom, ^17^European AIDS Treatment Group, Brussels, Belgium, ^18^Gilead, Foster City, United States, ^19^ViiV, Chapel Hill, United States, ^20^MSD, Rahway, United States

**Background**: Integrase strand inhibitor (INSTI) use in people living with HIV (PLWH) has been associated with an increased body mass index (BMI). BMI increases have also been associated with a higher risk of diabetes (DM). This study explored the relationship between INSTI and non‐INSTI regimens use, BMI changes and the risk of DM.

**Methods**: RESPOND participants were included if they had CD4, HIV RNA and multiple BMI measurements. Those with prior DM, and pregnant women, were excluded. DM was defined as a random blood glucose>11.1 mmol/L, HbA1c>6.5%/48 mmol/mol, use of antidiabetic medication or clinical diagnosis. Poisson regression assessed the association between time updated log BMI, current INSTI/non‐INSTI and TDF/TAF use, and their interactions, on DM risk.

**Results**: In total, 20,865 PLHIV were included, most were male (74%) and White ethnicity (73%). The median age was 45 years (IQR 37–52) with a median BMI of 24 kg/m^2^ (IQR 22–26). Over 107,641 PYFU, there were 785 DM diagnoses, a crude rate of 0.73 (CI 0.68–0.78)/100 person years. Log BMI was strongly associated with DM (aIRR 18.2 per log increase, 95% CI 11.7, 28.3; *p*<0.001). In univariate analyses, current INSTI use was associated with an increased risk of diabetes (IRR 1.58, 95% CI 1.37, 1.82; *p*<0.001). This was partially attenuated when adjusted for time updated log BMI and other variables (aIRR 1.48, 95% CI 1.28, 1.72; *p*<0.001) (Figure 1). In adjusted analyses, current TAF use had similar DM risk to current TDF (aIRR = 0.98, 95% CI 0.79–1.20, *p* = 0.818). There was little evidence of an interaction between log BMI, INSTI and non‐INSTI use, and DM (*p* = 0.130).

**Figure:**
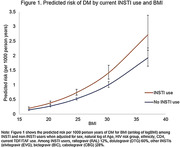
**Abstract OAB0402‐Figure 1**.

**Conclusions**: In RESPOND, the current use of INSTIs versus non‐INSTI was associated with an increased risk of diabetes which partially attenuated when adjusted for BMI changes and other variables. There was no difference in DM risk between current TAF and TDF users.

### OAB0403 Vitamin D and calcium intake are associated with bone deficits among adolescents living with HIV in Zambia and Zimbabwe

N. Dzavakwa ^1,2^, M. Chisenga^3^, K. Kranzer^4,1^, G. McHugh^1^, S. Filteau^5^, L. Kasonka^3^, H.B. Mabuda^3^, H. Mujuru^6^, N. Redzo^1^, S. Rowland‐Jones^7^, U.E. Schaible^8^, C.L. Gregson^9^, V. Simms^2,1^, R.A. Ferrand^4,1^, VITALITY team

^1^The Health Research Unit Zimbabwe (THRU ZIM), Harare, Zimbabwe, ^2^London School of Hygiene & Tropical Medicine, MRC International Statistics and Epidemiology Group, London, United Kingdom, ^3^University Teaching Hospital, Lusaka, Zambia, ^4^London School of Hygiene & Tropical Medicine, Clinical Research Department, London, United Kingdom, ^5^London School of Hygiene & Tropical Medicine, Department of Population Health, London, United Kingdom, ^6^University of Zimbabwe, Faculty of Medicine and Health Sciences, Harare, Zimbabwe, ^7^University of Oxford, Nuffield Department of Medicine, Oxford, United Kingdom, ^8^Research Center Borstel‐Leibniz Lung Center, Department of Cellular Microbiology, Borstel, Germany, ^9^University of Bristol, Bristol Medical School, Bristol, United Kingdom

**Background**: Adolescents living with HIV (ALWH) have lower bone density despite antiretroviral therapy (ART), and the aetiology may be multifactorial. We investigated whether dietary vitamin D and calcium intake were associated with bone density at baseline in peripubertal ALWH in Zambia and Zimbabwe, enrolled in a trial of vitamin D and calcium supplementation.

**Methods**: ALWH aged 11–19 years, established on ART for ≥6 months, were enrolled from five HIV clinics in Lusaka and Harare. A clinical history and examination was undertaken and HIV viral load was measured. Dual‐energy X‐ray absorptiometry was used to measure total body‐less head bone mineral density (TBLH‐BMD) Z‐score. The association between vitamin D/calcium daily dietary intake (calculated from a validated diet questionnaire) and TBLH‐BMD Z‐score was investigated using multivariable linear regression. A vitamin D dietary intake of <4 mcg/day was defined as low. Height‐ and weight‐ for‐age z‐scores of ←2 using UK reference standards were defined as stunting and wasting, respectively.

**Results**: Eight hundred and forty‐two ALWH (420 from Zambia and 422 from Zimbabwe, 448 [53.2%] female, mean age 15.0 years) were enrolled between February and November 2021. Unsuppressed HIV viral load (>1000 copies/ml) was observed in 11.7%; 29.9% were stunted and 30.3% were wasted. A low vitamin D intake was reported by 31.2%. Among 818 participants who had a DXA scan, lower dietary vitamin D was associated with lower TBLH‐BMD Z‐score; 0.07 (95% CI 0.01–0.13, *p* = 0.025) lower for each 1 mcg vitamin D, after adjusting for sex, Tanner stage, socio‐economic status and country. Similarly, lower dietary calcium intake was associated with lower TBLH‐BMD Z‐score; 0.10 (95% CI 0.02–0.17, *p* = 0.015) for each 100 mg calcium, adjusting for the same covariates. Mean TBLH‐BMD Z‐score was 0.26 lower in participants with unsuppressed HIV viral load.

**Conclusions**: ALWH in Zimbabwe and Zambia have low vitamin D and calcium dietary intake, and may benefit from supplementation to improve bone health.

### OAB0404 Effects of lifestyle modification and annual screening in the prevention of cardiovascular risk factors in South African women with HIV

S. Hanley ^1^, D. Moodley^2^, M. Naidoo^1^, S.S. Brummel^3^

^1^University of KwaZulu‐Natal, Family Medicine, Durban, South Africa, ^2^University of KwaZulu‐Natal, Obstetrics and Gynaecology, Durban, South Africa, ^3^Harvard T.H. Chan School of Public Health, Boston, United States

**Background**: Women with HIV (WHIV) are faced with an added burden of obesity and hypertension, particularly in under‐resourced settings. We sought to assess the effectiveness of regular screening and lifestyle modification interventions in modifying CVD risk factors in South‐African WHIV.

**Description**: Women with HIV aged 18 to <50 years were enrolled in a quasi‐experimental study (intervention [I‐arm] and control arm [C‐arm]) from two clinics in Umlazi, South Africa between November 2018 and May 2019. Women in the I‐arm received lifestyle modification advice and annual screening for CVD risk. The CVD risk factors were assessed through standardized questionnaires, clinical and laboratory procedures at baseline and at end of 3 years of follow‐up. Prevalence of metabolic syndrome (MetS) and other CVD indices were compared between arms at end‐of‐study (EOS) and incidence of CVD risk factors were measured within the intervention arm from baseline to EOS.

**Lessons learned**: A total of 372 WHIV (186 in each arm) were enrolled, and 269 WHIV (149 I‐arm and 120 C‐arm) with mean (SD) age of 36 (1) years were included in the EOS analyses after 32 (2) months of follow‐up. The MetS prevalence at EOS was 17% (25/149) in the I‐arm, and 20% (24/120) in the C‐arm (aRR 0.9; 95% CI 0.5–1.1). Proportion of women with low‐density lipoprotein (LDL) > 3 mmol/L in I‐arm and C‐arm were 19% (28/149) and 30% (36/120), respectively (aRR 0.6; 95% CI 0.4–0.9; *p*<0.05). Fasting blood glucose>5.6 mmol/L was reported in 3% (4/149) of women in I‐arm and 13% (16/20) in the C‐arm (aRR 0.2;95% CI 0.07–0.6; *p*<0.01). High‐density lipoprotein (HDL) improved within the I‐arm with a mean difference of −0.09 (95% CI: −0.16, −0.03]; *p*<0.01) from baseline to EOS. Mean body mass index increased in both arms: I‐arm from 29.6 (7) kg/m^2^ to 31.5 (7); C‐arm from 27.8 (7) to 29.9 (7) kg/m^2^.

**Conclusions/Next steps**: Regular screening and lifestyle modification advice play an important role in preventing CVD risk factors, such as LDL, HDL and glucose levels, and should be well integrated into HIV programmes. Further exploration is needed to understand the social determinants and perceptions of obesity in WHIV from under‐sourced settings, to inform future targeted interventions comprised of a culturally acceptable, multidisciplinary approach in the alleviation of obesity and CVD risk.

### OAB0405 Re‐evaluating risk: evaluating opioid‐related harm associated with stimulant use in people with chronic pain living with and without HIV

A. Appa ^1^, V. McMahan^2^, K. Long^2^, S. Shade^1^, P. Coffin^2^

^1^University of California San Francisco, San Francisco, United States, ^2^San Francisco Department of Public Health, San Francisco, United States

**Background**: Chronic pain, opioid therapy and substance use (particularly cocaine and methamphetamine) are all prevalent among people living with HIV (PLWH). However, there is lack of clarity regarding the impact of and optimal clinical response to stimulant use among people prescribed long‐term opioid therapy (LTOT) for chronic, non‐cancer pain. We sought to determine if a urine drug test (UDT) positive for stimulants was associated with opioid‐related harm or subsequent discontinuation of LTOT in a publicly insured population with a high proportion of PLWH.

**Methods**: Three hundred PLWH on LTOT were matched to 300 clients without HIV on LTOT (based on age, race/ethnicity and sex) and followed from January through June 2019. Using logistic generalized estimating equations, we assessed whether stimulant positive UDT results were associated with increased:

1. opioid‐related emergency department (ED) visits (oversedation, constipation, infections associated with injecting opioids and opioid seeking); and
2. LTOT discontinuation 90 days following a stimulant‐positive UDT.

**Results**: Overall, 1562 (24%) of 6471 UDTs were positive for stimulants; however, 30 clients had 39% of stimulant‐positive UDTs. There was no statistically significant association found between stimulant‐positive UDTs and opioid‐related ED visits or death within 90 days when accounting for repeated ED visits within individuals (adjusted hazard odds ratio [OR] = 1.24; 95% CI 0.73–2.11). This relationship did not differ by HIV status. Stimulant‐positive UDTs were associated with subsequent discontinuation of LTOT within 90 days (OR = 1.95; 95% CI = 1.68–2.27). This relationship was more likely among Latinx individuals (positive interaction; OR = 1.97; 95% CI = 1.08–3.60), and less likely among PLWH (negative interaction; OR = 0.59; 95% CI = 0.44–0.79).

**Conclusions**: The association between stimulant‐positive UDT and opioid‐related harm was concentrated in a minority of clients on LTOT with stimulant‐positive UDT. Despite this, stimulant‐positive UDT often led to LTOT discontinuation, though with heterogeneity across demographic and clinical groups. It is unknown whether less LTOT discontinuation among PLWH using stimulants reflects a more holistic approach to patient care among HIV providers, difficulty with opioid stewardship or both. Overall, these results suggest that detection of stimulant use should result in a discussion of substance use and risk, rather than reflex to discontinue LTOT.

### OAC0102 Australia's progress towards ending HIV as a public health threat: trends in epidemiological metrics over 2004–2021

R. Gray ^1^, H. McManus^1^, J. King^1^, K. Petoumenos^1^, A. Grulich^1^, R. Guy^1^, S. McGregor^1^

^1^Kirby Institute, UNSW Sydney, Sydney, Australia

**Background**: We describe Australia's overall progress towards achieving the UNAIDS 95‐95‐95 targets by analysing the trends in epidemiological metrics over 2004–2021.

**Methods**: We used mathematical modelling and national HIV notification, cohort and administration data to calculate annual estimates for the HIV cascade, the number of annual new HIV infections and other HIV epidemic metrics during 2004–2021. We used piecewise negative binomial regression to determine changes in trends and annual rate ratios (ARRs) for each cascade step and metric.

**Results**: We estimate there were 29,460 (range: 25,230‒34,070) people living with HIV in Australia at the end of 2021. All 90‐90‐90 targets were achieved in 2020, with 91.1% of people living with HIV diagnosed, 91.6% of those diagnosed on treatment and 97.8% of those on treatment being virally suppressed (achieving the final 95 target) at the end of 2021. There were reductions in each gap of the cascade and the number of people living and diagnosed with HIV is stabilizing. However, the percentage diagnosed and receiving treatment has plateaued under 92% since 2015 after a large fall in the number untreated over 2011–2014. Changes in the cascade gaps coincided with a slow increase in HIV notifications from 2004 to 2014, followed by a slow decline to 2019, and then a rapid fall. Similar trends were found for the estimates of new HIV infections. Most metrics showed substantial improvement since 2004, particularly after the emergence of COVID‐19 in 2020, with the incidence prevalence ratio falling below the UNAIDS global target of 0.03 in 2019.

**Conclusions**: While Australia saw substantial progress in the HIV cascade and achieved the 90‐90‐90 targets in 2020, reaching all 95‐95‐95 targets by 2025 is not guaranteed. Despite large falls in new infections, particularly since the start of the COVID‐19 pandemic, gaps in the cascade related to diagnosis and treatment remain. Further efforts are needed to achieve the UNAIDS targets and end HIV as a public health threat in Australia.

### OAC0103 Characterizing HIV incidence among people who inject drugs engaged with harm‐reduction programmes in four provinces in South Africa

R. Perry^1^, A. Artenie ^1^, A.L. McNaughton^1^, T. Jankie^2^, M. Mahaso^2^, P. Vickerman^1^, A. Scheibe^3,4^

^1^University of Bristol, Bristol, United Kingdom, ^2^NACOSA, Cape Town, South Africa, ^3^Durban University of Technology, Durban, South Africa, ^4^TB HIV Care, Cape Town, South Africa

**Background**: HIV incidence among people who inject drugs (PWID) in South Africa has never been estimated, yet it is essential for informing prevention efforts and tracking progress towards ending HIV/AIDS by 2030. We estimated HIV incidence and associated risk factors among PWID engaged with harm‐reduction services in four provinces in South Africa.

**Methods**: Programmatic data over April 2019–March 2022 were obtained from harm‐reduction services funded by the Networking HIV and AIDS Community of South Africa (NACOSA), which serve PWID in four provinces (Gauteng, KwaZulu‐Natal, Western Cape and Eastern Cape). At each visit, clients who were not known to be HIV positive were offered HIV testing. They also self‐reported socio‐demographic characteristics, drug use patterns and uptake of harm‐reduction services. HIV incidence was estimated using the person‐time method among PWID at‐risk of infection tested at least twice.

**Results**: Data were available for 31,873 PWID, of whom 2457 (7.7%) were initially HIV negative and had ≥2 HIV tests, forming the sample for this study. At baseline, most were male (90.2%), Black (72.2%), homeless or unstably housed (62.6%) and used heroin (97.3%). Median age was 30 yrs and few (7.9%) ever received opioid agonist treatment (OAT). Three hundred (12.2%) PWID acquired HIV over 2190.2 person‐years, resulting in an HIV incidence of 13.7/100 person years (95% CI: 12.2–15.3). The risk of HIV acquisition varied by province, being higher in Gauteng and KwaZulu‐Natal compared to Western and Eastern Capes, and by age, being higher in younger relative to older PWID (Table). PWID who have received OAT and a greater number of harm‐reduction packs had lower risks of HIV acquisition (Table).

**Conclusions**: Our results indicate that HIV incidence among PWID engaged with harm‐reduction services in South Africa is very high and illustrate the value of using programmatic data to monitor the HIV epidemic in this vulnerable population. An urgent expansion of prevention services is needed.

**Table:** 

**Abstract OAC0103‐Table 1**.							
Characteristic	Strata	HIV incidence (95% CI)	Incidence rate ratio (95% CI)	Characteristic	Strata	HIV incidence (95% CI)	Incidence rate ratio (95% CI)
Province	Gauteng	18.9 (16.5 −21.6)	Ref.	Gender	Male	14.2 (12.6 −15.9)	Ref.
	KwaZulu‐Natal	17.1 (13.3 −21.7)	0.9 (0.7−1.2)		Female	10.1 (6.7 −14.8)	0.7 (0.5−1.1)
	Western Cape	3.4 (2.1−5.2)	0.2 (0.1−0.3)	Use of opioid substitution therapy	No, never	14.7 (13.2 −16.6)	Ref.
	Eastern Cape	6.3 (3.2−11.2)	0.3 (0.2−0.6)		Yes, prior to the first HIV test	8.4 (2.1 −22.9)	0.6 (0.1−1.7)
Age (years)	17‐27	18.5 (15.2−22.2)	Ref.		Yes, following the first HIV test	3.8 (1.7−7.6)	0.3 (0.1−0.5)
	28–30	15.7 (12.3 −19.8)	0.9 (0.6−1.2)	Number of harm‐reduction packs received	0−2	17.5 (15.1−20.1)	Ref.
	31–35	13.7 (10.9 −16.9)	0.7 (0.6−1.0)		3−5	10.3 (8.3−12.6)	0.6 (0.5−0.8)
	36–75	7.4 (5.4−9.9)	0.4 (0.3−0.6)		≥6	8.7 (5.4−13.3)	0.5 (0.3−0.8)

### OAC0104 The effect of unplanned care interruptions on the mortality of adults resuming antiretroviral therapy in South Africa: a survival analysis

H. Moolla ^1^, M.‐A. Davies^1^, C. Davies^2^, J. Euvrard^1^, H.W. Prozesky^3^, M.P. Fox^4^, C. Orrell^5^, P. von Groote^6^, L.F. Johnson^1^

^1^Centre for Infectious Disease Epidemiology and Research, University of Cape Town, Cape Town, South Africa, ^2^Division of Epidemiology and Biostatistics, Faculty of Medicine and Health Sciences, Stellenbosch University, Cape Town, South Africa, ^3^Stellenbosch University, Department of Medicine, Cape Town, South Africa, ^4^Health Economics & Epidemiology Research Office, Department of Internal Medicine, University of the Witwatersrand, Johannesburg, South Africa, ^5^Desmond Tutu HIV Centre, Institute of Infectious Diseases and Molecular Medicine & Department of Medicine, University of Cape Town, Cape Town, South Africa, ^6^Institute of Social and Preventive Medicine (ISPM), University of Bern, Bern, Switzerland

**Background**: Interrupting antiretroviral therapy (ART) is associated with adverse outcomes, such as viral resistance, AIDS‐defining illnesses and mortality. However, little is known about the mortality of those resuming ART after unplanned interruptions. The objective of this study is to estimate the relative rate of mortality among adults resuming ART after unplanned interruptions compared to those who do not interrupt care.

**Methods**: We included data from 44,386 adults with HIV initiating ART between 2014 and 2019 at four South African cohorts of the International epidemiology Databases to Evaluate AIDS. We defined care interruption as a gap in contact longer than 180 days. Observation time prior to interruption was allocated to the “no interruption” group. Observation time after the first interruption was allocated to one of two groups based on whether the first interruption occurred before or after 6 months of ART. We determined vital status from clinic records and linkage to the National Population Register. Cox regression was used to estimate hazard ratios, which were adjusted for the effects of sex, baseline age, baseline CD4 count, year of ART initiation and cohort.

**Table:** 

**Abstract OAC0104‐Table 1. Adjusted hazard ratios for predictors of mortality**					
Variable		Person‐years of observation	Deaths	Crude mortality rate per 1000 person‐years (95% CI)	Adjusted hazard ratio (95% CI)
**Interruption status**	No ART interruption	62,228	484	7.78 (7.11–8.50)	1.00
	Interruption within the first 6 months	10,835	206	19.01 (16.59–21.79)	3.08 (2.58–3.68)
	Interruption after the first 6 months	6699	107	15.97 (13.21–19.30)	2.47 (1.96–3.11)
**Sex**	Female	54,724	410	7.49 (6.80–8.25)	1.00
	Male	25,038	387	15.46 (13.99–17.08)	1.47 (1.27–1.70)
**CD4 count at ART initiation (cells/mm^3^)**	500+	11,851	55	4.64 (3.56–6.04)	1.00
	350–499	18,656	110	5.90 (4.89–7.11)	1.31 (0.94–1.81)
	200–349	24,547	192	7.82 (6.79–9.01)	1.60 (1.18–2.17)
	<200	24,708	440	17.81 (16.22–19.55)	3.00 (2.25–4.00)

**Results**: There were 79,762 person‐years of observation and 797 deaths. In total, 12,601 people interrupted care, of whom 7038 interrupted within the first 6 months of ART. Those resuming ART experienced increased mortality compared to those without an interruption: those whose first interruption occurred within 6 months of starting ART had a 208% (95% CI: 158%–268%) increase in mortality, and those whose first interruption occurred after 6 months had a 147% (95% CI: 96%–211%) increase in mortality. Table 1 shows adjusted hazard ratios for selected variables.

**Conclusions**: The substantially increased mortality of those resuming ART after an interruption highlights the need to prioritize and support retention in care, particularly during the first 6 months of ART.

### OAC0105 The impact of same‐day and rapid ART initiation under the Universal Health Coverage programme on HIV outcomes in Thailand

S. Teeraananchai ^1,2^, D.C. Boettiger^3^, C. Lertpiriyasuwat^4^, R. Triamwichanon^5^, P. Wareechai^5^, P. Benjarattanaporn^6^, N. Phanuphak^7^

^1^Department of Statistics, Faculty of Science, Kasetsart University, Bangkok, Thailand, ^2^Biomedical Data Science Program, Faculty of Science, Kasetsart University, Bangkok, Thailand, ^3^Kirby Institute, University of New South Wales, Sydney, Australia, ^4^Bureau of AIDS and STIs, Department of Disease Control, Ministry of Public Health, Nonthaburi, Thailand, ^5^National Health Security Office, Bangkok, Thailand, ^6^Joint United Nations Programme on HIV/AIDS, Bangkok, Thailand, ^7^Institute of HIV Research and Innovation, Bangkok, Thailand

**Background**: Antiretroviral therapy (ART) initiation regardless of CD4 count has been recommended since 2014 in Thailand, with same‐day ART initiation recommended in 2021. We assessed HIV outcomes among Thai people living with HIV (PLHIV) by time from HIV diagnosis to starting ART under the Universal Health Coverage (UHC) programme and determined factors associated with virological failure (VF).

**Methods**: PLHIV aged ≥15 years initiating ART between 2014 and August 2022 were included and categorized into four groups based on duration from HIV diagnosis (or registration) to ART initiation: (1) same‐day or 2–7 days; (2) <1 month; (3) 1–3 months; and (4) >3 months. VF was defined as viral load (VL) ≥1000 copies/ml after at least 6 months of ART. Factors associated with VF were analysed using a competing risk model considering death and loss to follow‐up (LTFU) as competing events. Vital status was confirmed with Death Registry.

**Results**: Of 229,171 PLHIV who started ART, 65% had date of HIV diagnosis recorded. Median (IQR) age was 34 (26–43) years and pre‐ART CD4 count was 232 (77–419) cells/mm^3^. ART initiation happened same‐day in 17%, 2–7 days in 6%, <1 month in 23%, 1–3 months in 25% and >3 months in 30%. ART initiation within 7 days significantly increased from 19% during 2014–2016 to 30% during 2020–2022. ART initiation within 7 days resulted in the lowest mortality (10%: 1.31 [95% CI 1.27–1.36] per 100 person‐years [PY]), but the highest rate of LTFU (8%: 2.29 [95% CI 2.22–2.36] per 100 PY) when compared with others initiating ART groups. VF occurred with a rate of 3.44 (95% CI 3.40–3.489) per 100 PY. PLHIV initiating ART within 1 month were at lower risk of VF (aSHR 0.79, 95% CI 0.75–0.81) when compared to ART initiation >3 months after diagnosis/registration.

**Conclusions**: ART initiation within 7 days became more common in Thailand over time although this occurred in less than one‐third of PLHIV in the last 3 years of the UHC programme. ART initiation within 7 days significantly reduced mortality. ART initiation within 1 month significantly lowered the risk of VF. To further optimize health outcomes, innovative strategies are urgently needed to implement earlier ART initiation in Thailand.

### OAC0202 COVID‐19 vaccine effectiveness by HIV status and injection drug use history

J.H. Puyat ^1,2,3^, J. Wilton^1^, A. Fowokan^1^, N.Z. Janjua^1,2^, J. Wong^1,2^, T. Grennan^1,2^, C. Chambers^4^, A. Kroch^5^, C.T. Costiniuk^6^, C.L. Cooper^7^, D. Lauscher^8^, M. Strong^9^, A.N. Burchell^10,4,11^, A. Anis^2^, H. Samji^1,12^

^1^British Columbia Centre for Disease Control, Vancouver, Canada, ^2^University of British Columbia, School of Population and Public Health, Vancouver, Canada, ^3^Centre for Health Evaluation and Outcome Sciences, St Paul's Hospital, Vancouver, Canada, ^4^University of Toronto, Dalla Lana School of Public Health, Toronto, Canada, ^5^The Ontario HIV Treatment Network, Toronto, Canada, ^6^McGill University Health Centre, Department of Medicine, Division of Infectious Diseases and Chronic Viral Illness Service, Montreal, Canada, ^7^University of Ottawa, Department of Medicine, Ottawa, Canada, ^8^CIHR Canadian HIV Trials Network, Vancouver, Canada, ^9^PAN, Vancouver, Canada, ^10^University of Toronto, Department of Family and Community Medicine, Faculty of Medicine, Toronto, Canada, ^11^St. Michael's Hospital, Unity Health, MAP Centre for Urban Health Solutions, Li Ka Shing Knowledge Institute, Toronto, Canada, ^12^Simon Fraser University, Faculty of Health Sciences, Burnaby, Canada

**Background**: Some evidence suggests that people living with HIV (PLWH) and people who inject drugs (PWID) may experience lower COVID‐19 vaccine effectiveness (VE). This lower VE may be due to a direct impact of HIV acquisition/substance use on immune function, and/or an indirect effect via other comorbidities that are common in these populations. Our objective was to assess COVID‐19 VE by HIV status and injection drug use (IDU) history.

**Methods**: We applied validated algorithms to administrative datasets in the British Columbia COVID‐19 Cohort (BCC19C) to create a population‐based cohort of PLWH and a matched HIV‐negative cohort, and to ascertain the history of IDU. We included individuals who received RT‐PCR testing for SARS‐CoV‐2 between 15 December 2020 and 21 November 2021. A test‐negative study design was used to estimate VE. Analyses were performed using logistic regression with crossed terms for vaccination status, PLWH status and IDU history, adjusting for socio‐demographics, calendar time and co‐morbidities.

**Results**: The analysis included 2700 PLWH and 375,043 matched HIV‐negative individuals—of whom 40.7% and 4.3% had a history of IDU, respectively. VE with two doses was lower among PLWH with IDU history (65.8%, 95% CI = 43.5%–79.3%) than PLWH without (80.3%, 95% CI = 62.7–89.6), and VE increased slower and decreased more quickly in the former, although confidence intervals were wide. The finding of lower VE among people with IDU history was also present in the HIV‐negative cohort but was more pronounced among PLWH.

**Table:** 

**Abstract OAC0202‐Table 1**.				
	PLWH		HIV‐negative individuals	
	History of IDU	No history of IDU	History of IDU	No history of IDU
First dose	44.1 (−25.8 to 75.2)	−	36.5 (22.1−48.2)	56.3 (52.7−59.6)
Second dose (days)				
≥ 7*	65.8 (43.5−79.3)	80.3 (62.7−89.6)	82.1 (79.9−84.1)	88.6 (88.2−89.0)
7−59	65.7 (17.9−85.7)	80.3 (50.9−92.1)	87.7 (85.1−89.9)	91.5 (91.1−92.0)
60−89	91.3 (62.3−98.0)	85.9 (56.8−95.4)	83.9 (80.3−86.9)	89.9 (89.4−90.3)
90−119	65.9 (21.6−85.2)	88.1 (63.6−96.1)	79.7 (75.2−83.4)	87.8 (87.2−88.4)
120−179	42.4 (−17.8 to 71.8)	64.0 (15.7−84.7)	71.8 (65.2−77.1)	84.6 (83.8−85.4)

*Indicates result derived from a separate model with vaccination status categorized as unvaccinated and 2nd dose (>7 days).

**Conclusions**: PWID may experience lower VE against COVID‐19 acquisition, particularly for people who are also living with HIV. These findings highlight the convergence of the dual public health crises and the importance of prioritizing PLWH and PWID for booster doses. The higher prevalence of IDU among PLWH may partly explain our previously published finding of slower buildup/quicker waning among PLWH overall compared to HIV‐negative individuals.

### OAC0203 Mpox vaccination among gay, bisexual and other men who have sex with men in the United States, September–December 2022

T. Carpino ^1^, K. Atkins^1^, K. Delaney^2^, W. Abara^3^, M. Hannah^4^, E. Eschliman^5^, O.W. Edwards^4^, Y. Ogale^6^, S. Vos^7^, A. Rao^1^, S. Murray^8^, S. Baral^1^, T. Sanchez^4^

^1^Johns Hopkins University, Epidemiology, Baltimore, United States, ^2^Centers for Disease Control and Prevention, Division of HIV Prevention, Atlanta, United States, ^3^Centers for Disease Control and Prevention, Division of Sexually Transmitted Disease Prevention, Atlanta, United States, ^4^Emory University, Atlanta, United States, ^5^Johns Hopkins University, Health, Behavior, and Society, Baltimore, United States, ^6^Centers for Disease Control and Prevention, Division of Sexually Transmitted Disease Prevention & Epidemic Intelligence Service, Atlanta, United States, ^7^Centers for Disease Control and Prevention, Division of Violence Prevention & Epidemic Intelligence Service, Atlanta, United States, ^8^Johns Hopkins University, Mental Health, Baltimore, United States

**Background**: During the 2022 mpox outbreak, there was disproportionate mpox risk among gay, bisexual and other men who have sex with men (MSM), especially Hispanic men, non‐Hispanic Black men and people living with HIV (PLWH). These communities reported varying degrees of vaccine access, uptake and 2‐dose completion. Studying individual and structural determinants of vaccination can inform vaccination programme implementation for both new and ongoing vaccination efforts.

**Methods**: A convenience sample of 3041 cisgender MSM aged 15+ years participated in a cross‐sectional survey including socio‐demographic and behavioural factors related to mpox vaccination uptake and 2‐dose completion from 09/2022 to 12/2022 as part of the American Men's Internet Survey. Mpox‐related stigma was measured using a 9‐item scale pooled as a binary “any” versus no reported mpox stigma variable. We conducted bivariate analyses to explore associations with:

1. receiving any dose and
2. completion of 2‐dose mpox vaccination.

We further conducted unadjusted and adjusted logistic regression with age group and race/ethnicity. Associations between self‐reported mpox vaccination and HIV PrEP use were explored among the subset of MSM at risk for HIV acquisition.

**Results**: 33.3% (*n* = 1006) of participants received at least one dose of mpox vaccination; 16.6% (167/1006) received only one dose, 83.1% (*n* = 836) received two doses and 0.3% (*n* = 3) reported >two doses. HIV status was significantly associated (*p*<0.01) with any mpox vaccination, but not mpox vaccine completion (*p*>0.05). Factors associated with both mpox vaccination and completion included STI screening, mpox‐related stigma, awareness, concern and HIV PrEP visits for individuals at risk for HIV acquisition.

**Figure:**
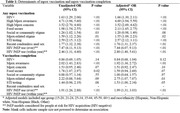
**Abstract OAC0203‐Table 1**.

**Conclusions**: The associations between mpox vaccination and STI testing and PrEP, but low overall uptake, suggest opportunities for further integration of mpox vaccination with sexual health services. However, broader uptake will also require complementary vaccine delivery strategies to reach MSM less connected to sexual health delivery systems.

### OAC0204 Risk of hepatitis C infection following bacterial sexually transmissible infections among gay and bisexual men in Australia 2016–2020

B. Harney ^1,2,3^, R. Sacks‐Davis^1,2^, P. Agius^1^, D. van Santen^1,2,4^, M. Traeger^1,2^, A. Wilkinson^1,2^, J. Asselin^1^, C. El‐Hayek^1,2^, C. Fairley^5,6^, N. Roth^7^, M. Bloch^8^, G. Matthews^9,10^, B. Donovan^9^, R. Guy^9^, M. Stoove^1,2^, M. Hellard^1,2,3^, J. Doyle^1,2,3^

^1^Burnet Institute, Melbourne, Australia, ^2^Monash University, Melbourne, Australia, ^3^Alfred Health and Monash University, Department of Infectious Diseases, Melbourne, Australia, ^4^Public Health Service of Amsterdam, Amsterdam, Netherlands, ^5^Melbourne Sexual Health Centre, Melbourne, Australia, ^6^Monash University, Central Clinical School, Melbourne, Australia, ^7^Prahran Market Clinic, Melbourne, Australia, ^8^Holdsworth House Medical Practice, Sydney, Australia, ^9^University of New South Wales, Kirby Institute, Sydney, Australia, ^10^St Vincent's Hospital, Sydney, Australia

**Background**: In Australia, the incidence of hepatitis C virus (HCV) infection has declined among gay and bisexual men (GBM) with HIV since 2015 and is low among GBM using HIV pre‐exposure prophylaxis (PrEP). However, ongoing HCV testing, and treatment, is required to sustain HCV elimination. Annual HCV testing is recommended in Australia for all GBM with HIV and GBM using PrEP regardless of sexual or substance use behaviours. Bacterial sexually transmissible infections (STIs) are associated with similar behaviours as HCV among GBM, and therefore, may be useful to guide more person‐centred and tailored HCV testing. To examine this, we measured the association between STIs and HCV from 2016 to 2020.

**Methods**: Data were from a national network of primary care and sexual health clinics participating in the Australian Collaboration for Coordinated Enhanced Sentinel Surveillance (ACCESS). GBM included had at least one HCV antibody negative test during the observation period and ≥1 subsequent HCV test. Discrete time modelling estimated the association between a positive syphilis, rectal chlamydia and rectal gonorrhoea diagnosis in the preceding 2 years on incident HCV infection, reported as an adjusted hazard ratio (aHR). Analyses were stratified by GBM with HIV and GBM prescribed PrEP and adjusted for age and the number of each STI test undertaken.

**Results**: Among 6529 GBM with HIV, 92 had an incident HCV infection which was associated with a syphilis infection (aHR 1.99, 95% CI: 1.11–3.56). Of 13,061 GBM prescribed PrEP, 48 had an incident HCV infection, which was associated with rectal chlamydia (aHR 2.73, 95% CI: 1.40–5.30) and rectal gonorrhoea (aHR 2.58, 95% CI: 1.30–5.13).

**Conclusions**: HCV was associated with bacterial STIs among GBM with HIV and GBM using PrEP. These findings suggest that an STI diagnosis should prompt a conversation about HCV testing, and that more frequent HCV testing may be justified among GBM with STIs.

### OAC0205 Integration of PrEP services and assisted partner notification into an STI clinic in Lilongwe, Malawi

C. Pedersen ^1,2^, J. Chen^1^, M. Matoga^2^, B. Ndalama^2^, E. Mathiya^2^, T. Munthali^2^, N. Nyirenda^2^, N. Bonongwe^2^, E. Jere^2^, D. Yatina^2^, H. Nkhata^2^, M. Kacheyo^2^, Z. Mpande^3^, M. Hosseinipour^1,2^, I. Hoffman^1,2^, S. Rutstein^1^

^1^UNC Chapel Hill, Institute for Global Health and Infectious Disease, Chapel Hill, United States, ^2^UNC Project Malawi, Lilongwe, Malawi, ^3^Lighthouse Trust, Lilongwe, Malawi

**Background**: HIV pre‐exposure prophylaxis (PrEP) has been integrated into sexually transmitted infection (STI) care in Malawi/sub‐Saharan Africa (SSA); however, the success and prevention impact of integration has not been evaluated. Expanding prevention services to sexual partners of persons with STIs is another opportunity to increase PrEP reach. We evaluated an integrated PrEP and assisted partner notification (aPN) programme in an STI clinic in Lilongwe, Malawi.

**Methods**: We enrolled “index” participants who were initiating oral PrEP at the Bwaila District Hospital STI Clinic (≥15 years, STI symptoms or exposure, HIV‐seronegative). Participants completed surveys and provided contact information for sexual partners. Clinic staff contacted named partners who did not report to clinic within 14 days for STI treatment and HIV testing. Returning partners were screened for PrEP eligibility and, if interested and eligible, initiated PrEP and enrolled in the study.

**Results**: One hundred and seventy‐five index participants enrolled between March and December 2022. The median age was 27 (interquartile range [IQR]: 23–32) and most were male (110/175; 63%). Twenty‐one percent (36/175) were adolescent girls and young women. In the preceding month, 40% (70/175) reported exchanging sex for goods/money/favours and 13% (23/175) had ≥1 known HIV‐positive partner. Before enrolment, 34% (60/175) had heard of PrEP. Forty‐nine percent (86/175) of participants provided contact information for 100 sexual partners. Fifty‐eight partners returned to clinic: 34% (20/58) were HIV‐seropositive and ineligible (4 newly diagnosed; 16 previously diagnosed). Of eligible partners, 71% (27/38) initiated PrEP and enrolled, and 29% (11/38) declined PrEP and/or study procedures. Median age of enrolled partners was 26 (IQR: 22–31) and most were female (20/27; 74%).

**Conclusions**: Ours is the first to demonstrate successful integration of PrEP within an STI clinic in SSA and highlights the benefits of aPN for promoting partner engagement in HIV prevention services. Although STI clinic‐based PrEP does not explicitly target key populations, more than half of indexes fell into at least one priority category (exchanged sex for goods/money/favours or young women), and many were in serodiscordant relationships. Engaging young heterosexual men in PrEP care has been a historically under‐examined opportunity to interrupt transmission and may be particularly powerful when coupled with PrEP referral for female partners.

### OAC0302 Enhanced linkage to care following home‐based HIV testing improves linkage, ART initiation and retention, and 12‐month viral suppression—the Ekkubo study: a cluster randomized trial in Uganda

S.M. Kiene ^1,2^, R. Naigino^1,2^, K. Schmarje Crockett^1^, M. Ediau^1,2^, A. Anecho^2^, C.‐D. Lin^3^, N.A Menzies^4^, M. Bateganya^5^, S. Sekamatte^6^, S.C. Kalichman^7^, R.K. Wanyenze^2^

^1^San Diego State University, School of Public Health, San Diego, United States, ^2^Makerere University School of Public Health, Kampala, Uganda, ^3^San Diego State University, Department of Mathematics and Statistics, San Diego, United States, ^4^Harvard T.H. Chan School of Public Health, Department of Global Health and Population, Boston, United States, ^5^USAID, Msasani, Tanzania, United Republic of, ^6^Gombe Hospital, Gombe, Uganda, ^7^University of Connecticut, Institute for Collaboration on Health, Intervention, and Policy, Storrs, United States

**Background**: HIV testing in the universal test and treat (UTT) era in settings where immediate ART initiation is not feasible may require support for care engagement to achieve viral suppression. The Ekkubo study, a cluster randomized trial in rural Uganda, tested an enhanced linkage to care intervention versus standard‐of‐care+referrals to care in the context of home‐based HIV testing.

**Methods**: Between November 2015 and March 2020, home‐based HIV testing was conducted in pair‐matched villages randomized to intervention or comparator arms across four districts in central Uganda. Individuals aged 15–59 newly diagnosed HIV positive or previously diagnosed but never linked to care in intervention villages received three brief sessions at‐home and one phone follow‐up focused on overcoming barriers to care, eliciting a plan to engage in care, and social support resources to address stigma and other needs. Those in standard‐of‐care+ villages received two brief sessions at‐home referring them to care and reinforcing the referral. The primary outcome was viral suppression (<20 copies/ml) at 12 months. Secondary outcomes included linkage, ART initiation, time to ART initiation and ART retention. Intention‐to‐treat analyses accounted for the cluster design. Trial registration: NCT02545673

**Results**: In the 56 clusters/villages (28 per arm), 284 (62.3% female) and 283 (62.9% female) individuals were enrolled in intervention and comparator arms, respectively. Average cluster size was 10.31 (SD 13.97, range 1–57). Average age was 30.77 (SD 9.45), 543 (95.8%) were newly diagnosed. At 12 months, 134/284 (47.2%) and 114/283 (40.3%) of participants in intervention and comparator clusters, respectively, achieved VL <20 (adjOR, 1.60, 95% CI 1.13–2.26, *p* = 0.008). Intervention participants also did better for linkage to care (assessed as a second visit; adjOR, 1.65, 95% CI 1.09–2.49, *p* = 0.017) and ART initiation (adjOR, 1.97, 95% CI 1.33–2.90, *p*<0.001) and retention (adjOR 4.16, 95% CI 1.14–13.87, *p* = 0.015) than standard‐of‐care+ participants, but there was no difference for time to ART initiation among those who initiated (adjHR 1.06, 95% CI 0.78–1.46, *p* = 0.69). Twelve‐month study retention was >84% in both arms, including deaths.

**Conclusions**: Focused linkage support at diagnosis and shortly thereafter increased linkage, ART initiation, retention and viral suppression. This intervention may have utility with populations experiencing linkage and viral suppression challenges.

### OAC0303 The effect of primary healthcare on AIDS: a cohort study of 3.4 million individuals in Brazil

P. Pinto ^1,2^, A. Silva^1,2^, I. Lua^1,2^, G. Jesus^1,2^, L. Magno^3^, C. Santos^2^, M. Ichihara^2^, M. Barreto^2^, L.E. Souza^1^, J. Macinko^4^, I. Dourado^1^, D. Rasella^1,5^

^1^Institute of Collective Health, Federal University of Bahia (ISC/UFBA), Salvador, Brazil, ^2^The Centre for Data and Knowledge Integration for Health (CIDACS), Salvador, Brazil, ^3^State University of Bahia (UNEB), Department of Life Sciences, Salvador, Brazil, ^4^Fielding School of Public Health of University of California (UCLA), Departments of Health Policy and Management and Community Health Sciences, Los Angeles, United States, ^5^ISGlobal, Hospital Clínic—Universitat de Barcelona, Barcelona, Spain

**Background**: Although primary healthcare (PHC) is part of the AIDS care and prevention services, the effect of PHC on AIDS outcomes is poorly understood in low‐ and middle‐income countries (LMICs). We evaluated the impact of one of the largest PHC programmes in the world, the Brazilian Family Health Strategy (FHS), on AIDS incidence and mortality using a cohort of 3.4 million individuals over a 9‐year study period in Brazil.

**Methods**: We analysed AIDS data from individuals aged 13 years or older who were members of a nationwide cohort of the poorest Brazilian people (The 100 Million Brazilians Cohort) from 1 January 2007 to 31 December 2015 and compared residents in municipalities with no FHS coverage with residents in municipalities with full FHS coverage. We used multivariable Poisson regressions, adjusted for all relevant demographic, socio‐economic and municipal variables, and weighted with inverse probability of treatment weighting (IPTW), to estimate the effect of FHS on AIDS incidence and mortality rates. We also estimated effects by sex, age, race/ethnicity and by AIDS municipal incidence. We conducted sensitivity and triangulation analyses.

**Results**: FHS coverage was associated with lower AIDS incidence (rate ratio [RR] 0.76, 95% CI 0.68–0.84) and mortality (0.68, 0.56–0.82). The effect of FHS on incidence was greater among females (0.70, 0.61–0.82) and Black population (0.64, 0.45–0.92). The effect of FHS on mortality was greater among males (0.64, 0.49–0.83). For both outcomes, the effect of FHS coverage was stronger among people aged 35 years or older (0.62, 0.53–0.72—incidence and 0.56, 0.43–0.72—mortality), although a lower incidence was also observed among people aged 13–34 years (0.83, 0.72–0.96).

**Conclusions**: FHS coverage impacts AIDS morbidity and mortality among the most vulnerable populations in Brazil. Our results show the importance of expanding and strengthening PHC in LMICs to contribute to the goal of ending AIDS by 2030, including the improvement of infrastructure and human resources for the decentralization of care, treatment and monitoring of people with HIV/AIDS.

### OAC0304 Initiation of pre‐exposure prophylaxis (PrEP) among key populations in PEPFAR‐supported countries during 2019–2022

H. B. Demeke ^1^, R. Nelson^1^, G. Djomand^1^, U. Ijeoma^1^, W. Ivy, III^1^, A. Lyn^1^, M. Michalak^1^, B. Gichuchi^1^, D. Selenic^1^, T. N. Hoang^2^, T. Bingham^1^

^1^Centers for Disease Control and Prevention, Center for Global Health, Atlanta, United States, ^2^Centers for Disease Control and Prevention, Vietnam, Hanoi, Vietnam

**Background**: Pre‐exposure prophylaxis (PrEP) is a highly effective intervention that can prevent HIV acquisition. Through the U.S. President's Emergency Plan for AIDS Relief (PEPFAR), the US Centers for Disease Control and Prevention (CDC) supports PrEP programmes for key populations (KP) (i.e. female sex workers [FSW], men who have sex with men [MSM], transgender people [TG], people who inject drugs [PWID] and people in prisons and other enclosed settings). UNAIDS’ current goal is to reach 10 million people with PrEP by 2025. We examined CDC's scale‐up of PrEP during Covid‐19 among KP beneficiaries to evaluate progress towards the UNAIDS goal.

**Methods**: Using programmatic data from PEPFAR's Monitoring, Evaluation and Reporting system, we assessed the number and percentage of KP beneficiaries who initiated PrEP, by KP group and by year, from 2019 to 2022 in 30 countries. We assessed trends by year among 27 countries with complete testing and PrEP initiation data. We also consulted narrative reports of programme services to understand changes made to PrEP delivery.

**Results**: CDC supported 1,371,984 PrEP initiations during 2019–2022, of whom 520,981 identified as KP. The percentage of HIV‐negative KP accepting PrEP increased by 532%, from 3.1% in 2019 to 19.6% in 2022. The largest relative increases in uptake were among PWID and prisoners, although the absolute percentages were <10%. Although FSW and MSM showed highest uptake in 2022, over 70% of these KP did not initiate PrEP.

**Table:** 

**Abstract OAC0304‐Table 1**.					
	PrEP initiation among key population beneficiaries supported by CDC in 27 PEPFAR‐funded countries, 2019–2022. Numbers of KP enrolled in PrEP/number of HIV‐negative KP (%)				
Key population groups	2019	2020	2021	2022	% Increase in proportion of HIV‐negative KP accepting PrEP from 2019 to 2022
Female sex workers (FSW)	13,180/245,659 (5.4%)	34,978/508,598 (6.9%)	91,521/700,578 (13.1%)	163,638/695,078 (23.5%)	335%
Men who have sex with men (MSM)	66,059/54,209 (11.2%)	118,555/238,672 (7.8%)	52,350/381,094 (13.7%)	88,084/340,985 (25.8%)	130%
People who inject drugs (PWID)	690/90,619 (0.8%)	2573/184,876 (1.4%)	9277/174,085 (5.3%)	16,866/178,902 (9.4%)	1075%
People in prisons	5514/269,276 (0.2%)	589/146,251 (0.4%)	8842/150,051 (5.9%)	7086/195,015 (3.6%)	1700%
Transgender people	183/1801 (10.2%)	635/2250 (28.2%)	1516/4959 (30.6%)	2341/11,406 (20.5%)	101%
Total	20,626/661,564 (3.1%)	57,330/1,080,647 (5.3%)	163,506/1,410,767 (11.6%)	278,015/1,421,386 (19.6%)	532%

**Conclusions**: Despite Covid‐19 disruptions in service delivery, we observed substantial PrEP scale‐up in CDC programmes. Programme modifications, such as HIV self‐testing, telemedicine and community‐based deliveries, may have facilitated PrEP expansion during this time. To reach UNAIDS’ global goal, additional innovations, such as long‐acting injectable PrEP, event‐driven PrEP and programmes to increase PrEP awareness/literacy among beneficiaries and providers, may increase uptake by KP. Continuous monitoring and evaluation of KP PrEP programmes is critical to meet ambitious UNAIDS global targets.

### OAC0305 Increased HIV prevention coverage among Australian gay and bisexual men with regular partners: results of National Behavioural Surveillance, 2018–22

T. Broady ^1^, C. Chan^2^, J. MacGibbon^1^, L. Mao^1^, J. Rule^3^, H. Paynter^4^, B. Bavinton^2^, M. Holt^1^

^1^Centre for Social Research in Health, UNSW, Sydney, Australia, ^2^Kirby Institute, UNSW, Sydney, Australia, ^3^National Association of People with HIV Australia, Sydney, Australia, ^4^Australian Federation of AIDS Organisations, Sydney, Australia

**Background**: Increasing use of biomedical HIV prevention methods has been reported among gay and bisexual men (GBM) in Australia with casual partners. Less attention has been paid to HIV prevention strategies among GBM with regular partners. We analysed trends in behavioural surveillance data to identify changes in HIV prevention methods by GBM with regular partners, and levels of prevention coverage (and risk).

**Methods**: Behavioural surveillance data from the Gay Community Periodic Survey (collected between 2018 and 22) were analysed. Trends in condom use, condomless sex with regular partners (CAIR) and prevention coverage with regular partners (i.e. use of any effective strategy, e.g. condoms, PrEP and undetectable viral load [UVL]) were assessed using logistic regression. Trends were stratified according to reported sexual activity with casual partners.

**Results**: In total, 24,316 survey responses from participants with regular male partners were included for analysis. The mean age of the sample was 38.2 years, 68.6% were Australian‐born, 90.0% gay‐identified and 8.6% living with HIV. The proportion who reported consistent condom use declined from 12.3% in 2018 to 9.3% in 2022, while any CAIR increased from 67.2% to 74.8% (both *p*<0.001). HIV prevention coverage with regular partners increased from 94.3% in 2018 to 98.4% in 2022 (*p*<0.001). The proportion of GBM with regular partners who were HIV positive and reported UVL remained stable (6.2% to 5.5%, *p* = 0.253), as did the proportion of non‐HIV‐positive GBM whose regular partners were HIV positive with UVL (1.2% to 1.0%, *p* = 0.103). The proportion of participants with regular partners who were HIV negative and on PrEP increased from 16.6% to 34.8% (*p*<0.001); however, this increase in PrEP use was concentrated among participants with regular partners who also reported condomless sex with casual partners (from 38.0% to 68.4%, *p*<0.001).

**Conclusions**: The risk of HIV transmission between regular partners was low and continually decreased between 2018 and 2022. GBM with regular partners increasingly use biomedical methods to prevent HIV transmission, and PrEP use was concentrated among those who also engaged in condomless sex with casual partners.

### OAC0402 No association between in‐utero PrEP exposure and bone mineral density at 36 months of age among mother−infant pairs in Kenya

L. Wu ^1,2^, J. Kinuthia^3^, F. Abuna^3^, J. Dettinger^2^, L. Gomez^2^, E. Mukenyi^3^, G. John‐Stewart^1,2,4,5^, M. Marwa^3^, N. Ngumbau^3^, B. Ochieng^3^, J. Pintye^2,6^

^1^University of Washington, Department of Epidemiology, Seattle, United States, ^2^University of Washington, Department of Global Health, Seattle, United States, ^3^Kenyatta National Hospital, Nairobi, Kenya, ^4^University of Washington, Department of Medicine, Seattle, United States, ^5^University of Washington, Department of Pediatrics, Seattle, United States, ^6^University of Washington, Department of Biobehavioral Nursing and Health Informatics, Seattle, United States

**Background**: Previous studies found that tenofovir‐based ART use during pregnancy among women living with HIV is associated with lower bone mineral density (BMD) in neonates. It is unknown whether these differences persist beyond the neonatal period or exist among neonates born to women without HIV who used tenofovir‐based PrEP during pregnancy.

**Methods**: We utilized data from an ongoing evaluation of perinatal PrEP use in Kenya. In the parent study (NCT03070600), HIV‐negative women were enrolled and offered tenofovir‐based PrEP during pregnancy at 20 public clinics and followed through 9 months postpartum regardless of PrEP status. An extension cohort to evaluate safety outcomes enrolled mother−child pairs at four sites to be followed until the child's fifth birthday. A subset of singleton children aged 36 months with in‐utero PrEP exposure was randomly selected and matched to children without in‐utero PrEP exposure on maternal age, education level, and child sex and age. Whole‐body BMD was measured by dual‐energy X‐ray absorptiometry (DEXA) at Aga Khan University Hospital in Nairobi, Kenya. Linear regression adjusting for matching characteristics was performed to evaluate the relationship between BMD and PrEP exposure.

**Results**: From December 2021 to December 2022, 40 children with in‐utero PrEP exposure and 71 without PrEP exposure had whole‐body BMD measurements. The median age at DEXA scanning was 36.7 months (IQR: 36.2–38.0), 40% of children were female and the median maternal age at delivery was 27.6 years (IQR: 22.1–32.6). The median height for children at DEXA scanning was similar between those with and without PrEP exposure (94.3 vs. 94.0 cm, *p* = 0.455). The median whole‐body BMD for children with and without in‐utero PrEP exposure was 418.5 mg/cm^2^ (IQR 399.2–440.0) and 423.0 mg/cm^2^ (IQR 395.5.0–457.5.0), respectively. There was no difference between mean whole‐body BMD among children with and without in‐utero PrEP exposure (adjusted mean difference −21.6 mg/cm^2^, 95% CI −60.1 to 17.0, *p* = 0.270).

**Conclusions**: PrEP exposure was not associated with BMD or height at 36 months among children with mothers who used PrEP during pregnancy. Our findings suggest that in‐utero PrEP exposure may not impact BMD into early childhood.

### OAC0403 Cofactors of HIV self‐testing and PrEP acceptance among pregnant women at high risk of HIV in Kenya

N.M. Ngumbau ^1^, J. Neary^2^, A. Wagner^3^, F. Abuna^1^, B. Ochieng^1^, J. Dettinger^3^, L. Gómez^2^, M. Marwa^1^, S. Watoyi^1^, E. Nzove^1^, J. Pintye^4^, J. Baeten^5^, J. Kinuthia^1^, G. John‐Stewart^2,3,6,5^

^1^Kenyatta National Hospital, Research and Programs, Nairobi, Kenya, ^2^University of Washington, Department of Epidemiology, Seattle, United States, ^3^University of Washington, Department of Global Health, Seattle, United States, ^4^University of Washington, Biobehavioral Nursing & Health Informatics, Seattle, United States, ^5^University of Washington, Medicine, Seattle, United States, ^6^University of Washington, Pediatrics, Seattle, United States

**Background**: PrEP and HIV self‐testing (HIVST) for male partners are being scaled up within antenatal clinics (ANC). Few data are available on how co‐distribution influences the acceptance of both interventions and the cofactors for PrEP, HIVST or combined PrEP/HIVST use among pregnant women at high risk for HIV.

**Methods**: We utilized data from the PrIMA (NCT03070600) trial in Kenya. Women included in this analysis were determined to be at high HIV risk and offered PrEP and partner HIVST. Characteristics were compared between women who chose: (1) PrEP and HIVST; (2) HIVST‐alone; (3) PrEP‐alone; or (4) declined both (reference), excluding women with partners known to be living with HIV.

**Results**: Among 911 women, the median age was 24 years, 87.3% were married and 13.0% had history of intimate partner violence (IPV); 68.8% accepted HIVST and 18.4% accepted PrEP. Of women accepting HIVST, 84% offered them to partners; 94% of partners used HIVST; 1.2% had a reactive HIVST. Partner HIV testing increased from 20% to 82% and women's knowledge of partner HIV status increased from 4.7% to 82.0% between pregnancy and 9‐months postpartum (*p*<0.001). Overall, 54.7% accepted HIVST‐alone, 4.1% PrEP‐alone and 14.3% both HIVST and PrEP. Compared to women who accepted neither, choosing: (1) HIVST‐alone was associated with being married, participant and partner higher level of education, and residing with partner; (2) PrEP‐alone with lower social support, IPV, not residing with partner, longer time with partner and suspicion of other sexual partners; and (3) PrEP and HIVST was associated with being married, IPV and suspicion that partner had other partners.

**Figure:**
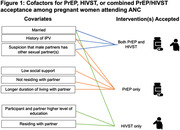
**Abstract OAC0403‐Figure 1**.

**Conclusions**: Understanding factors associated with accepting HIVST, PrEP or both can inform HIV prevention programmes for pregnant women. Strategies to improve women's self‐efficacy to take up HIV prevention interventions are important to reduce incident infections during pregnancy and postpartum.

### OAC0404 Drug−drug interaction between emtricitabine/tenofovir alafenamide (FTC/TAF)‐based PrEP and feminizing hormones in transgender women: peripheral blood mononuclear cells and urine analysis from the iFACT3 study

A. Hiransuthikul ^1,2^, N. Thammajaruk^1^, S. Kerr^3,4^, R. Janamnuaysook^1,5^, S. Nonenoy^1^, P. Hongchookiat^1^, R. Trichavaroj^1^, Y. Tawon^6^, J. Boonruang^1^, N. Teeratakulpisarn^1^, T.R. Cressey^6^, N. Phanuphak^1,5^, the iFACT3 study team

^1^Institute of HIV Research and Innovation (IHRI), Bangkok, Thailand, ^2^Chulalongkorn University, Department of Preventive and Social Medicine, Faculty of Medicine, Bangkok, Thailand, ^3^Chulalongkorn University, Biostatistics Excellence Centre, Faculty of Medicine, Bangkok, Thailand, ^4^HIV‐NAT, Thai Red Cross AIDS Research Centre, Bangkok, Thailand, ^5^Chulalongkorn University, Center of Excellence in Transgender Health (CETH), Faculty of Medicine, Bangkok, Thailand, ^6^Chiang Mai University, AMS‐PHPT Research Collaboration, Faculty of Associated Medical Sciences, Chiang Mai, Thailand

**Background**: We previously observed that plasma tenofovir (TFV) and emtricitabine (FTC) exposures were not significantly different among transgender women when FTC/TAF‐based PrEP was administered with and without feminizing hormone therapy (FHT). Herein, we report the impact on the intracellular TFV‐diphosphate (TFV‐DP) and FTC triphosphate (FTC‐TP) concentrations, the therapeutically active forms, in peripheral blood mononuclear cells (PBMCs), and on TFV/FTC urine concentrations.

**Methods**: Twenty transgender women who had not undergone orchiectomy were enrolled between January and February 2022. FHT (estradiol valerate 2 mg and cyproterone acetate 25 mg) was initiated at baseline and prescribed until week 9, while PrEP (FTC 200 mg/TAF 25 mg) was initiated at week 3 and prescribed until the end of study at week 12. PK sampling for drug‐level measurement was performed at weeks 9 (with FHT) and 12 (without FHT). PBMC samples were collected at 2 and 24 hours after FTC/TAF administration to assess FHT effect on TFV‐DP and FTC‐TP levels; and a 24‐hour urine collection was used to assess FHT effect on TFV/FTC.

**Results**: Eighteen participants completed the PK visits and were included in this analysis. Median (IQR) age and body mass index were 28 (23–32) years and 20.8 (19.9–21.9) kg/m^2^, respectively. The PBMC 2 hour (C_2_) and 24 hour (C_24_) geometric mean ratios (GMRs) (95% CI) at week 9 and week 12 (reference) were as follows: TFV‐DP, 1.04 (0.91−1.19, *p* = 0.59) and 0.96 (0.82−1.13, *p* = 0.65); and FTC‐TP, 0.97 (0.85−1.10, *p* = 0.61) and 0.91 (0.75−1.10, *p* = 0.33) (Figure). Urine GMRs for TFV and FTC were 1.05 (0.84−1.32, *p* = 0.67) and 0.92 (0.75−1.13, *p* = 0.42).

**Figure:**
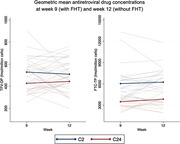
**Abstract OAC0404‐Figure 1**.

**Conclusions**: Intracellular TFV‐DP and FTC‐TP levels in PBMCs and urine TFV and FTC concentrations were comparable when F/TAF‐based PrEP was administered with and without FHT, suggesting no clinically significant drug−drug interaction from FHT towards FTC/TAF‐based PrEP. Tissues rectal measurements of TFV‐DP and FTC‐TP levels are ongoing.

### OAC0405 Experiences of reproductive coercion among women living with HIV in sub‐Saharan Africa, Eastern Europe and Central Asia

C. Lyons ^1^, G. Turpin^1^, S. Brion^2^, K. Dunaway^2^, O. Syarif^3^, P. Looze^3^, K. Lalak^3^, C. Garcia De Leon Moreno^4^, D. Matyushina^4^, L. Sprague^4^, G. Kadziyanhike^5^, M. Godlevskaya^6^, I. Abdulkadir^7^, S. Pavel^8^, S. Bessonov^9^, A. Ouedraogo^10^, N.A.J.C. Vako^11^, J.d.D. Anoubissi^3^, K.A. Dokla^12^, K.A. Hlomewoo^12^, S. Baral^1^, K. Rucinski^13^

^1^Johns Hopkins School of Public Health, Epidemiology, Baltimore, United States, ^2^The International Community of Women Living with HIV (ICW), N/A, United Kingdom, ^3^Global Network of People Living with HIV (GNP+), Amsterdam, Netherlands, ^4^UNAIDS, Geneva, Switzerland, ^5^Zimbabwe National Network of People Living with HIV (ZNNP+), Harare, Zimbabwe, ^6^EVA Network, N/A, Russian Federation, ^7^Network of People Living with HIV /AIDS in Nigeria, Abuja, Nigeria, ^8^Central Asian Association of People Living with HIV, N/A, Kazakhstan, ^9^Kyrgyz Harm Reduction Network, N/A, Kyrgyzstan, ^10^REGIPIV‐BF, N/A, Burkina Faso, ^11^Ivorian Network of People Living with HIV/AIDS (RIP+), Abidjan, Cote D'Ivoire, ^12^RAS+, N/A, Togo, ^13^Johns Hopkins School of Public Health, International Health, Baltimore, United States

**Background**: Evidence suggests that women living with HIV (WLHIV) experience coercion by healthcare providers related to sterilization, contraception/family planning, pregnancy and feeding practices. However, there has been limited quantification of the prevalence and determinants of reproductive coercion among WLHIV.

**Methods**: The People Living with HIV (PLHIV) Stigma Index 2.0 study was implemented in 16 countries in Eastern Europe and Central Asia (EECA) and sub‐Saharan Africa (SSA). Study implementation was led by networks of PLHIV (2020–2022). Interviewer‐administered questionnaires were used to collect self‐reported socio‐behavioural measures among 10,555 cisgender 18+ year old WLHIV. Reproductive coercion in these analyses was categorized based on coercive experiences relating to recent (within last 12 months) sterilization; contraception/family planning; and pregnancy and feeding practices. Multilevel logistic regression models were used to assess hierarchical determinants of reproductive coercion.

**Results**: Among participants in SSA, 0.5% reported sterilization, 1.6% reported coercion of contraception and family planning; and 4.8% reported coercion of pregnancy and feeding practices. Among participants in EECA, 3.2% reported sterilization, 3.9% reported coercion of contraception and family planning; 8.5% reported coercion of pregnancy and feeding practices. Women in EECA had an increased odds of sterilization, coercion related to contraception/family planning, and coercion related to pregnancy and feeding practices compared to women in SSA (Table 1). Across regions, sex workers, migrants and women who inject drugs, and women with disabilities had greater odds of reproductive coercion compared to other WLHIV.

**Conclusions**: In 2022, recent reproductive coercion is common among WLHIV globally. Programmes with trainings on accurate, evidence‐based and person‐centred care for PLHIV, and non‐stigmatizing care practices may improve healthcare provision of reproductive and sexual healthcare among WLHIV. Non‐discrimination protections for WLHIV may support prevention of reproductive coercion and allow accountability when it occurs. Lastly, initiatives to support WLHIV in knowing their rights and how to seek justice may improve the health and wellbeing of WLHIV.

**Figure:**
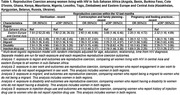
**Abstract OAC0405‐Table 1**.

### OAC0502 A pharmacist‐led oral PrEP refill visit with client HIV self‐testing significantly improved continuation in Kenya

K. Zewdie ^1^, K. Ngure^2^, N. Mugo^3,4^, J. Baeten^3^, D. Mwangi^4^, M. Mwangi^4^, S. Maina^4^, L. Etyang^4^, G. Maina^4^, V. Ogello^4^, E. Owidi^4^, K. Mugwanya^1^

^1^University of Washington, Epidemiology, Seattle, United States, ^2^Jomo Kenyatta University of Agriculture and Technology, Nairobi, Kenya, ^3^University of Washington, Seattle, United States, ^4^Kenya Medical Research Institute, Nairobi, Kenya

**Background**: Delivery of oral pre‐exposure prophylaxis (PrEP) is being scaled up in Africa. However, health system barriers including lengthy visits endanger client continuation. We evaluated the efficiency and impact of direct‐to‐pharmacy PrEP refill visits with HIV self‐testing (HIVST).

**Methods**: Between September 2020 and January 2022, we conducted a quasi‐experimental study of differentiated direct‐to‐pharmacy PrEP refill visits among adult men and women receiving PrEP at four public health HIV clinics in Central Kenya. Two clinics implemented the intervention which included direct‐to‐pharmacy for PrEP refill, HIVST while waiting and 3‐monthly refill visits with pharmacist‐led rapid risk assessment. Two clinics comparable in size and client volume served as controls with the usual standard of care (SOC), which typically includes monthly refills with multiple client room stops. We conducted 80 time and motion studies to determine client time in the clinics. PrEP continuation was evaluated by visit attendance and pharmacy refill records. We used logistic regression to assess the intervention effect on PrEP continuation and the Wilcoxon rank sum test to assess the impact on clinic time.

**Results**: Overall, 746 clients were enrolled: 338 in SOC and 380 in intervention clinics; 57% were female, the median age was 33 and 58% were in serodifferent partnerships. Prior to implementation, intervention and controls, clinics were comparable on client characteristics (female: 51% vs. 47%; median age: 33 vs. 33 yrs) and PrEP continuation (35% vs. 37% at 1 month, and 37% vs. 39% at 3 months; *p*>0.05 for all). The intervention reduced total time spent at the clinic by 35%, the median time spent in SOC was 51 minutes, while in intervention clinics was 33 minutes; *p*<0.001. However, time spent on HIV testing (20 vs. 20 minutes; *p* = 0.50) and pharmacy (8 vs. 8 minutes; *p* = 0.8) was unchanged. Similarly, PrEP continuation was significantly higher in the intervention clinics compared to control clinics: 45% versus 33% at 1 month and 34% versus 25% at 3 months; *p*<0.05 for all.

**Conclusions**: A client‐centred PrEP delivery approach with direct‐to‐pharmacy PrEP refill visits plus client HIV self‐testing reduced clinic visit time and significantly improved PrEP continuation in public health HIV clinics in Kenya.

### OAC0503 Same‐day initiation of oral pre‐exposure prophylaxis is high among adolescent men who have sex with men and transgender women in Brazil

F. Soares ^1^, L. Magno^2^, J. Arrais Pinto Jr.^3^, A. Grangeiro^4^, K. Bruxvoort^5^, D. Greco^6^, I. Dourado^1^

^1^Federal University of Bahia, Collective Health Institute, Salvador, Brazil, ^2^Bahia State University, Department of Life Sciences, Salvador, Brazil, ^3^Fluminense Federal University, Institute of Mathematics and Statistics, Niterói, Brazil, ^4^University of São Paulo, Faculty of Medicine, São Paulo, Brazil, ^5^University of Alabama at Birmingham, School of Public Health, Birmingham, United States, ^6^Federal University of Minas Gerais, Faculty of Medicine, Belo Horizonte, Brazil

**Background**: In recent years, human immunodeficiency virus (HIV) incidence has increased among adolescent men who have sex with men (aMSM) and transgender women (aTGW). Expanding prevention strategies, including daily oral HIV pre‐exposure prophylaxis (PrEP), is crucial for controlling the epidemic. Recently, PrEP offer in the Unified Health System was expanded to individuals aged between 15 and 17 years, sexually active and vulnerable to HIV. This study aimed to analyse the socio‐demographic and behavioural characteristics of aMSM and aTGW initiating oral PrEP in HIV prevention clinics.

**Methods**: PrEP1519 is a prospective, multicentre, open‐label PrEP demonstration cohort study of aMSM and aTGW aged 15–19 living in three large Brazilian capital cities. For this analysis, we included adolescents who enrolled in PrEP1519 from February 2019 to August 2021 and were followed until February 2022 to assess PrEP initiation. Adolescents who visited the PrEP clinics were classified into four groups based on PrEP eligibility and on their decision to use PrEP: (1) ineligible for PrEP; (2) eligible, initiated PrEP at first visit; (3) eligible, initiated PrEP after first visit; and (4) eligible, did not initiate. The groups were described and compared using the chi‐square and Fisher's exact tests.

**Results**: Of the 1254 adolescents who visited the PrEP clinics, 61 (4.9%) were clinically ineligible for PrEP initiation (37.7%) or had a low HIV risk (62.3%). Of the 1193 eligible for PrEP initiation, 1113 (93.3%) initiated PrEP, and 80 (6.7%) did not. Of the 1113 adolescents who initiated PrEP, 87.3% did so on the same day and 12.7% later. Half of those who initiated PrEP in subsequent visits did so within 42 days from the first visit. Despite 90% of the PrEP decliners declaring a low risk of HIV infection, most reported condomless anal sex in the past 6 months (70%).

**Conclusions**: Same‐day PrEP initiation among aMSM and aTGW was high, highlighting the importance and need for promoting effective PrEP offer and access among adolescents with increased vulnerability to HIV.

### OAC0504 PrEP and service utilization among sexual and gender minority youth (SGMY) were significantly improved with an intervention that includes multiple intervention strategies: automated text messages, peer support and coaching

M. Rotheram ^1^, D. Swendeman^2^, S. Comulada^1^, A.T.N. CARES Team, Comprehensive Adolescent Recruitment and Engagement Strategies^3^

^1^University of CA, Los Angeles, Psychiatry, Los Angeles, United States, ^2^University of CA, Los Angeles, Psychiatry, Los Angeles, United States, ^3^University of CA, Los Angeles, Adolescent Medicine, Los Angeles, United States

**Background**: Uptake of both healthcare interventions, especially pre‐exposure prophylaxis (PrEP) or post‐exposure prophylaxis (PEP) with antiretroviral drugs (ARV), and sustained engagement with social services to cope with comorbid conditions of homelessness, substance abuse, mental health disorders or a lack of food, clothing, transportation and toiletries are needed by young people at risk for HIV. This study evaluates the level of intervention needed to improve the uptake of these services.

**Methods**: Gay, bisexual, transgender and gender diverse youth aged 12–24 years old (*N* = 895), who were predominantly Black and Latino, were recruited from 2017 to 2019 from 13 community agencies in Los Angeles and New Orleans and assessed at 4‐month intervals over 24 months with 90%–70% follow‐up. Youth were randomized to interventions designed to support uptake of the HIV Prevention Continuum (linkage to healthcare, uptake and adherence to PrEP, PEP and/or 100% condom use) and hierarchies of needs (housing, income, social relationships, mental health and risks) in a four‐arm factorial design: (1) automated messaging and monitoring (AMMI) via text‐messages (*n* = 313); (2) AMMI plus peer support via private social media (AMMI‐PS; *n* = 205); (3) AMMI plus strengths‐based telehealth Coaching (AMMI‐C; *n* = 196); or (4) AMMI plus peer support and coaching (AMMI‐PS‐C; *n* = 181). Intent‐to‐treat analyses used Bayesian generalized linear modelling of intervention impact over 24 months.

**Results**: Significant benefits were found on two outcomes. PrEP uptake matched youth national rates initially (11.3% current, 18.8% lifetime) and increased at 4 months to 15% across intervention arms but continued to increase in the AMMI‐PS‐C arm over time compared to other groups (OR 2.35; 95% CI: 1.27–4.39 vs. AMMI control). By 12 months, over 23% of AMMI‐PS‐C participants reported PrEP use, with some fall‐off at 20–24 months, concurrent with the COVID‐19 epidemic. About half of the youth receive no ancillary services during each assessment period. Youth receiving the AMMI condition had significantly greater use of services when recruited; however, over time, the rate of service use remained most consistent in the AAMI‐PS‐C condition.

**Conclusions**: The most intensive intervention group receiving AMMI, peer support, and coaching improved and sustained PrEP use and services utilization over time compared to other groups.

### OAC0505 Increased use of event‐driven PrEP during COVID‐19 in Australia: results from behavioural surveillance 2019–2021

C. Chan ^1^, M. Holt^2^, T.R. Broady^2^, J. MacGibbon^2^, L. Mao^2^, G. Prestage^1^, B. Wilcock^3^, B. Clifton^4^, B.R. Bavinton^1^

^1^Kirby Institute, UNSW Sydney, Sydney, Australia, ^2^Centre for Social Research in Health, UNSW, Sydney, Australia, ^3^Australian Federation of AIDS Organisations, Sydney, Australia, ^4^National Association of People with HIV Australia, Sydney, Australia

**Background**: COVID‐19 has impacted sexual behaviour and engagement with sexual health services, including HIV/STI testing and PrEP. Event‐driven PrEP (ED‐PrEP) has been included in Australian PrEP guidelines since 2019 and may be an attractive option for gay and bisexual men (GBM) who reduced their sexual activity due to COVID‐19.

**Methods**: ED‐PrEP use trends between 2019 and 2021 were assessed using national data from the Gay Community Periodic Surveys. Additional analyses using data from the last available COVID‐19‐affected rounds (2020–21) were included to compare characteristics between daily PrEP users and ED‐PrEP users.

**Results**: Between 2019 and 2021, 24,815 survey responses were included. Of these, 6754 (27.0%) reported PrEP use in the last 6 months (25.6% in 2019 to 26.9% in 2021). Among PrEP users, ED‐PrEP use increased from 7.8% in 2019 to 20.0% in 2021. There were 2077 PrEP users in the last available round in 2020–2021; 79.5% were daily PrEP users and 20.5% ED‐PrEP users. Compared to daily PrEP users, ED‐PrEP users were less likely to identify as gay (84.2% vs. 88.7%, aOR = 0.70, 95% CI = 0.50–0.97), have received an STI diagnosis in the last 12 months (29.4% vs. 38.5%, aOR = 0.74, 95% CI = 0.58–0.96) or have engaged in condomless anal sex with casual partners in the last 6 months (64.0% vs. 72.4%, aOR = 0.72, 95% CI = 0.56–0.94). ED‐PrEP users were more likely to have reduced their PrEP use due to COVID‐19 (60.8% vs. 46.5%, aOR = 1.75, 95% CI = 1.38–2.21).

**Conclusions**: ED‐PrEP use more than doubled between 2019 and 2021. ED‐PrEP users had a lower risk profile as they are less likely to engage in condomless sex or receive an STI diagnosis. ED‐PrEP users were more likely to report PrEP disruptions due to COVID‐19 and may have switched from daily to ED‐PrEP. Continued monitoring of ED‐PrEP use is recommended for tracking the impact of COVID‐19 on PrEP use and uptake of this regimen generally.

### OAD0102 Reporting, prevention and response of gender‐based violence against men who have sex with men in Taraba State, Nigeria

C. Nwagbo ^1^, E. Anene^2^

^1^University of Port Harcourt/International Centre for Total Health and Right Advocacy Empowerment (ICTHARAE), Programmes, Jalingo, Nigeria, ^2^International Centre for Total Health and Rights Advocacy Empowerment (ICTHARAE), Programmes, Jalingo, Nigeria

**Background**: Men who have sex with men (MSM) in Taraba state have HIV prevalence that is 10 to 15 times higher than the general population. They are also frequently affected by gender‐based violence (GBV). The experience of GBV has been associated with higher vulnerability to HIV among MSM. To prevent GBV and mitigate its effects on HIV outcomes and to promote wellbeing of its MSM participants, ICTHARAE has implemented GBV activities for HIV prevention, treatment and care programme in Taraba between 2019 and 2022.

**Description**: ICTHARAE's GBV reporting, prevention and care programme component promotes a dynamic of empowerment of MSM. First, we used psychosocial education and funder‐support capacity building to strengthen MSM's knowledge of their human and legal rights with an enlightenment on the constitutional and legal environment in the fight against GBV as well as de‐normalize the violence that they experience frequently. MSM who have been victims of GBV received legal and psychosocial services and are assisted to report and prosecute if they wish. ICTHARAE leaders and MSM community members (MSM‐CM) hold advocacy sessions with authorities Taraba.

**Lessons learned**: From 2019 to 2022, 189 cases of physical, sexual, social and emotional GBV were reported by key population leaders (41 cases), ICTHARAE staff (50 cases) and MSM‐CM (98 cases). Cases that have elements of physical GBV were 57, sexual GBV were 85, that of social GBV were 59 and emotional GBV were 157. Between 2019 and 2022, 69 authorities participated in advocacy sessions and 130 MSM participated in capacity building and psychoeducation/sensitization on GBV. Also, with funder support, a new strategy that empowers ICTHARAE to support organizations with $1000 to tackle GBV issues was also implemented and five organizations participated.

**Conclusions/Next steps**: Unfortunately, GBV continues to be a barrier to HIV prevention and treatment for MSM in Taraba. In 2023, ICTHARAE plans to strengthen GBV reporting network to include beneficiary of the $1000 support fund while planning to reach MSM community members in other states of Nigeria. ICTHARAE is also exploring other means to strengthen referral of GBV survivors to medical, legal, social support services and to promote the systematic documentation of violence to support national advocacy.

### OAD0103 Different forms of violence among displaced and conflict‐affected women living with HIV (WLHIV) in Ukraine

A. Wolfe ^1^, M.A. Roach^2^, G. Turpin^1^, O. Syarif^3^, P. Looze^3^, K. Lalak^3^, J. Anoubissi^3^, S. Brion^4^, K. Dunaway^4^, D. Matyushina^5^, L. Sprague^5^, C. Garcia de Leon Moreno^5^, S. Baral^1^, C. Lyons^1^, K. Rucinski^2^

^1^Johns Hopkins Bloomberg School of Public Health, Epidemiology, Baltimore, United States, ^2^Johns Hopkins Bloomberg School of Public Health, International Health, Baltimore, United States, ^3^Global Network of People living with HIV (GNP+), Amsterdam, Netherlands, ^4^The International Community of Women living with HIV (ICW), London, United Kingdom, ^5^UNAIDS, Geneva, Switzerland

**Background**: Women living with HIV (WLHIV) in conflict zones are at high risk of sexual and physical violence due to instability, stigma and proximity to military personnel. Given sustained ongoing conflict in parts of Ukraine, this study evaluated the relationship between displacement and sexual violence, healthcare‐related stigma and reproductive coercion among WLHIV in Ukraine.

**Methods**: Led by the All‐Ukrainian Network of PLHIV (100% Life), WLHIV aged 18+ were recruited from 17 regions of Ukraine and completed a socio‐behavioural questionnaire (July–August 2020). Displacement was defined as ever being a refugee, migrant worker or internally displaced person. Outcomes included sexual violence, abuse perpetrated by healthcare workers and reproductive coercion related to pregnancy, sterilization and contraception. Log binomial regression models estimated prevalence ratios (PR) and 95% confidence intervals (CI) for associations between displacement and each outcome. Models were adjusted for age, education, years knowing positive HIV status and ever being on antiretrovirals.

**Results**: A total of 820 WLHIV completed the questionnaire. Displaced WLHIV were significantly more likely to have experienced sexual violence (PR: 2.72; 95% CI 1.69–4.36) and verbal or physical abuse in healthcare settings due to their HIV status (PR: 2.40, 95% CI 1.38–4.19) compared to non‐displaced WLHIV. Displacement was also associated with contraceptive coercion (PR: 3.31, 95% CI 1.20–9.24).

**Figure:**
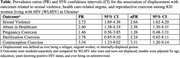
**Abstract OAD0103‐Table 1**.

**Conclusions**: Given the growing conflict in Ukraine, these data suggest that sexual violence and stigma are fundamental barriers to health and wellbeing among WLHIV in Ukraine. Training uniformed officers and clinicians at all levels to ensure understanding and accountability is likely central to mitigating these rights violations in the future, and scale‐up of trauma‐informed care for displaced WLHIV may serve to mitigate violations to date. As short‐term humanitarian health needs lessen and long‐term reproductive health needs are re‐prioritized for displaced populations living with HIV, a national focus on autonomy and client communication can also serve to mitigate rights violations.

### OAD0104 Violence among adult Venezuelan migrants: results from a multi‐site survey in Colombia

J. Guillen ^1^, A. Wirtz^2^, M. Stevenson^2^, M. Barriga^3^, D. Martinez^3^

^1^Corporacion Red Somos, Colombia, ^2^John Hopkins University, Maryland, Baltimore, United States, ^3^Corporacion Red Somos, Bogota, Colombia

**Background**: While globally prevalent, violence victimization of migrant populations is a concern due to contexts of migration and associated social and structural vulnerabilities. We estimated the prevalence and correlates of violence victimization among Venezuelan migrants in Colombia.

**Methods**: Venezuelan adults who migrated since 2015 and resided in four cities from Colombia were sampled using RDS. Socio‐behavioural surveys included measures of psychological, physical, sexual violence and sexual exploitation during their time in Colombia and within the past 12 months. Referrals were provided to participants who reported victimization. Descriptive statistics and RDS‐weighted estimation were used to calculate sample characteristics and prevalence estimates, respectively. We calculate adjusted odds ratios (aOR) of correlates of victimization using logistic regression models, stratified by gender.

**Results**: Participants (*N* = 6087) were median age of 32 years; 65% women, 34% men and 1% transgender/non‐binary‐identified. Twelve percent of participants reported any victimization while in Colombia (RDS‐weighted population prevalence: 11.7; 95% CI: 11.0–12.6), which was lower in Barranquilla/Soledad versus Bogotá/Soacha (8.3% vs. 14.9%, *p*<0.001) and higher among men (15%) than transgender/non‐binary participants (13%) and women (10%, *p*<0.001). Five percent (RDS‐weighted population prevalence: 6.0; 95% CI: 5.4–6.7) reported any violence in the past 12 months. Victimization in Colombia included psychological (7%), physical (7%), forced sex (1%) and sexual exploitation (2%). Men more commonly reported psychologic and physical violence, while women and transgender/non‐binary participants more commonly reported forced sex and sexual exploitation. Strangers were the most common perpetrator across forms of violence, followed by intimate‐partners, family members, other trusted individuals and police. Among women, violence was associated with unsafe housing: having 1–10 unsafe nights (aOR: 1.9, 95% CI: 1.4–2.7), > = 11 unsafe nights (aOR: 2.6, 95% CI: 1.7–4.0); food‐insecurity (aOR: 2.4; 95% CI: 0.9–6.7); exchange‐sex (aOR: 12.8; 95% CI: 7.5–21.8); and pregnancy while in Colombia (aOR: 1.4; 95% CI: 1.2–1.8) after controlling for site and year of migration. Among men, violence was associated with unstable housing: 1–10 unsafe nights (aOR: 1.7, 95% CI: 1.2–2.5), > = 11 unsafe nights (aOR: 2.3, 95% CI: 1.4–3.7); food insecurity (aOR: 2.0; 95% CI: 0.8–5.2), exchange sex (aOR: 3.5; 95% CI: 1.7–7.3) and same‐sex partnerships (aOR: 1.5; 95% CI: 1.0–2.2).

**Conclusions**: Reported violence victimization while in Colombia are slightly lower than anticipated and likely attributed to under‐reported intimate partner violence due to perceived low severity of violence. Findings highlight the experiences of violence among migrants that relate to structural vulnerabilities associated with housing instability, food‐insecurity, transactional sex and stigmatization of sexual orientation or transgender identity.

### OAD0105 Prevalence of intimate partner violence among adolescent girls and young women enrolled in the Determined, Resilient, Empowered, AIDS‐free, Mentored and Safe programme in Zimbabwe; evidence from Sentinel Survey, 2022

F.H. Mudzengerere ^1^, E. Tachiwenyika^1^, D. Dhakwa^1^, K. Yogo^1^, F. Mudokwani^1^, B. Nyamwanza^2^, R. Yekeye^2^, K. Muzondo^1^, B. Madzima^2^, C. Rugube^3^, T.A. Tafuma^1^, H.W. Mafaune^1^

^1^Zimbabwe Health Interventions, Harare, Zimbabwe, ^2^National AIDS Council, Harare, Zimbabwe, ^3^Family Health International, Harare, Zimbabwe

**Background**: Intimate partner violence (IPV) is pervasive globally, with 27% of women aged 15–49 years having experienced IPV in 2021, while it was 36% in sub‐Saharan Africa. IPV against women is a human rights concern with negative outcomes, including physical and mental health problems, as well as associated with acquiring HIV. IPV among adolescent girls and young women (AGYW) enrolled in the Determined, Resilient, Empowered, AIDS‐free, Mentored and Safe (DREAMS) programme has not been explored. We assessed the prevalence of IPV among the AGYW aged 9–19 years enrolled in the DREAMS programme in Zimbabwe.

**Methods**: We conducted an analytical study among AGYW aged 9–19 years enrolled in the DREAMS programme from the 1st of October 2021 to the 30th of September 2022. AGYW were randomly selected from the DREAMS programme DHIS 2 database, and data were collected across all nine Zimbabwe Health Interventions (ZHI) DREAMS districts. Data were collected using structured questionnaires with Kobo Toolbox and were analysed using STATA generating descriptive statistics and measures of association. Study received ethics approval from Medical Research Council of Zimbabwe (MRCZ/A/2933).

**Results**: Of the 1616 AGYW interviewed, 32.1% were aged 15–19 years, 3.8% were married and 13.4% were sexually active. Nearly 7% (121/1616) AGYW experienced different forms of IPV, and of these, 25% were slapped, 16% were hit by fist or threatened, 15% were pushed or shoved and 14% were forced to have sex. AGYW who were married were more likely to experience IPV than those single and widowed (COR = 3.90 [95% CI = 1.68: 9.05]). AGYW who attained lower levels of education were more likely to experience IPV than those with higher levels of education (COR = 4.71; 95% CI [4.11;5.40]). Over 62% of AGYW reported that community leaders acted against perpetrators of IPV, and this was spearheaded through community norms change intervention under the DREAMS programme.

**Conclusions**: A significant proportion of AGYW experienced IPV including slapping and forced sex. We recommend strengthening IPV reporting and service delivery through the DREAMS GBV platforms and to cascade community norms change intervention to all DREAMS districts to prevent IPV.

### OAD0202 An incognito patient approach to measure enacted HIV and gay stigma in healthcare settings

Y. Zhang ^1^, S. Sylvia^2^, S. Meng^3^, H. Xie^4^, R. Zhao^5^, A. Kwan^6^, D. Luo^3^, K. Zhang^3^, I. Fei^5^, K. Smith^5^, spr

^1^Kirby institute, UNSW Sydney, Sydney, Australia, ^2^UNC's Gillings School of Global Public Health, Charlotte, North Carolina, United States, ^3^UNC‐Project China, Guangzhou, China, ^4^University of Wisconsin‐Milwaukee, Milwaukee, United States, ^5^University of Minnesota Twin Cities, Twin Cities, United States, ^6^University of California, San Francisco, United States

**Background**: Enacted healthcare stigma has a complex and severe impact on physical and mental health of men who have sex with men (MSM). However, most studies measured enacted healthcare stigma using self‐reported measures that may have been limited by social desirability bias. This study applies standard patient (SP) approach to covertly observe provider behaviours to assess the impact of stigma against MSM in terms of HIV, sexual or its intersection on the quality of syphilis care in healthcare settings in China.

**Methods**: Trained SPs conducted unannounced visits with consenting providers. We randomly varied the HIV status and sexual orientation of each presented case to quantify stigma as differences in care across case scenarios. Care quality was assessed in four domains, including adherence to clinical guidelines, diagnostic testing, patient‐centred care and visit duration. Item response theory models were used to calculate weighted indices for continuous care scores. Fixed effect linear and logistic regressions were used to assess differences in care quality across scenarios.

**Results**: SPs conducted 123 clinic visits with 41 providers across 17 clinics. Scores for clinical guideline adherence were lower in all stigmatized scenarios as compared to the referent condition of an HIV‐negative straight man, though only the estimate for HIV‐MSM was statistically significant (β, −0.61, 95% CI, −1.18, −0.04). Appropriate diagnostic testing was less likely when SPs presented as HIV positive irrespective of sexual orientation (HIV‐positive straight: OR, 0.35, 95% CI 0.00−3.35; HIV‐positive MSM: OR 0.08, 95% CI 0.01−0.77). We did not observe differences in patient‐centred care scores. No adverse events or overtly hostile provider behaviours were reported.

**Conclusions**: Our novel incognito patient approach documented notable declines in healthcare quality among cases presenting as HIV positive, MSM or both. SPs presenting with stigmatized identities experienced less thorough clinical assessments or diagnostic testing, suggesting that stigma most often manifests in the form of less attentive or even neglectful care. Our findings provide key insights to understand how stigma in marginalized populations can impair health even in the absence of grossly negligent. Results also informed the design of a pilot stigma reduction for providers, results of which are forthcoming.

### OAD0203 Effects of a novel group‐based cognitive behavioural therapy (CBT) intervention on stigma, psychosocial wellbeing and HIV service use among sexual and gender minorities in Nigeria

J. Pulerwitz ^1^, A. Gottert^1^, W. Tun^1^, A. Fernandez^2^, P.L. Oladimeji^3^, S. Sangowawa^3^, A. Adedimeji^2^

^1^Population Council, Washington DC, United States, ^2^Population Council, Abuja, Nigeria, ^3^Consultant, Abuja, Nigeria

**Background**: High levels of stigma due to identifying as a sexual or gender minority (SGM) as well as living with HIV (i.e. intersectional stigma) are increasingly documented in the African setting, and often manifest as self‐stigma (also called internalized stigma). Such stigmas impede psychosocial wellbeing as well as HIV prevention/care, and there are few (if any) evidence‐based internalized stigma reduction interventions in this context. We developed and evaluated a novel, group‐based CBT stigma intervention for men who have sex with men (MSM) and transgender women (TGW) at risk for/living with HIV in Lagos, Nigeria.

**Methods**: The intervention, adapted from a Canadian curriculum, comprised four weekly in‐person sessions facilitated by community health workers. We conducted a delayed intervention group randomized controlled trial, with pre‐post surveys plus 3‐month follow‐up, as well as qualitative interviews with participants/programme staff. Outcomes included internalized stigma related to SGM and HIV status, depression, resiliency and PrEP/HIV treatment use.

**Results**: Mean age of the 240 participants was 26 years (range 18–42). Seventy‐seven percent were MSM and 23% TGW; 27% were living with HIV. Most (88%) participants attended all four sessions, and 98% expressed high intervention satisfaction. There was significant improvement in each psychosocial outcome between baseline and second surveys, in both the immediate (post‐intervention) and delayed (pre‐intervention) arms. Qualitative data obtained from participants post intervention described enhanced self‐confidence, resilience when facing stigma and coping skills, and indicated that positive changes found in the delayed group (pre‐intervention) were mainly due to perceived support from the interviewers/survey experience. There were further positive changes from baseline to 3‐month follow‐up in, for example intersectional internalized stigma and depression, for the immediate intervention group. Controlling for baseline levels of ever PrEP use, 75% of immediate‐group participants reported currently using PrEP at 3 months post‐intervention versus 53% of delayed‐group participants right after the intervention (*p*<0.01).

**Conclusions**: This study demonstrated feasibility and acceptability of a group‐based CBT model in Nigeria. There were also indications of preliminary efficacy related to mental health outcomes and PrEP, despite the randomized design not holding up (where study participation/contact became an intervention in itself).

### OAD0204 Political conservatism and social distancing from people living with HIV among medical students: mediating roles of negative stereotypes and negative intergroup emotions

S.A. Nemli ^1^, Y. Bayramoglu^2^, B. Turan^3^

^1^Izmir Katip Celebi University, Infectious Diseases and Clinical Microbiology, Izmir, Turkey, ^2^The University of Alabama at Birmingham, Psychology, Birmingham, United States, ^3^Koc University, Psychology, Istanbul, Turkey

**Background**: HIV‐related stigma within the healthcare system is a major barrier preventing people living with HIV (PLWH) from accessing and continuing treatment. Psychosocial factors, such as political orientation, personality characteristics and personal moral values of healthcare providers, have not been adequately investigated. Furthermore, a deeper understanding of the mechanisms in the effects of these drivers on social distancing from PLWH is needed, especially among medical students (future healthcare providers). The present study aims to fill these gaps by studying stigmatizing attitudes of medical students from the perspective of the inevitability of prejudice due to negative intergroup emotions.

**Methods**: Participants were 609 medical students (326 women, 53.5%) attending a medical school in Izmir, Turkey. Demographic features, political orientation, endorsing stereotypes about PLWH, stigmatizing attitudes, emotional reactions towards PLWH, social distance from PLWH and HIV knowledge were assessed via self‐reported questionnaires between March 2021 and June 2021. Multiple regression analyses and a serial mediation analysis calculating indirect effects using bootstrapping were used by adjusting for demographic factors and HIV knowledge.

**Results**: Political conservatism, endorsing negative stereotypes about PLWH and negative intergroup emotions towards PLWH were all significantly associated with attitudes towards social distancing from PLWH. A serial mediation first by endorsing negative stereotypes about PLWH and then by negative intergroup emotions towards PLWH in the association between higher political conservatism and higher social distancing from PLWH was found, supporting our hypothesis.

**Conclusions**: Findings suggest that interventions may target stereotyping and negative intergroup emotions to reduce discriminatory behaviours of medical students. Furthermore, to reduce HIV‐related stigma and discrimination and to improve healthcare delivery to PLWH, more research is needed on the roles of stigma within the healthcare system specifically, and on governmental policies and societal factors that contribute to structural stigma in general.

### OAD0205 Gender identity stigma and discrimination and condomless anal sex among transgender women of TransCITAR study from Buenos Aires, Argentina: mediating role of depressive symptoms

R. Caballero^1,2^, E. Frontini^3^, V. Zalazar^3^, N. Cardozo^3,4,5^, M. Duarte^3,5^, S. Fabian^3,6^, F. Vissicchio^3^, S. Cahn^3^, P. Radusky^3,2^, C. Frola^1,7^, M.I. Figueroa^3^, I. Aristegui ^3^

^1^Fundación Huésped, Research Department, Buenos Aires, Argentina, ^2^Universidad de Buenos Aires, Faculty of Psychology, Buenos Aires, Argentina, ^3^Fundación Huésped, Research Institute, Buenos Aires, Argentina, ^4^REDLACTRANS, Buenos Aires, Argentina, ^5^Asociación de Travestis, Transexuales y Transgéneros de Argentina (A.T.T.T.A.), Buenos Aires, Argentina, ^6^Asociación Civil Gondolin, Buenos Aires, Argentina, ^7^Hospital Juan A. Fernández, Infectious Diseases Unit, Buenos Aires, Argentina

**Background**: Co‐occurrence of stigma, depression and condomless anal sex (CAS) among transgender women (TGW) is widely reported. However, interactions between these factors remain unclear. TransCITAR is a prospective cohort study of 500 trans and non‐binary individuals from Buenos Aires, Argentina; initiated in September 2019. This sub‐analysis explored the mediating role of depressive symptoms in the relation between gender identity stigma, gender identity discrimination and CAS in TGW participating in TransCITAR.

**Methods**: Until December/2022, 421 TGW, recruited by peer navigators, completed baseline psychosocial interviews that included questionnaires designed ad hoc to collect information on socio‐demographic variables, gender identity discrimination (in healthcare, police, etc.) in lifetime, and CAS (receptive and insertive) in the last month, a gender identity stigma (GIS) scale and the CES‐D (depressive symptoms). Path analysis was performed to examine the relationships between these variables. Parameters were estimated using maximum likelihood (ML) estimator. Model fit was assessed through several goodness of fit indices, including comparative fit index (CFI), Tucker–Lewis index (TLI) and root mean square error of approximation (RMSEA).

**Results**: Median age was 30 years (IQR 25–37), 50% reported incomplete high school or lower, 41% unstable housing, 45% receiving financial aid, 31% being migrant and 53% current engagement in sex work. The prevalence of CAS was 26% (*n* = 109) and HIV laboratory‐confirmed basal prevalence was 42%. The mediation model showed an excellent goodness of fit (CFI = 1.000; TLI = 1.000; RMSEA = 0.000). The indirect effect of GIS to CAI through depressive symptoms was significant (estimate = 0.057, *p* = 0.000), while the indirect effect of GIS experience of discrimination to CAS was not mediated by depressive symptoms (estimate = 0.138, *p* = 0.38). GIS was positively associated with depressive symptoms (Std. estimate = 0.356; *p*<0.001), which were positively associated with CAS (Std. estimate = 161, *p* = 0.000).

**Conclusions**: Depressive symptoms played a mediating role in the association between gender identity stigma and CAS. HIV and STIs preventive interventions for TGW should incorporate elements of trauma‐informed and skills‐building care. Moreover, it is crucial for reducing CAS to advocate for social inclusion programmes, trans‐inclusive environments and anti‐discriminatory policies that reduce stigma and its health‐related effects.

### OAD0302 “We want them to know that we exist”: how can we better meet the needs of sexuality and gender diverse young people living with HIV? Qualitative research from Zimbabwe

J. Lariat ^1^, W. Mavhu^2,3^, T. Mudhuma^4^, P. Shaba^2^, S. Sibanda^2^, R. Mbundure^2^, C. Wogrin^4^, A. Mutsinze^4^, N. Willis^4^, S. Bernays^1,5^

^1^University of Sydney, School of Public Health, Sydney, Australia, ^2^CeSHHAR, Harare, Zimbabwe, ^3^Liverpool School of Tropical Medicine, Liverpool, United Kingdom, ^4^Zvandiri, Harare, Zimbabwe, ^5^London School of Hygiene and Tropical Medicine, Department of Global Health and Development, London, United Kingdom

**Background**: There is a scarcity of research about the experiences, challenges and needs of sexuality and gender diverse (SGD) young people living with HIV. Failing to recognize, understand and respond to this group's needs perpetuates long‐standing inequities in the HIV response and restrains progress towards key targets. We present qualitative findings on experiences and challenges of SGD young people living with HIV in Zimbabwe.

**Methods**: We conducted two focus group discussions in 2022 with 14 self‐identified SGD young people (18–24 years) all of whom were accessing a recently formed SGD HIV support group at Zvandiri (“As I Am”), a community‐based HIV programme. We conducted interpretive thematic analysis to generate themes across the data.

**Results**: All the young people refuted the imposition of static binary categories of sexuality and gender. They understood their identities as fluid and still in the process of becoming. All participants described that being an SGD young person living with HIV had led to “double stigma and double trouble.” This manifested in physical and verbal harassment, social exclusion and family rejection. In most situations, they had to keep both their sexuality and/or gender identity and HIV status hidden, but many also felt compelled to conceal their HIV status in SGD social spaces. This negatively impacted their psychosocial wellbeing and social connectedness.

Participants shared positive experiences of Zvandiri, describing the service as “speaking to me.” The mutual witnessing of others’ experiences of living with HIV, in a safe and destigmatizing environment, enhanced self‐acceptance and improved motivation to maintain engagement in treatment. However, reflecting their prevailing experiences, participants were cautious about being open about their gender and sexuality outside of their SGD group even within Zvandiri.

**Conclusions**: Understanding how intersectional stigma impacts SGD young peoples’ social and relational lives, and their access to healthcare services, is a critical step towards appropriately responding to their needs. Community‐based HIV support services which emphasize and promote principles of inclusivity are well‐positioned to support and advance SGD young peoples’ health rights. Efforts to strengthen understanding and responsiveness to this group's needs should be prioritized as a mechanism to improve their wellbeing and HIV outcomes.

### OAD0303 “It felt like a weight was being taken off my shoulders”: the impact of lending a hand intervention in supporting migrant adolescents and young people, in KwaZulu‐Natal, South Africa

N. Dlamini ^1^, J. Seeley^2^, M. Shahmanesh^3^, N. Ngwenya^1^, S. Hlongwane^1^, C. Herbst^1^

^1^Africa Health Research Institute, Social Science Core, Mtubatuba, South Africa, ^2^London School of Hygiene and Tropical Medicine, Global Health and Development, London, United Kingdom, ^3^University College London, London, United Kingdom

**Background**: In South Africa, many young people move away from their homes to semi‐urban areas for education. They attend day schools while staying in rented accommodation. They may experience alcohol and drug abuse, sexual exploitation and violence all of which put them at risk of contracting HIV. Our aim was to develop and test the feasibility and acceptability of a support structure for migrant adolescents and young people (MAYP), aged 14–24, and to understand their experiences of the intervention, in KwaZulu‐Natal, South Africa.

**Methods**: Five peer navigators (PNs) were trained on needs assessment (clinical, social, educational and psychosocial) of MAYP. The PNs enrolled 283 MAYP, aged 14–24, between June 2021 and October 2022. The intervention included: (1) using a mobile phone to provide support identified during the needs assessment; (2) facilitating the referral process at a call centre to the study social worker for psychosocial and emotional services, local health facilities for healthcare services and peer support on general issues. We conducted repeat in‐depth interviews (IDIs), *n* = 20; and five key informant IDIs with PNs, *n* = 5, both face‐to‐face and telephonically.

**Results**: The majority of MAYP were in grades 11 and 12 at day schools. They rented rooms in an unsafe environment on their own to be closer to their schools. Transitioning from living with their parents or guardians to living on their own in an unfamiliar environment caused physical (e.g. violence), psychological and mental challenges. In describing the intervention, most of them “felt like a weight was being taken off of their shoulders.” They also felt that it was beneficial for them as they were linked to treatment for sexually transmitted infections, received psychosocial support from the intervention social worker and were also supported by PNs in facing general challenges. This helped them develop adaptive coping strategies and avoid risky behaviours.

**Conclusions**: Interventions targeted for young people are much more effective when they are led by peers who understand young people's experiences. The intervention was designed for and centred around the needs of MAYP and the short turnaround time during the referral process made it acceptable and they felt respected.

### OAD0304 “If I don't take care of me, then I can't be there for others”: a qualitative study of caregiving relationships among older women living with HIV

T. Vu ^1^, M. Quinn^1^, J. Monin^1^

^1^Yale University School of Public Health, Social and Behavioral Sciences, New Haven, United States

**Background**: There are over 100,000 women ages 50+ diagnosed with HIV in the United States. Yet, the experiences of older women living with HIV (WLWH) remain understudied. Little is known about the social support networks that older WLWH use to manage their health. This study examines the social support, or care networks, of older WLWH, with an emphasis on their caregiving and care receiving relationships.

**Methods**: We recruited women ages 50+ who are living with HIV from community‐based organizations and clinics in the United States. We conducted one‐on‐one, semi‐structured phone interviews between May 2022 and August 2022. Interviews were recorded and transcribed verbatim. Following a Grounded Theory approach, two coders performed open coding and then thematic coding to generate themes.

**Results**: Participants (*N* = 23) came from 11 U.S. states, were on average 60.3 years old (min 51, max 68) and had been living with HIV for an average of 23.7 years. 78.3% of participants were Black. 78.3% identified as heterosexual. We identified five main themes regarding care networks and perceptions of caregiving in older adulthood: (1) Participants received the most care (i.e. instrumental/emotional support to help manage HIV) from their adult children and HIV support group peers. Participants provided the most care to their grandchildren and own parents. (2) Despite occasional periods of stress balancing caregiving responsibilities while managing HIV, participants have pride and joy in being caregivers to loved ones. (3) Caregiving and receiving networks not only help disease management, but also promote self‐love and acceptance. (4) Despite receiving care, participants are highly proactive in their own HIV management. (5) Many had concerns about being able to keep up with their HIV care needs due to uncertainty about who will be in their care networks in the future and comorbidity.

**Conclusions**: Findings highlight that both being a care recipient and a caregiver can be sources of meaning for older WLWH to help in their HIV management. To address the concerns brought forth by our participants about ageing with HIV, public health programmes and policies for older WLWH may benefit from engaging their care networks in healthcare discussions and educational efforts.

### OAD0305 Assessing the health‐related quality of life and risk of poverty of older people living with HIV in Spain: a cross‐sectional study applying a gender perspective

N. Nuño^1^, A. Martínez^1^, S. Martínez^1^, M. Cobos ^1^, J.S. Hernández^2^, R. Polo^1^

^1^Spanish Ministry of Health, Madrid, Spain, ^2^Working Group on HIV Treatments (gTt‐HIV), Barcelona, Spain

**Background**: Modern antiretroviral therapies have increased the life expectancy of people living with HIV. However, the health‐related quality of life and living conditions of older people living with HIV in Spain have yet to be studied.

**Methods**: We implemented a self‐administered online questionnaire in 2022 to assess the health‐related quality of life and risk of poverty of Spanish older people living with HIV (≥50 years) and identify gender differences. We applied the standardized WHOQoL‐HIV BREF questionnaire to estimate health‐related quality of life and the Europe 2020 guidelines to calculate the risk of poverty. The statistical analysis included multivariate generalized linear models with potential confounding variables and robust estimates.

**Results**: A total of 242 older people living with HIV (187 men and 55 women) participated in the study. The average age of men and women was 57.1 (standard deviation [SD] = 5.1) and 56.3 (SD = 4.0) years, respectively. Women scored lower (compared to men) in 84% of the WHOQoL‐HIV BREF questionnaire items. In consequence, women had significantly lower health‐related quality of life in five (out of six) of the questionnaire domains: physical health (β: −1.5; 95% confidence interval [CI]: −2.5, −0.5; *p*: 0.003), psychological health (β: −1.0; 95% CI: −2.0, −0.1; *p*: 0.029), level of independence (β: −1.1; 95% CI: −2.0, −0.2; *p*: 0.016), environmental health (β: −1.0; 95% CI: −1.8, −0.3; *p*: 0.007) and spirituality/personal beliefs (β: −1.4; 95% CI: −2.5, −0.3; *p*: 0.013). The risk of poverty was substantial for both men (30%) and women (55%), but women were significantly more likely to be at risk of poverty (odds ratio: 3.3; 95% CI: 1.5, 7.7; *p*: 0.004).

**Conclusions**: Our study supports the need for policies focused on improving the structural and living conditions and comprehensive care of older people living with HIV and reducing gender inequalities in health‐related quality of life. Future policies and interventions for older people living with HIV in Spain should prioritize the improvement of the structural and living conditions in which they subsist.

### OAD0402 Multilevel barriers to methadone for HIV prevention among people who inject drugs in Kazakhstan: opportunities for change

A.R. Liberman ^1^, E. Rozental^2^, D.J. Bromberg^3^, R. Ivasiy^1^, A.Z. Kussainova^4^, S. Primbetova^2^, L.M. Madden^1,5^, A. Terlikbayeva^2^, F.L. Altice^5,1,3,6^

^1^Yale School of Medicine, Section of Infectious Diseases, New Haven, United States, ^2^Global Health Research Center of Central Asia, Almaty, Kazakhstan, ^3^Yale School of Public Health, Department of Social and Behavioral Sciences, New Haven, United States, ^4^Kazakhstan National Medical University, Almaty, Kazakhstan, ^5^APT Foundation, New Haven, United States, ^6^Yale University, Center for Interdisciplinary Research on AIDS, New Haven, United States

**Background**: Central Asia (EECA) remains one of few regions where HIV incidence and mortality continue to increase, and this epidemic is concentrated among people who inject drugs (PWID). In Kazakhstan, the prevalence of HIV among PWID (9.2%) is higher than for any other key population. Opioid agonist therapies (OAT) like methadone or buprenorphine are evidence‐based treatment for opioid use disorder (OUD) and crucial primary and secondary HIV prevention. Though methadone has been provided for free in Kazakhstan since 2008 through support from international donors, there are currently only approximately 340 (<1%) people on OAT among the estimated 90,000 PWID.

**Methods**: To assess barriers and facilitators to methadone uptake for HIV prevention, we conducted nominal group technique (NGT) focus groups (FGs) with people with OUD in four cities in Kazakhstan. Among the eight FGs, four included people currently on methadone, while the other four included people who had never received methadone. Additionally, we conducted two focus groups with local doctors and in‐depth interviews with the directors at the four OAT sites and with several political figures who shape methadone policy in Kazakhstan.

**Results**: Multi‐level barriers included: policy (e.g. required national registration as a “drug user” to access addiction treatment services); structural (e.g. inaccessible locations of clinics, rigid enrolment requirements); clinician (e.g. viewing potential methadone programme participants as undisciplined, and therefore, not ready for treatment); and patient (e.g. too many logistical requirements). A detailed rank‐ordered list of barriers and facilitators will be expanded below.

**Conclusions**: Findings from this study identify many opportunities for potential methadone scale‐up, which is required to control the HIV epidemic in Kazakhstan and throughout Central Asia.

### OAD0403 Dapivirine vaginal ring (DPV‐R): an acceptable and feasible HIV prevention option. Evidence from Zimbabwe​

M. Munjoma ^1^, J. Mavudze^1^, T. Moga^1^, I. Moyo^1^, N. Shoko^1^, N. Nhando^1^, T. Sola^2^, J. Majongosi^1^, M. Mutseta^2^, G. Ncube^2^, B. Mutede^1^, N. Taruberekera^1^, S. Leuschner^3^

^1^Population Solutions for Health, Harare, Zimbabwe, ^2^Ministry of Health and Child Care, Harare, Zimbabwe, ^3^Population Services International, Harare, Zimbabwe

**Background**: HIV burden remains high in Zimbabwe. Adolescent girls and young women (AGYW) are disproportionately affected with an HIV incidence of 0.54% compared to 0.13% among their male counterparts (ZIMPHIA, 2020). While oral PrEP remains a key HIV prevention, modality, pill burden and privacy are key barriers to oral PrEP uptake and continuation. Population Solutions for Health, PSI, and the Ministry of Health and Child Care are implementing a demonstration project to determine the acceptability and feasibility of using monthly DPV‐R as an alternative to oral daily PrEP for HIV prevention.

**Methods**: A two‐arm prospective cohort design is being implemented across eight districts in Zimbabwe. AGYW aged 18–25, screened as high‐risk and eligible for PrEP chose between oral PrEP and DPV‐R. Clients from both arms were followed up monthly between June and November 2022. Uptake and continuation rates were compared between the two arms for significant differences. Key informant interviews were conducted with clinicians involved in PrEP service provision.

**Figure:**
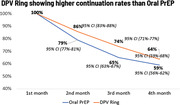
**Abstract OAD0403‐Figure 1**.

**Results**: A total of 1535 AGYW were screened for PrEP, 1466 were eligible and of these, 1128 (76.9%) (95% CIs: 74.7–79.7) chose DPV‐R. Uptake was similar by age but differed significantly by residence, with higher uptake observed in rural (97.5%—95% CI: 96.0–98.6) relative to urban (61.0%—95% CI: 57.6.0–64.3) districts. Continuation rates were consistently higher among clients on DPV‐R compared to oral PrEP as shown below. Five of 1128 high‐risk AGYW (0.4%) tested HIV positive since commencement on DPV‐R compared to 1/338 (0.3%) receiving oral PrEP over the same period. Service providers reported high motivation for DPV‐R among AGYW for its convenience and discretion.

**Conclusions**: DPV‐R is a feasible and preferable PrEP option for AGYW in Zimbabwe and should be scaled up. More demand‐generation activities are required in urban settings for improved uptake.

### OAD0404 “PrEP4U”: how edutainment, student outreach and multisector engagement are helping youth in Vietnam access HIV prevention and sexual healthcare

T.T. Tran ^1^, T.M. Le^2^, T.L.V. Tran^2^, T.K. Cao^3^, M.C.H. Vu^4^, K.E. Green^1^, D. Nguyen^5^, H.T. Nguyen^1^, P.H. Vu^1^, T.M. Phan^1^, T. Ngo^5^, Z. Humeau^1^

^1^PATH, Hanoi, Vietnam, ^2^Glink Social Enterprise, Ho Chi Minh City, Vietnam, ^3^Vietnam Administration for HIV/AIDS Control, Vietnam Ministry of Health, Hanoi, Vietnam, ^4^Vietnam Network of Transgender People, Hanoi, Vietnam, ^5^USAID/Vietnam, Hanoi, Vietnam

**Background**: In Vietnam, students and youth have several risk factors for HIV acquisition and transmission including poor HIV risk perception and awareness and limited knowledge on sexual and reproductive health (SRH) services, such as HIV testing and pre‐exposure prophylaxis (PrEP). “PrEP4U” is a behaviour change campaign co‐created by the USAID/PATH STEPS Project (STEPS) and partners to promote HIV and sexual healthcare engagement and practices among students in Vietnam.

**Description**: To enhance student/youth knowledge about SRH and safe sex and encourage their use of PrEP and other health services, STEPS, the Vietnam Ministry of Health and youth leaders generated student insights in designing the online‐to‐offline PrEP4U campaign. PrEP4U targets educational settings in three urban provinces (Hanoi, Ho Chi Minh City and Dong Nai) through talk shows with clinical experts, interactive edutainment games focused on safe sex and sexual health, integration with sex‐ed programmes at schools, booth exhibitions and other activities where students can interact with community influencers and staff from community‐based clinics and receive HIV testing, PrEP counselling and referral for other services directly on site. The campaign also runs across online platforms and leverages a network of PrEP4U Ambassadors and a PrEP4U Facebook page blending informative and humorous content derived by youth to motivate viewers to seek PrEP/SRH information and services.

**Lessons learned**: From March to September 2022, 32 in‐person PrEP4U events reached more than 8500 students, distributed 1096 HIV self‐test kits and enrolled 317 individuals on PrEP. The PrEP4U Facebook page has become a hub of trustworthy SRH and PrEP information for students, supporting the campaign to garner over 1.1 million views cross‐platform. Targeted edutainment activities centred around principles of choice, equity and people‐centredness to ensure that PrEP4U messaging and imaging resonated with different youth segments, including gay, transgender and gender non‐binary individuals.

**Conclusions/Next steps**: PrEP4U addressed a major gap in youth access to HIV and SRH services by offering these services directly within schools and engaging campaign ambassadors and influencers, and as a result increased healthcare access and convenience. Drawing from lessons learned, youth‐focused PrEP/SRH campaigns will be further scaled and adapted for other settings to bring PrEP and sex‐ed closer to populations in need.

### OAD0405 Racial inequalities in HIV testing among adolescent men who have sex with men and transgender women in three Brazilian capitals

M. França ^1^, I. Dourado^2^, A. Grangeiro^3^, L. Magno^1,2^

^1^Bahia State University, Health Science, Salvador, Brazil, ^2^Institute of Collective Health, Bahia Federal University, Salvador, Brazil, ^3^São Paulo University, Medicine School, São Paulo, Brazil

**Background**: Black people have a higher prevalence of HIV infection in several countries worldwide, including Brazil. Studies have also highlighted barriers to HIV testing in these populations because of structural racism. We aimed to investigate the association between HIV testing in a lifetime and race among adolescent men who have sex with men (aMSM) and transgender women (aTGW) in three Brazilian state capitals.

**Methods**: PrEP1519 is a prospective, multicentre, open‐label PrEP demonstration cohort study of aYMSM and aYTGW aged 15–19 in three Brazilian capitals: Salvador, São Paulo and Belo Horizonte. The outcome variable was having been tested for HIV in a lifetime (no, yes). Race was self‐reported in three categories: White, Black and mixed race (or Brown; in Portuguese: “pardo”). In Brazil, the last one is historically combined with Black in the analyses. We compared Black and mixed race combined versus White. We conducted descriptive, bivariate and multivariate analyses to estimate the adjusted odds ratio (aOR) and 95% confidence interval (95% CI).

**Results**: White adolescents underwent more HIV tests in their lifetime than Black and mixed race combined (74.0% vs. 54.0%, respectively). The testing rates decreased as the skin tone darkened: 56.9% among exclusive Blacks, 58.1% among exclusive mixed‐race (or brown) and 63.9% among Whites (*p* = 0.003). In the multivariate analysis, Black and mixed‐race combined people were 33% less likely to have been tested for HIV in a lifetime than Whites (aOR = 0.67, 95% CI: 0.51–0.90), adjusting for age, education, employment, living with family, mother's lack of knowledge about their sexual orientation, sex work and unprotected anal sex.

**Conclusions**: The lower rate of HIV testing among Black and mixed‐race combined among aMSM and aTGW people indicates inequalities in health and structural barriers to accessing HIV testing and prevention services. Further, it evidences the structural racism in society and health institutions in Brazil.

### OAD0502 Pill count effectiveness in detecting non‐adherence in a mature HIV programme with overall high viral suppression rates in Eswatini

L. Mamba ^1^, V. Williams^1^, A. Mafukidze^1^, P. Bongomin^1^, P. Dlamini^1^, G. Mchunu^1^, S. Mhlanga^1^, H. Byarugaba^1^, J. Opoku^2^, S. Kibwana^3^, S. Ojoo^2^, D. Bazira^2^, S. Haumba^1^

^1^Center for Global Health Practice and Impact, Georgetown University, Mbabane, Eswatini, ^2^Center for Global Health Practice and Impact, Georgetown University Medical Center, Washington DC, United States, ^3^Center for Global Health Practice and Impact, Georgetown University Medical Center, Washington, United States

**Background**: Pill count is widely used as an adherence measure among clients taking antiretroviral therapy (ART). Conducted before each refill, it is a self‐reported process where the actual number of pills remaining is compared against the expected. Pill count is a standard of practice to identify non‐adherence for early intervention, yet its effectiveness is unknown in Eswatini. This study examines the ability of pill count to detect non‐adherence among ART clients in the Lubombo and Manzini regions in Eswatini.

**Methods**: We used a case−control study design with chart abstraction at 11 purposively selected health facilities in Lubombo and Manzini, based on the number of clients with a high viral load between October 2021 and September 2022. Controls were matched to cases at a ratio of 2:1 giving a total of 615 study participants (205 cases and 410 controls). Cases were adult first‐line ART clients with unsuppressed viral load (VL > 1000 copies/ml), while controls were clients with a suppressed viral load (≤ 1000 copies/ml) randomly selected from the government client management information system. Adherence of 95%–105% was defined as good, and social determinants and mental health evaluated to assess adherence.

**Results**: All cases and controls had a pill count done. Overall, 60% of all participants with a suppressed viral load had a good pill count classification, while 98% of clients with a high viral load had a suboptimal pill count (<95% or >105%). Forty six percent and 99.5% of the cases and controls had a good pill count classification, respectively. Eighty percent of the poor adherence was caused by social and cognitive problems. Clients with good pill count classification had significantly reduced odds of a high viral load (OR 0.007; 95% CI: 0.003, 0.04; *p*<0.001). Pill count had a sensitivity of 98% (CI: 92%–100%); a specificity of 59% (CI: 53%–64%); a positive predictive value of 30% (CI: 24%–37%) and a negative predictive value of 99.5% (CI: 97%–100%).

**Conclusions**: Pill count predicted non‐adherence among cases, but its specificity should be improved by using a modified approach. This is vital considering the changing landscape in HIV care and increased access to more tolerable antiretroviral medication for prevention and treatment.

### OAD0503 Identifying longitudinal patterns of HIV treatment (dis)engagement and re‐engagement from oral histories of virologically unsuppressed adults in Uganda: a thematic trajectory analysis

J.G. Rosen ^1^, N. Nakyanjo^2^, W. Ddaaki^2^, T. Zhao^1^, A.V. Vo^1^, R. Nakubulwa^2^, C. Ssekyewa^2^, D. Isabirye^2^, R.L. Katono^2^, P. Nabakka^2^, R.J. Ssemwanga^2^, G. Kigozi^2^, S. Odiya^2^, G. Nakigozi^2^, F. Nalugoda^2^, G. Kigozi^2^, J. Kagaayi^2^, M.K. Grabowski^2,3^, C.E. Kennedy^1,2^

^1^Johns Hopkins Bloomberg School of Public Health, Department of International Health, Baltimore, United States, ^2^Rakai Health Sciences Program, Entebbe, Uganda, ^3^Johns Hopkins School of Medicine, Division of Pathology and Transfusion Medicine, Baltimore, United States

**Background**: There is limited study of persons deemed “harder to reach” by HIV treatment services, including those discontinuing or never initiating antiretroviral therapy (ART). We conducted narrative research in Rakai, Uganda, with virologically unsuppressed adults identified through population‐based sampling to discern longitudinal patterns in HIV service engagement and identify factors shaping treatment engagement throughout the life course.

**Methods**: In mid‐2022, we sampled adult participants with high‐level viremia (≥1000 RNA copies/ml) from the Rakai Community Cohort Study, a population‐based HIV surveillance cohort. Using life history calendars, we conducted initial and follow‐up in‐depth interviews to elicit oral histories of participants’ journeys in HIV care, from diagnosis to the present. We then used thematic trajectory analysis to identify discrete archetypes of HIV treatment engagement by “re‐storying” participant narratives and visualizing HIV treatment timelines derived from interviews and abstracted clinical records.

**Results**: Overall, 38 participants (median age: 34 years, 68% men) completed 75 interviews. We identified six HIV care engagement archetypes from narrative timelines (Figure): (1) delayed ART initiation; (2) early treatment discontinuation; (3) treatment cycling; (4) prolonged treatment interruption; (5) transfer‐related care disruption; and (6) episodic viremia. Patterns of service (dis)engagement were highly gendered, occurred in the presence and absence of optimal ART adherence, and were shaped by various factors emerging at different time points, including: denial of HIV serostatus and disclosure concerns; worsening HIV‐related symptoms; psychological distress and depression; social support; intimate partner violence; ART side effects; accessibility constraints during periods of mobility; incarceration; and inflexible ART dispensing regulations.

**Figure:**
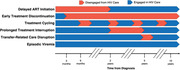
**Abstract OAD0503‐Figure 1**.

**Conclusions**: Identified trajectories uncovered heterogeneities in both the timing and drivers of ART (re‐)initiation and (dis)continuity, demonstrating the distinct characteristics and needs of people with distinct longitudinal patterns of HIV treatment engagement. Enhanced mental health service provision, expanded eligibility for differentiated service delivery models and streamlined facility switching processes may facilitate timely (re‐)engagement in HIV services.

### OAD0504 Characterizing TB diagnosis and the associations with economic stability and employment discrimination among women living with HIV across 11 countries in sub‐Saharan Africa during the COVID‐19 pandemic

C. Lyons ^1^, G. Turpin^1^, K. Rusinski^2^, O. Syarif^3^, P. Looze^3^, K. Lalak^3^, J.d.D. Anoubissi^4^, S. Brion^5^, K. Dunaway^5^, L. Sprague^6^, C.d.L. Moreno^6^, T. Mwareka^7^, K.A. Hlomewoo^8^, K.A. Dokla^8^, E. Ayeh^9^, N.A.J.C. Vako^10^, N. Otwoma^11^, A. Ouedraogo^12^, C. Beyrer^13,1^, B. Genberg^1^, S. Baral^1^

^1^Johns Hopkins School of Public Health, Epidemiology, Baltimore, United States, ^2^Johns Hopkins School of Public Health, International Health, Baltimore, United States, ^3^Global Network of People Living with HIV (GNP+), Amsterdam, Netherlands, ^4^Global network of People living with HIV (GNP+), Amsterdam, Netherlands, ^5^The International Community of women living with HIV (ICW), London, United Kingdom, ^6^UNAIDS, Geneva, Switzerland, ^7^Zimbabwe National Network of People Living with HIV (ZNNP+), Harare, Zimbabwe, ^8^RAS+, Lome, Togo, ^9^Ghana Network of Persons Living with HIV, Accra, Ghana, ^10^Ivorian Network of People Living with HIV/AIDS (RIP+), Abidjan, Cote D'Ivoire, ^11^NEPHAK, Nairobi, Kenya, ^12^REGIPIV‐BF, Ouagadougou, Burkina Faso, ^13^Duke Global Health Institute, Durham, United States

**Figure:**
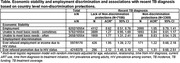
**Abstract OAD0504‐Table 1**.

**Background**: Among people living with HIV, tuberculosis (TB) is the leading cause of death and TB‐related mortality increased during the COVID‐19 pandemic. While poverty is an established determinant for TB progression, characterizing the role of employment discrimination and legal protections on HIV and TB outcomes can inform both programmes and policy.

**Methods**: The People Living with Stigma Index 2.0 study was implemented in 11 countries across sub‐Saharan Africa, including Angola, Benin, Burkina Faso, Cote D'Ivoire, Ghana, Kenya, Mauritania, Nigeria, Lesotho, Togo and Zimbabwe. Study implementation was led by networks of people living with HIV in each country between 2020 and 2022. Interviewer‐administered questionnaires were used to collect self‐reported socio‐behavioural measures. The analytic sample included 10,555 cisgender adult women living with HIV. Multilevel logistic regression was used to assess associations between exposures and recent TB diagnoses in the context of varying discrimination protections for PLHIV.

**Results**: Among participants, 7.8% reported TB diagnosis within the last 12 months. Among individuals in countries without non‐discrimination protections, recent TB diagnosis was negatively associated with employment (AOR: 0.62; 95% CI: 0.51, 0.76). Among individuals in countries without non‐discrimination protections, recent TB diagnosis was positively associated with being unable to meet basic needs often (AOR: 1.77; 95% CI: 1.31, 2.41), being refused employment and income due to HIV status (AOR: 1.95; 95% CI: 1.39, 2.72) and being refused a promotion due to HIV status (AOR: 2.12; 95% CI: 1.45, 3.10). Among individuals in countries with non‐discrimination protections, recent TB diagnosis was not associated with employment; being unable to meet basic needs often; being refused employment, income or a promotion due to HIV status.

**Conclusions**: Employment discrimination may impede HIV and TB outcomes among women living with HIV, and may be a determinant of TB regardless of ART use. Establishment and enforcement of non‐discrimination protections for women living with HIV may improve economic stability, support TB control and reduce deaths among people living with HIV.

### OAD0505 Improving mental wellbeing and economic empowerment of PLHIV through ODH‐SEGT: an integrated and client‐centred psycho‐socio‐economic intervention for unemployed PLHIV experiencing homelessness in Caloocan City, Philippines

R.A. Olete ^1,2,3^, J. Cadelina^1,4^, E. Arriola^1^, C. Chu^1,5^, J.M.P. Oyco^3^, A.L. Macalalag^6^, N.Y. Ko^2^, C. Strong^2^

^1^Gabay sa Pulang Laso Inc., Caloocan City, Philippines, ^2^National Cheng Kung University, Department of Public Health, Tainan City, Taiwan, Republic of China, ^3^Iloilo Doctors' College, College of Nursing, Iloilo City, Philippines, ^4^De La Salle University, Department of Behavioral Science, Metro Manila, Philippines, ^5^Xavier University—Ateneo de Cagayan University, Department of Psychology, Cagayan de Oro City, Philippines, ^6^Iloilo Doctors' College, Research Department, Iloilo City, Philippines

**Background**: In the Philippines, a needs assessment showed significant associations between unemployment, homelessness and mental distress among PLHIV. Gabay sa Pulang Laso Inc. (GPLI) integrated Supportive‐Expressive Group Therapy (SEGT) into its Open Doors Home (ODH) initiative (a temporary shelter provision and socio‐economic support for unemployed PLHIV experiencing homelessness). The core foundation of ODH‐SEGT is reconnecting with oneself through a supportive and empowering environment where a PLHIV shares experiences with fellow PLHIV while addressing socio‐economic needs through employment and education opportunities.

**Description**: Through open social media invitations, 22 PLHIV voluntarily participated and completed the ODH‐SEGT Programme between 19 August and 30 October 2022. The intervention was conducted in four phases: (1) baseline screening for anxiety‐ and depression‐related symptoms using a 7‐item Generalized Anxiety Disorder and 9‐item Patient Health Questionnaire (GAD‐7 and PHQ‐9); (2) assessment of PLHIV perceived socio‐economic needs; (3) conducting 12‐week SEGT sessions with bi‐monthly GAD‐7 and PHQ‐9 monitoring; and (4) linkage to employment or education.

**Lessons learned**: The participants’ age ranged between 19 and 52 years old (mean = 33.3 years old, SD = 7.9). Among 22 participants, 18 were unemployed, while four had stopped attending school. At the end of the ODH‐SEGT intervention, 16 were linked to employment, while five were included in GPLI educational support and are currently attending the alternative learning system of the government. While attending the SEGT, averages in PHQ‐9 and GAD‐7 at baseline were at moderate levels (12.2 and 12.4, respectively) and significantly decreased to be at low to no risk by Week 10 (4.8 and 4.8, respectively). One participant reported to have stopped taking antiretroviral medication for 5 years and was immediately linked back to HIV care.

**Conclusions/Next steps**: GPLI's ODH‐SEGT has the potential to improve mental health of people living with HIV by addressing their non‐biomedical needs and contributing to a higher quality of life. The next step for the project is the development of the Training of Trainers programme so that the SEGT framework can be replicated in other regions of the country. In addition, a multisectoral collaboration is currently being advocated with the local government unit, social welfare department and academic institutions in the Philippines.

### OAE0102 Anticipated preferences for long‐acting HIV PrEP among current oral PrEP users at pharmacies: findings from a pilot study extension

S.D. Roche ^1^, V. Omollo^2^, P. Mogere^3^, M. Asewe^2^, S. Gakuo^3^, P. Banerjee^1^, K. Harkey^1^, J. Odoyo^2^, P. Ongwen^4^, D. Were^4^, E. Bukusi^2,5^, K.F. Ortblad^1^, K. Ngure^3,6^

^1^Fred Hutchinson Cancer Center, Public Health Sciences, Seattle, United States, ^2^Kenya Medical Research Institute, Kisumu, Kenya, ^3^Partners in Health Research and Development, Thika, Kenya, ^4^Jhpiego, Nairobi, Kenya, ^5^University of Washington, Seattle, United States, ^6^Jomo Kenyatta University of Agriculture and Technology, Nairobi, Kenya

**Background**: As many sub‐Saharan African countries prepare to implement two long‐acting (LA) forms of HIV pre‐exposure prophylaxis (PrEP)—injectable PrEP and the dapivirine vaginal ring (DVP‐VR)—there is potential to expand delivery to new access points, such as private pharmacies. A recent pilot study of private pharmacy‐delivered oral PrEP in Kenya found that these venues may reach a different demographic than public clinics. During a 6‐month extension of this study, we assessed the current oral PrEP users’ anticipated preferences for LA PrEP.

**Methods**: At 12 pharmacies in Kisumu and Kiambu Counties, Kenya, trained pharmacy providers delivered oral PrEP to eligible clients ≥18 years. We surveyed PrEP clients 1 month following PrEP initiation, and asked whether, if given the option, they would choose to take oral PrEP every day or get a PrEP injection every 2 months. For those selecting “PrEP injection,” we further explained that the injection would go into the muscles of the buttocks region and resolicited their preferences. Lastly, we described the DVP‐VR to female clients and asked them to rank oral PrEP, injectable PrEP and DVP‐VR in order of preference. We report our findings descriptively.

**Results**: From January to July 2022, we surveyed 491 pharmacy PrEP clients; 55% (270/491) were female, median age was 22 (IQR: 25–32) and 84% (412/491) had never taken oral PrEP prior to this study. About two‐thirds (65%, 319/491) anticipated they would choose injectable over oral PrEP. After being told details about the injection site, 58% (287/491) still reported a preference for injectable PrEP, with this preference notably higher among males (77%, 170/221) than females (43%, 117/270). Among females, 57% (155/270) said their first choice would be injectable PrEP, followed by oral PrEP, then DVP‐VR; an additional 30% (81/270) of females chose oral PrEP first, followed by injectable PrEP, then DVP‐VR.

**Conclusions**: Over half of the current oral PrEP users at private pharmacies anticipate that, if given the option, they would choose injectable over oral PrEP. Few female oral PrEP users in this setting thought they would choose the DVP‐VR. More research is needed to test pharmacy delivery of LA PrEP and assess real‐world uptake and product switching.

### OAE0103 Drivers of pre‐exposure prophylaxis choice for transgender women in 11 countries in Asia: a discrete choice experiment

W. Tieosapjaroen ^1,2^, B.R. Bavinton^3^, H.‐M. Schmidt^4,5^, K.E. Green^6^, N. Phanuphak^7^, M. Poonkasetwattana^8^, N.S. Suwandi^8^, D. Fraser^3^, C. Chan^3^, H. Boonyapisomparn^9^, M. Cassell^10^, L. Zhang^1,2^, W. Tang^11^, J.J. Ong^1,2,12^

^1^Alfred Health, Melbourne Sexual Health Centre, Carlton, Australia, ^2^Monash University, Central Clinical School, Melbourne, Australia, ^3^University of New South Wales, Kirby Institute, Sydney, Australia, ^4^UNAIDS Regional Office for Asia and the Pacific, Bangkok, Thailand, ^5^Global HIV, Hepatitis and STIs Programme, World Health Organization, Geneva, Switzerland, ^6^The Program for Appropriate Technology in Health, Hanoi, Vietnam, ^7^Institute of HIV Research and Innovation, Bangkok, Thailand, ^8^APCOM Foundation, Bangkok, Thailand, ^9^Asian Pacific Transgender Network, Bangkok, Thailand, ^10^Family Health International 360, Hanoi, Vietnam, ^11^The University of North Carolina at Chapel Hill Project‐China, Guangzhou, China, ^12^London School of Hygiene and Tropical Medicine, Faculty of Infectious and Tropical Diseases, London, United Kingdom

**Background**: Transgender women (TGW) are approximately 66 times more at risk of HIV acquisition than the general population. Designing appealing pre‐exposure prophylaxis (PrEP) programmes for TGW is urgently needed. We evaluated the drivers of choice for PrEP among TGW living in 11 Asian countries and forecasted their PrEP uptake given different PrEP programme configurations.

**Methods**: An online discrete choice experiment (DCE) survey was delivered through trans‐networks in each country between May and November 2022. Participants who identified as TGW, age ≥ 18 years and had no prior HIV diagnosis were included. Final attributes included: (1) type of PrEP; (2) service location; (3) cost; (4) side effects; (5) visit frequency; and (6) extra services. We calculated the relative importance of each attribute and PrEP uptake prediction using random parameters logit (RPL) models.

**Results**: Overall, 1522 TGW were included, with a mean age of 28.1 (±7.0), 63% (956/1522) reported multiple partners, 38% (581/1522) had condomless vaginal sex and 16% (249/1522) were diagnosed with a sexually transmitted infection (STI) in the last 6 months. The biggest drivers of PrEP uptake were cost (62% relative importance), type of PrEP (10%), location (8%), extra services (8%), visit frequency (7%) and side effects (5%). The most wanted PrEP service (with a predicted uptake of 87%) was: free injectable PrEP with no side effects, accessing PrEP from a peer‐led community clinic that provided STI testing and requiring 6–12 monthly visits. The least preferred PrEP service (with a predicted uptake of 50%) was: PrEP implant with out‐of‐pocket fees and a rare chance of kidney problems, accessing PrEP from a hospital, no extra services and requiring 2‐monthly visits.

**Figure:**
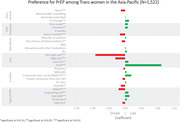
**Abstract OAE0103‐Figure 1**.

**Conclusions**: Our study, the largest DCE for TGW globally, emphasizes the importance of measuring and incorporating preferences for PrEP services to accelerate the scale‐up of PrEP among TGW in Asia.

### OAE0104 Snow balling peer to peer mModel—a silver bullet for improved PrEP uptake among FSWs in Zambia's border town of Chirundu

K. Mwanda ^1^, E. Berghammer^2^, J. Masedza^3^, D. Mazunda^3^, M. Simata^4^

^1^USAID CHEKUP II, Technical, Lusaka, Zambia, ^2^USAID, Social Protection, Lusaka, Zambia, ^3^USAID CHEKUP II, Clinical, Chirundu, Zambia, ^4^Ministry of Health, Clinical, Chirundu, Zambia

**Background**: With an annual HIV incidence rate of 47%, over 56% of the female sex workers in Zambia are living with HIV (UNAIDS, 2020). However, the uptake of pre‐exposure prophylaxis among FSWs in the Zambia‐Zimbabwe border town of Chirundu remains low at 22% of those who test negative.

**Description**: Between December 2022 and January 2023, USAID CHEKUP implemented by John Snow Health Zambia embarked on an initiative to orient and then engage FSWs already accessing PrEP to identify peers in their network, provide PrEP messaging and facilitate them to take up PrEP. In a snow ball approach, five trained healthcare workers trained a pool of 15 community health workers on how to provide orientation around PrEP messaging to 75 FSWs who were mostly (75%) aged 20–24 years. FSWs in turn delivered these PrEP promotion messages alongside sharing their lived experiences around the benefits of PrEP, addressed the misgivings and facilitated the linkage to PrEP.

**Lessons learned**: Over a 3‐week period, the 75 FSWs reached out to 284 of their peers among which 43 (15.1%) were discovered to be positive and already on treatment, 61 (21.5%) were newly identified positives and were linked for antiretroviral therapy. From the remaining 180 HIV‐negative FSWs, 164 (91.1%) were initiated on PrEP, while the rest (16) (8.9%) indicated that they needed to think about it, or the time was not appropriate and that they could commence at a later date. The majority (52%) of the FSWs that took up PrEP were in the same age band (20–24 years old) as their PrEP promoting peers. Being spoken to by someone who is in my situation and also taking the medicine (85%) and making it convenient for me to access the medicine (71%) were the prominent reasons given by the new recipients for then to be initiated on PrEP.

**Conclusions/Next steps**: With the PrEP uptake under the peer‐to‐peer modality being significantly higher than the routine approach (91.1% vs. 22%), the peer‐to‐peer model has a potential to increase both acceptability and uptake of PrEP among FSWs particularly for programmes recording low PrEP usage among FSWs.

### OAE0105 The acceptability of pharmacy‐delivered PrEP and PEP in Kenya: provider perceptions

M. Asewe ^1^, B. Kwach^1^, S. Zhang^2^, K. Harkey^3^, G. Rota^1^, P. Otieno^1^, J. Odoyo^1^, P. Ongwen^4^, D. Were^4^, S. Roche^3^, K. Ngure^5^, V. Omollo^1^, E. Bukusi^1,2^, K. Ortblad^3^

^1^Kenya Medical Research Institute, Kisumu, Kenya, ^2^University of Washington, Seattle, United States, ^3^Fred Hutchinson Cancer Center, Seattle, United States, ^4^Jhpiego, Nairobi, Kenya, ^5^Jomo Kenyatta University of Agriculture and Technology, Nairobi, Kenya

**Background**: Pilot studies indicate that delivering oral HIV pre‐ and post‐exposure prophylaxis (PrEP and PEP) at private pharmacies may help increase access to these highly effective HIV prevention products among individuals with HIV risk. In many high HIV prevalence settings, most of the core components of PrEP and PEP delivery (e.g. counselling, medical safety assessment, HIV testing and drug dispensing) fall within pharmacy providers’ scope of practice. We assessed the anticipated acceptability of delivering PrEP and PEP at private pharmacies among pharmacy providers in Kenya.

**Methods**: Pharmacists and pharmaceutical technologists working at 20 private pharmacies in Kisumu County completed a cross‐sectional questionnaire that assessed acceptability using questions informed by the Theoretical Framework of Acceptability (TFA). The TFA posits that acceptability is a multi‐faceted construct, comprising seven component constructs (e.g. affective attitude towards the intervention, perceived effectiveness, burden and opportunity cost). We measured select acceptability component constructs using 5‐point Likert items and analysed these using descriptive statistics. Statements with which >80% of participants agreed or strongly agreed were classified as “acceptable.”

**Results**: From May to June 2022, 40 pharmacy providers completed the questionnaire; 42% (*n* = 17) were pharmacy owners, 60% (*n* = 24) were male and the median time in pharmacy practice was 6 years (IQR 4–10). All or nearly all providers (97%–100%) liked the idea of delivering PrEP and PEP (TFA construct: affective attitude) and thought that pharmacy‐delivered PrEP and PEP services could reach people with HIV risk (TFA construct: perceived effectiveness). Most pharmacy providers (96%–99%) did not think it would be hard to deliver PrEP or PEP (TFA construct: burden) or that this would interfere with their other priorities (TFA construct: opportunity cost).

**Conclusions**: The Kenyan pharmacy providers in this study found the idea of pharmacy‐delivered PrEP and PEP services to be highly acceptable. To inform whether and how pharmacy‐delivered PrEP and PEP should be implemented and scaled up in Kenya and similar settings, additional research is needed to develop and test delivery models, assessing both effectiveness outcomes (e.g. uptake and continuation) and implementation outcomes (e.g. acceptability, feasibility and cost).

### OAE0202 Leveraging community and private‐sector HIV self‐testing (HIVST) distribution to improve access to HIV testing services (HTS) and treatment for adolescent girls and young women (AGYW) in Uganda

J. Tumusiime ^1^, G. Taasi^2^, J. Musinguzi^1^, P. Kikobye^1^, A. Scott^3^, D. Canagasabey^3^, K. Granger^3^, I. Thior^3^, K.E. Green^4^

^1^PATH, Kampala, Uganda, ^2^Ministry of Health, Kampala, Uganda, ^3^PATH, Washington DC, United States, ^4^PATH, Hanoi, Vietnam

**Background**: Twenty‐nine percent of new HIV infections in Uganda occur among AGYW, and 32% of estimated adolescents living with HIV are not on treatment (Uganda AIDS Commission 2021). There is a need for strategies that better engage AGYW in HIV case‐finding and linkage to care‐given persisting gaps. Through the Unitaid/STAR‐III project, PATH, with Ministry of Health, leveraged human‐centred design to adapt HIVST services to improve reach and uptake of HIV services for AGYW.

**Description**: We introduced community and private‐sector distribution models to expand outlets for AGYW to access HIVST beyond public‐sector facilities, including targeted community outreach, hotspots, tertiary educational institutions, pharmacies and nurse/midwife‐led clinics. We trained AGYW peers to lead mobilization during community campaigns or at popular hangout areas; link interested clients to healthcare providers for assisted or unassisted HIVST; follow up by phone/WhatsApp to confirm results; and provide escorted referrals for those with reactive self‐test results. We developed easily downloadable HIVST training videos that peers could share through social media for AGYW who preferred anonymous access options. We analysed programme data from three central Ugandan districts from November 2020 to November 2022 to understand the impact of these adaptations on HIVST uptake and preferences among AGYW.

**Lessons learned**: AGYW (69% between 20 and 24 years) received HIVST kits, 60% through community distribution (primarily targeted community or hotspot modalities), 22% through public sector (mainly outpatient services) and 18% through private sector (mainly nurse/midwife‐led clinics). Two hundred and twenty‐four (2.4%) had reactive results, among whom 184 (82%) were confirmed HIV positive (2.0% positivity), 182 (99%) were linked and 144 (79%) initiated on treatment. AGYW demonstrated preference for directly assisted HIVST (51%), and half had not tested in the last 12 months. Using peers to lead engagement/escort referrals for follow‐on care was key to high follow‐up and linkage rates.

**Conclusions/Next steps**: Our results highlight the feasibility and acceptability of using community and private‐sector outlets, shareable online content, and peer‐led outreach and referral to effectively reach and link AGYW to treatment through HIVST. Diversifying HTS access points and reinforcing peer‐driven models are core approaches that should be scaled up to address persisting access barriers among adolescents and enable Uganda to reach epidemic control.

### OAE0203 Community Led Monitoring (CLM): a new CLM toolkit to generate evidence to support community‐led HIV service improvements

M. Kusen ^1^, C. Byambaa^2^, G. Nyampurev^2^, J. Oorloff^3^, P. Vjayabandara^4^, T. Dendup^5^, J. Norbu^5^, B. Morris^6^, S. Anthony^7^, M. Merrigan^1^

^1^Australian Federation of AIDS Organisations, Bangkok, Thailand, ^2^Youth for Health Centre, Ulaanbaatar, Mongolia, ^3^Family Planning Association of Sri Lanka, Colombo, Sri Lanka, ^4^Positive Hopes Alliance, Colombo, Sri Lanka, ^5^Save the Children, Thimpu, Bhutan, ^6^The Global Fund to Fight AIDS, Tuberculosis and Malaria, Geneva, Switzerland, ^7^APCOM, Bangkok, Thailand

**Background**: Community Led Monitoring (CLM) shifts the dynamic of monitoring from HIV service providers to monitoring led by the people using the services. In order to build an evidence‐base for CLM, the Global Fund supported Sustainability of HIV Services for Key Populations in Asia (SKPA‐2) programme has developed the “Sustainable Community‐led Monitoring of HIV Services Toolkit for Key Populations.” This provides a framework, indicators and guidance on how to ensure the CLM process is community‐led and guided through measurable bottom‐up approaches.

**Description**: Launched in 2022, the CLM toolkit has been piloted in Mongolia, Bhutan and Sri Lanka for key population groups, including men who have sex with men, transwomen, sex workers, people living with HIV and people who use drugs. The toolkit provides a framework of eight core indicators to monitor Availability, Accessibility, Acceptability and Quality (AAAQ) along with five indicators to measure and ensure there is follow‐up for those experiencing stigma, discrimination and/or violence. The selection of final indicators, pilot sites, data processes and follow‐up should be decided by CLM technical working group made up of national stakeholders and includes key population representatives.

**Lessons learned**:

Helped identify the type of facilities where stigma and discrimination (S&D) was more prevalent to support more targeted efforts to reduce S&D in healthcare settings.

Supported the identification of the type of services with the least availability, including CD4 count and viral load testing.

CLM data should be designed to share data visually and in an easy to digest manner to community groups, such as through electronic dashboards.

Funding for CLM is not assured as health programmes also have their own feedback systems, so community led models need to be cost‐effective.

Identified the need to include well‐managed referral mechanisms to accountability mechanisms to manage and deal with experiences of S&D when accessing HIV services.

**Conclusions/Next steps**: The CLM toolkit helps provide interesting findings for other countries when designing and rolling out CLM. Future research and programmes may include an evaluation to assess the impact CLM has on supporting countries to improve service quality and achieve their 95‐95‐95 targets as well as the sustainability of CLM funding.

### OAE0204 Uptake of integrated HIV and sexual and reproductive health services for youth at community centres in Zimbabwe

V. Simms^1,2^, E. Dauya^2^, C. Dziva Chikwari^1,2^, T. Bandason^2^, K. Kranzer^3,2^, R.A. Ferrand ^3,2^, CHIEDZA Trial Team

^1^London School of Hygiene & Tropical Medicine, MRC International Statistics and Epidemiology Group, London, United Kingdom, ^2^The Health Research Unit Zimbabwe (THRU ZIM), Harare, Zimbabwe, ^3^London School of Hygiene & Tropical Medicine, Clinical Research Department, London, United Kingdom

**Background**: Limited engagement with health services contributes to the poorer HIV outcomes observed in youth. We conducted a cluster‐randomized trial of a community‐based integrated HIV and sexual and reproductive health (SRH) service (CHIEDZA) for youth in three provinces in Zimbabwe.

**Methods**: Weekly integrated HIV and SRH services were delivered from community centres in 12/24 intervention clusters to cluster residents aged 16–24 years over 30 months. Fingerprint scanning was used to anonymously identify clients and track their attendances and service uptake over time. Services included HIV testing, treatment and adherence support, management of sexually transmitted infections (STIs), menstrual health management, contraception, counselling and registration for text messages on SRH topics. All services were optional.

**Results**: In total 36,991 clients attended, for a total of 78,810 visits; each centre had a median of 55 clients per day; 40.6% of clients returned for more than one visit. Overall, 75.0% of clients were female and 53.0% were aged <20 years. In total, 84.1% of eligible clients had at least one HIV test and 17.4% had more than one. At their first visit, 78.6% of eligible clients had an HIV test, and out of those who were not tested at the first visit, 28.3% later returned and were tested. Three hundred and seventy‐seven clients tested HIV positive at CHIEDZA (prevalence 1.3%) and 75% linked to care, while 1162 clients were previously diagnosed young people living with HIV. HIV incidence among those with repeated visits was 0.72 per 100 person years (95% CI 0.53–0.98). The most popular services for women were menstrual hygiene products (taken up by 96.5% of those eligible), HIV testing (83.7%) and period pain management (59.9%); for men, the most popular were condoms (93.9%), HIV testing (85.6%) and text messages on SRH (67.1%). Among women aged ≥20 years, 43.7% took condoms and 60.3% took up other forms of contraception.

**Conclusions**: An integrated HIV and SRH programme had high attendance and uptake, with most clients taking multiple services per visit, including HIV testing. There is a need for accessible, youth‐friendly sources of SRH information, menstrual health management, contraception and HIV testing in Zimbabwe.

### OAE0205 Utilizing community HIV/AIDS service agents to find, link and retain in care pregnant, breastfeeding mothers and children aged 0–14 living with HIV

G. Kadziyanhike ^1^, G. Kadziyanike^1^, C. Mademutsa^1^, A. Nhapi^1^, T. Mwareka^1^

^1^Zimbabwe National Network of People Living with HIV, Harare, Zimbabwe

**Background**: Despite efforts to end the AIDS epidemic by 2030, pregnant and breastfeeding mothers, children and young adolescents continue to face barriers to accessing quality HIV and other health‐related services. The Zimbabwe National Network of People Living with HIV (ZNNP+) has collaborated with communities in four districts of Zimbabwe to improve service access for these groups by increasing awareness of available services and referring those who have interrupted treatment into care. Furthermore, community cadres met with community leaders to discuss barriers to service access identified during their work.

**Description**: As a community partner, the Zimbabwe National Network of People Living with HIV compliment the Ministry of Health and Child Care in ensuring that pregnant and breastfeeding women and children aged 0–14 are linked and retained in care. The organization trained and deployed 40 mentor mothers and 40 young Community HIV/AIDS Service Agents (yCHASAs) in the community who helped to track and trace those not in care. Each month, the cadres submit data on their activities electronically to the central server where it is analysed, and insights generated to inform advocacy at the facility or community.

**Lessons learned**: Between July 2022 and September 2022, the mentor mothers referred 1612 individuals for HIV services, 52 (3%) who had an unknown HIV status got tested and had a positive result. The newly diagnosed were initiated on treatment and linked to support groups, while community cadres continued to provide adherence counselling. During the same time, 48 (3%) of those who had been interrupted in treatment (IIT) successfully returned to care. Ten survivors of gender‐based violence (GBV) were linked to appropriate services. The cadres coordinated three community dialogues, which were attended by 90 community leaders, to discuss feedback from recipients of care to jointly find solutions to structural barriers that prevent people from accessing services.

**Conclusions/Next steps**: With proper training, lay cadres can identify peers who are not on treatment or those interrupted in treatment and successfully navigate them back into care. If properly engaged, community leaders are willing to offer cadres a platform for them to air concerns from their peers and help break down barriers to service access.

### OAE0302 Long‐acting HIV pre‐exposure prophylaxis (PrEP) among adolescent girls and young women (AGYW) in South Africa: cost‐effective at what cost?

E.Y. Jin^1^, A.R. Ahmed^1^, L.‐G. Bekker^2^, A. Ciaranello^1,3,4^, C.F. Flanagan^1^, K.A. Freedberg^1,3,4,5^, C. Orrell^2^, K.P. Reddy^1,4,6^, M. Wallace^2^, A.M. Neilan ^1,3,4,7^

^1^Medical Practice Evaluation Center, Boston, United States, ^2^Desmond Tutu HIV Centre, University of Cape Town, Cape Town, South Africa, ^3^Massachusetts General Hospital, Division of Infectious Diseases, Department of Medicine, Boston, United States, ^4^Harvard Medical School, Boston, United States, ^5^Massachusetts General Hospital, Division of General Internal Medicine, Boston, United States, ^6^Massachusetts General Hospital, Division of Pulmonary and Critical Care Medicine, Boston, United States, ^7^Massachusetts General Hospital, Division of General Academic Pediatrics, Department of Pediatrics, Boston, United States

**Background**: HIV Prevention Trials Network (HPTN) 084 demonstrated superior efficacy of long‐acting, injectable cabotegravir (CAB‐LA) compared to daily oral tenofovir/emtricitabine (TDF‐FTC) for HIV PrEP in cisgender women. We projected the drug cost at which CAB‐LA would provide good value compared to TDF‐FTC among adolescent girls and young women (AGYW) in South Africa.

**Methods**: Using microsimulation modelling, we examined two PrEP strategies over 10 years among AGYW (ages 15–30 y; scaled to *n* = 10,000): TDF‐FTC and CAB‐LA. Published data informed model inputs, including: HIV incidence (TDF‐FTC: 1.85/100 person‐years, CAB‐LA: 0.2/100 person‐years; HIV transmissions off‐PrEP from 10,000 AGYW to partners (16/year); and 2‐year retention (TDF‐FTC: 88%, CAB‐LA: 85%). We assumed constant incidence and transmission risk over time. Annual costs included: PrEP drug+programme (TDF‐FTC: $77+$74, CAB‐LA: $153+$75), ART ($58–$834) and HIV‐related care ($215–$1621). Model‐projected outcomes include incident infections among and transmissions from AGYW, life‐years (LYs), costs, incremental cost‐effectiveness ratios (ICER = $/LY) and CAB‐LA's maximum price premium (MPP: the highest drug price at which CAB‐LA would have an ICER below a willingness‐to‐pay [WTP] of 50% South Africa's per‐capita GDP [$3500/LY]).

**Results**: Per 10,000 AGYW in South Africa, projected infections and transmissions were higher and LYs lower in TDF‐FTC (2050 infections/658 transmissions/85,889 LYs), compared to CAB‐LA (1151/342/86,057) (Table). HIV infections avoided among male partners resulted in 143 LYs gained in CAB‐LA over TDF‐FTC. At $153/year drug cost, CAB‐LA would exceed the WTP threshold (ICER = $6600/LY). The projected MPP for CAB‐LA to be cost‐effective and cost‐saving would be $136 and $118/year, respectively. Accounting only for the benefits accruing to AGYW, the MPP to be cost‐effective would be lower, $122/year. Varying transmissions from 2 to 40/year would yield an MPP of $124–158/year to remain cost‐effective.

**Table:** 

**Abstract OAE0302‐Table 1. Ten‐year model‐projected outcomes of CAB‐LA versus TDF‐FTC for HIV PrEP among adolescent girls and young women in South Africa (*n* = 10,000)**										
			Discounted LYs		Discounted costs, USD		ICER ($/LY)^a^		CAB‐LA maximum price premium, USD	
Strategy	Incident infections in AGYW, *n*	Transmissions to male partners, *n*	AGYW	LYs gained from partners^b^	AGYW	Costs saved from partners^b^	AGYW	All	AGYW	All
TDF‐FTC	2050	658	85,889		12,579,969					
CAB‐LA	1151	342	86,057	143	14,979,829	340,232	14,300	6600	122	136

Abbreviations: AGYW, adolescent girls and young women; CAB‐LA, long‐acting, injectable cabotegravir; ICER, incremental cost‐effectiveness ratio; LY, life‐year; PrEP, pre‐exposure prophylaxis; TDF‐FTC, tenofovir/emtricitabine; USD, United States Dollars.

^a^ The ICER is the difference in cost divided by the difference in life expectancy for each strategy compared with the next less‐costly strategy. Results are rounded to the nearest $100.

^b^ Life‐years gained and costs saved are among male partners in CAB‐LA compared to TDF‐FTC who, in the absence of the PrEP strategy being provided to AGYW, would have acquired HIV.

Life‐years and costs are discounted at 3%/year and scaled to *n* = 10,000 AGYW.

**Conclusions**: Among AGYW in South Africa, CAB‐LA could reduce transmissions and increase life‐years compared to TDF‐FTC. CAB‐LA should be priced at less than twice the cost of TDF‐FTC to be cost‐effective in South Africa.

### OAE0303 Predicted HIV acquisition rates for cabotegravir versus TDF/FTC as PrEP in Brazil: effects of compulsory licensing

A. Hill ^1^, S. Cross^2^, T. Pepperrell^3^, K. Heath^4^, M. Mirchandani^5^

^1^University of Liverpool, Department of Pharmacology and Therapeutics, Liverpool, United Kingdom, ^2^Imperial College London, School of Medicine, London, United Kingdom, ^3^University of Edinburgh, School of Medicine and Veterinary Medicine, Edinburgh, United Kingdom, ^4^Burnet Institute, Melbourne, Australia, ^5^Imperial College London, Faculty of Medicine, London, United Kingdom

**Background**: Worldwide, 1.5 million people acquired HIV in 2021. Cabotegravir is the most effective drug to prevent HIV acquisition, with an estimated efficacy of 90%–95%, superior to oral tenofovir/emtricitabine (TDF/FTC). TDF/FTC is available as generic PrEP in most countries, costing $48/year in LMICs. CAB‐LA costs $22,200/year in the United States and $9000 in the UK. The ViiV‐MPP licence permits generic companies to sell CAB‐LA at low prices, estimated $250/year. However, the ViiV‐MPP agreement excludes many middle‐income countries, such as Brazil. CHAI estimated costs of production of $34–$63/year (median $50).

**Methods**: We modelled the effects of four strategies for PrEP in Brazil: (1) No PrEP used. (2) TDF/FTC generic used for PrEP, costing $48/year. (3) CAB‐LA used, with a high price, outside the ViiV‐MPP license: $3500/year (68% below UK price), or $250/year (target price). (4) CAB‐LA used at CHAI target price $50/year. TDF/FTC and CAB‐LA were assumed to lower HIV acquisition risks by 70% and 94%, respectively, versus no PrEP. We assumed a target population of 125,000 people at high risk of HIV acquisition (incidence 6%), treated in Brazil with an annual budget of $6 million.

**Results**: Using TDF/FTC costing $48/year, the $6 million Brazilian PrEP budget could treat all 125,000 people, lowering the annual HIV acquisition from 8750 to 2525/year. By contrast, the use of CAB‐LA for $3500/year covers 1714 people; the remaining 123,285 people receive no PrEP. Even at target prices of $250/year, the overall HIV acquisition rates are still higher. The CHAI cost price of $50/year would provide for 120,000 people, lowering HIV acquisition to 854/year. CAB‐LA needed to cost less than $80/year to lead to fewer HIV acquisitions than TDF/FTC.

**Conclusions**: When demand for PrEP is high and budgets are limited, CAB‐LA will only lower the overall HIV acquisition rates if costing <$80/year. If prices are higher, limited people can be given CAB‐LA: then, mass use of TDF/FTC for $48/year allows an increased coverage, lowering the overall HIV acquisition rates. Compulsory licensing may be required to lower CAB‐LA prices in countries outside the ViiV‐MPP voluntary license.

**Table:** 

**Abstract OAE0303‐Table 1**.			
Budget	Cost of PrEP	Number given PrEP	HIV acquisitions
**Current PrEP budget‐$6 million**			
No PrEP	$0	0	8750
TDF/FTC	$48	125,000	2250
CAB‐LA base case	$3500	1714	8637
CAB‐LA target price	$250	24,000	6632
CAB‐LA cost price	$50	120,000	732

### OAE0304 Cost‐effectiveness and budget impact analysis of the implementation of differentiated service delivery models for HIV treatment in Mozambique—a modelling study

D. Moiana Uetela ^1,2^, M. Zimmermann^3^, O. Uetela^2^, E. Samo Gudo^1^, S. Chicumbe^1^, A. Couto^4^, I. Gaspar^4^, K. Sherr^2,5,6^

^1^Instituto Nacional de Saúde, Marracuene, Mozambique, ^2^University of Washington, Department of Global Health, Seattle, United States, ^3^University of Washington, The Comparative Health Outcomes, Policy, and Economics (CHOICE) Institute, Seattle, United States, ^4^Ministry of Health, National STI‐HIV/AIDS Program, Maputo, Mozambique, ^5^University of Washington, Department of Epidemiology, Seattle, United States, ^6^University of Washington, Department of Industrial & Systems Engineering, Seatle, United States

**Background**: In 2018, Mozambique's Ministry of Health launched a guideline to implement eight differentiated service delivery models (DSDMs) to optimize HIV service delivery, improve retention in care, and ultimately reduce HIV associated mortality. The models were:

1. Fast‐track (FT),
2. Three‐month antiretrovirals dispensing (3M),
3. Community antiretroviral therapy (ART) groups (CAGs),
4. Adherence clubs (AC),
5. Family‐approach (FA), and three one‐stop shop models:
6. adolescent‐friendly health services (OSS‐AFHS),
7. maternal and child health (OSS‐MCH), and
8. tuberculosis (OSS‐TB).

We conducted a cost‐effectiveness analysis (CEA) and a budget impact analysis (BIA) comparing these DSDMs to conventional services.

**Methods**: We constructed a decision tree model based on the percentage of enrolment on each DSDM and the probability of the outcome (12 months retention on ART), with and without DSDMs implementation, for each year of the study period; three for CEA (2019–2021) and three for BIA (2022–2024). The economic and financial costs for CEA and BIA, respectively, were estimated per client‐year from the health system perspective, and included start‐up, ARV drugs, laboratory tests, and clinical and pharmacy visits. Effectiveness was estimated using the Mozambique ART database, employing an uncontrolled interrupted time series analysis comparing the outcome before and during the implementation of DSDMs. A one‐way sensitivity analysis was conducted to identify drivers of uncertainty.

**Results**: During the 3 years of DSDMs implementation, there was a mean increase of 14.9 percentage points (95% confidence interval [CI]: 12.2, 17.8) in 12 months retention comparing DSDMs implementation (62.5% [95% CI: 60.9, 64.1]) to conventional care (47.6% [95% CI: 44.9, 50.2]), and the mean base‐case economic cost per person‐year was estimated to be $253 and $359 for DSDMs and convention care, respectively; therefore, DSDMs dominated conventional care. The base‐case 3‐year financial costs associated with the DSDMs and the conventional care for a population of 1,535,575 were estimated to be $1,653,814,275 and $990,194,425, respectively. The results were most sensitive to clinical visit costs.

**Conclusions**: DSDMs were less expensive and more effective in retaining clients 12 months after ART initiation, and their implementation was estimated to save approximately $670 million to the health system from 2022 to 2024.

### OAE0305 Cost‐effectiveness of the WHO‐endorsed advanced HIV care package in Malawi

E. Hyle ^1^, T. Maphosa^2^, A. Rangaraj^3^, M. Feser^1^, K. Reddy^1^, A. Shroufi^4^, P. Shrestha^1^, R. Horsburgh^5^, N. Ford^3^, A. Tiam^6^, A. Phillips^7^, K. Freedberg^1^

^1^Massachusetts General Hospital, Boston, United States, ^2^Elizabeth Glaser Pediatric AIDS Foundation, Lilongwe, Malawi, ^3^World Health Organization, Geneva, Switzerland, ^4^The Global Fund, Geneva, Switzerland, ^5^Boston University School of Public Health, Boston, United States, ^6^Elizabeth Glaser Pediatric AIDS Foundation, Washington, D.C., United States, ^7^University College London, London, United Kingdom

**Background**: In sub‐Saharan Africa, more than 20% of PLHIV present with advanced HIV disease (AHD). Our objective was to project the clinical outcomes, costs and cost‐effectiveness of the WHO‐recommended AHD package of care in Malawi.

**Methods**: Using the validated CEPAC‐I model, we simulated a cohort of PLHIV aged ≥18 y initiating ART with measured CD4 count using published data and assessed seven strategies applied to people identified with AHD (CD4<200/μl and/or WHO 3/4 disease): (1) no specific AHD care (sputum Xpert for people with TB symptoms), and then sequential addition of: (2) urine LAM; (3) co‐trimoxazole (CTX); (4) cryptococcal antigen (CrAg) and CTX; (5) LAM and CTX; (6) LAM, CTX and CrAg; (7) LAM, CTX, CrAg and isoniazid preventive therapy (IPT). The cohort had mean age 37 y, 51% were female and mean CD4 362/μl (15% with CD4 <200/μl and 4% with WHO3/4 disease and CD4 ≥200/μl). Among people with CD4 <200/μl, the prevalence was: 18%–37% TB disease; 20%–39% latent TB; 5% asymptomatic cryptococcal disease; and 87% of people with TB disease had symptoms. Test costs were $13 (Xpert), $5 (LAM) and $3 (serum CrAg); medication costs were $0.83/month (CTX), $4/month (Fluconazole) and $1/month (IPT). Model outcomes included life expectancy, costs and incremental cost‐effectiveness ratio (ICER, $/year‐of‐life saved [YLS]); we considered ICERs <$640/YLS (Malawi's annual per capita GDP) cost‐effective.

**Results**: All AHD strategies would improve clinical outcomes and increase costs (Table). The full AHD package, LAM+CTX+CrAg+IPT, would result in the greatest life expectancy (21.61 life‐years) and be cost‐effective (ICER, $250/YLS). All other strategies would be less efficient than the full AHD package at the cost‐effectiveness threshold. Results are most sensitive to TB and cryptococcemia prevalence.

**Conclusions**: Using published data, the full AHD package at ART initiation would be cost‐effective in Malawi compared with only some elements of the package, when CD4 count is measured.

Table. Model‐projected outcomes comparing the clinical outcomes, costs and cost‐effectiveness of different strategies for AHD care for PLHIV initiating ART in Malawi

**Table:** 

**Abstract OAE0305‐Table 1**.			
Strategy	1 y survival (%)	Undiscounted life expectancy (y)	Discounted life expectancy (y)^a^	Lifetime costs ($)^a^	ICER ($/YLS)^a^
No AHD care (ART+Xpert)	93.89	20.89	13.62	1354	–
+LAM	94.20	21.13	13.74	1371	140
+CTX	94.31	21.21	13.81	1412	dom
+CrAg+CTX	94.34	21.21	13.81	1413	dom
+LAM+CTX	94.64	21.45	13.93	1430	dom
+LAM+CTX+CrAg	94.66	21.46	13.94	1431	dom
+LAM+CTX+CrAg+IPT	94.88	21.61	14.03	1444	250

Abbreviations: AHD, advanced HIV disease; CrAg, cryptococcal antigen; CTX, co‐trimoxazole; ICER, incremental cost‐effectiveness ratio; IPT, isoniazid preventive therapy; LAM, lipoarabinomannan; PLHIV, people living with HIV; YLS, years‐of‐life saved.

^a^ Discounted at 3%/year. Dominated (dom): the ICER of this strategy compared to the next more costly strategy is higher and, therefore, not preferred.

### OAE0402 Blood pressure control among patients receiving integrated care for HIV, diabetes and hypertension in primary healthcare facilities in Tanzania and Uganda

J.B. Birungi ^1^, I. Namakoola^1^, J. Okebe^2^, S. Kivuyo^3^, S. Jaffar^4^

^1^MRC/UVRI & LSHTM, Entebbe, Uganda, ^2^Liverpool School of Tropical Medicine, Liverpool, United Kingdom, ^3^National Institute for Medical Research, Dar es Salaam, Tanzania, United Republic of, ^4^University College of London, London, United Kingdom

**Background**: Non‐communicable diseases (NCDs) are a growing cause of morbidity and mortality among persons with or without HIV in sub‐Saharan Africa. Diabetes and hypertension alone are estimated to cause about 2 million deaths annually. This dual burden requires that healthcare delivery systems are re‐organized to address the needs of patients with chronic conditions for better outcomes. In partnership with public health services, we conducted a large cluster‐randomized trial; INTE‐Africa and compared blood pressure control among patients between the two arms; integrated management and standard care.

**Methods**: The study was part of a large cluster‐randomized control trial. High‐volume primary care facilities were randomized. Study participants were selected systematically. In the integrated care clinic (intervention arm), participants with either HIV, diabetes or hypertension or combinations of these were managed in a single common clinic by the same clinical teams, had joint triage and waiting areas, and shared laboratory, counselling and pharmacy services. Standard care was organized in separate clinics. Participants were followed up for 12 months.

**Results**: A total of 32 facilities were randomized. Sixteen to integrated care and 16 to standard care. We enrolled 7030 participants in the study, of whom 5150 (73.3%) were female. Of these, 3081 (43.8%) had hypertension (either as a single or multiple condition) and had been in care for at least 6 months. At the end of the study, 2440/3081 (79.2%) participants’ blood pressure measurement was recorded, 1190 (48.8%) were in the integrated care arm and 1250 (51.2%) in the standard of care. In our study, 586/1190 (49.2%) participants in integrated care compared to 480/1259 (38.4%) participants in the standard of care arm had blood pressure controlled at the end of the study. There was a difference in blood pressure control among patients with hypertension only, 379/692 (54.8%) in the integrated care arm compared with 288/689 (41.8%) in the standard of care arm of although not statistically significant (*p* value of 0.05). Among patients with diabetes and hypertension, there was a statistically significant difference between the two arms (*p* = 0.01).

**Conclusions**: There was some improvement in blood pressure control among patients in integrated care arm compared to those in the standard of care arm.

### OAE0403 Scaling up cervical cancer prevention services among women living with HIV: lessons and experiences from FIKIA+ project in Mwanza, Tanzania

B. Msongole ^1^, A. Msalilwa^1^, N. Shemndolwa^1^, O. Joseph^1^, M. Mahiti^1^, M. Jalloh^2^, M. Amuri^2^, S. Mrema^3^, D. Magesa^2^, O. Munishi^1^, C. Wells^4^, N. Sugandhi^4^, J. Franks^4^, J. Kahemele^1^, H. Maruyama^1^

^1^ICAP at Columbia University, Dar es Salaam, Tanzania, United Republic of, ^2^U.S Center for Disease Control and Prevention, Dar es Salaam, Tanzania, United Republic of, ^3^Regional Administrative Secretary Office, Mwanza, Tanzania, United Republic of, ^4^ICAP at Columbia University, New York, United States

**Background**: Cervical Cancer Prevention (CECAP) is a priority intervention as part of comprehensive HIV care for women living with HIV (WLHIV). There was low uptake of CECAP services in Tanzania's Mwanza region in the first quarter of fiscal year 2022 (FY22), with 5% achievement of the annual programmatic target of screening 26,618 WLHIV (33% of women on ART) for precancerous cervical lesions. The initial poor performance was due to low service coverage, inadequate supplies, cryotherapy machine breakdown and scale up of 6 multi‐months dispensing (6 MMD) of antiretroviral therapy (ART), which reduced WHLIV's clinic appointment frequency. We describe here progress in scaling up CEPAP services in Mwanza.

**Description**: Between January and March 2022, facilities offering CECAP services were scaled up from 29 to 54. In addition, 97 facilities were added as outreach sites following on‐site mentorship and provision of technical support by trained staff. Services were integrated with community ART refill through a mobile clinic to reach WLHIV who missed services, including those with 6 MMD of ART. Biomedical engineers were engaged to conduct corrective and preventive maintenance of cryotherapy machines and monitoring and timely delivery of CECAP supplies was done. We analysed routine programme data to illustrate trends in cervical cancer screening and treatment and how they proportionally correspond to the pre‐set targets.

**Lessons learned**: WLHIV screened for cervical cancer increased from 1294 in the first quarter of FY22 to 10,188 in the second quarter. By the end of FY22, 25,701 WLHIV were screened, corresponding to 97% of the annual target; 65% of WLHIV were screened at outreach sites. Among WLHIV screened, 753 (3%) were found to have precancerous cervical lesions and 743 (99%) were linked to treatment with 92% completing treatment in the same visit. Out of 25,701 WLHIV screened, 103 (0.4%) were suspected to have cancer and were referred for further diagnosis.

**Conclusions/Next steps**: The target for cervical cancer screening was almost achieved (97%) with 99% linkage to treatment following a combination of enhanced service delivery. The findings demonstrate that it is feasible to make substantial improvements in the uptake of CECAP services by implementing approaches to increase programmatic reach and quality.

### OAE0404 Integrating hepatitis C services into ART clinics in low‐ and middle‐income countries (LMICs) as an approach towards hepatitis C micro‐elimination: pilot experience in Nigeria

C. Agwuocha ^1^, O. Adisa^1^, O. Nwobi^1^, M.‐M. Akanmu^1^, C.E. Boeke^2^, I. Ikpeazu^1^, A. Azania^2^, I. Adamu^3^, A.Y. Ahmed^3^, R. Abade Bello^3^, J. Kama^1^, O. Fernandes^2^, F. Lufadeju^1^, O. Wiwa^1^

^1^Clinton Health Access Initiative, Abuja, Nigeria, ^2^Clinton Health Access Initiative, Boston, United States, ^3^Nasarawa State Ministry of Health, Lafia, Nigeria

**Background**: Globally, an estimated one in 10 people living with HIV (PLHIV) is co‐infected with viral hepatitis B or C (HBV, HCV), which increases morbidity and mortality [1]. HCV can be cured with low‐cost, well‐tolerated direct‐acting antiviral treatment. Improving outcomes among PLHIV with HCV/HBV co‐infection demands strategic service delivery approaches, including integrating HCV/HBV services in antiretroviral therapy (ART) clinics. This abstract highlights HCV/HIV service integration outcomes achieved at four ART sites in Nasarawa State, Nigeria.

[1] https://pubmed.ncbi.nlm.nih.gov/26922272/

**Methods**: A baseline assessment was conducted at ART facilities and community ART engagements including home visits to identify critical points for service integration, including HCV screening, viral load (VL) confirmatory testing and treatment without disruption to existing services. No routine HCV screening for PLHIV was previously available. Relevant healthcare workers were trained on HCV management and data reporting. From July 2020 to December 2022, PLHIV coming for ART visits received HCV screening during triaging and positive patients were linked to care. Patient navigators and ART defaulter trackers identified unscreened PLHIV using facility HCV screening and enrolment data and prompted their return to the facility for HCV services through texts/calls or provided these services in community settings. Positive patients were linked to VL testing and treatment in either the facility or community.

**Results**: During this integration pilot, a total of 3831 PLHIV were screened of 4042 receiving ART across the sites (94.8%; Male (M)/Female (F) = 29%/71%). Four hundred and twenty‐six (11.1%) were seropositive, 371 received a confirmatory HCV VL (87.1%), 218 were viraemic (58.8%) and 175 initiated HCV treatment.

**Figure:**
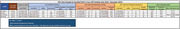
**Abstract OAE0404‐Figure 1**.

**Conclusions**: Despite the effects of the COVID‐19 pandemic, and financing barriers necessitating domestic resource mobilization, HIV/HCV service integration at ART clinics and community settings has been a successful strategy to dramatically expand HCV screening and treatment among HIV clients and a critical step to achieving HCV micro‐elimination in PLHIVs in LMICs.

### OAE0405 Willingness to access PrEP for adolescent girls and young women seeking emergency contraception at community pharmacies in Uganda

T.R. Muwonge ^1^, A. Nalumansi^1^, K. Beima‐Sofie^2^, R. Nsubuga^1^, E. Laker Agnes Odongpiny^1^, M. Nakitende^1^, A. Nakyanzi^1^, A. Mujugira^1,2^, R. Heffron^3^

^1^Infectious Diseases Institute Makerere University, Kampala, Uganda, ^2^University of Washington, Seattle, United States, ^3^University of Alabama, Birmingham, United States

**Background**: Adolescent girls and young women (AGYW) account for 26% of new HIV diagnoses in Uganda, but only 4.6% of pre‐exposure prophylaxis (PrEP) users. Differentiated delivery of PrEP services at private pharmacies, often frequented by this population, could increase prevention coverage.

**Methods**: We recruited AGYW, aged 18–24 years, seeking emergency contraception (EC) from 13 randomly selected community‐pharmacies in central Uganda between May and December 2022. We hypothesized that AGYW seeking EC at community‐pharmacies would be willing to access and use PrEP. Following brief training, pharmacy providers delivered study information to interested AGYW and referred them to the research clinic for data collection. The primary outcome was willingness of AGYW to access PrEP, evaluated using an interviewer‐administered cross‐sectional survey. We used descriptive statistics and modified Poisson regression to analyse the data.

**Results**: We enrolled 130 AGYW, median age of 22 years (interquartile range [IQR] 20–24). Of these, 104 (80%) were living alone or had no partner, and 64 (49%) had no child. Eighty‐four (65%) were employed and median monthly income was $54.4 [40.8–81.6]. AGYW accessed EC a median of three times (IQR 2–6) in the prior 3 months. Most (81%) chose community‐pharmacies as the most convenient place to receive PrEP services instead of public/private health facilities, or community outreach. Approximately half of AGYW (73; 56%) were willing to accept PrEP from community‐pharmacies. Willingness to access pharmacy‐delivered PrEP was associated with PrEP awareness (adjusted prevalence ratio [APR] 1.90; 95% confidence interval [CI]: 1.43–2.51; *p*<0.001), having a busy schedule (APR 1.60; 95% CI: 1.20–2.13; *p* = 0.001), experiencing symptoms of a sexually transmitted disease in the prior 3 months (APR 1.58; 95% CI: 1.22–2.06; *p* = 0.001), number of sexual partners in the previous 3 months (APR 1.15 per partner; 95% CI: 1.02–1.31; *p* = 0.03) and number of sex acts in the prior 3 months (APR 1.01 per act, 95% CI: 1.00–1.02; *p* = 0.006). By contrast, dysuria and condom use were associated with decreased willingness to access PrEP (APR 0.38, 95% CI: 0.20–0.71; *p* = 0.003) and (APR 0.78, 95% CI: 0.67–0.92; *p* = 0.002), respectively.

**Conclusions**: More than half of AGYW in our survey were willing to access PrEP from community‐pharmacies. If scaled, pharmacy‐based PrEP delivery models could significantly improve PrEP access for this population.

### OAE0502 Tackling health facility intersectional stigma faced by men who have same‐gender sex in Ghana: early results from a randomized control trial

L. Nyblade ^1^, E. Oga^1^, S. Clay^2^, M. Chonta^2^, E. Mankattah^3^, R. Vormawor^3^, R.P. Amoh‐Otu^4^, E. Gyamerah^3^, R. Akrong^3^, P. Appiah^4^, F. Boakye^5^, K. Saalim^1^, M.A. Stockton^6^, G. Abu‐Ba'are^7^, N. Smith Ordain^1^, C. Logie^8^, K. Torpey^9^, L.E. Nelson^10^

^1^RTI International, Washington, D.C., United States, ^2^3C Regional Consultants, Lusaka, Zambia, ^3^Educational Assessment & Research Center, Accra, Ghana, ^4^Youth Alliance for Health & Rights, Kumasi, Ghana, ^5^Priorities on Rights & Sexual Health, Accra, Ghana, ^6^Columbia University Irving Medical Center, Department of Psychiatry, New York, United States, ^7^University of Rochester, Rochester, United States, ^8^University of Toronto, Factor‐Inwentash Faculty of Social Work, Toronto, Canada, ^9^University of Ghana, School of Public Health, Department of Population, Family & Reproductive Health, Accra, Ghana, ^10^Yale University, School of Nursing, New Haven, United States

**Background**: Intersectional stigma and discrimination (ISD) in the health system towards gay, bisexual and other men who have sex with men (GBMSM) is a barrier to HIV prevention and treatment for this underserved population, particularly in settings where same‐gender sex is criminalized. Yet, few empirically tested health‐facility (HF) ISD‐reduction interventions exist. Therefore, we adapted an evidence‐based HIV HF stigma‐reduction intervention to address the intersection of HIV, same‐gender sex and gender non‐conforming stigma and discrimination in Ghana, where GBMSM are 11 times more likely to be living with HIV than the general population.

**Methods**: We assessed the effect of the facility‐level stigma reduction intervention using baseline (*n* = 200), 3‐month (*n* = 200) and 6‐month (*n* = 200) follow‐up survey data of staff in eight HFs randomly assigned to intervention and wait‐list control in Greater Accra and Ashanti regions, Ghana. Differences‐in‐differences analysis was conducted to assess whole‐facility intervention effects using the 18‐item and three sub‐scales validated HCF Intersectional Stigma Scale (HCF‐ISS). The outcomes of interest were facility‐level composite stigma scores for key stigma drivers.

**Results**: At 3 months post intervention, we observed a significant reduction in the whole‐facility intersectional stigma score (and the gender‐and‐sexuality norms belief subscale) in the intervention HFs compared to wait‐list facilities. This difference held at 6 months post intervention for the gender‐and‐sexuality norms belief subscale (see Table).

**Table:** 

**Abstract OAE0502‐Table 1**.						
Healthcare Facilities Staff Intersectional Stigma Scale (HCF‐ISS)						
	Control			Intervention		
	Mean (SD) Stigma Scores			Mean (SD) Stigma Scores		
Scale is average score—range 1–5, with 5 highest corresponding to highest level of stigma (undesirable outcome)	Baseline	3‐month	6‐month	Baseline	3‐month	6‐month
Subscale 1: Intersectional Identities Discomfort (Cronbach's alpha = 0.71)	2.02 (0.69)	2.09 (0.74)	1.90 (0.57)	2.12 (0.74)	1.94 (0.67)	1.88 (0.73)
Subscale 2: Gender and Sexuality Norms Beliefs (Cronbach's alpha = 0.72)	3.67 (0.63)	3.55 (0.81)	3.52 (0.76)	3.88 (0.71)	3.43 (0.88)^a^	3.38 (0.87)^a^
Subscale 3: HIV stigma (Cronbach's alpha = 0.68)	2.23 (0.73)	2.22 (0.67)	2.23 (0.68)	2.27 (0.76)	2.24 (0.75)	2.22 (0.68)
Total Intersectional Stigma Score (Cronbach's alpha = 0.74)	2.72 (0.45)	2.7 (0.51)	2.61 (0.45)	2.85 (0.49)	2.58 (0.55)^a^	2.56 (0.58)

^a^ Statistically significant change in average facility stigma score in intervention hospitals compared to control hospitals.

**Conclusions**: Upholding human rights, delivering quality services for GBMSM and ending AIDS by 2030 is not achievable without reducing the ISD faced by populations that are disproportionately impacted by HIV and underserved by the health system. Reducing ISD in HFs is particularly critical, given their essential role in providing prevention and treatment services. This study provides evidence that it is possible to reduce intersectional stigma towards GBMSM in health systems, even in the context of challenging political and social climates. It provides a replicable intervention approach as well as practical intervention tools.

### OAE0503 Catalysing access to HIV services and essential healthcare for key populations: effective innovations for African countries

F. Ilika ^1^, U. Ihekanandu^1^

^1^Palladium, Abuja, Nigeria

**Background**: About four‐fifths of the 24 million people in Nigeria's Lagos State live below the poverty line and cannot afford to pay for essential healthcare without risking catastrophic financial consequences. A large number of key population groups—men who have sex with men, injection drug users, female sex workers and transgender populations reside in Lagos state. Nigeria, like many African counties, often criminalize and stigmatize these population groups with resultant lack of access to essential healthcare, risk of health catastrophe and poverty as traditional government programmes do not fund their healthcare needs.

**Description**: The USAID Health Policy Plus Project HP+ supported Lagos State to expand its health insurance scheme and devised a special focus to target orphans, vulnerable children, people living with HIV and key populations. HP+ collaborated with key actors including the Network of People living with HIV and State Health Insurance Agency and secured the release of the state's equity fund—an annual contribution by the government for provision of healthcare to poor and vulnerable communities—and for key populations to be prioritized as beneficiaries of this fund, for the first time. The programme also facilitated their enrolment into the social register which enables them to access government intervention funds for improving livelihoods.

**Lessons learned**: These efforts led to the release of NGN750‐million in equity funds, for increased access to quality healthcare, and HIV services. In total, 100,000 poor and vulnerable children and people living with HIV, including 12,000 from key populations, were enrolled. This policy change allowed the targeted groups to be enrolled into health insurance and receive free healthcare at their preferred one stop shop facilities, increasing access to essential HIV and healthcare services, and financial protection.

**Conclusions/Next steps**: Catalysing government funding for key populations is possible even in settings where they are criminalized through strategic, targeted, collaborative and integrated approaches. This can be adapted by other countries facing similar challenges in obtaining government funding for key populations. This integrated approach also provides health, economic and social benefits, which is essential for ensuring the complete wellbeing of key population groups—a critical factor for HIV epidemiological control.

### OAE0504 Increasing access to SelfCare: employing an online‐based demand generation strategy to increase uptake of peer‐led unassisted HIV self‐testing among key populations in the Philippines

R.M. Briñes ^1^, R. Domingo^1^, J.D. Rosadiño^1^, D. Cruz^1^, M. Brines^1^, P. Junio^1^, J.l. Dinglasan^1^, R. Pagtakhan^1^, J.P. Benito^1^

^1^LoveYourself Inc., Mandaluyong, Philippines

**Background**: Ease of access is one factor in getting tested for HIV. The imposed limited mobility due to COVID‐19 limited this access even further. Since its introduction in 2020, unassisted HIV self‐testing has been established as a choice for key populations to access HIV testing in the Philippines. Aiming to bring HIV testing awareness to key populations, a programme designed to generate demand is introduced in SelfCare (LoveYourself's unassisted HIV self‐testing programme).

**Description**: Guided by the AIDA model (Figure 1), this programme aimed to create awareness and demand generation by creating a massive number of leads of potential clients accessing SelfCare. A communications plan was designed by members of key populations to determine the campaign architecture, keeping in mind the target market: gay, bisexual and other men who have sex with men (GBMSM) and transgender people. The key messages focused on testing information, access and its impact on the lifestyle of clients. The visual theme and the messages developed include a motivational tone of espousing self‐empowerment. These campaigns are promoted on various social media platforms.

**Figure:**
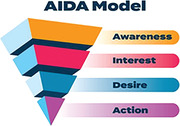
**Abstract OAE0504‐Figure 1**.

**Lessons learned**: The communications plan was implemented starting in October 2020. The continuous posting of content has increased uptake by 1012% compared to data from July to September 2020. During the campaign, a total of 513,024 clients were reached (as of December 2022). These campaigns have generated 20,043 clients accessing SelfCare, with a reactivity rate of 6% among those who reported results. 39.13% of the clients tested for HIV for the first time. Among those reactive, 75.33% of clients have been enrolled in treatment.

**Conclusions/Next steps**: It was seen that demand generation campaigns powered by the community are effective in bringing awareness of HIV self‐testing. Community consultations are essential in creating these programmes for key communities. This momentum will be maintained with offline community engagement activities and campaigns promoted on other online platforms.

### OAE0505 Differentiated online‐to‐offline (O2O) interventions for HIV services: impacts on HIV testing and case finding among key populations in Thailand, Nepal and the Philippines

P. Patpeerapong ^1^, T. Chaisalee^2^, S. Janyam^3^, S. Sittikarn^4^, Y.R. Sapkota^5^, A. Sharma^5^, D. Ortiz^6^, G.‐J. Alatiit^7^, S. Mills^8^, A. Arunmanakul

^1^Mplus Foundation, Chiang Mai, Thailand, ^2^Rainbow Sky Association of Thailand, Bangkok, Thailand, ^3^Service Workers in Group Foundation, Bangkok, Thailand, ^4^Caremat Foundation, Chiang Mai, Thailand, ^5^FHI 360, Kathmandu, Nepal, ^6^Laguna Medical Center, Laguna, Philippines, ^7^Hospital Ospital ng Biñan, Laguna, Philippines, ^8^FHI 360, Bangkok, Thailand

**Background**: The COVID‐19 pandemic brought renewed emphasis on the strategic use of online to offline (O2O) interventions, since in‐person outreach was reduced, and HIV service options were often limited. In Thailand, Nepal and the Philippines, USAID EpiC Project‐supported community‐based organizations and hospitals adapted multiple O2O approaches to reach and test key populations, with a focus on reaching undiagnosed HIV‐positive individuals. We identify and analyse here the impacts of these O2O approaches in the three countries from October 2021 to September 2022.

**Description**: Social influencers, targeted ads and online outreach engaged sexual, drug‐use, and chemsex networks to promote HIV testing on social media and chat apps, for example Facebook, Twitter, LINE, TikTok, Grinder, Hornet and Blued. A unique online reservation web application permitted in‐depth analysis of client flow from the source of client online exposure to messaging through clinic attendance and service utilization, for example HIV testing.

**Lessons learned**: In Thailand, O2O interventions brought in 11% of all project‐supported HIV testing clients (5% HIV case detection). A majority of online clients were reached by Facebook, but two apps—Blued and Twitter—had the highest case detection (10.4% and 7.8%, respectively). In Nepal, O2O interventions brought in 10.3% of all HIV testing clients, and Facebook was the most common site for recruiting clients (57% of all O2O activity). A total of 8.4% of clients tested through O2O interventions were positive, though some platforms showed higher case detection, for example WhatsApp, 11.1%. In the Philippines, O2O interventions specifically assisted project‐supported government hospitals where they contributed more than one‐third (37%) of the clients HIV tested and almost one‐fifth (19%) of all identified HIV cases. At one government facility, the online reservation app contributed 56% of all HIV testing and 79% of all identified HIV cases.

**Conclusions/Next steps**: Differentiated O2O interventions focused on key populations clearly demonstrated their added value in HIV testing and case finding across three countries. Programmes need to regularly analyse the effectiveness of specific platforms, use innovations, such as online reservation apps that allow more in‐depth analysis, and continually adapt O2O interventions so that they remain impactful and relevant to KP's dynamic use of social media.

## LATE BREAKING ABSTRACTS

### OALBA0502 CD8 T cell counts negatively correlate with IL‐15‐mediated HIV reactivation **ex vivo**

J. Howard ^1^, C. Levinger^1^, R. Fromentin^2^, N. Chomont^2^, A. Bosque^1^

^1^George Washington University, Microbiology, Immunology, and Tropical Medicine, Washington, United States, ^2^Université de Montréal, Centre de recherche du CHUM, Montreal, Canada

**Background: **The latent HIV reservoir remains the largest barrier to cure despite effective antiretroviral therapy (ART). Most assays measuring viral reactivation rely on stimulation of cells ex vivo with latency reversing agents (LRA) and quantification of viral RNA. In this work we aimed to evaluate the ability of IL‐15 to reactivate the translation‐competent viral reservoir in CD4 T cells isolated from ART‐suppressed people with HIV (PWH).

**Methods: **We optimized a novel assay to evaluate TRAnslationally CompEtent viral Reservoirs (TRACER Assay) using a planar array ultrasensitive p24 Gag ELISA. We have previously shown that this assay can detect viral p24 at fg/ml in both supernatants and cell lysates (Levinger et al., Scientific Reports, 2021). Using this assay, we evaluated the ability of IL‐15 and aCD3/aCD28 to reactivate the translation‐competent viral reservoir in CD4T cells from 12 ART‐suppressed participants and correlated viral reactivation with different markers of HIV persistence and other clinical characteristics.

**Results: **IL‐15 and aCD3/aCD28 both reactivated translationally competent virus in 9 out of 12 participants. In responders, viral reactivation by IL‐15 when compared to unstimulated was an average of 14‐fold (3.4‐37.3) in supernatants (p = 0.0039) and 11‐fold (0.83‐84.4) in lysates (p = 0.0078). Viral reactivation by aCD3/aCD28 was an average of 387‐fold (1.05‐724) in supernatants (p = 0.0039) and 302‐fold (0.51‐1758.3) in lysates (p = 0.0078). Levels of p24 induced by IL‐15 and aCD3/aCD28 were positively correlated between supernatants and lysates (r = 0.79, p = 0.048; r = 0.69 p = 0.017, respectively). No correlation was observed between reactivation of translationally competent virus by both stimuli and markers of persistence including total HIV DNA, integrated HIV DNA, TILDA, or HIV flow. Interestingly, we observed a strong negative correlation between absolute CD8 counts and the ability of IL‐15 to reactivate latent HIV (r = ‐0.78 p = 0.0043). This correlation was also observed with aCD3/aCD28 albeit to a lesser degree (r = ‐0.52 p = 0.089)

**Conclusions: **We have developed a new assay to measure reactivation of translationally competent virus ex vivo from ART‐suppressed PWH and have identified CD8 absolute levels as a potential response marker to the LRA activity of IL‐15.

### OALBA0503 Venetoclax, alone and in combination with the BH3‐mimetic S63845, depletes HIV‐1 latently infected cells and delays rebound in humanized mice

P. Arandjelovic ^1^, Y. Kim^2^, J. Cooney^1^, S. Preston^1^, M. Doerflinger^1^, J. McMahon^3^, S. Garner^1^, J. Zerbato^2^, M. Roche^2,4^, C. Tumpach^2^, J. Ong^2^, D. Sheerin^1^, G. Smyth^1,5^, J. Anderson^2^, C. Allison^1^, S. Lewin^2,6^, M. Pellegrini^1^

^1^The Walter and Eliza Hall Institute of Medical Research, Department of Medical Biology, Melbourne, Australia, ^2^The University of Melbourne at the Peter Doherty Institute for Infection and Immunity, Department of Infectious Diseases, Melbourne, Australia, ^3^Alfred Hospital and Monash University, Department of Infectious Diseases, Melbourne, Australia, ^4^Emerging Infections Program, RMIT University, School of Health and Biomedical Sciences, Melbourne, Australia, ^5^The University of Melbourne, School of Mathematics and Statistics, Melbourne, Australia, ^6^Royal Melbourne Hospital at the Peter Doherty Institute for Infection and Immunity, Victorian Infectious Diseases Service, Melbourne, Australia

**Background: **HIV‐1 persists indefinitely in people living with HIV (PLWH) on antiretroviral therapy (ART) as a long‐lived viral reservoir. Recent evidence suggests that the viral reservoir is resistant to cell death as a result of up‐regulation of anti‐apoptotic molecules including B‐cell lymphoma (BCL)‐2. BH3‐mimetics, such as venetoclax and other compounds, are small‐molecule therapeutics which lower the threshold for induction of intrinsic apoptosis by antagonising the function of Bcl‐2 family pro‐survival proteins.

**Methods: **We employed a humanized mouse model of latent HIV‐1 infection, as well as CD4^+^ T cells from PLWH on ART collected by leukapharesis, to investigate whether antagonising host pro‐survival proteins with clinically‐relevant BH3‐mimetics can preferentially prime latent cells to die and facilitate clearance of the viral reservoir. We quantified the time to viral rebound in humanized mice following cessation of ART and changes in the viral reservoir using either integrated DNA or the intact proviral DNA assay (IPDA). We performed RNA sequencing on venetoclax‐treated CD4^+^ T cells from PLWH on ART.

**Results: **Venetoclax, a clinically‐approved inhibitor of Bcl‐2, depleted total and intact HIV‐1 DNA in CD4^+^ T cells from PLWH treated ex vivo in a dose‐dependent manner (mean percentage decrease in intact DNA was 41.8% with 100 nM Venetoclax). Venetoclax induced higher rates of death in naïve and central memory T‐cells, compared to other T‐cell subsets. RNA‐Seq analysis revealed that following venetoclax treatment ex vivo, cells with higher expression of transcripts of pro‐apoptotic BH3‐only proteins were over‐represented. Venetoclax (dosed every weekday for 6 weeks) significantly delayed viral rebound following cessation of ART in a humanized mouse model of HIV‐1 infection. The combination of venetoclax (dosed every weekday for 3 weeks) with the Mcl‐1 inhibitor S63845 achieved a longer delay in viral rebound compared to either intervention alone (median time to viral rebound was 3 weeks).

**Conclusions: **Selective inhibition of pro‐survival proteins, including BCL‐2 and MCL‐1, can induce elimination of the viral reservoir and prolong time to viral rebound after cessation of ART in a mouse model. Given the well‐established dosage and safety profile of venetoclax in humans as a licensed drug, rapid translation to a human clinical trial of venetoclax is warranted.

### OALBA0504 Absence of viral rebound for 18 months without antiretrovirals after allogeneic hematopoietic stem cell transplantation with wild‐type CCR5 donor cells to treat a biphenotypic sarcoma

A. Sáez‐Cirión ^1^, A.‐C. Mamez^2^, V. Avettand‐Fenoel^3^, P. Thoueille^4^, M. Nabergoj^5^, M. Hentzien^6^, E. Mereles Costa^6^, M. Salgado^7^, M. Nijhuis^8^, A. Melard^3^, E. Gardiennet^3^, V. Monceaux^1^, C. Passaes^1^, A. Chapel^1^, F. Perdomo‐Celis^1^, A. Wensing^8^, J. Martínez‐Picado^7^, S. Yerly^9^, M. Rougemont^10^, A. Calmy^6^, ICISTEM study group

^1^Institut Pasteur, Viral Reservoirs and Immune Control Unit, Paris, France, ^2^Geneva University Hospitals, Department of Oncology, Geneva, Switzerland, ^3^Institut Cochin, CNRS 8104 / INSERM U1016 / Université de Paris, Paris, France, ^4^Lausanne University Hospital, Laboratory of Clinical Pharmacology, Lausanne, Switzerland, ^5^Institut Central des Hôpitaux, Sion, Switzerland, ^6^Geneva University Hospitals, HIV/AIDS Unit, Geneva, Switzerland, ^7^AIDS Research Institute, IrsiCaixa, Badalona, Spain, ^8^University Medical Center Utrecht, Utrecht, Netherlands, ^9^Geneva University Hospitals, Laboratory of Virology, Geneva, Switzerland, ^10^Private practitioner, Geneva, Switzerland

**Background: **Durable HIV‐1 remission after antiretroviral treatment (ART) discontinuation has been reported for 5 individuals receiving allogeneic hematopoietic stem cell transplant (aHSCT) from CCR5Δ32 homozygous donors. We report here a Caucasian male (Icistem‐34), diagnosed with HIV‐1 in 1990 and on continuous suppressive‐ART since 2005. In 2018, he received chemotherapy followed by aHSCT from an unrelated HLA‐matched (9/10) wild‐type CCR5 donor to treat a biphenotypic sarcoma. ART was discontinued in November 2021. His viral load has remained undetectable for 18 months so far.

**Methods: **Samples, pre‐aHSCT, pre and/or post treatment interruption (TI), were analyzed for HIV RNA, HIV DNA, antiretrovirals, HIV‐1 antibodies, NK and T cells phenotype, and HIV/CMV T‐cell responses. Intact proviral DNA analyses (IPDA), tests of viral production by purified CD4+ T cells and their susceptibility to HIV were performed post‐aHSCT.

**Results: **Ultrasensitive HIV RNA (4 copies/ml) and HIV DNA (457 and 1096 copies/million CD4 cells in blood and bone marrow) were detected before aHSCT. The virus was predicted R5. Full chimerism was achieved within a month post‐aHSCT. Acute hepatic graft vs host diseases (GVHD) occurred soon after aHSCT, and was treated with corticosteroid/calcineurin inhibitor. Chronic hepatic GVHD occurred 8m after aHSCT and was treated with ruxolitinib, which was transiently discontinued but had to be resumed due to GVHD relapse. Standard plasma viremia remained undetectable after aHSCT, ultrasensitive RNA dropped to undetectable values. Proviral DNA also decreased significantly, despite low levels (4 to 40 copies/million cells) being detected sporadically post‐aHSCT, including defective but not intact HIV DNA by IPDA. No virus was amplified from in vitro stimulated CD4+ T cells post‐TI. Cells remained susceptible to HIV‐1 in vitro. ART levels were undetectable post‐TI except coinciding with two episodes of event‐driven “PreP” (4 pills) at M2 and M12 post‐TI. HIV‐1 antibodies slightly declined since aHSCT. No HIV‐specific T cell responses were detected post‐TI.

**Conclusions: **We report an individual with HIV‐1 who at 18m post‐TI, 57m post‐aHSCT with cells from a wild‐type CCR5 donor, has no evidence of HIV‐1 RNA rebound or replicating virus. These results suggest that HIV remission could be achieved in some cases in the context of aHSCT with wild‐type CCR5.

### OALBA0505 Anti‐SIV Env RhmAbs +/‐ CD8a depletion and N‐803 in ART‐suppressed rhesus macaques leads to post‐treatment control of viremia in a subset of animals

V. Singh ^1^, D. Burgess^1^, A. Dashti^1^, J. McBrien^2^, H. King^3^, R. Mason^3^, J. Safrit^4^, J. Lifson^5^, M. Tuyishime^6^, G. Ferrari^6^, M. Roederer^3^, G. Silvestri^2^, A. Chahroudi^1^

^1^Emory University, Pediatric Infectious Disease, Atlanta, United States, ^2^Emory University, Emory National Primate Research Center, Atlanta, United States, ^3^National Institute of Allergy and Infectious Diseases (NIAID), National Institutes of Health (NIH), Vaccine Research Center, Bethesda, United States, ^4^ImmunityBio, Culver City, United States, ^5^Frederick National Laboratory, AIDS and Cancer Virus Program, Frederick, United States, ^6^Duke School of Medicine, Department of Medicine, Durham, United States

**Background: **Building upon robust latency reversal seen after CD8a‐depletion and IL‐15 superagonist in SIV‐infected, ART‐suppressed rhesus macaques (RMs), here we combined these agents with four anti‐SIV Env‐specific rhesus IgG_1_ monoclonal antibodies (RhmAbs) with the goal to reduce reservoirs and/or modulate viral rebound dynamics after ART interruption.

**Figure:**
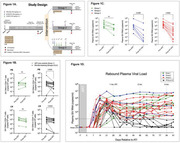
**Abstract OALBA0505‐Figure**.

**Methods: **28 RMs were infected with SIV_mac239_; ART was initiated 8 weeks post‐infection. Groups were assigned after 96‐weeks on‐ART: Group 1 (n = 7): ART‐only; Group 2 (n = 7): ART+RhmAbs; Group 3 (n = 14): ART+RhmAbs+CD8a‐depletion+N‐803 (Fig 1A). Analytical treatment interruption (ATI) of ART was initiated ∼6 months post‐2nd RhmAbs dose, when levels had declined below 1 mcg/ml in most RMs.

**Results: **Latency reversal (defined as on‐ART viremia >60 copies/ml) was achieved in 11/14 Group 3 RMs versus 0/14 Group 1+2 RMs. Prior to ATI, all groups had similar levels of SIV‐DNA in CD4+ T‐cells from blood, lymph nodes and rectum; however, significant reduction from pre‐intervention was seen only in RhmAb‐receiving RMs (Fig 1B). By ATI day 21, all RMs rebounded to >60 copies/ml with no intergroup difference in time‐to‐rebound. Intragroup rebound setpoints 3 months post‐ATI were significantly lower than pre‐ART setpoints in Group 2 (p = 0.03) and Group 3 (p<0.001), but not Group 1 (Fig 1C). Post‐treatment viral control (PTC, defined as ≥3 consecutive viral loads <10^3^ copies/ml) off‐ART was observed in 4/7 Group 2 and 3/14 Group 3 RMs (Fig1D). No Group 1 RMs exhibited PTC. PTCers had lower peak viremia (p = 0.02) and pre‐ART viremia (p = 0.01) versus non‐PTCers, but similar levels of infected cells post‐intervention. PTC was not associated with RhmAb concentrations.

**Conclusions: **Time‐to‐viral‐rebound was not impacted by MT807R1+N‐803+RhmAbs despite robust latency reversal and evidence of treatment response on infected cell level. PTC was observed only in RhmAb‐receiving animals. Lower pre‐ART viral loads in PTCers suggest SIV‐RhmAbs may boost endogenous immune responses, giving rise to observed control of viremia off‐ART.

### OALBB0502 A prospective, randomized trial to assess a protease inhibitor–based regimen switch strategy to manage integrase inhibitor–related weight gain

W.R. Short ^1^, M. Ramgopal^2^, D.P. Hagins^3^, J. Lee^4^, R.B. Simonson^4^, T.‐H. Hsu^4^, P. Xu^5^, D. Anderson^4^

^1^Perelman School of Medicine, University of Pennsylvania, Philadelphia, United States, ^2^Midway Immunology and Research Center, Fort Pierce, United States, ^3^Chatham CARE Center, Savannah, United States, ^4^Janssen Scientific Affairs, LLC, Titusville, United States, ^5^Janssen Research & Development, LLC, Titusville, United States

**Background: **Integrase inhibitor (INI)‐based antiretroviral (ARV) therapies are associated with greater weight gain than non‐nucleoside reverse transcriptase inhibitor‐ or boosted protease inhibitor‐based regimens, disproportionately affecting Black and Hispanic individuals and women. There are no prospective, randomized data exploring the impact of switching ARV classes to mitigate or reverse ARV‐related weight gain.

**Methods: **DEFINE (ClinicalTrials.gov: NCT04442737) is a randomized (1:1), prospective, 48‐week, active‐controlled, open‐label, multicenter phase 4 study evaluating switching to darunavir/cobicistat/emtricitabine/tenofovir alafenamide (D/C/F/TAF) versus continuing INI+TAF/emtricitabine (FTC) in virologically‐suppressed HIV‐1‐infected adults who had ≥10% weight gain while on the INI‐based regimen. The primary objective was to assess percent change in body weight from baseline to Week 24 in both arms. Data through Week 24 are reported.

**Results: **Overall, 103 adults were randomized to D/C/F/TAF (n = 53) or continued INI+TAF/FTC (n = 50); 30% were female and 61% were Black/African American (**Table**). At Week 24, there was no significant difference in percent change in body weight from baseline between the D/C/F/TAF and INI+TAF/FTC arms (**Figure 1A**). Most participants in each arm had body weight changes of ≤±3% and remained within baseline body mass index and waist circumference categories. Percent body weight changes for key subgroups are shown in **Figure 1B**. Switching to D/C/F/TAF was safe and well tolerated, and efficacy was maintained.

**Figure:**
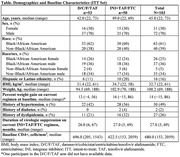
**Abstract OALBB0502‐Table**.

**Figure:**
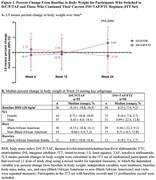
**Abstract OALBB0502‐Figure**.

**Conclusions: **There was no significant difference in weight change through 24 weeks after switching from an INI‐based regimen to D/C/F/TAF in adults with INI‐related weight gain. Additional analyses are ongoing, including follow up through Week 48 and evaluation of changes in biomarkers and body composition (DEXA).

### OALBB0503 Increasing second‐line antiretroviral therapy options for children with HIV in Africa: week‐96 efficacy and safety results of the CHAPAS‐4 randomised trial

V. Musiime ^1,2^, A.J. Szubert^3^, M. Bwakura Dangarembizi^4^, C. Chabala^5^, K. Doerholt^3^, N. Dukakia^3^, C. Kityo Mutuluza^1^, A. Lugemwa^6^, J. Lungu^5^, S. Makumbi^6^, H. Mcllleron^7^, H.A. Mujuru^4^, V. Mulenga^5^, G. Musoro^4^, M. Mwamabazi^8^, E. Nambi^1^, R. Nazzinda^1^, W. Ndebele^9^, B. Nduna^8^, C. Shakeshaft^3^, G. Siziba^9^, S. Tafeni^4^, A. Turkova^3^, A.S. Walker^3^, D.M. Gibb^3^

^1^Joint Clinical Research Centre, Kampala, Uganda, ^2^Makerere University, Kampala, Uganda, ^3^Medical Research Council Clinical Trials Unit at University College London, London, United Kingdom, ^4^University of Zimbabwe Clinical Research Centre, Harare, Zimbabwe, ^5^University Teaching Hospital, Lusaka, Zambia, ^6^Joint Clinical Research Centre, Mbarara, Uganda, ^7^University of Cape Town, Cape Town, South Africa, ^8^Arthur Davison Children's Hospital, Ndola, Zambia, ^9^Mpilo Central Hospital, Bulawayo, Zimbabwe

**Background: **There are limited options for second‐line antiretroviral therapy(ART) for children with HIV. CHAPAS‐4(ISRCTN22964075) evaluated long‐term outcomes for children starting second‐line ART.

**Methods: **In this 2 × 4 factorial trial, children from Uganda, Zambia and Zimbabwe were randomised to second‐line tenofovir alafenamide/emtricitabine(TAF/FTC) or standard‐of‐care(SOC) backbone (abacavir(ABC) or zidovudine(AZT) with lamivudine(3TC))(randomisation 1) and to one of four anchor drugs: dolutegravir(DTG) or ritonavir‐boosted darunavir(DRV/r), atazanavir(ATV/r) or lopinavir(LPV/r) (randomisation 2). Primary endpoint was viral load(VL)<400copies/mL at week‐96. We hypothesised that TAF/FTC would be non‐inferior to SOC(10% margin); ATV/r non‐inferior to LPV/r(12% margin); DRV/r and DTG superior to LPV/r and ATV/r arms combined(superiority threshold p≤0.03; as multiple comparisons). Analysis was intention‐to‐treat, based on logistic regression.

**Results: **919 children aged 3–15years (54%male, median[IQR] viral load 17,573copies/mL[5549, 55,700]; CD4 count 669[413, 971]) switching NNRTI‐based ART, were randomised and spent 98% of time on allocated regimen. At week‐96, 406/454(89.4%) on TAF/FTC vs 378/454(83.3%) on SOC had VL<400copies/mL (no evidence of difference between ABC and ZDV arms). For randomisation 2, 208/226(92.0%) on DTG, 203/230(88.3%) on DRV/r, 193/229(84.3%) on ATV/r, 180/223(80.7%) on LPV/r had VL<400c/ml. TAF/FTC was superior to SOC; DTG was superior to LPV/r and ATV/r; DRV/r showed a trend to superiority to LPV/r and ATV/r; ATV/r was non‐inferior to LPV/r (Table). Results were similar for VL<60copies/mL and <1000copies/mL and at weeks 48 and 144. CD4 count improved in all arms. More grade 3/4 adverse events (AE), predominately hyperbilirubinemia, occurred ATV/r vs LPV/r(p<0.0001); DTG had fewer AE vs LPV/r(p = 0.02). There was no evidence of excess weight‐gain with DTG±TAF. Improvement in growth parameters were greater with TAF vs SOC; and with DTG, DRV/r and ATV/r vs LPV/r. Renal and bone health was similar between arms. One child died (treatment‐unrelated); 3% had serious adverse events.

**Table:** 

**Abstract OALBB0503‐Table 1. Week 96 VL comparisons**				
	<400c/ml difference (%) [95% CI]	p‐value	<60c/ml: difference (%) [95% CI]	p‐value
TAF vs SOC	6.3 [2.0, 10.6]	0.004	6.3 [1.0, 11.5]	0.02
ATV/r vs LPV/r	3.4 [‐3.4, 10.2]	0.33	5.4 [‐2.5, 13.2]	0.18
DRV/r vs LPV/r+ATV/r	5.6 [0.3, 11.0]	0.04	3.1 [‐3.5, 9.8]	0.35
DTG vs LPV/r+ATV/r	9.7 [4.8, 14.5]	<0.0001	10.5 [4.4, 16.6]	0.0007

**Conclusions: **TAF/FTC and DTG were virologically superior to SOC backbone and comparators(ATV/r, LPV/r) respectively, with excellent safety profiles. Child‐friendly fixed‐dose combinations of TAF/FTC(±DTG or boosted DRV or ATV) would increase access to safe, effective second‐line ART options for children.

### OALBB0504 Risks of hypertension with first‐line dolutegravir (DTG) and tenofovir alafenamide (TAF) in the NAMSAL and ADVANCE trials

F. Venter ^1^, S. Sokhela^1^, B. Bosch^1^, G. Akpomiemie^1^, M. Mirchandani^2^, K. McCann^2^, M. Mpoudi‐Etame^3^, T. Tovar Sanchez^4^, M.‐a.‐Q. Bousmah^5,6^, A. Calmy^7^, E. Delaporte^4,8^, C. Kouanfack^9,10,11,12^, A. Hill^13^

^1^Ezintsha, University of the Witwatersrand, Faculty of Health Sciences, Johannesburg, South Africa, ^2^Imperial College London, Faculty of Medicine, London, United Kingdom, ^3^Military Hospital Region N°1, Yaoundé, Cameroon, ^4^University of Montpellier, TransVIHMI, Montpellier, France, ^5^SESSTIM: University of Aix Marseille, Inserm, Marseille, France, ^6^Université Paris Cité, Inserm, Ceped, Paris, France, ^7^Genva University Hospitals, Division of Infectious Diseases, HIV‐AIDS Unit, Geneva, Switzerland, ^8^Montpellier University Hospital Center, Montpellier, France, ^9^University of Dschang, Faculty of Medicine and Pharmaceutical Sciences, Dschang, Cameroon, ^10^Yaoundé Central Hospital, Yaoundé, Cameroon, ^11^Yaoundé Central Hospital, Cameroon ANRS‐site, Yaoundé, Cameroon, ^12^CREMER, Yaoundé, Cameroon, ^13^University of Liverpool, Department of Pharmacology and Therapeutics, Liverpool, United Kingdom

**Background: **Hypertension is a leading cause of death in sub‐Saharan Africa, with a high background prevalence in the general population. First‐line use of TAF and DTG lead to higher risks of clinical obesity than tenofovir disoproxil fumarate (TDF) or efavirenz (EFV). Clinical obesity increases the risks of hypertension and other non‐communicable diseases (NCDs).

**Methods: **In the NAMSAL trial, 613 PLWH in Cameroon were randomised to TDF/3TC/DTG or TDF/3TC/EFV (EFV low‐dose). In the ADVANCE trial, 1053 PLWH in South Africa were randomised to TAF/FTC/DTG, TDF/FTC/DTG or TDF/FTC/EFV. In both trials, blood pressure was measured at every study visit. Grade 1 hypertension was defined as SBP/DBP >140/90 mmHg. In ADVANCE, all participants developing Grade 1 hypertension were given antihypertensives. In NAMSAL, <1% of participants were given anti‐hypertensives, as funding was not available.

**Results: **In NAMSAL <1% of participants were treated with anti‐hypertensive drugs. By Week 192, 31% of participants developed Grade 1 hypertension on TDF/FTC/DTG, versus 19% on TDF/3TC/EFV (p = 0.002). In multivariate analysis, Grade 1 hypertension was significantly correlated with use of DTG, age, sex and BMI (p<0.01 for each comparison). In ADVANCE, 6% of participants were already being treated for hypertension at baseline, rising to 20% by Week 192. Treatment‐emergent Grade 1 hypertension was diagnosed for 42/315 (13%) participants on TAF/FTC/DTG, 33/316 (10%) on TDF/FTC/DTG, and 25/314 (8%) taking TDF/FTC/EFV. The risk of Grade 1 hypertension was significantly higher for TAF/FTC/DTG versus TDF/FTC/EFV (p = 0.04). However, 94% of participants developing hypertension were given anti‐hypertensives. By Week 192, there was no significant difference in mean SBP or Grade 1 hypertension between the arms.

**Conclusions: **In the NAMSAL and ADVANCE trials, first‐line use of DTG was associated with significantly higher risks of treatment‐emergent hypertension, especially when combined with TAF. In NAMSAL, where hypertension was not consistently treated, risks of hypertension remained higher for TDF/3TC/DTG through Week 192. However in ADVANCE, most cases of hypertension were successfully treated, and there was no significant difference between treatment arms by Week 192. Hypertension can be diagnosed and treated with low‐cost generic drugs. Mass HIV treatment programmes need to include support and funding for diagnosis and treatment for hypertension and other NCDs.

### OALBB0505 Impact of INSTI and TAF‐related BMI changes and risk on hypertension and dyslipidemia in RESPOND

D.M. Byonanebye ^1,2^, M.N. Polizzotto^3^, F. Maltez^4^, A. Rauch^5^, K. Grabmeier‐Pfistershammer^6^, F. Wit^7^, S. De Wit^8^, A. Castagna^9^, A. D'ARMINIO MONFORTE^10^, C. Mussini^11^, J.‐C. Wasmuth^12^, E. Fontas^13^, I. Abela^14,15^, M. Sarcletti^16^, L. Bansi‐Matharu^17^, N. Jaschinski^18^, L. Peters^18^, S.R Hosein^19^, V. Vannappagari^20^, C. Cohen^21^, E. Bissio^22^, A. Mocroft^18,17^, M. Law^23^, L. Ryom^18^, K. Petoumenos^23^, on behalf of the RESPOND study group

^1^The Kirby Institute, Biostatistics and Databases Program, Sydney, Australia, ^2^Makerere University, School of Public Health, Kampala, Uganda, ^3^The Australian National University, Canberra, Australia, ^4^Hospital Curry Cabral, Lisbon, Portugal, ^5^Bern University Hospital, Department of Infectious Diseases, Inselspital, Bern, Switzerland, ^6^Medizinische Universität Wien, Vienna, Austria, ^7^Stichting HIV Monitoring (SHM), Amsterdam, Netherlands, ^8^St Pierre University Hospital Bruxelles, Department of Infectious Diseases, Brussels, Belgium, ^9^Università Vita‐Salute San Raffaele, San Raffaele Scientific Institute, Milano, Italy, ^10^ASST Santi Paolo e Carlo, Milan, Italy, ^11^Università degli Studi di Modena, Modena, Italy, ^12^University Hospital Bonn, Bonn, Germany, ^13^Université Côte d'Azur et Centre Hospitalier Universitaire, Nice HIV cohort, Nice, France, ^14^University of Zurich, Zurich, Switzerland, ^15^University Hospital of Zurich, Zurich, Switzerland, ^16^Medical University Innsbruck, Department of Dermatology, Venerology and Allergology, Innsbruck, Austria, ^17^University College London, Center for Clinical Research, Epidemiology, Modelling and Evaluation (CREME), Institute for Global Health, London, United Kingdom, ^18^CHIP, Rigshospitalet, University of Copenhagen, Copenhagen, Denmark, ^19^European AIDS Treatment Group, Brussels, Belgium, ^20^ViiV Healthcare, Research Triangle Park, United States, ^21^Gilead Sciences, Foster City, United States, ^22^MSD, Rahway, United States, ^23^Kirby Institute, Biostatistics and Databases Program, Sydney, Australia

**Background: **We determined whether change in body mass index (BMI) differentially increases the risk of hypertension or dyslipidaemia in people with HIV (PLWH) receiving integrase inhibitors (INSTI) and/or tenofovir alafenamide (TAF) compared to other contemporary regimens.

**Figure:**
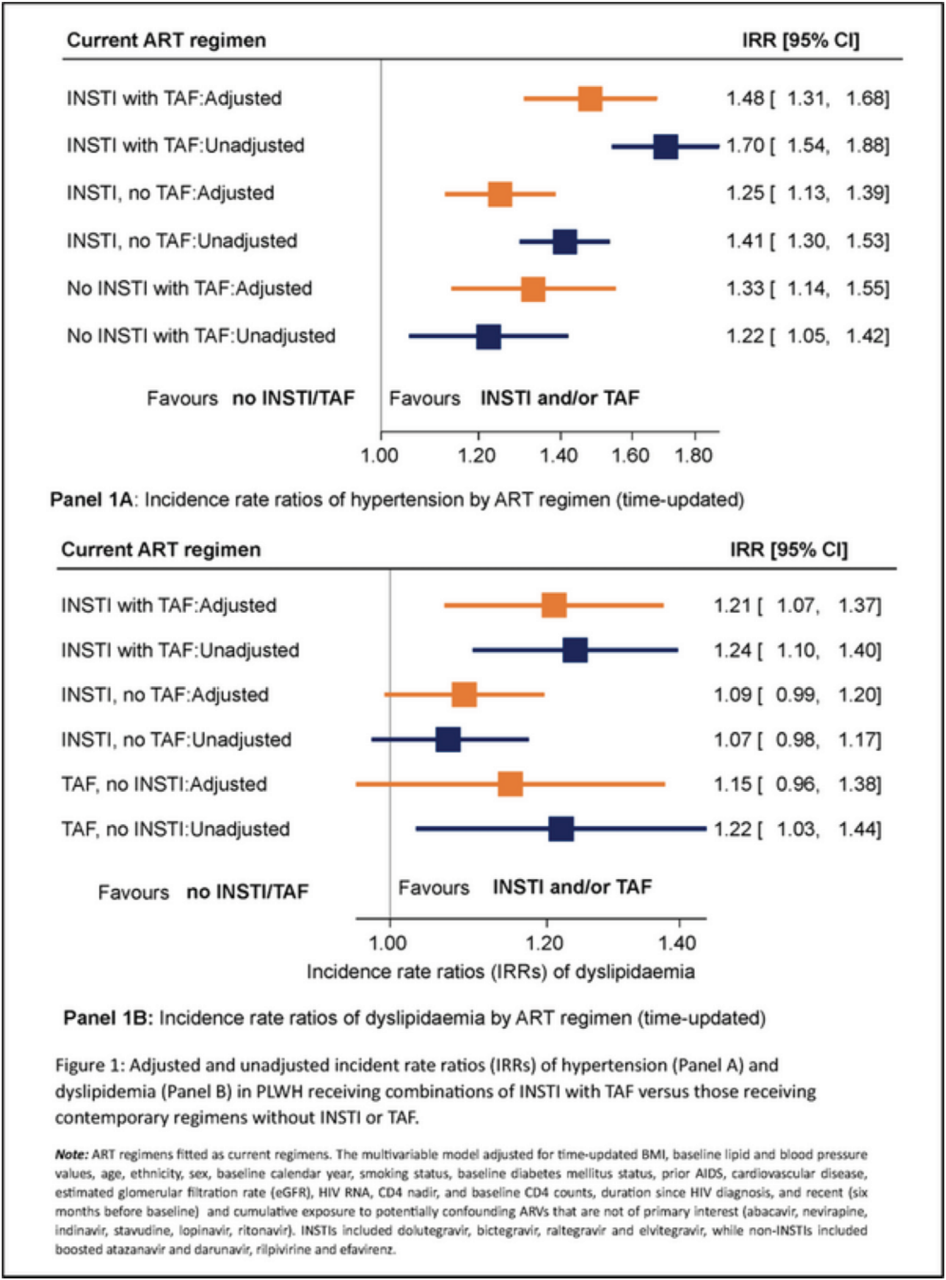
**Abstract OALBB0505‐Figure**.

**Methods: **PLWH ≥18 years, receiving INSTIs (DTG, BIC, RAL, EVG/c), or contemporary non‐INSTIs (DRV/b, ATV/b, EFV, RPV), with baseline and ≥2 follow‐up BMI and lipid/blood pressure results were followed‐up from the latest RESPOND or local cohort baseline date and censored at the earliest event date, last visit or 31/12/2021. We used multivariate Poisson regression adjusted for time‐updated BMI and confounder to determine the adjusted rate ratios (aIRR) of hypertension and dyslipidaemia by time‐updated ART regimens and test for interaction between BMI and ART.

**Results: **Of the 9,704 participants without hypertension, 2977(30.7%) developed hypertension over 39993 person‐years. In the unadjusted estimates, hypertension was more common with the use of INSTI with TAF or INSTI without TAF than ART without INSTI or TAF. Adjustment for time‐updated BMI attenuated the risk with concurrent use of INSTI with TAF (aIRR 1.48 confidence intervals [CI], 1.31–1.68) or INSTI without TAF (1.25, 1.13–1.39) (Fig.1). Of the 5231 participants included in the dyslipidemia analysis, 2689(51.4%) developed events over 19547 person‐years. In the unadjusted analysis, dyslipidaemia incidence was higher with concurrent use of TAF with INSTI or TAF alone. Adjustment for BMI attenuated dyslipidaemia risk associated with receiving TAF with INSTI (aIRR 1.21, CI 1.07–1.37), while the risk associated with TAF alone became non‐significant (1.15, 0.96–1.38). Hypertension and dyslipidemia increased with increasing BMI, but the association was not different between regimens (interaction P = 0.459 and 0.303, respectively).

**Conclusions: **In RESPOND, current use of INSTI or TAF and increases in BMI were associated with incident hypertension and dyslipidaemia. The relationship between BMI and hypertension or dyslipidaemia did not differ by ART regimen.

### OALBC0602 Should social network testing be offered as an additional HIV testing approach? A GRADE systematic review and meta‐analysis

A. Choong^1^, Y.M. Lyu^1^, E. Chow^1^, C. Johnson^2^, R. Baggaley^2^, N. Siegfried^3^, M. Barr‐DiChiara^2^, M. Jamil^2^, C. Fairley^1^, V. Macdonald^2^, J. Ong ^1^

^1^Monash University, Melbourne, Australia, ^2^World Health Organisation, Global HIV, Hepatitis and STIs Programme, Geneva, Switzerland, ^3^Independent clinical epidemiologist, Cape Town, South Africa

**Background: **Social network testing approaches (SNA) encourage individuals or ‘seeds’ to motivate sexual partners and/or those in their social networks who may benefit from HIV testing to test for HIV. To inform the World Health Organisation Guidelines Development Group, we conducted a systematic review to guide recommendations regarding SNA as an additional testing approach for all populations.

**Methods: **We systematically searched five databases: Medline, Embase, Global Health, CINAHL and Web of Science, from Jan 2010 to July 2022. We included randomized controlled trials (RCT) and non‐randomized studies (NRS) that compared SNA with non‐SNA or that compared different types of SNA. We used random‐effects meta‐analysis to combine effect estimates of studies that shared similar interventions, control, and outcomes. Certainty was assessed using the GRADE approach.

**Results: **From 18,956 records, we included 43 unique studies: 11 studies for the effectiveness of SNA vs. non‐SNA, 10 studies for the effectiveness of different types of SNA, four studies for resource use of SNA vs. non‐SNA, three studies for resource use of different types of SNA, and 23 studies for the acceptability of SNA. Based on one RCT and four NRS with low certainty evidence, SNA may increase uptake of HIV testing services compared to non‐SNA (Pooled RR 1.67, 95%CI:1.35–2.05, I^2^ = 99%). Based on four NRS with moderate certainty evidence, the proportion of first‐time testers was probably higher among partners or social contacts of seeds using SNA (compared to non‐SNA) (Pooled RR 1.23, 95%CI:1.01–1.48, I^2^ = 97%). Based on eight NRS with low certainty evidence, the proportion of people who tested positive for HIV may be higher among partners or social contacts of seeds using SNA (compared to non‐SNA) (Pooled RR 2.28, 95% CI: 1.18–4.39, I^2^ = 95%). High heterogeneity for these outcomes were mainly explained by population type and type of SNA.

**Conclusions: **SNA for HIV testing may be an effective, acceptable, and cost‐effective approach to improve HIV testing in all populations. The type of SNA to implement should be based on the setting, epidemiology, client preferences and resources available. SNA should be further scaled up to strengthen global efforts to end HIV as a public health threat by 2030.

### OALBC0603 Acceptability of CAB‐LA in cisgender female adolescents in South Africa, Uganda, and Zimbabwe (HPTN 084‐01)

E. Hamilton ^1^, D. Kemigisha^2^, H. Chauke^3^, M. Chitukuta^4^, K. Matambanadzo^4^, J. Etima^2^, N. Khoza^3^, L. Stranix‐Chibanda^5^, S. Hosek^6^, HPTN 084‐01 study team

^1^FHI 360, Science Facilitation, Durham, United States, ^2^MU‐JHU Research Collaboration, Kampala, Uganda, ^3^Wits RHI, Johannesburg, South Africa, ^4^University of Zimbabwe Clinical Trials Research Centre, Harare, Zimbabwe, ^5^UZ‐UCSF Collaborative Research Programme, Harare, Zimbabwe, ^6^Stroger Hospital of Cook County, Chicago, United States

**Background: **Initiation of and adherence to daily oral HIV pre‐exposure prophylaxis (PrEP) has been low among African adolescent girls and young women (AGYW). Along with other sociocultural factors, PrEP uptake and adherence are a function of product acceptability. Understanding the acceptability barriers and facilitators faced by AGYW in regard to long‐acting HIV prevention products is critical for successful implementation. This qualitative analysis explored acceptability of long‐acting injectable cabotegravir (CAB‐LA) among cisgender adolescent females in South Africa, Uganda, and Zimbabwe.

**Methods: **The HPTN 084‐01 study, which examined safety, tolerability and acceptability of CAB‐LA among 55 adolescent cisgender females, included a qualitative component to better understand the participants’ experiences with CAB‐LA (2021‐2022). In‐depth qualitative interviews were conducted near the end of the product exposure period (Week 34 – after 5 injections) with 15 participants (5 per site) to explore issues of acceptability of CAB‐LA injections, including negatives and positives associated with CAB‐LA, as well as qualities of the injection itself. Participant interviews were deductively coded by five team members using NVivo 12 and representative memos were created via thematic analysis.

**Results: **Several major themes emerged regarding acceptability of CAB‐LA injections. The needle size (1½ inch) and site of administration (gluteal muscle) were generally deemed acceptable by participants. Injection pain was the most reported barrier to acceptability, followed by injection site reactions and fear of the injection. Despite this, positive overall experiences with injections were reported because of the lack of adherence challenges with bi‐monthly injections as well as the discretion offered by CAB‐LA in comparison to daily oral tablets. In addition, familiarity with the mode of administration of CAB‐LA emerged as a theme around CAB‐LA and injectable contraceptives.

**Conclusions: **In regard to HIV prevention products, the importance of choices is evident in the HPTN 084‐01 data. While many participants reported a preference for CAB LA, and most (92%) chose to stay on CAB‐LA during the open label extension (HPTN 084), some participants still preferred oral tablets for various reasons, including pain and fear of the injection. These barriers and facilitators should be discussed with future clients as part of the decision‐making process around HIV prevention product choice.

### OALBC0604 High in‐hospital mortality in SARS‐CoV‐2 infected patients living with HIV during pre‐Delta, Delta and Omicron variant waves: finding from the WHO Global Clinical Platform for COVID‐19

S. Bertagnolio^1^, S. Inzaule^1^, R. Silva^2^, S.S. Thwin^2^, W. Jassat^3^, N. Ford ^1^, M. Vitoria^1^, M. Doherty^1^, J. Diaz^4^

^1^World Health Organization, Department of Global HIV, STI & Hepatitis Programmes, WHO, Geneva, Switzerland, ^2^World Health Organization, Department of Sexual and Reproductive Health and Research, WHO, Geneva, Switzerland, ^3^National Institute for Communicable Diseases, Johannesburg, South Africa, ^4^World Health Organization, Department of Country Readiness Strengthening, Health Emergencies Programme, WHO, Geneva, Switzerland

**Background: **There is limited data on the impact of SARS‐CoV‐2 variants on mortality among people living with HIV (PLHIV). We investigated changes in in‐hospital mortality during the different SARS‐CoV‐2 variant waves.

**Methods: Method**: We analyzed individual‐level data from the WHO Global Clinical Platform comprising 821,331 hospitalized children and adults from 42 countries. We used Cox regression to evaluate association of HIV co‐infection with in‐hospital mortality across SARS‐CoV‐2 pre‐Delta, Delta and Omicron variant waves and to assess risk factors for mortality among PLHIV.

**Figure:**
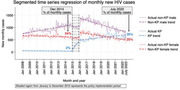
**Abstract OALBC0605‐Figure**.

**Results: **PLHIV had a 54% (aHR 1.54, 95%CI 1.42‐1.68) higher risk of death during the pre‐Delta variant wave, 56% (aHR 1.56, 95%CI 1.40‐1.74) during Delta variant wave and 142% (aHR 2.42, 95%CI 2.11‐2.78) during Omicron variant wave compared to HIV negative populations, with the risk being higher among those with CD4≤200 cells/mm^3^. While the mortality rate among HIV negative population declined from 21% (Delta wave) to 7.9% (Omicron wave), the reduction among PLHIV was only modest (from 25% to 18%). Reduction in mortality was even less apparent for PLHIV with CD4<200 cells/mm^3^. People with unknown HIV status also had a higher risk of death across the three waves. Common risk factors for mortality across the three SARS‐COV‐2 variant waves among PLHIV were severe/critical COVID‐19 at admission and CD4 ≤200 cells/mm^3^. PLHIV with at least one dose of COVID‐19 vaccination had 39% (aHR 0.61, 95%CI 0.40‐0.92) lower risk of death during the Delta variant wave and 38% (aHR 0.62, 95%CI 0.45‐0.85) during the Omicron variant wave compared to the unvaccinated.

**Conclusions: **While the mortality risk among HIV negative people decreased drastically in the omicron wave, only a modest reduction was observed in PLHIV, and especially in those with low CD4, resulting in a relatively greater hazard for PLHIV. The observed high risk of death among COVID‐19 patients with unknown HIV status calls for the need to intensify HIV testing and treatment as PLHIV who are unaware of their serologic status may be at risk of worse outcomes during the pandemic. These findings highlight the need to implement WHO guidelines recommending booster vaccine for populations most‐at‐risk of severe COVID‐19 outcomes, including in PLHIV.

### OALBC0605 An HIV Prevention model focused on key populations alone will not end HIV/AIDS in Thailand by 2030

C. Lertpiriyasuwat ^1^, S. Kerr^2^, R. Triamwichanon^3^, S. Jirajariyavej^4^, C. Bowonwatanuwong^5^, J. Sophonphan^2^, S. Gatechompol^2^, O. Putcharoen^6^, A. Avihingsanon^2^, K. Ruxrungtham^7^

^1^Thai Ministry of Public Health, Division of AIDS and STIs, Nonthaburi, Thailand, ^2^HIV‐NAT, Thai Red Cross AIDS Research Centre, Bangkok, Thailand, ^3^National Health Security Office, Bangkok, Thailand, ^4^Thaksin Hospital, Bangkok, Thailand, ^5^Thai AIDS Society, Bangkok, Thailand, ^6^Thai Red Cross AIDS Research Centre, Bangkok, Thailand, ^7^Chulalongkorn University, School of Global Health, Bangkok, Thailand

**Background: **Key populations (KP) are hidden drivers of the HIV epidemic. To facilitate achieving the 95:95:95 goals, Thailand implemented the Key Population‐Led Health Services (KPLHS) model through community‐based organisations (CBO) in 2015. We examined trends in new HIV positive diagnoses in the Thai National Treatment program (NAP) after at least 1 year of KPLHS implementation.

**Figure:**
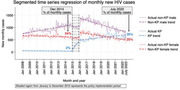
**Abstract OALBB0505‐Figure**.

**Methods: **The most recent voluntary counselling and testing (VCT) record of 14.5 million unique individuals with at least one VCT test result was matched with the National AIDS Program (NAP) database. Self‐reported risk was used to categorise the individual as a KP member, a non‐KP male or a non‐KP female. Trends in the number of new cases per month were assessed with segmented time‐series models. Three model segments corresponded to baseline (January 2008 to December 2014); a year lead‐in for policy implementation (from January 2015), and post‐implementation (January 2016 to July 2022).

**Results: **The policy increased case detection in all study groups during 2015 (Figure). In December 2014, KP accounted for 35/1756 (2%) new HIV cases, but increased to 482/2661 (18%) of new HIV cases in December 2015. From January 2016, new KP HIV case numbers increased by 2.8 (95%CI 1.7 to 3.9) per month, to 707/2013 (35%) of new HIV cases in July 2022. Non‐KP females accounted for 614/1756 (45%) of new HIV cases in December 2014 and 798/2661 (30%) of new cases in December 2015. Non‐KP female new HIV cases after January 2016 decreased by ‐3.8 (95%CI ‐4.8 to ‐2.9) cases per month, but accounted for 493/2013 (25%) of new HIV cases in July 2022.

**Conclusions: **The KPLHS model though CBO has effectively improved testing and diagnosis in KP. However, non‐KP women continue to account for a quarter of new cases. Policies to target these women are necessary to end AIDS.

### OALBD0602 “It was just the most horrible experience of my life” understanding social and care experiences during and after mpox illness: qualitative accounts of people diagnosed and close contacts in Australia

A.KJ Smith ^1,2^, D. Storer^3^, K. Lancaster^1^, V. Cornelisse^3,4^, J. MacGibbon^1^, B. Haire^2,3^, C. Newman^1,2^, V. Delpech^4^, S. Paparini^5^, J. Rule^6^, A. McNulty^7,8^, A. Bourne^3,9^, T. Broady^1^, H. Paynter^10^, M. Holt^1^

^1^Centre for Social Research in Health, University of New South Wales, Sydney, Australia, ^2^Australian Human Rights Institute, University of New South Wales, Sydney, Australia, ^3^Kirby Institute, University of New South Wales, Sydney, Australia, ^4^NSW Health, Sydney, Australia, ^5^SHARE Collaborative, Wolfson Institute of Population Health, Queen Mary University of London, London, United Kingdom, ^6^National Association of People Living with HIV Australia (NAPWHA), Sydney, Australia, ^7^Sydney Sexual Health Centre, Sydney, Australia, ^8^School of Population Health, University of New South Wales, Sydney, Australia, ^9^Australian Research Centre in Sex, Health and Society (ARCSHS), La Trobe University, Melbourne, Australia, ^10^Australian Federation of AIDS Organisations (AFAO), Sydney, Australia

**Background: **In May 2022, a global outbreak of mpox emerged, with a small number of mpox cases (n = 144) identified in Australia. There is scarce qualitative research focused on understanding people's experiences of mpox illness and their interactions with healthcare services. This study sought to document in‐depth qualitative accounts of the social, care, and health experiences of people directly affected by mpox.

**Methods: **Semi‐structured interviews were conducted between October‐December 2022 with 13 people diagnosed with mpox living in Australia, as well as 3 close contacts (household or sexual partners). 6‐month follow‐up interviews were conducted in April‐May 2023 with 7 participants, providing 23 interviews. Interviews were deidentified and thematically analysed.

**Results: **All participants were gay or bisexual cisgender men. Most reported acquiring mpox overseas on holiday (n = 11) in July or August 2022, and isolated or received care in Australia (n = 8). Participants’ experiences of mpox illness, diagnosis, care and recovery were highly distressing amidst the uncertainty of the outbreak, and severe symptoms and long isolation periods were difficult to manage. Physical symptoms were primarily confined to the acute illness period, including lesions, fever, and pain, but half of participants (n = 7) reported longer‐term social and physical sequelae from mpox, including continuing changes to sexual practices (avoiding sex), ongoing fatigue, psychological distress related to pain or clinical care, major scarring, and the need for corrective rectal surgery. Most participants diagnosed with mpox (n = 10) reported dissatisfaction with clinical care, including challenging communication with contact tracers, perceived judgement about sexual behaviour, inadequate pain management, or stigmatising care in hospital. Participants expressed a desire for greater empathy from clinicians and contact tracers and more proactive pain management.

**Conclusions: **Participants’ accounts portray negative healthcare experiences during an unfamiliar disease outbreak. This study highlights potential vulnerabilities in health system capacity to provide culturally‐appropriate care when responding to a disease that is linked to sexual practices, anogenital symptoms, and requires pain management. The potentially enduring aftereffects of mpox, including physical symptoms and healthcare‐related distress, suggest a need for attention to follow‐up care.

### OALBD0603 Using client‐centered models to sustain HIV service delivery to key populations in Uganda

V. Vasireddy ^1^, N.E. Brown^2^, N. Shah^3^

^1^U.S. Department of Defense Walter Reed Army Institute of Research, Kampala, Uganda, ^2^U.S. Ambassador to Uganda, U.S. Department of State, Kampala, Uganda, ^3^U.S. Department of Defense Walter Reed Army Institute of Research, Silver Spring, United States

**Background: **On 21 March 2023, the Parliament of Uganda passed the Anti‐Homosexuality Act (AHA) with overwhelming majority and re‐approved a revised version on 2 May 2023. The AHA criminalizes homosexual behavior with sentences ranging from 10 years to the death penalty. The hostile environment created with passing of AHA and the fear of law enforcement has led to reduced access to Key Population (KP)‐friendly services.

**Description: **The President's Emergency Plan for AIDS Relief (PEPFAR) supports over 1.4 million Ugandans on HIV treatment. PEPFAR supports over 50 drop‐in‐centers (DIC) that provide HIV prevention and treatment services focusing on KP clients. Service delivery data from DICs is reported weekly and disaggregated by type of KP and services. To protect client safety and confidentiality at the DICs, we de‐identified the three DICs used in this analysis.

**Lessons learned: **The AHA discourse increased in the Ugandan media starting in January 2023. Weekly data show a steady decrease in KP client visits to the 3 DICs, with the lowest being when the first version of AHA was debated and approved in the Parliament. PEPFAR instituted measures in early March, some of which are below:

- home delivery of anti‐retroviral therapy (ART), and prevention products like condoms and Pre‐Exposure Prophylaxis (PrEP)
- reinforcing safety measures at DICs
- scaling up multi‐month dispensing (MMD) for eligible clients
- employing paralegal peers to offer legal support for KP clients

These supportive measures led to a resumption of KP clients accessing HIV services at these 3 DICS by April (Chart 1).

**Figure:**
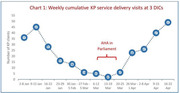
**Abstract OALBD0603‐Figure**.

**Conclusions/Next steps: **The AHA is not a law yet but shows significant negative consequences on access to HIV services. While these 3 DICs show that PEPFAR programs can rapidly implement supportive measures, over 20 DICs have not seen a resurgence of KP clients despite interventions. Punitive laws against KP have the potential to derail HIV epidemic control.

### OALBD0604 Bespoke strategies for coping with addiction and HIV during and after COVID‐19 crisis by prisoners with comorbid HIV and a history of opioid use disorder in Kyrgyzstan

Y. Rozanova ^1^, M. Sabirova^2^, Y. Malyshev^2^, A. Opobekova^2^, E. Yuldashev^2^, N. Shumskaya^2^

^1^Yale University, AIDS Program, New Haven, United States, ^2^AIDS Foundation East West Kyrgyzstan, Bishkek, Kyrgyzstan

**Background: **A third of 10,500‐prisoner population in Kyrgyzstan, a low‐income Central Asian country, have a history of opioid use disorder (OUD), and 70% have injected drugs within‐prison. Since June 2020, strict 18‐month Covid‐19 lockdown disrupted heroin‐smuggling channels into prisons. We examined how prisoners with OUD managed their addiction during the lockdown.

**Methods: **From Nov’22‐Jan 2023 using a semi‐structured guide we interviewed twenty prisoners with HIV and OUD from two male medium security prisons near Bishkek. Audio‐recorded interviews asked participants about physical and mental health, methadone maintenance treatment (MMT) and HIV care uptake during and after Covid‐19 lockdown, and how prisoners managed their addiction and withdrawal symptoms in the absence of heroin. Transcripts were analyzed using NVivo qualitative management software by three researchers. Texts were coded using an inductively developed codebook for both explicit and latent meanings. Codes were aggregated into broader themes, with quotes exemplifying each theme. Per agreement with the Republican Penitentiary Service researchers were not obligated to report individual risky behavior but shared recommendations for future service improvement.

**Results: **Covid‐19 lockdown blocked heroin procurement channels for prisoners with OUD, causing withdrawal experiences and anxiety about impending withdrawal symptoms. Heroin shortages caused restructuring of within‐prison gang hierarchies, increasing financial pressures on the peripheral members. Consequently, prisoners with OUD developed make‐shift strategies for managing their addiction in the context of the crisis. Common strategies to alleviate withdrawal symptoms involved using alternative substances, abusing pharmacy‐purchased medications, and drinking alcohol that prisoners made on site. Prisoners with HIV purposefully discontinued ART to provoke physical health worsening and be transferred to a prison hospital where links to the external heroin market were preserved during lockdown. Interestingly, despite availability of MMT programs, prisoners were reluctant to join them despite suffering withdrawal symptoms due to stigmatization of MMT by prison sub‐culture.

**Conclusions: **Covid‐19 lockdown disrupted heroin market access, and prisoners’ coping with opioid withdrawal compromised their HIV care and prevention. Despite heroin shortages, MMT uptake did not increase in prisons during the lockdowns due to stigma. Covid‐19 crisis learnings suggest co‐production of interventions by researchers, prisoner participants, and clinicians to manage withdrawal (and overdose) among prisoners.

### OALBD0605 Moral trauma among health professionals providing HIV services in Mozambique: preliminary results of a qualitative study

P. Paulo^1^, C. De Schacht ^2^, E. Graves^3^, C. Lucas Fonseca^2^, A. Aboobacar^4^, V. Cumbe^5^, C.M. Audet^6,3^

^1^Friends in Global Health (FGH), Evaluations, Quelimane, Mozambique, ^2^Friends in Global Health (FGH), Evaluations, Maputo, Mozambique, ^3^Vanderbilt University Medical Center (VUMC), Vanderbilt Institute for Global Health (VIGH), Nashville, United States, ^4^Provincial Health Directorate of Zambézia (DPS‐Z), Quelimane, Mozambique, ^5^Beira Central Hospital, Psychology, Beira, Mozambique, ^6^Vanderbilt University Medical Center, Department of Health Policy, Nashville, United States

**Background: **Provider burnout and HIV‐related stigma is an urgent, and frequently interconnected problem globally. In sub‐Saharan Africa, health professionals often deliver care in under‐resourced facilities to clients who lack the financial resources, social support, and/or understanding to fully adhere to medications. This confluence of challenges can lead to provider frustration, burnout, and moral trauma, resulting in poor care provision and client dissatisfaction. This study assessed factors influencing burnout and stigma among health professionals in Zambézia Province, Mozambique.

**Methods: **We conducted a qualitative study employing in‐depth interviews among health professionals providing HIV services in four health facilities, in Zambézia Province, between November 2022 and January 2023. A semi‐structured interview guide probed potential factors impacting professional burnout, stigmatizing attitudes and behaviors, and suggestions for improving the well‐being of health professionals. Thematic analysis was performed.

**Results: **Forty‐eight health professionals were interviewed, 23 (48%) men; median age was 30 years (IQR 27–37); 23 (48%) were clinicians, 9 (19%) lab/pharmacy technicians, 14 (29%) counselors/peer educators, 14 (4%) non‐clinicians. Health professionals choose a career in the health sector wanting to help people, but stated that lack of materials, medicines, staff, support from their superiors, low salary, and attending clients deemed as difficult were factors causing frustration and burnout. They usually deal with frustrations through conversation with friends/family or taking a break from the clinic between client attendance. Defaulting persons, those complaining about their care, men, adolescents and educated persons were reported as those causing most frustration. The respondents felt that seeing individuals’ improvement, positive interactions with clients and colleagues and clients following recommendations, are factors contributing to their job satisfaction. For the improvement of their well‐being, respondents suggested psychological support for health professionals, off‐site trainings/workshops, improvement of infrastructure of health facilities, more staff, and salary increase.

**Conclusions: **Working with persons attending HIV services deemed as difficult, lacking support and limited resources (staff, medicines, materials) influence burnout and frustration of health professionals providing HIV services in Zambézia Province. Providing psychosocial support for health professionals, trainings on effective communication and coping with frustrations should be explored to improve their well‐being, and to ensure provision of quality services for HIV care.

### OALBE0602 e‐PrEPPY: Enabling an all‐virtual, community‐led and demedicalized PrEP service for men who have sex with men (MSM) in the Philippines

J.D. Rosadiño ^1,2^, D. Cruz^1,2^, A. Aspiras^1^, J.P. Benito^1^, E.J. Tamboong^1^, J.D.M. Dela Cruz^1^, R. Pagtakhan^1^

^1^LoveYourself, Inc., Mandaluyong, Philippines, ^2^University of the Philippines ‐ Open University, Faculty of Management and Development Studies, Los Baños, Philippines

**Background: **Since its introduction in 2017, the uptake of PrEP in the Philippines has reached >10,000 individuals. With the rate of HIV transmission experienced by the country, other models to effectively roll‐out PrEP are urgently needed. Barriers in PrEP uptake include challenges to service access, further highlighted by limited mobility caused by COVID‐19. Here we introduce an all‐virtual, community‐led PrEP program for MSM using a demedicalized approach.

**Description: **MSM clients who reported HIV‐negative in a online unassisted HIV self‐testing program were offered their interest in PrEP. A blood‐based self‐test kit is sent to their delivery address, and are guided with instructions‐for‐use and result reporting. Once marked HIV non‐reactive, a self‐assessment tool is sent to determine their sexual behavior and the presence of the following: acute retroviral syndrome, kidney‐related morbidity, and supplementation. These information are then validated by trained community peers, and are provided PrEP information and counseling via telemedicine. Once assessed and marked eligible, the client is sent one PrEP bottle, another self‐test kit, and a QR code for their refill instructions delivered via courier within 3 days. For follow‐ups, the client uses the received self‐test kit and reports the results. Once marked HIV non‐reactive, the client is sent one self‐test kit with PrEP bottles relative to the number of their visit. The demedicalized process is detailed in Figure 1.

**Lessons learned: **Between August and April 2023, 230 clients were initiated with PrEP, which translates to 10.44% (of 2203 clients) who reported HIV‐negative via the all‐virtual HIV self‐testing process. 100 clients (43.48%) have completed their first monthly visit, with 92 clients reported taking PrEP daily.

**Conclusions/Next steps: **This program provides evidence that an all‐virtual, community‐led and demedicalized PrEP approach integrated with unassisted HIV self‐testing is possible. Further demand‐generation and other offline‐based key‐population‐friendly activities will be done to increase awareness and enable access to more clients.

**Figure:**
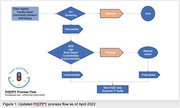
**Abstract OALBE0602‐Figure**.

### OALBE0603 Costs and cost‐effectiveness of immediate initiation of antiretroviral therapy upon diagnosis of HIV (Rapid Start) in the United States

S.B. Shade ^1^, P. Murnane^1^, M. Damle^2^, K. Brooks^2^, L. Senter^2^, D. Middleton^2^, T. Donohoe^3^

^1^University of California, San Francisco, Epidemiology and Biostatistics, San Francisco, United States, ^2^Cicatelli Associates Incorporated (CAI), New York, United States, ^3^University of California, Los Angeles, Family Medicine, Los Angeles, United States

**Background: **Immediate initiation of antiretroviral therapy (ART) upon diagnosis of HIV (Rapid Start) results in better viral suppression and retention in HIV care. Cicatelli Associates Inc. (CAI) was funded by the Health Resources and Services Administration's HIV/AIDS Bureau, Special Projects of National Significance for the Rapid Antiretroviral Dissemination Assistance Provider (DAP) project from 2020–2023. In partnership with the University of California, Los Angeles (UCLA), we estimated the cost‐per‐person of Rapid Start and modeled the potential cost‐effectiveness of Rapid Start services.

**Methods: **We worked with seven established Rapid Start provider sites in the United States from 7/2022‐2/2023 to estimate costs associated with initiation of ART and follow‐up through eight weeks: 1) during the year prior to implementation of Rapid Start; 2) the first year of implementation; and 3) during a year of sustained implementation. Effort and resources (i.e., personnel, recurring goods and services) were recorded in standardized Excel workbooks to estimate cost‐per‐person each year. Provider sites reported information on the number of people who initiated ART each year. Quality‐adjusted life‐years (QALYs) gained during Rapid Start implementation were modeled based on ongoing and published research. We report information on the additional cost‐per‐person who received Rapid Start services and the incremental cost‐per‐QALY gained during the first year of implementation and during sustained implementation relative to the year prior to implementation of Rapid Start.

**Results: **Median additional cost‐per‐person of Rapid Start was $98 (range = cost saving to $13,250) during the first year of implementation and $46 (range = cost saving to $12,737) during sustained implementation. Costs were lower in sites with more concentrated clinical teams, more established support service programs, and larger increases in the number of people who initiated ART. Within the six organizations that experienced increases in the number of people who initiated ART, the median incremental cost‐per‐QALY gained was $3971 (range = cost saving to $52,915) during the first year of implementation and $2492 (range = cost saving to $46,578) during sustained implementation.

**Conclusions: **Rapid Start programs were reasonably inexpensive to implement and uniformly cost‐effective or cost saving both during initial and sustained implementation. These programs have the potential to improve clinical outcomes and onward transmission of HIV.

### OALBE0604 Feasibility and acceptability of delivering event driven PrEP (9ED‐PrEP) to prevent HIV among MSM: a pilot project in Harare, Zimbabwe

P. Moyo ^1^, J. Murungu^1^, C. Hera^1^, S. Nyakuwa^1^, C.P. Muchemwa^1^, I. Mahaka^1^, P. Manyanga^2^, H.T. Bara^3^, T. Sola^4^

^1^Pangaea Zimbabwe AIDS Trust, Harare, Zimbabwe, ^2^Zimbabwe Technical Assistance, Training & Education Center for Health, Harare, Zimbabwe, ^3^City of Harare, Harare, Zimbabwe, ^4^Ministry of Health and Child Care, Harare, Zimbabwe

**Background: **Zimbabwe has made significant progress towards HIV epidemic control, with a 70% reduction in new HIV infections since 2010. However, key populations (KPs), including men who have sex with men (MSM) continue to contribute a significant proportion of new HIV infections relative to their population size. Oral Pre‐exposure prophylaxis (PrEP), which is highly effective in reducing the risk of HIV infection, is recommended for all people at substantial risk in Zimbabwe. Following the guidance from WHO, we implemented a pilot project This intervention was to assess the feasibility and acceptability of delivering event driven PrEP (ED‐PrEP) to prevent HIV among MSM in Harare, Zimbabwe

**Methods: **A cohort study was conducted from November 2022 to April 2023. Qualitative and quantitative data was collected to assess feasibility, acceptability, client and provider experiences and outcomes among clients testing HIV negative who were assessed for risk, counselled and offered a choice of daily oral PrEP (D‐PrEP) or ED‐PrEP. Patterns of use and outcomes such as continuation on PrEP, incidence of sexually transmitted infections and adverse events were assessed by providers during the quarterly visits and by peers through mobile based platforms. Focus group discussions and in‐depth interviews were conducted using a semi‐structured questionnaire to explore experiences.

**Results: **A total of 196 MSM were initiated on PrEP in the six months of implementation (19 D‐PrEP; 177 on ED‐PrEP). At initiation, 60% (106) of the clients switched from D‐PrEP to ED‐PrEP citing infrequent events and reduction in pill burden whilst 10% switched back to D‐PrEP citing unplanned events. Continuation was significantly higher among ED‐PrEP users, 87.5% vs 12.5% at month 1 and 74% vs 26% at month 3. No adverse events were recorded; Forty‐four percent (44%, 69) preferred ED‐PrEP. Incidence of STIs was at 7% (13). No seroconversions were recorded.

**Conclusions: **It is feasible to deliver ED‐PrEP as an additional HIV prevention method for MSM. Clients can also switch from one method to another based on their prevention needs and preferences.

### OALBE0605 Key population‐led same‐day antiretroviral therapy initiation hubs in Bangkok, Thailand: an evaluation of HIV cascade outcomes from a hybrid type 3 implementation‐effectiveness trial

S. Lujintanon ^1,2^, A. Wongsa^1^, K. Sinchai^1^, J. Boonruang^1^, S. Thitipatarakorn^1^, P. Panpet^3^, T. Chaisalee^3^, S. Janyam^4^, N. Teeratakulpisarn^1^, P. Phanuphak^1^, R.A. Ramautarsing^1^, R. Janamnuaysook^1,5^, N. Phanuphak^1,5^

^1^Institute of HIV Research and Innovation, Bangkok, Thailand, ^2^Johns Hopkins Bloomberg School of Public Health, Epidemiology, Baltimore, United States, ^3^Rainbow Sky Association of Thailand, Bangkok, Thailand, ^4^Service Worker in Group Foundation, Bangkok, Thailand, ^5^Chulalongkorn University, Center of Excellence in Transgender Health, Bangkok, Thailand

**Background: **Approximately 20% of key populations (KPs), particularly men who have sex with men (MSM) and transgender women, diagnosed with HIV at community‐based organizations (CBOs) in Bangkok, Thailand, experienced pre‐antiretroviral therapy (ART) attrition. To bridge this gap, we pioneered the KP‐led same‐day ART initiation (SDART) hubs. This analysis evaluated the HIV cascade outcomes from ART initiation to viral suppression.

**Methods: **An implementation‐effectiveness trial, guided by Proctor's Implementation Outcome Framework and Consolidated Framework for Implementation Research, was conducted at 2 CBOs in Bangkok. The following pre‐specified strategies facilitated the implementation process: developing multi‐stakeholder partnerships, adapting SDART to CBO context, training KP lay providers, and using evaluative and iterative strategies. CBO clients were eligible for KP‐led SDART if they were 13+ years, HIV‐positive, ART‐naïve, willing, and clinically ready. Trained KP lay providers led counseling, laboratory testing, opportunistic infection screening, and ART dispensing under physician supervision primarily via telehealth. Participants were followed‐up at 2–4 weeks after initiation, referred to their long‐term ART facility, and supported for 12 months by lay providers. Additional strategies, including care coordination between different providers and assisting participants with health system navigation, were enacted to enhance HIV outcomes.

**Results: **Between 8‐Oct‐2021 and 31‐Mar‐2023, 587 individuals enrolled in the study. The median (IQR) age was 25 (22‐31), 72.1% were MSM, 7.3% were transgender women, and median (IQR) CD4 cell count was 384 (272‐512). 585 (99.7%) accepted KP‐led SDART; 97.9% (573/585) started ART in which 52.0% (298/573) started on the same day of HIV diagnosis at CBO and the median (IQR) ART initiation duration was 0 (0‐1) day. 12 participants who did not start ART were referred to hospitals for clinical investigations, including for suspected tuberculosis (n = 5), cryptococcal meningitis (n = 2), and pneumocystis pneumonia (n = 3). Among KP‐led SDART participants who reached months 6 and 12, 87.0% (349/401) and 84.6% (115/136) remained in care, respectively. Of 210 participants who tested viral load, 94.2% had viral load <50 copies/mL.

**Conclusions: **Task‐shifting ART initiation to trained KP lay providers can promptly link healthy KPs to care with high retention and viral suppression. KP‐led SDART should be scaled up nationally to accelerate the end of AIDS epidemic in Thailand.

### OALBX0102 Doravirine/Islatravir (100mg/0.75mg) once daily compared to bictegravir/emtricitabine/tenofovir alafenamide (B/F/TAF) as Initial HIV‐1 treatment: 48 week results from a double‐blind phase 3 trial

J.K. Rockstroh ^1^, R. Paredes^2^, P. Cahn^3^, J.‐M. Molina^4^, S. Sokhela^5^, F. Hinestrosa^6^, S. Kassim^7^, E. Valencia Ortega^8^, D. Cunningham^9^, J. Ghosn^10^, J.R. Bogner^11^, H. Gatanaga^12^, E. Asante‐Appiah^13^, Y. Zang^13^, S.O. Klopfer^13^, K. Eves^13^, M.L. Pisculli^13^, K. Squires^13^, T.A. Correll^13^

^1^University Hospital Bonn, Department of Medicine I, Bonn, Germany, ^2^Germans Trias i Pujol University Hospital, Barcelona, Spain, ^3^Fundación Huesped, Buenos Aires, Argentina, ^4^University of Paris Cité, Paris, France, ^5^University of the Witwatersrand, Johannesburg, South Africa, ^6^Orlando Immunology Center, Orlando, United States, ^7^Desmond Tutu HIV Foundation, Capetown, South Africa, ^8^La Paz University Hospital, Madrid, Spain, ^9^Pueblo Family Physicians, Phoenix, United States, ^10^Bichat‐Claude Bernard Hospital, Paris, France, ^11^University Hospital of Munich, Med Department IV, Munich, Germany, ^12^AIDS Clinical Center, NCGM, Tokyo, Japan, ^13^Merck & Co., Inc., Rahway, United States

**Figure:**
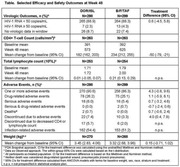
**Abstract OALBX0102‐Table**.

**Background: **Doravirine (DOR), an approved NNRTI, and Islatravir (ISL), an investigational nucleoside reverse transcriptase translocation inhibitor (NRTTI), have complementary mechanisms of action and resistance profiles. In virologically suppressed adults, switching to DOR/ISL (100mg/0.75mg) was non‐inferior to continuing prior antiretroviral regimens. We report week 48 results from a double‐blind non‐inferiority trial (NCT04233879) evaluating initial HIV‐1 treatment with DOR/ISL (100mg/0.75mg) compared to B/F/TAF.

**Methods: **Antiretroviral treatment‐naïve adults with HIV‐1 were randomized (1:1) to once‐daily oral DOR/ISL (100mg/0.75mg) or B/F/TAF, stratified by screening CD4 count (</≥200 cells/mm^3^) and HIV‐1 RNA (≤/>100,000 copies/mL). The primary efficacy endpoint was HIV‐1 RNA <50 copies/mL at week 48 (FDA Snapshot approach, non‐inferiority margin 10%, planned N = 680). Due to exposure‐related reductions in CD4+ T‐cells and total lymphocytes, enrollment was closed before full accrual.

**Results: **Of 599 randomized participants, 597 were treated with DOR/ISL (n = 298) or B/F/TAF (n = 299); mean age 35.2 years, 25% female, 29% Black, 20% had pre‐treatment CD4 count <200 cells/mm^3^ and 19% had pre‐treatment HIV‐1 RNA >100,000 copies/mL. At week 48, 88.9% receiving DOR/ISL and 88.3% receiving B/F/TAF had HIV‐1 RNA <50 copies/mL. Virologic failure (2 consecutive HIV‐1 RNA ≥200 copies/mL) occurred in one DOR/ISL participant (with acquired resistance‐associated mutations to DOR, due to non‐adherence) and 4 B/F/TAF participants. Mean increase from baseline in CD4+ T‐cell count was 182 cells/mm^3^ for DOR/ISL and 234 cells/mm^3^ for B/F/TAF. The treatment groups had similar mean weight gain (table) and similar rates of drug‐related adverse events (AEs) and infection‐related AEs (table). Discontinuation due to AE was higher for DOR/ISL (7.4% vs 3.3%) due to protocol‐required discontinuations for decreased CD4 or total lymphocyte counts.

**Conclusions: **DOR/ISL (100mg/0.75mg) was non‐inferior to B/F/TAF for initial treatment of HIV‐1 and was generally well‐tolerated. There were small treatment differences in CD4+ T‐cell and lymphocyte count changes, with similar rates of infection‐related AEs observed.

### OALBX0103 A social network‐based intervention increases HIV self‐testing and linkage to health facilities among fishermen in Kenya

C.S. Camlin ^1^, L. Sheira^2^, Z.A. Kwena^3^, E.D. Charlebois^4^, K. Agot^5^, J. Moody^6^, B. Ayieko^5^, S.A. Gutin^7^, A. Ochung^5^, P. Olugo^5^, J. Lewis‐Kulzer^1^, H. Nishimura^4^, M. Gandhi^4^, E.A. Bukusi^3^, H. Thirumurthy^8^

^1^University of California, San Francisco, Obstetrics, Gynecology & Reproductive Sciences, San Francisco, United States, ^2^University of California, San Francisco, Epidemiology & Biostatistics, San Francisco, United States, ^3^Kenya Medical Research Institute, Nairobi, Kenya, ^4^University of California, San Francisco, Medicine, San Francisco, United States, ^5^Impact Research & Development Organization, Kisumu, Kenya, ^6^Duke University, Durham, United States, ^7^University of California, San Francisco, Community Health Systems, San Francisco, United States, ^8^University of Pennsylvania, Philadelphia, United States

**Background: **Engaging men in HIV prevention and treatment is crucial to ending the AIDS epidemic in sub‐Saharan Africa, but many men are unaware of their serostatus and highly‐mobile men like Lake Victoria fishermen have particularly low uptake of prevention and treatment. We conducted a cluster randomized trial to determine if an HIV status‐neutral, social network‐based approach could improve testing and linkage outcomes among fishermen.

**Methods: **The Owete study (NCT04772469) mapped the male social networks of fishermen in three beach communities in Siaya, Kenya, and identified distinct social networks (“clusters”) with a highly‐connected, network‐central man (“promoter”) in each network. Clusters were randomized to an intervention group in which promoters were trained and offered (a)multiple HIV self‐tests (HIVST) to distribute to cluster members, and (b)transport vouchers (US$4) to encourage cluster members to link to HIV treatment or pre‐exposure prophylaxis (PrEP). In control clusters, promoters received HIV information and referral vouchers for free self‐tests in nearby clinics, and encouraged to offer them to cluster members. We compared self‐reported HIV testing in past three months among participants in intervention and control clusters at three‐month follow‐up visit, using a cluster‐adjusted two‐sample test of proportions. Participants with missing data were coded as failure unless known to be living with HIV (per health facility records). Secondary analyses examined testing via any modality (counselor or HIVST) and linkage to facility.

**Results: **A total of 934 men in 156 social network clusters were mapped. Of these, 733 completed baseline and 666 follow‐up surveys, with 14 deaths due to study‐unrelated causes. Participants’ average age was 37 years; 78% were married, 22% in polygynous relationships. Self‐reported HIV testing via HIVST at three months was significantly higher in intervention clusters (60% vs. 10%, p<0.001, intent‐to‐treat). HIV testing via any modality was also significantly higher in intervention clusters (47% vs. 27%, p<0.001). Following testing, linkage to facility for HIV treatment or PrEP was significantly higher in intervention clusters (70% vs. 17%, p<0.001).

**Conclusions: **A social network‐based, status‐neutral intervention in Kenya improved men's HIV testing and linkage outcomes and is a promising way to engage hard‐to‐reach populations of men in prevention or treatment.

### OALBX0104 Sustained aviraemia in the absence of anti‐retroviral therapy in male children following in utero vertical HIV transmission

G. Cromhout ^1^, N. Bengu^2^, E. Adland^3^, N. Herbert^3^, N. Lim^3^, K. Govender^4^, R. Fillis^5^, K. Sprenger^6^, V. Vieira^3^, S. Kannie^7^, J. van Lobenstein^7^, K. Chinniah^8^, C. Kapongo^2^, R. Bhoola^9^, M. Krishna^9^, N. Mchunu^5^, M.C. Puertas^10^, A. Groll^11^, K. Reddy^4^, T. Ndung'u^4^, C. Ochsenbauer^12^, N. Cotugno^13^, P. Palma^13^, A. Tagarro^14^, J. Roider^15^, C. Giaquinto^16^, P. Rossi^13^, J. Kappes^12^, J. Martinez‐Picado^10^, M. Archary^1^, P. Goulder^3^

^1^University of KwaZulu‐Natal, Paediatrics, Durban, South Africa, ^2^Queen Nandi Regional Hospital, Paediatrics, Empangeni, South Africa, ^3^University of Oxford, Paediatrics, Oxford, United Kingdom, ^4^African Health Research Institute, Durban, South Africa, ^5^Umkhuseli Innovation Research Management, Pietermaritzburg, South Africa, ^6^Umkhuseli Innovation Research Managemwnt, Pietermaritzburg, South Africa, ^7^General Justice Gizenga Mpanza Regional Hospital, Stanger, South Africa, ^8^Mahatma Gandhi Memorial Hospital, Durban, South Africa, ^9^Harry Gwala Regional Hospital, Pietermaritzburg, South Africa, ^10^IrsiCaixa AIDS Research Institute, Barcelona, Spain, ^11^TU Dortmund University, Dortmund, Germany, ^12^University of Alabama at Birmingham, Birmingham, United States, ^13^Ospedale Pediatrico Bambino Gesù, Rome, Italy, ^14^Hospital 12 de Octubre, Madrid, Spain, ^15^LMU University Hospital, Munich, Germany, ^16^University of Padova, Padua, Italy

**Background: **Case reports of post‐treatment control of HIV in children initiating early combination antiretroviral therapy (cART) prompt the hypothesis that a subset of very‐early treated children can achieve post‐treatment control without additional interventions. To test this, and identify underlying control mechanisms, we conducted a longitudinal study in KwaZulu‐Natal, South Africa of 281 mother‐child pairs monitored from delivery following in utero HIV transmission.

**Methods: **The study period was 2015–2023. All infants received ART at birth, and >92% of infants received cART prior to birth via placental transfer of maternal cART. ART adherence was monitored via plasma cART concentrations determined using liquid chromatography‐tandem mass spectrometry (LC‐MS/MS), maternal history, pill‐counting and pharmacy records. After generating chimeric gag‐protease‐NL4‐3 viruses following viral RNA isolation and nested RT‐PCR amplification of mother and child gag‐protease from baseline plasma, type I interferon (IFN‐I) sensitivity and replicative capacity of transmitted viruses were determined using the reporter cell lines U87‐snLuc/EGFP and CEM‐GXR, respectively.

**Results: **Maintenance of aviraemia was highly dependent on cART adherence, irrespective of infant baseline plasma viral load. Exceptionally, five males were identified in whom aviraemia was maintained (for >3m to >19m) despite persistent cART non‐adherence. By contrast, the majority (60%) of the cohort was female (p = 0.01). Higher rates of in utero transmission to female fetuses was associated with female susceptibility to IFN‐I resistant virus (p<0.0001) that tended to have low viral replication capacity (p = 0.0001). While viruses transmitted to male fetuses overall were typically IFN‐I sensitive and of higher replicative capacity, those transmitted to males maintaining aviraemia despite persistent cART non‐adherence had low replicative capacity (p<0.0001; **Figure**: circled are the 4 baseline viruses from the 5 males that were analysed; all 4 were replication competent, in one case replication capacity was <LoD).

**Figure:**
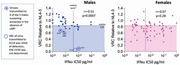
**Abstract OALBX0104‐Figure**.

**Conclusions: **These data suggest that early‐life innate immune sex differences contribute significantly to post‐treatment control in children living with HIV.

### OALBX0105 Socioeconomic support to improve completion of tuberculosis preventive therapy among adults starting HIV treatment in Tanzania: a quasi‐cluster randomized trial

C.A. Fahey ^1^, W. Maokola^2^, A. Sabasaba^3^, J. Msasa^3^, A. Mnyippembe^3^, Y. Ndungile^2^, S.I. McCoy^4^, P.F. Njau^2^

^1^University of Washington, Department of Epidemiology, Seattle, United States, ^2^Ministry of Health, National AIDS Control Program, Dodoma, Tanzania, United Republic of, ^3^Health for a Prosperous Nation, Dar es Salaam, Tanzania, United Republic of, ^4^University of California, Berkeley, Division of Epidemiology, Berkeley, United States

**Background: **Robust evidence shows that financial incentives for clinic attendance provided during the first months of antiretroviral therapy (ART) reduce loss to follow‐up (LTFU). Given low rates of completion of isoniazid preventive therapy (IPT) for tuberculosis (TB) during the first months of ART, financial incentives may also improve IPT completion, especially when combined with additional support for individuals at highest risk of LTFU. This study evaluated whether an intervention comprised of financial incentives plus targeted peer counseling improved completion of IPT.

**Methods: **We conducted a quasi‐cluster randomized trial (CRT) at 19 HIV primary care facilities in northwestern Tanzania: 16 control sites from a parallel CRT and three additional randomly selected intervention sites meeting the same eligibility criteria. At each facility, clinic staff enrolled adults (≥18 years) who had recently initiated ART (≤30 days). Participants at control facilities received usual HIV care, while those at intervention facilities additionally received (1) digital financial incentives for monthly clinic attendance [22,500 TZS (US $10)] for up to 6 months; plus (2) early pairing with a peer counselor for a subset of participants with high predicted LTFU risk, determined using a brief screening questionnaire at enrollment. We compared receipt of the full 6‐month course of IPT between intervention and control groups, measured via medication dispensing records, using generalized estimating equations clustered by facility.

**Results: **From May to November, 2021, we enrolled 1,018 participants (intervention n = 90, control n = 928). Participants were 58% female with a mean age of 37 years. Intervention facilities were smaller and more urban than the average control site, while individual‐level characteristics were balanced between groups. In the intervention group, 85 (94%) participants received at least one cash transfer (mean: 5.2) and 40 (44%) participants who met the screening threshold for higher LTFU risk were also paired with a community health worker for additional adherence counseling. At 6 months after starting ART, 67% of intervention participants versus 51% of control participants had received a full course of IPT (risk difference: 16.0, 95% CI: 2.3, 29.7).

**Conclusions: **These findings strengthen the case for providing short‐term socioeconomic support to ART initiates, with potential benefit for TB prevention efforts.

### OALBX0202 Positive Predictive Value of HIV Serological Tests in HPTN 084 Trial

M. Hosseinipour ^1,2^, E. Voldal^3^, B. Hanscom^3^, S. Eshleman^4^, E. Piwowar‐Manning^4^, Y. Agyei^4^, K Nyarota^5^, F Angira^6^, S. Dadabhai^7^, D. Gadama^2^, BG Mirembe^8^, P. Hunidzarira^5^, S. Innes^9^, D Kalonji^10^, J. Makhema^11^, P Mandima^5^, A. Marais^12^, J Mpendo^13^, P Mukwekwerere^5^, N Mgodi^5^, V Naidoo^14^, P Nahirya Ntege^15^, H Nuwagaba‐Biribonwoha^16^, E Roos^17^, N Singh^10^, B Siziba^9^, E Spooner^10^, J. Farrior^18^, S. Rose^18^, L. Soto‐Torres^19^, J Rooney^20^, A.R. Rinehart^21^, R Landovitz^22^, M.S. Cohen^1^, S. Delany‐Moretlwe^17^, on behalf of the HPTN 084 study team

^1^University of North Carolina at Chapel Hill School of Medicine, Chapel Hill, United States, ^2^UNC Project Malawi, Lilongwe, Malawi, ^3^Statistical Centre for HIV/AIDS Research and Prevention Fred Hutchinson Cancer Center, Seattle, United States, ^4^Johns Hopkins University School of Medicine, Department of Pathology, Baltimore, United States, ^5^University of Zimbabwe Clinical Trials Research Centre, Harare, Zimbabwe, ^6^Kisumu CRS, KEMRI, Kisumu, Kenya, ^7^Johns Hopkins Research Project, Kamuzu University of Health Sciences, Blantyre, Malawi, ^8^Makerere University ‐ Johns Hopkins University Research Collaboration, Kampala, Uganda, ^9^Desmond Tutu Health Foundation, University of Cape Town, Cape Town, South Africa, ^10^HIV and other Infectious Diseases Research Unit, South African Medical Research Council, Durban, South Africa, ^11^Botswana Harvard AIDS Institute Partnership, Gaborone, Botswana, ^12^Perinatal HIV Research Unit, University of the Witwatersrand, Johannesburg, South Africa, ^13^UVRI‐IAVI, Entebbe, Uganda, ^14^Desmond Tutu TB Centre, University of Stellenbosch, Stellenbosch, South Africa, ^15^Baylor College of Medicine Children's Foundation Uganda, Kampala, Uganda, ^16^Eswatini Prevention Center, ICAP at Columbia University Mailman School of Public Health, New York, United States, ^17^Wits RHI, University of the Witwatersrand, Johannesburg, South Africa, ^18^FHI 360, Durham, United States, ^19^Division of AIDS, National Institute for Allergy and infectious diseases, Rockville, United States, ^20^Gilead Sciences, Foster City, United States, ^21^ViiV Healthcare, Durham, United States, ^22^UCLA, Los Angeles, United States

**Background: **HPTN 084 showed that injectable cabotegravir (CAB) is effective for PrEP in women. HIV diagnosis in the context of PrEP use may be complicated by both false negative and false positive tests results. We evaluated the positive predictive value (PPV) of the HPTN 084 testing algorithm to guide HIV treatment initiation decisions in women on PrEP.

**Table:** 

**Abstract OALBX0202‐Table. PPV for initial reactive visits with adjudicated HIV status (162 total, 74 confirmed positive) by treatment arm from HPTN 084**						
Rapid test (All types)	HIV‐positive/ total reactive (CAB)	HIV‐positive/ total reactive (TDF/FTC)	PPV (95% CI) (CAB)	PPV (95% CI) (TDF/FTC	Difference in PPV (95% CI) (CAB vs. TDF/FTC)	Overall PPV (95% CI) (CAB and TDF/FTC)
Rapid Test (All types)	8/20	81/94	40% (19%,64%)	86% (78%, 92%)	‐46% (‐72%, ‐21%)	78% (69%, 85%)
Alere Determine (3^rd^ gen)	4/12	36/44	33% (10%,65%)	82% (67%, 92%)	‐48% (‐83%, ‐14%)	71% (58%, 83%)
OraQuick ADVANCE Rapid HIV‐1/2 (3rd gen)	4/5	40/44	Insufficient sample size	91% (78%, 97%)	Insufficient sample size	90% (78%, 97%)
Ag/Ab test	7/42	66/94	17% (7%,31%)	70% (60%, 79%)	‐54% (‐70%, ‐37%)	54% (45%, 62%)
Two reactive rapid tests	4/4	36/36	Insufficient sample size	100% (90%, 100%)	Insufficient sample size	100% (91%, 100%)
Any reactive rapid or Ag/Ab test	8/55	66/107	15% (6%, 27%)	62% (52%, 71%)	‐47% (‐62%, ‐33%)	46% (38%, 54%)

**Methods: **Site HIV testing included rapid tests (RT) and a lab‐based antigen/antibody (Ag/Ab) test. Any reactive test prompted study product hold and further HIV status evaluation. Final HIV status was adjudicated based on study site test results and a centralized laboratory. PPV (95% confidence intervals [CI]) for initial site‐based reactive visits were assessed for different testing algorithms compared to adjudicated results.

**Results: **Of 20 sites, 14 used two parallel RT and 6 used one RT. 3180 participants contributed 67314 HIV testing visits. Overall, 5% (162/3180) had 1 reactive test and 74/162 were adjudicated HIV positive by November 2022 (8 CAB, 66 TDF/FTC) while 86 participants did not acquire HIV. The PPV for any reactive RT or Ag/Ab test was 46% (CI: 38%, 54%). Two parallel reactive RT had a PPV of 100% (CI: 91%, 100%). Oraquick Advance as a single test had high PPV (90%, CI: 78%, 97%). Ag/Ab testing detected 73/74 HIV diagnoses but had low PPV (54%, CI: 45%, 62%). PPV of all tests assessed was higher in those randomized to TDF/FTC compared to CAB (Table 1).

**Conclusions: **In this study, two reactive RT were sufficient to confirm HIV diagnosis and recommend treatment initiation. With a single reactive HIV test and high frequency of false positive testing, PrEP programs should anticipate the need for further testing, counseling about false positivity, and plans to resume PrEP after excluding HIV. More data is needed to determine if additional testing may be required in the setting of CAB.

### OALBX0203 Initial PrEP product choice: results from the HPTN 084 open‐label extension

S. Delany‐Moretlwe ^1^, B. Hanscom^2^, F. Angira^3^, S. Dadabhai^4^, D. Gadama^5^, B. Mirembe^6^, M. Bhondai^7^, S. Innes^8^, D. Kalonji^9^, J. Makhema^10^, P. Mandima^7^, A. Marais^11^, J. Mpendo^12^, P. Mukwekwerere^7^, N. Mgodi^7^, V. Naidoo^13^, P. Nahirya Ntege^14^, H. Nuwagaba‐Biribonwoha^15^, E. Roos^1^, N. Singh^16^, B. Siziba^7^, E. Spooner^16^, J. Farrior^17^, S. Rose^17^, E. Piwowar‐Manning^18^, M. Burton^19^, L. Soto‐Torres^20^, J. Rooney^21^, A. Rinehart^22^, M. Cohen^23^, M. Hosseinipour^24,23^, HPTN 084 study team

^1^University of the Witwatersrand, Wits RHI, Johannesburg, South Africa, ^2^Fred Hutchinson Cancer Research Institute, Statistical Centre for HIV/AIDS Research Prevention, Seattle, United States, ^3^KEMRI, Kisumu, Kenya, ^4^College of Medicine, Blanytre, Blantyre, Malawi, ^5^UNC‐Project Malawi, Lilongwe, Malawi, ^6^Makerere University ‐ Johns Hopkins University Research Collaboration, Kampala, Uganda, ^7^University of Zimbabwe Clinical Trials Research Centre, Harare, Zimbabwe, ^8^Desmond Tutu Health Foundation, Cape Town, South Africa, ^9^South African Medical Research Council, HIV and other Infectious Diseases Research Unit, Durban, South Africa, ^10^Botswana Harvard AIDS Institute Partnership, Gabarone, Botswana, ^11^University of the Witwatersrand, Perinatal HIV Research Unit, Soweto, South Africa, ^12^UVRI‐IAVI, Entebbe, Uganda, ^13^University of Stellenbosch, Desmond Tutu TB Centre, Stellenbosch, South Africa, ^14^Baylor College of Medicine Children's Foundation Uganda, Kampala, Uganda, ^15^Columbia University Mailman School of Public Health,, Eswatini Prevention Center, Mbabane, South Africa, ^16^South African Medical Research Council, Durban, South Africa, ^17^FHI360, Durham, United States, ^18^Johns Hopkins University School of Medicine, Baltimore, United States, ^19^Fred Hutchinson Cancer Research Institute,, Seattle, United States, ^20^National Institute for Allergy and infectious diseases, Rockville, United States, ^21^Gilead Sciences, Foster City, United States, ^22^ViiV Healthcare, Durham, United States, ^23^University of North Carolina at Chapel Hill, Chapel Hill, United States, ^24^UNC Project‐Malawi, Lilongwe, Malawi

**Background: **HPTN 084 demonstrated that long‐acting injectable cabotegravir (CAB) was superior to daily oral TDF/FTC for HIV prevention in individuals born female. In 2022, following a protocol amendment, eligible participants were offered the choice of open‐label CAB or TDF/FTC as PrEP in an open‐label extension (OLE).

**Methods: **In HPTN 084 participants who were eligible for (n = 3028) and accepted OLE participation (n = 2472), we assessed initial PrEP choice and reasons. We used the Decisional Conflict Scale to measure perceptions of effective decision‐making (0 = no conflict and 100 = high decisional conflict). We compared participant demographic, behavioral and decision characteristics by initial product choice using chi‐squared tests.

**Results: **Of 2472 participants, 1931 (78%) chose CAB and 536 (22%) chose TDF/FTC. Among those initially randomized to TDF/FTC (n = 1219), 817 (67%) chose CAB with 177 (15%) choosing the oral lead‐in and 640 (53%) preferring direct‐to‐inject. Among those initially randomized to CAB (n = 1253), 131 (11%) chose TDF/FTC. Participants who chose CAB (n = 1931) preferred injections (77%), desired a convenient or discrete PrEP method (11%), valued CAB effectiveness (8%) or gave other/no reasons (4%). Those that chose TDF/FTC (n = 536) preferred pills (81%), feared injection pain or side effects (5%), desired pregnancy (1%) or efficient clinic visits (1%), or gave no reason (12%). Product choice varied by country (p<0.001). Participants who chose CAB were more likely to be sexually active but not live with partner (p<0.025), to have experienced recent physical intimate partner violence (p<0.013) and to have been paid for sex (0.002). Although overall decision conflict scores were low and similar between groups (CAB 14; TDF/FTC16; p = 0.9), effective decision sub‐score differences suggest CAB users perceived more strongly that they had made a good decision (CAB 6; TDF/FTC 13; p 0.011). While most participants (66%) reported their choice was their own, discussions with study staff (20%) or family and friends (11%) were also influential.

**Conclusions: **The majority of HPTN 084 participants chose CAB for PrEP. Product choice was influenced by personal preference for product attributes, participant risk behaviours and social context. Future PrEP programs will need to adopt strategies that align user values and preferences with product choices.

### OALBX0204 Racial disparities in HIV incidence and PrEP non‐adherence among gay and other men who have sex with men (MSM) and transgender women (TGW) using oral PrEP in Brazil: ImPrEP Study

L. Freitas ^1^, C. Coutinho^1^, J. Freitas^1^, T.A. Santos^1^, A. Alves^1^, M.S.T. Silva^1^, R.I. Moreira^1^, I. Leite^1^, B. Hoagland^1^, M. Benedetti^1^, C. Pimenta^2^, T.S. Torres^1^, B. Grinsztejn^1^, V.G. Veloso^1^, ImPrEP Study Group

^1^Instituto Nacional de Infectologia Evandro Chagas, Fundação Oswaldo Cruz, Rio de Janeiro, Brazil, ^2^Ministry of Health, Brasilia, Brazil

**Background: **Access to healthcare can impact PrEP uptake, adherence, and persistence, especially among racial and ethnic minorities. Black Brazilians historically have worse health outcomes compared to white Brazilians. We evaluated HIV incidence and factors related to PrEP non‐adherence among MSM and TGW from Brazil according to self‐reported race.

**Methods: **ImPrEP was a prospective, single‐arm, open‐label, implementation study of same‐day oral PrEP that enrolled 9509 MSM/TGW in Brazil, Mexico, and Peru (Feb/2018‐June/2021). For this analysis, we used data from Brazil (n = 3928 participants from 14 HIV/STI clinics in 11 cities). We calculated HIV incidence per 100 person‐years using the Poisson model for black, pardo (mixed‐race), and white races. We created two logistic regression models (black/pardo and white) to identify factors associated with PrEP non‐adherence (medication possession ratio (MPR)<0.6).

**Results: **From the 3928 enrolled, 47% were white, 36% pardo/mixed, and 15% black (Table 1).

HIV incidence rate was higher among black individuals [2.16(CI95%0.54‐8.63)] compared to pardo [1.49(CI95%0.48‐4.62)] and white [1.00(CI95%0.25‐4.01)]. Non‐adherence was higher among black (26.2%) compared to pardo (24.2%) and white (18.7%) participants. TGW and young participants presented higher odds of PrEP non‐adherence across all races. Among black/pardo participants, those with less education and transactional sex had higher odds of PrEP non‐adherence (Figure 1).

**Conclusions: **Higher HIV incidence and lower PrEP adherence among pardo and black individuals highlight race disparities and the impact of structural racism on health outcomes. The implementation of public policies to mitigate racial and social inequalities is urgent in Brazil, increasing equity in health access and promoting social justice.

**Figure:**
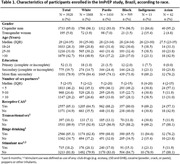
**Abstract OALBX0204‐Table**.

**Figure:**
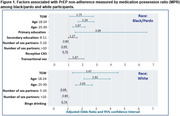
**Abstract OALBX0204‐Figure**.

### OALBX0205 Association of HIV pre‐exposure prophylaxis (PrEP) use and bacterial sexually transmitted infections (bSTI) among men who have sex with men (MSM) and transgender women (TGW) in HVTN 704/HPTN 085

J.A. Gallardo‐Cartagena ^1^, P. Hunidzarira^2^, K. Gillespie^3^, M. Juraska^3^, M. Neradilek^3^, P. Gilbert^3^, R. Cabello^4^, J.R. Lama^5^, J. Valencia^5^, J.C. Hinojosa^6^, J.J. Montenegro‐Idrogo^1^, V.G. Veloso^7^, B. Grinsztejn^7^, R. De La Grecca^3^, S. Karuna^3,8^, S. Edupuganti^9^, K. Konda^10^, A. Roxby^3,11,12^, M. Cohen^13^, L. Corey^3^, J. Sanchez^1^, HVTN 704/HPTN 085 Study Team

^1^Universidad Nacional Mayor de San Marcos, Centro de Investigaciones Tecnológicas, Biomédicas y Medioambientales, Callao, Peru, ^2^University of Zimbabwe, Clinical Trials Research Centre (UZ‐CTRC), Harare, Zimbabwe, ^3^Fred Hutchinson Cancer Center, Vaccine and Infectious Diseases Division, Seattle, United States, ^4^Asociación Civil Vía Libre, Lima, Peru, ^5^Asociación Civil Impacta Salud y Educación, Lima, Peru, ^6^Asociación Civil Selva Amazónica, Lima, Peru, ^7^Instituto Nacional de Infectologia Evandro Chagas, Fundação Oswaldo Cruz (INI/Fiocruz), Rio de Janeiro, Brazil, ^8^GreenLight Biosciences, Inc, Lexington, United States, ^9^Emory University, Department of Medicine, Division of Infectious Disease, Atlanta, United States, ^10^University of Southern California, Keck School of Medicine, Department of Population and Public Health Sciences, Los Angeles, United States, ^11^University of Washington, Department of Medicine, Seattle, United States, ^12^University of Washington, Department of Global Health, Seattle, United States, ^13^University of North Carolina, Department of Medicine, Chapel Hill, United States

**Background: **HIV prevention trials enroll participants with high vulnerability to HIV and provide access to an enhanced HIV prevention package, including PrEP. HVTN 704/HPTN 085 was a randomized clinical trial evaluating VRC01 for HIV prevention in MSM and TGW in the Americas and Switzerland. We conducted a post‐hoc analysis to characterize bSTI burden and evaluate the association of PrEP use with bSTI incidence among trial participants.

**Methods: **We included trial participants who received at least one VRC01/placebo infusion and had bSTI results from at least one bSTI visit (baseline and every 6 months). Participants received education about oral PrEP and could opt to use it. Prevalence of bSTI was summarized at each visit and categorized by socio‐demographics, geographic region, and PrEP use (a time‐varying covariate indicating self‐reported PrEP use). We estimated incidence rates (for 100 person‐years at risk [PYR]) of first bSTI occurrence among those who were negative at baseline, categorized by PrEP use. Cox proportional hazards models were used to evaluate the effect of PrEP use on bSTI incidence, additionally adjusted for age, region, race, ethnicity, and VRC01/placebo.

**Table:** 

**Abstract OALBX0205‐Table**							
	Baseline prevalence summarized by n/N (%)				Incidence rates for 100 PYR (95%CI)		
bSTI	Overall	Brazil	Peru	US/Switzerland	Overall	While on PrEP	While not on PrEP
Any bSTI	749/2687 (27.9%)	55/150 (36.7%)	392/1124 (34.9%)	302/1413 (21.4%)	28.6 (26.4‐31.0)	37.2 (33.0‐41.8)	24.0 (21.5‐26.7)
Chlamydia	297/2687 (11.1%)	11/150 (7.3%)	158/1124 (14.1%)	128/1413 (9.1%)	16.1 (14.5‐17.8)	20.4 (17.5‐23.7)	13.5 (11.7‐15.5)
Gonorrhea	273/2687 (10.2%)	22/150 (14.7%)	151/1124 (13.4%)	100/1413 (7.1%)	12.2 (10.9‐13.7)	16.2 (13.7‐19.1)	9.9 (8.4‐11.6)
Syphilis	353/2687 (13.1%)	34/150 (22.7%)	194/1124 (17.3%)	125/1413 (8.8%)	6.7 (5.7‐7.8)	8.8 (7.0‐11.0)	5.5 (4.4‐6.8)

**Results: **The analysis included 2687 participants, of whom 31.8% initiated PrEP during follow‐up. The table summarizes bSTI baseline prevalence by region and incidence rates by PrEP use. The baseline prevalence of any bSTI was highest among <20yo (35.7%), Hispanic/Latinx (32.4%), non‐Black/non‐Whites (32.1%), TGW (35.3%), and MSM (28.9%). Oropharyngeal (gonorrhea 6.5%) and rectal bSTI were more prevalent (chlamydia 9.0% and gonorrhea 5.8%) than genitourinary bSTI (chlamydia 3.0% and gonorrhea 0.9%) at baseline. PrEP use was significantly associated with increased hazard of any bSTI (HR 1.7, 95%CI 1.4‐2.1), chlamydia (HR 1.7, 95%CI 1.3‐2.2), gonorrhea (HR 1.8, 95%CI 1.4‐2.5), and syphilis (HR 1.9, 95%CI 1.3‐2.8).

**Conclusions: **HVTN 704/HPTN 085 engaged communities with significant HIV/STI vulnerability. While on PrEP, users had higher rates of bSTI, suggesting risk compensation and underscoring the need for advancing bSTI testing and prevention measures in HIV prevention trials.

## AUTHOR INDEX

### A

Abade Bello, R. OAE0404

Abara, W. OAC0203

Abdulkadir, I. OAC0405

Abela, I. OALBB0505

Aboobacar, A. OALBD0605

Abu‐Ba'are, G. OAE0502

Abuna, F. OAC0402, OAC0403

Achanya, A. OAB0105

Adamu, I. OAE0404

Adedimeji, A. OAD0203

Adeoye, A. OAB0105

Adisa, O. OAE0404

Adland, E. OALBX0104

Afifi, A. OAA0403

Agius, P. OAC0204

Agot, K. OALBX0103

Agu, F. OAB0105

Agu, K. OAB0105

Agwuocha, C. OAE0404

Agyei, Y. OALBX0202

Ahmed, A.R. OAE0302

Ahmed, A.Y. OAE0404

Akanmu, M.‐M. OAE0404

Akpan, U. OAB0105

Akpomiemie, G. OAB0204, OALBB0504

Akrong, R. OAE0502

Alatiit, G.‐J. OAE0505

Alexandrova Ezerska, L. OAB0302

Allison, C. OALBA0503

Altice, F.L. OAD0402

Altman, J.D. OAA0202

Alves, A. OALBX0204

Amara, R.R. OAA0202

Amoh‐Otu, R.P. OAE0502

Amuri, M. OAE0403

Anderson, D. OALBB0502

Anderson, J. OALBA0503

Anecho, A. OAC0302

Anene, E. OAD0102

Angelovich, T. OAA0103

Angira, F. OALBX0202, OALBX0203

Ani, I. OAB0105

Anis, A. OAC0202

Anoubissi, J.d.D. OAC0405, OAD0103, OAD0504

Anthony, S. OAE0203

Appa, A. OAB0405

Appiah, P. OAE0502

Arandjelovic, P. OALBA0503

Archary, M. OALBX0104

Aristegui, I. OAD0205

Arneson, D. OAA0305

Arora, P. OAB0104

Arrais Pinto Jr., J. OAC0503

Arriola, E. OAD0505

Artenie, A. OAC0103

Arunmanakul, A. OAE0505

Asante‐Appiah, E. OALBX0102

Asewe, M. OAE0102, OAE0105

Aspiras, A. OALBE0602

Asselin, J. OAC0204

Atkins, K. OAC0203

Audet, C.M. OALBD0605

Avettand‐Fenoel, V. OALBA0504

Avihingsanon, A. OAB0104, OAB0205, OALBC0605

Axthelm, M. OAA0205

Ayeh, E. OAD0504

Ayieko, B. OALBX0103

Azania, A. OAE0404

### B

Baeten, J. OAC0403, OAC0502

Baggaley, R. OALBC0602

Ballesteros, Á. OAB0103

Bandason, T. OAE0204

Banerjee, P. OAE0102

Bansi‐Matharu, L. OALBB0505

Bara, H.T. OALBE0604

Baral, S. OAC0203, OAC0405, OAD0103, OAD0504

Barr‐DiChiara, M. OALBC0602

Barreto, M. OAC0303

Barriga, M. OAD0104

Bateganya, M. OAC0302

Bavinton, B.R. OAC0305, OAC0505, OAE0103

Bayramoglu, Y. OAD0204

Bazira, D. OAD0502

Beca, L. OAB0103

Begovac, J. OAB0402

Beima‐Sofie, K. OAE0405

Bekker, L.‐G. OAE0302

Beloor, J. OAA0203

Benedetti, M. OALBX0204

Bengu, N. OALBX0104

Benito, J.P. OAE0504, OALBE0602

Benjarattanaporn, P. OAC0105

Berghammer, E. OAE0104

Bernays, S. OAD0302

Bertagnolio, S. OALBC0604

Bessonov, S. OAC0405

Beyrer, C. OAD0504

Bhondai, M. OALBX0203

Bhoola, R. OALBX0104

Biasin, M. OAA0105

Bingham, T. OAC0304

Birungi, J.B. OAE0402

Bissa, M. OAA0402

Bissio, E. OALBB0505

Blick, G. OAB0202

Bloch, M. OAC0204

Boakye, F. OAE0502

Boeke, C.E. OAE0404

Boettiger, D.C. OAC0105

Bogner, J.R. OALBX0102

Bondoc, S. OAA0103

Bongomin, P. OAD0502

Bonongwe, N. OAC0205

Boonruang, J. OAC0404, OALBE0605

Boonyapisomparn, H. OAE0103

Bosch, B. OAB0204, OALBB0504

Bosinger, S.E. OAA0103, OAA0202

Bosque, A. OALBA0502

Bourne, A. OALBD0602

Bousmah, M.‐a.‐Q. OALBB0504

Bowonwatanuwong, C. OALBC0605

Bramugy, J. OAB0103

Brandes, V. OAB0402

Braun, D. OAB0203

Brew, B. OAA0103

Briñes, R.M. OAE0504

Brines, M. OAE0504

Brion, S. OAC0405, OAD0103, OAD0504

Broady, T.R. OAC0305, OAC0505, OALBD0602

Bromberg, D.J. OAD0402

Brooks, K. OALBE0603

Brown, N.E. OALBD0603

Brummel, S.S. OAB0404

Bruxvoort, K. OAC0503

Buck, W.C. OAB0103

Bukusi, E.A. OAE0102, OAE0105, OALBX0103

Burchell, A.N. OAC0202

Burger, D. OAB0103

Burgess, D. OALBA0505

Burton, M. OALBX0203

Busman‐Sahay, K. OAA0103

Butte, A. OAA0305

Bwakura Dangarembizi, M. OALBB0503

Bwakura, M. OAB0103

Byambaa, C. OAE0203

Byarugaba, H. OAD0502

Byonanebye, D.M. OALBB0505

Byrnes, S. OAA0103

### C

Caballero, R. OAD0205

Cabello, R. OALBX0205

Cadelina, J. OAD0505

Cahn, P. OALBX0102

Cahn, S. OAD0205

Calmy, A. OALBA0504, OALBB0504

Camlin, C.S. OALBX0103

Canagasabey, D. OAE0202

Cao, T.K. OAD0404

Cappelletti, G. OAA0105

Cardozo, N. OAD0205

Cardozo, T. OAA0402

Carpino, T. OAC0203

Cassell, M. OAE0103

Castagna, A. OALBB0505

Cerreta, E. OAB0202

Chabala, C. OAB0103, OALBB0503

Chahroudi, A. OAA0202, OALBA0505

Chaillon, A. OAA0104, OAA0303

Chaisalee, T. OAE0505, OALBE0605

Chambers, C. OAC0202

Chan, C. OAC0305, OAC0505, OAE0103

Chandiwana, N. OAB0204

Chapel, A. OALBA0504

Charaf, S. OAA0404

Charlebois, E.D. OALBX0103

Chateau, M. OAA0102

Chauke, H. OALBC0603

Chen, J. OAC0205

Chen, T.‐C. OAB0304

Chen, X. OAA0404

Cheng, C. OAA0404

Cheng, C.‐Y. OAB0304

Chhaganlal, K.D. OAB0103

Chicumbe, S. OAE0304

Chinniah, K. OALBX0104

Chisenga, M. OAB0403

Chitsamatanga, M. OAB0103

Chitukuta, M. OALBC0603

Chkhartishvili, N. OAB0402

Chomchey, N. OAB0303

Chomont, N. OALBA0502

Chonta, M. OAE0502

Choong, A. OALBC0602

Chow, E. OALBC0602

Chu, C. OAD0505

Chukwukere, U. OAA0204

Churchill, M. OAA0103

Ciaranello, A. OAE0302

Clark, L.M. OAA0304

Clay, S. OAE0502

Clemmer, D.C. OAA0304

Clifton, B. OAC0505

Cobos, M. OAD0305

Cochrane, C. OAA0103

Coffin, P. OAB0405

Cohen, C. OAB0402, OALBB0505

Cohen, M.S. OALBX0202, OALBX0203, OALBX0205

Colbers, A. OAB0103

Colby, D.J. OAB0303

Colombrita, C. OAA0105

Comulada, S. OAC0504

Connick, E. OAA0303

Cooney, J. OALBA0503

Cooper, C.L. OAC0202

Copertino, D. OAA0204

Corey, L. OAA0205, OALBX0205

Cornelisse, V. OALBD0602

Correll, T.A. OAB0203, OALBX0102

Corrigan, A.R. OAA0404

Cosenza, A. OAB0202

Costiniuk, C.T. OAC0202

Cotugno, N. OALBX0104

Coutinho, C. OALBX0204

Couto, A. OAE0304

Cressey, T.R. OAC0404

Cromhout, G. OALBX0104

Cross, S. OAE0303

Cruz, D. OAE0504, OALBE0602

Cumbe, V. OALBD0605

Cunningham, D. OALBX0102

### D

d'Arminio Monforte, A. OAB0402, OALBB0505

Dadabhai, S. OALBX0202, OALBX0203

Damle, M. OALBE0603

Danesh, A. OAA0204

Dannay, R. OAA0103

Dashti, A. OALBA0505

Dauya, E. OAE0204

Davies, C. OAC0104

Davies, M.‐A. OAC0104

Davis, M. OAB0104

Ddaaki, W. OAD0503

De La Grecca, R. OALBX0205

De Schacht, C. OALBD0605

De Wit, S. OAB0402, OALBB0505

Deeks, S. OAA0302, OAA0305

Dela Cruz, J.D.M. OALBE0602

Delaney, K. OAC0203

Delany‐Moretlwe, S. OALBX0202, OALBX0203

Delaporte, E. OALBB0504

Deleage, C. OAA0103

Delpech, V. OALBD0602

Demeke, H.B. OAC0304

Dendup, T. OAE0203

Dettinger, J. OAC0402, OAC0403

Dhakwa, D. OAD0105

Diaz, J. OALBC0604

Dinglasan, J.l. OAE0504

Djomand, G. OAC0304

Dlamini, N. OAD0303

Dlamini, P. OAD0502

Doerflinger, M. OALBA0503

Doerholt, K. OALBB0503

Doherty, M. OAB0302, OALBC0604

Dokla, K.A. OAC0405, OAD0504

Domingo, R. OAE0504

Dominguez‐Rodríguez, S. OAB0103

Donohoe, T. OALBE0603

Donovan, B. OAC0204

Doria‐Rose, N.A. OAA0404

Doster, M. OAA0402

Dourado, I. OAC0303, OAC0503, OAD0405

Doyle, J. OAC0204

Dross, S. OAA0205

Duarte, M. OAD0205

Duell, D. OAA0205

Dukakia, N. OALBB0503

Dunaway, K. OAC0405, OAD0103, OAD0504

Dvory‐Sobol, H. OAB0205

Dzavakwa, N. OAB0403

Dziva Chikwari, C. OAE0204

### E

Easley, K.A. OAA0202

Ediau, M. OAC0302

Edupuganti, S. OALBX0205

Edwards, O.W. OAC0203

Eichholz, K. OAA0205

El‐Hayek, C. OAC0204

Engelman, K. OAA0202

Eschliman, E. OAC0203

Eshleman, S. OALBX0202

Estes, J. OAA0103

Etima, J. OALBC0603

Etyang, L. OAC0502

Euvrard, J. OAC0104

Eves, K. OALBX0102

Eyo, A. OAB0105

Ezissi, C. OAB0105

### F

Fabian, S. OAD0205

Fahey, C.A. OALBX0105

Fairley, C. OAC0204, OALBC0602

Fang, L. OAB0202

Farrior, J. OALBX0202, OALBX0203

Fei, I. OAD0202

Fernandes, O. OAE0404

Fernandez, A. OAD0203

Ferrand, R.A. OAB0403, OAE0204

Ferrari, G. OALBA0505

Feser, M. OAE0305

Figueroa, M.I. OAD0205

Fillis, R. OALBX0104

Filteau, S. OAB0403

Fitzer, J. OAB0302

Flanagan, C.F. OAE0302

Florence, E. OAB0402

Folkvord, J.M. OAA0303

Fontas, E. OAB0402, OALBB0505

Ford, N. OAE0305, OALBC0604

Fowokan, A. OAC0202

Fox, M.C. OAB0203

Fox, M.P. OAC0104

França, M. OAD0405

Franchini, G. OAA0402

Franks, J. OAE0403

Fraser, D. OAE0103

Freedberg, K. OAE0305

Freedberg, K.A. OAE0302

Freitas, J. OALBX0204

Freitas, L. OALBX0204

Frola, C. OAD0205

Fromentin, R. OALBA0502

Frontini, E. OAD0205

Frouard, J. OAA0104, OAA0305

Fukazawa, Y. OAA0205

Fuller, D. OAA0205

### G

Gómez, L. OAC0403

Gadama, D. OALBX0202, OALBX0203

Gakuo, S. OAE0102

Gallardo‐Cartagena, J.A. OALBX0205

Gandhi, M. OALBX0103

Garcia De Leon Moreno, C. OAC0405, OAD0103

Garcia, J.V. OAA0102

Gardiennet, E. OALBA0504

Garner, S. OALBA0503

Garziano, M. OAA0105

Gaspar, I. OAE0304

Gatanaga, H. OALBX0102

Gatechompol, S. OALBC0605

Genberg, B. OAD0504

George, A. OAA0104

Ghosn, J. OALBX0102

Gianella, S. OAA0104, OAA0303

Giaquinto, C. OALBX0104

Gibb, D.M. OALBB0503

Gichuchi, B. OAC0304

Gichuhi, S. OAB0305

Gilbert, P. OALBX0205

Gill, K. OAA0202

Gillespie, K. OALBX0205

Girish, S. OAB0104

Godlevskaya, M. OAC0405

Gomez, L. OAC0402

Gottert, A. OAD0203

Goulder, P. OALBX0104

Govender, K. OALBX0104

Govindu, V. OAA0202

Grabmeier‐Pfistershammer, K. OALBB0505

Grabowski, M.K. OAD0503

Gramatica, A. OAA0204

Grandhi, A. OAB0203

Grangeiro, A. OAC0503, OAD0405

Granger, K. OAE0202

Graves, E. OALBD0605

Gray, R. OAC0102

Greco, D. OAC0503

Green, K.E. OAD0404, OAE0103, OAE0202

Greenberg, L. OAB0402

Greene‐Cramer, B.J. OAB0302

Gregson, C.L. OAB0403

Grennan, T. OAC0202

Grinsztejn, B. OALBX0204, OALBX0205

Groh, A. OAB0402

Groll, A. OALBX0104

Grulich, A. OAC0102

Guillen, J. OAD0104

Gutin, S.A. OALBX0103

Gutowska, A. OAA0402

Guy, R. OAC0102, OAC0204

Gyamerah, E. OAE0502

### H

Haeseleer, F. OAA0205

Hagins, D.P. OALBB0502

Haire, B. OALBD0602

Hamblion, E. OAB0302

Hamilton, E. OALBC0603

Hammermeister Nezu, I. OAB0302

Hanley, S. OAB0404

Hannah, M. OAC0203

Hanscom, B. OALBX0202, OALBX0203

Harada, S. OAA0405

Harkey, K. OAE0102, OAE0105

Harney, B. OAC0204

Haumba, S. OAD0502

Heany, S. OAB0102

Heath, K. OAE0303

Heffron, R. OAE0405

Hellard, M. OAC0204

Hentzien, M. OALBA0504

Hera, C. OALBE0604

Herbert, N. OALBX0104

Herbst, C. OAD0303

Hernández, J.S. OAD0305

Hii, I.‐M. OAB0304

Hill, A. OAB0204, OAE0303, OALBB0504

Hinestrosa, F. OALBX0102

Hinojosa, J.C. OALBX0205

Hiransuthikul, A. OAC0404

Hlomewoo, K.A. OAC0405, OAD0504

Hlongwane, S. OAD0303

Hoagland, B. OALBX0204

Hoang, T.N. OAC0304

Hoare, J. OAB0102

Hoffman, I. OAC0205

Hoffmeister, S. OAA0205

Hoh, R. OAA0305

Holt, M. OAC0305, OAC0505, OALBD0602

Hongchookiat, P. OAC0404

Horsburgh, R. OAE0305

Horvath, S. OAB0102

Hosein, S.R. OALBB0505

Hosek, S. OALBC0603

Hosseinipour, M. OAC0205, OALBX0202, OALBX0203

Howard, J. OALBA0502

Hoxha, A. OAB0302

Hsu, D. OAB0303

Hsu, T.‐H. OALBB0502

Huang, H. OAB0104

Humeau, Z. OAD0404

Hung, C.‐C. OAB0304

Hunidzarira, P. OALBX0202, OALBX0205

Hyle, E. OAE0305

### I

Ichihara, M. OAC0303

Idemudia, A. OAB0105

Ihekanandu, U. OAE0503

Ijeoma, U. OAC0304

Ikpeazu, I. OAE0404

Ilika, F. OAE0503

Innes, S. OALBX0202, OALBX0203

Inzaule, S. OALBC0604

Isabirye, D. OAD0503

Ishii, H. OAA0405

Ivasiy, R. OAD0402

Ivy, III, W. OAC0304

### J

Jacobs, T. OAB0103

Jaffar, S. OAE0402

Jalloh, M. OAE0403

James, E. OAB0105

Jamil, M. OALBC0602

Janamnuaysook, R. OAC0404, OALBE0605

Janjua, N.Z. OAC0202

Jankie, T. OAC0103

Janssens, J. OAA0302

Janyam, S. OAE0505, OALBE0605

Jaschinski, N. OAB0402, OALBB0505

Jassat, W. OALBC0604

Jenkins, T. OAA0103

Jere, E. OAC0205

Jesus, G. OAC0303

Jin, E.Y. OAE0302

Jin, L. OAA0205

Jirajariyavej, S. OALBC0605

John‐Stewart, G. OAC0402, OAC0403

Johnson, C. OALBC0602

Johnson, L.F. OAC0104

Jones, B. OAA0204

Jones, R. OAA0403

Joseph, O. OAE0403

Junio, P. OAE0504

Juraska, M. OALBX0205

### K

Kaasik‐Aaslav, K. OAB0302

Kacheyo, M. OAC0205

Kadiyala, G.N. OAA0302, OAA0305

Kadziyanhike, G. OAC0405, OAE0205

Kadziyanike, G. OAE0205

Kagaayi, J. OAD0503

Kahemele, J. OAE0403

Kahn, F. OAA0204

Kakooza, L. OAB0103

Kalichman, S.C. OAC0302

Kalonji, D. OALBX0202, OALBX0203

Kama, J. OAE0404

Kaneda, D. OAA0405

Kangethe, J. OAB0305

Kannie, S. OALBX0104

Kapongo, C. OALBX0104

Kappes, J. OALBX0104

Karuna, S. OALBX0205

Karunakaran, K.A. OAA0202

Kasonka, L. OAB0403

Kassim, S. OALBX0102

Katono, R.L. OAD0503

Kayode, Y.I. OAA0304

Kemigisha, D. OALBC0603

Kennedy, C.E. OAD0503

Kerr, S. OAB0302, OAC0404, OALBC0605

Khoza, N. OALBC0603

Kibwana, S. OAD0502

Kiem, H.‐P. OAA0205

Kiene, S.M. OAC0302

Kigozi, G. OAD0503, OAD0503

Kikobye, P. OAE0202

Kim, A.J. OAB0203

Kim, P. OAA0302

Kim, S.J. OAA0302

Kim, Y. OALBA0503

King, H. OALBA0505

King, J. OAC0102

Kinuthia, J. OAC0402, OAC0403

Kityo Mutuluza, C. OALBB0503

Kivuyo, S. OAE0402

Klopfer, S.O. OAB0203, OALBX0102

Ko, N.Y. OAD0505

Koenig, E. OAB0104

Konda, K. OALBX0205

Kouanfack, C. OALBB0504

Koup, R. OAA0404

Kranzer, K. OAB0403, OAE0204

Krishna, M. OALBX0104

Kroch, A. OAC0202

Kumar, P. OAA0203

Kusen, M. OAE0203

Kussainova, A.Z. OAD0402

Kwach, B. OAE0105

Kwan, A. OAD0202

Kwena, Z.A. OALBX0103

Kwong, G.A. OAA0202

Kwong, P. OAA0404

### L

Labriola, C. OAA0205

Laker Agnes Odongpiny, E. OAE0405

Lalak, K. OAC0405, OAD0103, OAD0504

Lama, J.R. OALBX0205

Lancaster, K. OALBD0602

Landovitz, R. OALBX0202

Lapp, S.A. OAA0202

Lariat, J. OAD0302

Laurenson‐Schafer, H. OAB0302

Lauscher, D. OAC0202

Law, M. OAB0402, OALBB0505

Le Polain de Waroux, O. OAB0302

Le, T.M. OAD0404

Lee, J. OALBB0502

Lee, M.Y.‐H. OAA0202

Lee, N.‐Y. OAB0304

Lee, S. OAA0305

Lee, Y.‐T. OAB0304

Leite, I. OALBX0204

Lertpiriyasuwat, C. OAC0105, OALBC0605

Leuschner, S. OAD0403

Levine, A. OAB0102

Levinger, C. OALBA0502

Lewin, S. OALBA0503

Lewis, R. OAB0302

Lewis‐Kulzer, J. OALBX0103

Leyre, L. OAA0204

Li, G. OAA0202

Liang, S. OAA0202

Liao, B. OAA0102

Liberman, A.R. OAD0402

Lifson, J. OALBA0505

Lim, N. OALBX0104

Limanaqi, F. OAA0105

Lin, C.‐D. OAC0302

Lin, C.‐Y. OAB0304

Lin, K.‐Y. OAB0304

Lin, L. OAB0104

Lin, M.C. OAA0202

Ling, L. OAA0102

Liou, B.‐H. OAB0304

Logie, C. OAE0502

Long, K. OAB0405

Looze, P. OAC0405, OAD0103, OAD0504

Louder, M. OAA0404

Lu, P.‐L. OAB0304

Lua, I. OAC0303

Lucas Fonseca, C. OALBD0605

Lufadeju, F. OAE0404

Lugemwa, A. OALBB0503

Lujintanon, S. OALBE0605

Lungu, J. OALBB0503

Luo, D. OAD0202

Luo, X. OAA0104, OAA0305

Lyn, A. OAC0304

Lyons, C. OAC0405, OAD0103, OAD0504

Lyu, Y.M. OALBC0602

### M

Ma, J. OAA0202

Ma, T. OAA0104

Mabuda, H.B. OAB0403

Macalalag, A.L. OAD0505

Macdonald, V. OALBC0602

MacGibbon, J. OAC0305, OAC0505, OALBD0602

Macinko, J. OAC0303

Madden, L.M. OAD0402

Mademutsa, C. OAE0205

Madrid, L. OAB0103

Madzima, B. OAD0105

Mafaune, H.W. OAD0105

Mafukidze, A. OAD0502

Magesa, D. OAE0403

Magnani, D. OAA0202

Magno, L. OAC0303, OAC0503, OAD0405

Mahaka, I. OALBE0604

Mahar, E.A. OAA0202

Mahaso, M. OAC0103

Mahiti, M. OAE0403

Maina, G. OAC0502

Maina, S. OAC0502

Maiyo, A. OAB0305

Majongosi, J. OAD0403

Makhema, J. OALBX0202, OALBX0203

Makumbi, S. OALBB0503

Maltez, F. OALBB0505

Malyshev, Y. OALBD0604

Mamba, L. OAD0502

Mamez, A.‐C. OALBA0504

Mancini, G. OAB0202

Mandima, P. OALBX0202, OALBX0203

Manickam, C. OAA0403

Mankattah, E. OAE0502

Manyanga, P. OALBE0604

Mao, L. OAC0305, OAC0505

Maokola, W. OALBX0105

Maphosa, T. OAE0305

Marais, A. OALBX0202, OALBX0203

Marathe, D. OAB0104

Martínez, A. OAD0305

Martínez, S. OAD0305

Martínez‐Picado, J. OALBA0504

Martin, H. OAB0104

Martinez, D. OAD0104

Martinez‐Picado, J. OALBX0104

Maruyama, H. OAE0403

Marwa, M. OAC0402, OAC0403

Mascola, J.R. OAA0404

Masedza, J. OAE0104

Mason, R. OALBA0505

Matambanadzo, K. OALBC0603

Matano, T. OAA0405

Matharu, L.B. OAB0402

Mathiya, E. OAC0205

Matoga, M. OAC0205

Matthews, G. OAC0204

Matyushina, D. OAC0405, OAD0103

Mavhu, W. OAD0302

Mavigner, M. OAA0202

Mavudze, J. OAD0403

Mazunda, D. OAE0104

Mbundure, R. OAD0302

McBrien, J. OALBA0505

McCann, K. OAB0204, OALBB0504

McComsey, G.A. OAB0203

McCoy, S.I. OALBX0105

McGowan, I. OAA0102

McGregor, S. OAC0102

McHugh, G. OAB0403

Mchunu, G. OAD0502

Mchunu, N. OALBX0104

Mckee, K. OAA0404

Mcllleron, H. OALBB0503

McMahan, V. OAB0405

McMahon, J. OALBA0503

McManus, H. OAC0102

McNaughton, A.L. OAC0103

McNulty, A. OALBD0602

Medina, M. OAA0205

Melard, A. OALBA0504

Meng, S. OAD0202

Menzies, N.A. OAC0302

Mereles Costa, E. OALBA0504

Merrigan, M. OAE0203

Metcalf, T.P. OAB0302

Metz, A.M. OAA0202

Mgodi, N. OALBX0202, OALBX0203

Mhlanga, S. OAD0502

Michalak, M. OAC0304

Middleton, D. OALBE0603

Miller, I. OAA0204

Mills, A.M. OAB0203

Mills, S. OAE0505

Mirchandani, M. OAB0204, OAE0303, OALBB0504

Mirembe, B. OALBX0203

Mirembe, B.B. OAB0302

Mirembe, B.G. OALBX0202

Mitchell, B.I. OAA0303

Miyauchi, K. OAA0405

Mngqibisa, R. OAB0203

Mnyippembe, A. OALBX0105

Mocroft, A. OALBB0505

Moga, T. OAD0403

Mogere, P. OAE0102

Moiana Uetela, D. OAE0304

Molina, J.‐M. OAB0203, OAB0205, OALBX0102

Monceaux, V. OALBA0504

Monin, J. OAD0304

Montenegro‐Idrogo, J.J. OALBX0205

Moodley, D. OAB0404

Moody, J. OALBX0103

Moolla, H. OAC0104

Mopuri, R. OAA0202

Moraleda, C. OAB0103

Moreira, R.I. OALBX0204

Moreno, C.d.L. OAD0504

Morris, B. OAE0203

Mota, T. OAA0204

Moyo, I. OAD0403

Moyo, P. OALBE0604

Mpande, Z. OAC0205

Mpendo, J. OALBX0202, OALBX0203

Mpoudi‐Etame, M. OALBB0504

Mrema, S. OAE0403

Msalilwa, A. OAE0403

Msasa, J. OALBX0105

Msongole, B. OAE0403

Muchemwa, C.P. OALBE0604

Mudhuma, T. OAD0302

Mudokwani, F. OAD0105

Mudzengerere, F.H. OAD0105

Mugo, N. OAC0502

Mugwanya, K. OAC0502

Mujugira, A. OAE0405

Mujuru, H. OAB0403

Mujuru, H.A. OAB0103, OALBB0503

Mukenyi, E. OAC0402

Mukwekwerere, P. OALBX0202, OALBX0203

Mulenga, V. OALBB0503

Mumbiro, V. OAB0103

Munishi, O. OAE0403

Munjoma, M. OAD0403

Munthali, T. OAC0205

Murane, R. OAA0205

Mureithi, M. OAB0305

Murnane, P. OALBE0603

Murray, S. OAC0203

Murungu, J. OALBE0604

Musiime, V. OAB0103, OALBB0503

Musinguzi, J. OAE0202

Musoro, G. OALBB0503

Mussini, C. OAB0402, OALBB0505

Mutai, K. OAB0305

Mutede, B. OAD0403

Mutesi, S. OAB0103

Mutseta, M. OAD0403

Mutsinze, A. OAD0302

Muwonge, T.R. OAE0405

Muzondo, K. OAD0105

Mwamabazi, M. OALBB0503

Mwanda, K. OAE0104

Mwangi, D. OAC0502

Mwangi, M. OAC0502

Mwareka, T. OAD0504, OAE0205

Myer, L. OAB0102

### N

N'guessan, K. OAA0402

Nabakka, P. OAD0503

Nabergoj, M. OALBA0504

Nahirya Ntege, P. OALBX0202, OALBX0203

Naidoo, M. OAB0404

Naidoo, V. OALBX0202, OALBX0203

Naigino, R. OAC0302

Nakamura‐Hoshi, M. OAA0405

Nakigozi, G. OAD0503

Nakitende, M. OAE0405

Nakubulwa, R. OAD0503

Nakyanjo, N. OAD0503

Nakyanzi, A. OAE0405

Nalugoda, F. OAD0503

Nalumansi, A. OAE0405

Nalwanga, D. OAB0103

Namakoola, I. OAE0402

Nambi, E. OALBB0503

Namuzizya, N. OAB0103

Nazzinda, R. OALBB0503

Ncube, G. OAD0403

Ndalama, B. OAC0205

Ndarukwa, V. OAB0302

Ndebele, W. OALBB0503

Ndhlovu, L.C. OAA0303

Ndumbi Ngamala, P. OAB0302

Nduna, B. OAB0103, OALBB0503

Ndung'u, T. OALBX0104

Ndungile, Y. OALBX0105

Neary, J. OAC0403

Neesgaard, B. OAB0402

Neidleman, J. OAA0104

Neilan, A.M. OAE0302

Nekorchuk, M. OAA0103

Nelson, L.E. OAE0502

Nelson, R. OAC0304

Nemli, S.A. OAD0204

Neradilek, M. OALBX0205

Newman, C. OALBD0602

Ngo, T. OAD0404

Ngumbau, N. OAC0402

Ngumbau, N.M. OAC0403

Ngure, K. OAC0502, OAE0102, OAE0105

Nguyen, D. OAD0404

Nguyen, H.T. OAD0404

Ngwenya, N. OAD0303

Nhando, N. OAD0403

Nhapi, A. OAE0205

Nijhuis, M. OALBA0504

Nishimura, H. OALBX0103

Njau, P.F. OALBX0105

Nkhata, H. OAC0205

Nonenoy, S. OAC0404

Norbu, J. OAE0203

Nsubuga, R. OAE0405

Nuño, N. OAD0305

Nuwagaba‐Biribonwoha, H. OALBX0202, OALBX0203

Nwagbo, C. OAD0102

Nwanja, E. OAB0105

Nwobi, O. OAE0404

Nyakuwa, S. OALBE0604

Nyampurev, G. OAE0203

Nyamwanza, B. OAD0105

Nyarota, K. OALBX0202

Nyblade, L. OAE0502

Nyirenda, N. OAC0205

Nzove, E. OAC0403

### O

Ocampo, F. OAB0303

Ochieng, B. OAC0402, OAC0403

Ochsenbauer, C. OALBX0104

Ochung, A. OALBX0103

Odari, E. OAB0305

Odiya, S. OAD0503

Odoyo, J. OAE0102, OAE0105

Oga, E. OAE0502

Ogale, Y. OAC0203

Ogbuagu, O. OAB0205

Ogello, V. OAC0502

Ogundehin, D. OAB0105

Ojoo, S. OAD0502

Okazaki, M. OAA0405

Okebe, J. OAE0402

Okoye, A. OAA0205

Oladimeji, P.L. OAD0203

Olete, R.A. OAD0505

Ollerton, M. OAA0303

Olugo, P. OALBX0103

Omeh, O. OAB0105

Omollo, V. OAE0102, OAE0105

Ong, J. OALBA0503, OALBC0602

Ong, J.J. OAE0103

Ongwen, P. OAE0102, OAE0105

Onyedinachi, O. OAB0105

Oorloff, J. OAE0203

Opobekova, A. OALBD0604

Opoku, J. OAD0502

Oqua, D. OAB0105

Orrell, C. OAC0104, OAE0302

Ortblad, K.F. OAE0102, OAE0105

Ortiz, D. OAE0505

Otieno, P. OAE0105

Otwoma, N. OAD0504

Ouedraogo, A. OAC0405, OAD0504

Owidi, E. OAC0502

Owolabi, A.A. OAA0304

Oyco, J.M.P. OAD0505

### P

Pagtakhan, R. OAE0504, OALBE0602

Paiardini, M. OAA0103

Palaparthy, R. OAB0104

Palma, P. OALBX0104

Panpet, P. OALBE0605

Paparini, S. OALBD0602

Paquin‐Proulx, D. OAA0402

Paredes, R. OALBX0102

Park, H. OAA0205

Passaes, C. OALBA0504

Passanduca, A. OAB0103

Patpeerapong, P. OAE0505

Paudel, M. OAB0303

Paulo, P. OALBD0605

Pavel, S. OAC0405

Pavlin, B.I. OAB0302

Paynter, H. OAC0305, OALBD0602

Pedersen, C. OAC0205

Pellegrini, K. OAA0202

Pellegrini, M. OALBA0503

Peng, A.‐T. OAB0304

Pepperrell, T. OAE0303

Perdomo‐Celis, F. OALBA0504

Perry, R. OAC0103

Peters, L. OALBB0505

Peterson, C. OAA0205

Petoumenos, K. OAB0402, OAC0102, OALBB0505

Phan, T.M. OAD0404

Phanuphak, N. OAB0303, OAC0105, OAC0404, OAE0103, OALBE0605

Phanuphak, P. OALBE0605

Phillips, A. OAE0305

Phillips, N. OAB0102

Picker, L. OAA0205

Pimenta, C. OALBX0204

Pinto, P. OAC0303

Pintye, J. OAB0305, OAC0402, OAC0403

Pinyakorn, S. OAB0303

Pisculli, M.L. OALBX0102

Piwowar‐Manning, E. OALBX0202, OALBX0203

Polizzotto, M.N. OALBB0505

Polo, R. OAD0305

Poltavee, K. OAB0303

Poonkasetwattana, M. OAE0103

Porrachia, M. OAA0104, OAA0303

Prestage, G. OAC0505

Preston, S. OALBA0503

Primbetova, S. OAD0402

Prochazka, M. OAB0302

Promsena, P. OAB0303

Prozesky, H.W. OAC0104

Puertas, M.C. OALBX0104

Pulerwitz, J. OAD0203

Putcharoen, O. OALBC0605

Puyat, J.H. OAC0202

### Q

Quinn, M. OAD0304

### R

Radusky, P. OAD0205

Rahman, A.M. OAA0402

Rajashekhar, J. OAA0203

Ramautarsing, R.A. OALBE0605

Ramgopal, M. OALBB0502

Rangaraj, A. OAE0305

Rao, A. OAC0203

Rasella, D. OAC0303

Ratnaratorn, N. OAB0303

Ratti, A. OAA0105

Rauch, A. OAB0402, OALBB0505

Reddy, K. OAE0305, OALBX0104

Reddy, K.P. OAE0302

Redzo, N. OAB0403

Reeves, R.K. OAA0403

Rhee, M. OAB0205

Richardson, C. OAA0102

Rinehart, A.R. OALBX0202, OALBX0203

Roach, M.A. OAD0103

Roan, N. OAA0104, OAA0305

Roche, M. OAA0103, OALBA0503

Roche, S.D. OAE0102, OAE0105

Rockstroh, J.K. OAB0203, OALBX0102

Roederer, M. OALBA0505

Roider, J. OALBX0104

Rojo, P. OAB0103

Rooney, J. OALBX0202, OALBX0203

Roos, E. OALBX0202, OALBX0203

Rosadiño, J.D. OAE0504, OALBE0602

Rose, S. OALBX0202, OALBX0203

Rosen, J.G. OAD0503

Rossi, P. OALBX0104

Rota, G. OAE0105

Roth, N. OAC0204

Rotheram, M. OAC0504

Rougemont, M. OALBA0504

Rowland‐Jones, S. OAB0403

Roxby, A. OALBX0205

Rozanova, Y. OALBD0604

Rozental, E. OAD0402

Rucinski, K. OAC0405, OAD0103

Rugube, C. OAD0105

Rule, J. OAC0305, OALBD0602

Rupasinghe, D. OAB0402

Rusinski, K. OAD0504

Rust, B. OAA0205

Rutstein, S. OAC0205

Ruxrungtham, K. OALBC0605

Ryom, L. OAB0402, OALBB0505

### S

Sáez‐Cirión, A. OALBA0504

Sönnerborg, A. OAB0402

Saalim, K. OAE0502

Sabasaba, A. OALBX0105

Sabirova, M. OALBD0604

Sacarlal, J. OAB0103

Sacdalan, C.P. OAB0303

Sacks‐Davis, R. OAC0204

Safrit, J. OALBA0505

Salgado, M. OALBA0504

Samji, H. OAC0202

Samo Gudo, E. OAE0304

Sanchez, J. OALBX0205

Sanchez, T. OAC0203

Sangowawa, S. OAD0203

Santangelo, S. OAA0105

Santos, C. OAC0303

Santos, T.A. OALBX0204

Sapkota, Y.R. OAE0505

Sarcletti, M. OALBB0505

Sarfo, E. OAA0404

Sarkis, S. OAA0402

Sartor, R.B. OAA0102

Schaible, U.E. OAB0403

Scheibe, A. OAC0103

Schmarje Crockett, K. OAC0302

Schmidt, H.‐M. OAE0103

Schoof, N. OAA0202

Scott, A. OAE0202

Seale, A.N. OAB0302

Seeley, J. OAD0303

Segal‐Maurer, S. OAB0205

Sekamatte, S. OAC0302

Seki, S. OAA0405

Selenic, D. OAC0304

Senter, L. OALBE0603

Shaba, P. OAD0302

Shade, S.B. OAB0405, OALBE0603

Shah, N. OALBD0603

Shahmanesh, M. OAD0303

Shakeshaft, C. OALBB0503

Shapiro, L. OAA0404

Sharma, A. OAE0505

Sheerin, D. OALBA0503

Sheira, L. OALBX0103

Shemndolwa, N. OAE0403

Sheng, Z. OAA0404

Sherr, K. OAE0304

Shikuma, C.M. OAA0303

Shoko, N. OAD0403

Short, W.R. OALBB0502

Shrestha, P. OAE0305

Shroufi, A. OAE0305

Shumskaya, N. OALBD0604

Sibanda, S. OAD0302

Siegfried, N. OALBC0602

Silani, V. OAA0105

Silva de Castro, I. OAA0402

Silva Trenkle, A.D. OAA0202

Silva, A. OAC0303

Silva, M.S.T. OALBX0204

Silva, R. OALBC0604

Silvestri, G. OALBA0505

Simata, M. OAE0104

Simmons, B. OAB0204

Simms, V. OAB0403, OAE0204

Simonson, R.B. OALBB0502

Sinchai, K. OALBE0605

Singh, N. OALBX0202, OALBX0203

Singh, V. OALBA0505

Sittikarn, S. OAE0505

Siziba, B. OALBX0202, OALBX0203

Siziba, G. OALBB0503

Sklar, P. OAB0205

Sklenovská, N. OAB0302

Smedley, J. OAA0205

Smith Ordain, N. OAE0502

Smith, A.K.J. OALBD0602

Smith, D. OAA0104, OAA0303

Smith, K. OAD0202

Smyth, G. OALBA0503

Soares, F. OAC0503

Sokhela, S. OAB0204, OALBB0504, OALBX0102

Sola, T. OAD0403, OALBE0604

Sophonphan, J. OALBC0605

Soto‐Torres, L. OALBX0202, OALBX0203

Souza, L.E. OAC0303

Spooner, E. OALBX0202, OALBX0203

Sprague, L. OAC0405, OAD0103, OAD0504

Sprenger, K. OALBX0104

Squires, K. OALBX0102

Sriplienchan, S. OAB0303

Ssekyewa, C. OAD0503

Ssemwanga, R.J. OAD0503

Stamos, J.D. OAA0402

Stancofski, E.‐S. OAA0404

Stecher, M. OAB0402

Stein, D. OAB0102

Stevenson, M. OAD0104

Stockton, M.A. OAE0502

Stoove, M. OAC0204

Storer, D. OALBD0602

Stranix‐Chibanda, L. OALBC0603

Strizzi, S. OAA0105

Strong, C. OAD0505

Strong, M. OAC0202

Su, F.‐H. OAB0203

Su, L. OAA0202

Sugandhi, N. OAE0403

Sugawara, S. OAA0403

Sun, H.‐Y. OAB0304

Sun, Y. OAA0203

Suwandi, N.S. OAE0103

Swendeman, D. OAC0504

Syarif, O. OAC0405, OAD0103, OAD0504

Sylvia, S. OAD0202

Szubert, A.J. OALBB0503

### T

Taasi, G. OAE0202

Tachiwenyika, E. OAD0105

Tafeni, S. OALBB0503

Tafuma, T.A. OAD0105

Tagarro, A. OAB0103, OALBX0104

Tamboong, E.J. OALBE0602

Tang, H.‐J. OAB0304

Tang, W. OAE0103

Tarr, P. OAB0402

Taruberekera, N. OAD0403

Tawon, Y. OAC0404

Taylor, H.E. OAA0304

Taylor‐Brill, S. OAA0103

Teeraananchai, S. OAC0105

Teeratakulpisarn, N. OAC0404, OALBE0605

Telwatte, S. OAA0302, OAA0305

Terlikbayeva, A. OAD0402

Terry, M. OAA0103

Thammajaruk, N. OAC0404

Thior, I. OAE0202

Thirumurthy, H. OALBX0103

Thitipatarakorn, S. OALBE0605

Thoueille, P. OALBA0504

Thwin, S.S. OALBC0604

Tiam, A. OAE0305

Tieosapjaroen, W. OAE0103

Ton, T. OAA0202

Torpey, K. OAE0502

Torres, T.S. OALBX0204

Tovar Sanchez, T. OALBB0504

Toyo, O. OAB0105

Trabattoni, D. OAA0105

Traeger, M. OAC0204

Tran, T.L.V. OAD0404

Tran, T.T. OAD0404

Trautmann, L. OAB0303

Triamwichanon, R. OAC0105, OALBC0605

Trichavaroj, R. OAC0404

Tucker, J. OAA0102

Tumpach, C. OALBA0503

Tumusiime, J. OAE0202

Tun, W. OAD0203

Turan, B. OAD0204

Turkova, A. OALBB0503

Turpin, G. OAC0405, OAD0103, OAD0504

Tuyishime, M. OALBA0505

### U

Uchil, P. OAA0203

Udofia, E. OAB0105

Uetela, O. OAE0304

Ullah, I. OAA0203

Umana, E. OAB0105

Unimuke, M. OAB0105

### V

Vako, N.A.J.C. OAC0405, OAD0504

Valencia Ortega, E. OALBX0102

Valencia, J. OALBX0205

van Lobenstein, J. OALBX0104

van Santen, D. OAC0204

Vanetti, C. OAA0105

Vannappagari, V. OAB0402, OALBB0505

Varco‐Merth, B. OAA0205

Vasan, S. OAB0303

Vasireddy, V. OALBD0603

Veloso, V.G. OALBX0204, OALBX0205

Venter, F. OALBB0504

Venter, W.D.F. OAB0204

Vickerman, P. OAC0103

Vieira, V. OALBX0104

Vissicchio, F. OAD0205

Vitoria, M. OALBC0604

Vjayabandara, P. OAE0203

Vo, A.V. OAD0503

Voldal, E. OALBX0202

Volny, A.A. OAB0402

von Groote, P. OAC0104

Vormawor, R. OAE0502

Vos, S. OAC0203

Vu, M.C.H. OAD0404

Vu, P.H. OAD0404

Vu, T. OAD0304

### W

Wagner, A. OAC0403

Wahl, A. OAA0102

Walker, A.S. OALBB0503

Wallace, M. OAE0302

Walmsley, S. OAB0203

Wang, H. OAB0205

Wang, N.‐C. OAB0304

Wansom, T. OAB0303

Wanyenze, R.K. OAC0302

Ward, A. OAA0204

Wareechai, P. OAC0105

Wasmuth, J.‐C. OALBB0505

Watoyi, S. OAC0403

Wedrychowski, A. OAA0302, OAA0305

Weiler, J. OAA0204

Wells, C. OAE0403

Wensing, A. OALBA0504

Were, D. OAE0102, OAE0105

Wilcock, B. OAC0505

Wilkinson, A. OAC0204

Williams, V. OAD0502

Willis, N. OAD0302

Wilton, J. OAC0202

Wirtz, A. OAD0104

Wit, F. OAB0402, OALBB0505

Wiwa, O. OAE0404

Wogrin, C. OAD0302

Wolfe, A. OAD0103

Wong, J. OAA0302, OAC0202

Wongsa, A. OALBE0605

Woodworth, B. OAA0104

Wu, L. OAC0402

### X

Xiao, D. OAB0104

Xie, H. OAD0202

Xu, P. OALBB0502

### Y

Yang, C.‐J. OAB0304

Yao, W. OAA0102

Yatina, D. OAC0205

Yekeye, R. OAD0105

Yerly, S. OALBA0504

Yin, K. OAA0104

Yogo, K. OAD0105

Yost, F. OAA0303

Young, K. OAA0104

Young, L. OAB0402

Younger, S. OAA0103

Yukl, S. OAA0302, OAA0305

Yuldashev, E. OALBD0604

### Z

Zalazar, V. OAD0205

Zang, Y. OALBX0102

Zangerle, R. OAB0402

Zar, H. OAB0102

Zeller, S. OAA0203

Zerbato, J. OALBA0503

Zewdie, K. OAC0502

Zhang, B. OAA0404

Zhang, H. OAB0104

Zhang, K. OAD0202

Zhang, L. OAE0103

Zhang, S. OAE0105

Zhang, Y. OAD0202

Zhao, R. OAD0202

Zhao, T. OAD0503

Zhou, T. OAA0404

Zhou, Y.‐P. OAB0203

Zhu, L. OAA0203

Zimmermann, M. OAE0304

